# Nutrition Interventions in the Treatment of Gastrointestinal Symptoms during Cancer Therapy: A Systematic Review and Meta-analysis

**DOI:** 10.1016/j.advnut.2025.100485

**Published:** 2025-07-22

**Authors:** Zainab Alzoubi, Brett R Loman

**Affiliations:** 1 Division of Nutritional Sciences, University of Illinois at Urbana-Champaign, Champaign, IL, USA; 2 Department of Animal Sciences, University of Illinois at Urbana-Champaign, Champaign, IL, USA; 3 Personalized Nutrition Initiative, University of Illinois at Urbana-Champaign, Champaign, IL, USA; 4 Microbial Systems Initiative, University of Illinois at Urbana-Champaign, Champaign, IL, USA

**Keywords:** chemotherapy, radiation, surgery, dietary counseling, diarrhea

## Abstract

Modern cancer therapy is effective at reducing tumor burden and extending lifespan. However, cancer therapy also induces various gastrointestinal (GI) side-effects that are dose-limiting, reduce quality of life, and potentially lead to treatment failure. Standard medical nutrition therapy for patients undergoing cancer treatment focuses on preventing weight loss and malnutrition but not reducing GI symptoms. Therefore, the objective of this study was to assess efficacy of nutrition therapy to reduce GI side-effects during cancer treatment via systematic review and meta-analysis. A systematic search was conducted in Scopus and PubMed databases. A meta-analysis was performed on articles meeting inclusion criteria to estimate the pooled effect size on GI symptoms, separated by nutrition intervention type (nutrient supplementation, oral nutrition supplement, or dietary counseling). Further subgroup analyses were conducted based on cancer type, cancer therapy, and nutrient intervention. All statistical analyses were performed in Stata/MP version 17.0 using 2-sided tests with *P* < 0.05 as the threshold for statistical significance. A total of 15,556 articles were captured by the search algorithm, and 139 studies met inclusion criteria for meta-analysis. Articles reported 12 different GI symptoms, resulting in 151 total meta-analyses across symptom, cancer treatment, and nutrition intervention subtypes. Meta analyses indicated that collectively (all interventions combined), nutrient supplementation reduced nausea, vomiting, and diarrhea incidence (all *P* < 0.001). Oral nutrition supplements had no effects on GI symptoms (all *P* > 0.05). Dietary counseling reduced constipation and diarrhea incidence. Although 9 individual nutrient supplementation interventions reduced 8 symptoms, probiotic supplementation had some of the strongest effects on abdominal pain, vomiting, and diarrhea incidence. This meta-analysis supports implementation of specific medical nutrition therapies to treat GI symptoms during cancer therapy and identifies those requiring additional investigation. Given the large variation in responses within and across studies, future experiments should explore personalized nutrition-based strategies to optimize treatment efficacy.

This study was register at PROSPERO as 549116.

**Statement of significance**

Gastrointestinal (GI) symptoms are extremely prevalent and burdensome for patients undergoing cancer treatment. Although the main concern of cancer therapy is to diminish cancer progression and recurrence, GI symptoms are often overlooked. Nutrition recommendations to patients undergoing cancer treatment generally target malnutrition without consideration for GI responses that can undermine these goals. This review encompasses oral nutrition interventions and their effect on specific GI symptoms that decrease patient quality of life and can serve as a basis for development of advanced medical nutrition therapy guidelines for clinicians providing cancer therapy.

## Introduction

Cancer treatment induces multiple gastrointestinal (GI) side-effects, with ∼81% of patients experiencing symptoms at some point during their treatment [1,2]. These symptoms vary in incidence and severity, arising from chemotherapy and radiation-induced apoptosis and tissue damage, surgery and immunotherapy-induced inflammation, alterations in endocrine signaling, and other causes [[1], [2], [3]]. The most common symptoms are nausea, vomiting, diarrhea, mucositis, and constipation, all of which reduce quality of life and induce nutrient malabsorption, anorexia, and weight loss [3,4]. Persistent and severe symptoms are dose-limiting, which may ultimately lead to treatment failure. To treat these symptoms, physicians prescribe antiemetic and antidiarrheal drugs such as serotonin receptor antagonists, opiate agonists, corticosteroids, and neurokinin-1 receptor antagonists [[5], [6], [7]]. Although this is a viable solution for some patients, increased burdens of treatment cost, treatment self-management, and limited pharmaceutical efficacy create barriers to effective symptom resolution [8].

By comparison, medical nutrition therapy (MNT) can be a more affordable and personalized solution [7]. MNT is implemented as standard of care for patients undergoing cancer treatment with the primary goals of preventing weight loss and malnutrition. Most often, the approach is to simply encourage macronutrient intake (calories) without consideration for the underlying causes of malnutrition [9]. Unfortunately, chronic nausea and diarrhea often lead to malnutrition and left untreated these symptoms undermine a calorie-oriented approach [10]. Targeted nutrient supplementation holds high therapeutic potential, as food and nutrients interact directly with the GI tract and thus play an important role in modulating gut pathophysiology [11]. Therefore, an MNT approach that addresses both nutrient requirements and GI symptoms is more likely to enhance patient outcomes.

Several MNT strategies can be implemented to reduce incidence of GI symptoms; common approaches include direct nutrient supplementation, provision of oral nutrition supplements (ONS), and dietary counseling. Supplementation of specific nutrients is the most common approach, which entails directing the patient to consume specific foods or supplements to modulate nutrient-related metabolic pathways and GI motility. This approach can be tailored to a patient’s symptoms and preferences without drastically changing their overall diet [12]. Another approach involves ONS, which generally provide complete nutrition, but can also include functional nutrients [13]. ONS is a simple but effective method to provide high nutrient-density in relatively small volumes while maintaining usual food intake in the transition from inpatient to outpatient care [13]. Finally, dietary counseling encourages patients to focus on specific goals related to their overall dietary pattern, usually through one-on-one interactions [14]. Dietary counseling allows dietitians to tailor treatment plans to patient preferences and track progress through follow-up appointments. Counseling can be a more inclusive form of MNT, as patients have variable: access to foods, food preferences, and education needed to support dietary changes [15]. Although all 3 of these approaches provide specific advantages and disadvantages to the MNT process during cancer treatment, their comparable efficacy remains undetermined.

Previous systematic reviews have explored very specific intersections of MNT, cancer treatments, and aspects surrounding GI symptoms. Systematic reviews by Allenby et al. [16], Andreou et al. [14], and Wedlake et al. [17] investigated the ability of dietary interventions to increase quality of life in patients undergoing cancer therapy, but no meta-analyses were conducted. Systematic reviews and meta-analyses by Baldwin et al. [18] and de van der Schueren et al. [19] were conducted specifically on ONS interventions and dietary counseling on inflammatory markers and GI side-effects. A systematic review by Croisier et al. [20] focused on effects of fiber on GI symptoms in individuals with gynecological cancers undergoing pelvic radiation therapy, a very narrow focus that included studies without a randomized, controlled design with no meta-analysis. Another systematic review and meta-analysis conducted by Baguley et al. [21] focused on the effect of nutrition interventions on cancer-related fatigue and overall scoring for quality of life, but not GI symptoms. Given these limitations in the existing literature, the aim of this study was to conduct a systematic review and meta-analysis assessing the efficacy of nutrition interventions to reduce GI symptoms during cancer treatment. We hypothesized that gut-targeted nutrition interventions would reduce the incidence and severity of cancer treatment-induced GI symptoms. Herein, we provide evidence that several approaches, particularly those that supplement specific nutrients, successfully reduce GI symptoms induced by cancer treatment and propose that they should be incorporated into routine MNT during cancer treatment.

## Methods

The PRISMA guidelines were used to ensure transparent reporting of the scientific evidence related to nutritional therapies investigated for relieving GI symptoms during cancer therapy [22].

### Study selection criteria

The selection of an adequate search algorithm was adapted according to the medical subject heading (MeSH) terms implemented in PubMed on 1 January, 2024. A keyword search was performed in PubMed and Scopus through all years <2024 (Supplemental Tables 1 and 2). The search algorithm included all possible combinations of the MeSH terms from the following 3 groups: *1*) cancer treatment; *2*) GI symptoms; and *3*) nutrition. Titles and abstracts of the articles identified through the keyword search were screened against the study selection criteria. Articles with 1 of the following keywords were excluded: “cross-over trial,” “parenteral nutrition,” “IV nutrient administration,” “non-human,” “time of refeeding,” “fasting,” “exercise,” “study protocols,” “suppository administration,” and “drug therapy.” Potentially relevant articles were retrieved for evaluation of the full texts. Reviewers ZA and BRL independently conducted title and abstract screening and identified potentially relevant articles. Discrepancies were resolved through discussion between the 2 reviewers [23].

Full-text screening evaluated articles against the study selection criteria. Studies that met all of the following criteria were included in the review—study design: randomized, controlled clinical trial, study participants: patients receiving cancer treatment with concurrent nutrition intervention; main outcome: GI symptom incidence or severity; article type: peer-reviewed publication; and language: English. Population, Intervention, Comparator, Outcome, Study design criteria were used to define the research question for the systematic review (Table 1). All studies included in meta-analyses utilized appropriate control groups that aligned with the specific intervention being tested (e.g., placebo, standard care, or no intervention). The information on the type of control interventions for each study is presented in Table 2 [[24], [25], [26], [27], [28], [29], [30], [31], [32], [33], [34], [35], [36], [37], [38], [39], [40], [41], [42], [43], [44], [45], [46], [47], [48], [49], [50], [51], [52], [53], [54], [55], [56], [57], [58], [59], [60], [61], [62], [63], [64], [65], [66], [67], [68], [69], [70], [71], [72], [73], [74], [75], [76], [77], [78], [79], [80], [81], [82], [83], [84], [85], [86], [87], [88], [89], [90], [91], [92], [93], [94], [95], [96], [97], [98], [99], [100], [101], [102], [103], [104], [105], [106], [107], [108], [109], [110], [111], [112], [113], [114], [115], [116], [117], [118], [119], [120], [121], [122], [123], [124], [125], [126], [127], [128], [129], [130], [131], [132], [133], [134], [135], [136], [137], [138], [139], [140], [141], [142], [143], [144], [145], [146], [147], [148], [149], [150], [151], [152], [153], [154], [155], [156], [157], [158], [159], [160], [161], [162]].

**TABLE 1:** PICOS criteria for inclusion and exclusion of studies.

Criteria	Inclusion criteria	Exclusion criteria
Population	Male and female patients of any age with cancer receiving ≥1 of the following cancer treatments: chemotherapy, radiation, immunotherapy, surgical resection, transplant, hormone therapy, stem cell transplant, or a combination	Patients in cancer remission, patients not receiving cancer therapy
Intervention	Oral nutrition or nutrition education	Pharmacological treatment, acupuncture, exercise, physical therapy, psychological therapy, complex combinations of Chinese herbs
Comparison	Compared with independent control group: placebo, isocaloric nutrition, standard of care, no treatment	Crossover design, comparison to own baseline values, no control group
Outcome	Changes to GI symptoms after intervention	No reported values for GI symptoms
Study design	Any randomized, controlled, clinical trial	Nonoriginal study or case report, crossover, non–peer reviewed article, nonrandomized trial

Abbreviation: GI, gastrointestinal.

**TABLE 2:** Basic characteristics of studies included in the review in chronological order split by treatment type.

References	Cancer Type	Cancer Treatment	Treatment Type	Age, y; Sex distribution	Study Design	Country	Nutrition Dose, Frequency, Duration	Gastrointestinal Symptom/Measurement/Effect	Sample Size
Smit et al. [31]	Malignant Melanoma	Chemotherapy	ONS	43.6±12.1; M:47.1% F:52.9%	Controlled Trial	Netherlands	4000kcal Pepti-2000	Diarrhea incidence WHO (NE)	Int=9 (N=17)
							Daily		
							3 weeks		
Ravasco et al. [25]	Colorectal Cancer	Radiotherapy	ONS	59 ± 15; M: 59.5% F: 40.5%	Prospective, Randomized, Controlled Trial	Portugal	2 cans of 20g protein total of 200kcal	Anorexia incidence PG-SGA (-); Diarrhea incidence PG-SGA (-)	Int=37 (N=74)
							Daily		
							3 months		
Ravasco et al. [24]	Head And Neck Cancer	Radiotherapy	ONS	60 ± 11; M: 80.0% F:20.0%	Prospective, Randomized, Controlled Trial	Portugal	2 cans of 20g protein total of 200kcal	Anorexia incidence (-)	Int = 25 (N=50)
							Daily		
							3 months		
Giger-Pabst et al. [27]	Gastrointestinal Cancer	Surgery: Abdomen	ONS	(64.1 ± 12.7); M: 61.1% F:38.9%	Prospective, Randomized, Double-Blind, Placebo-Controlled Study	Switzerland	750mL IEF ONS	Diarrhea Incidence (NE)	Int=55 (N=108)
							Daily		
							3 Days		
Jiang et al. [29]	Nasopharyngeal Cance	Chemotherapy: Docetaxel And Cisplatin Radiation	ONS	47.44±11.0; M:69.0% F: 31.0%	Single-Center, Prospective, Randomized	China	100g EnterNutr	Nausea Incidence CTCAE (NE), Mucositis Incidence CTCAE (NE)	Int=50 (N=100)
							Daily		
							6 weeks		
Harada et al. [28]	Oral Cancer	Chemotherapy	ONS	40-92; M: 70% F:30%	Prospective Study	Japan	1 bottle ELental	Mucositis Incidence (-)	Int=25 (N=50)
							Daily		
							7 weeks		
Toyomasu et al. [32]	Gastric Cancer	Chemotherapy	ONS	67.8; M:77.3% F:22.7%	Prospective Pilot Study	Japan	1 bottle Elental	Mucositis incidence CTCAE (-); Nause incidence/severity CTCAE (NE); Anorexia severity CTCAE (NE); Diarrhea incidence/severity CTCAE (NE)	Int=11 (N=22)
							Daily		
							28 Days		
Filipp et al. [26]	Myeloma And Lymphoma	Chemotherapy And Stem Cell Transplant	ONS	(25-77); M: 74.7% F:25.3%	Prospective, Double Blinded, 2-Arm Randomized Multi-Center Study	USA	8oz Enterade	Diarrhea incidence/severity CTCAE (NE); Nausea incidence/severity CTCAE (NE)	Int=58 (N=114)
							2x/Day		
							2 weeks		
Katada et al. [30]	Esophageal Cancer	Chemotherapy: Cisplatin, 5-Fluorouracil, Docetaxel	ONS	67.22±4.91 ; M:83.1% F:16.9%	Randomized Study	Japan	160g ELENTAL diet	Mucositis, vomiting, and constipation Incidence CTCAE (NE) Anorexia, diarrhea, nausea incidence/severity CTCAE (NE)	Int=36 (N=71)
							Daily		
							9 weeks		
Sittitrai et al. [162]	Head And Neck Cancer	Surgery	DC	56.2±9.3; M:65.5% F: 34.5%	A Randomized Controlled Trial	Thailand	Immune enhanced diet	Vomiting incidence (NE); Nausea incidence (NE)	Int=60(N=116)
							7 weeks		
Bye et al. [34]	Gynecological Malignancies	Radiation	DC	53.12±12.24; F:100.0%	Prospective Clinical Trial	Norway	40g fat	Nausea, Diarrhea, and Anorexia incidence (NE)	Int=71(N=141)
							15g Lactose		
							Daily		
							6 weeks		
Ravasco et al. [25]	Colorectal Cancer	Radiotherapy	DC	58 ± 15; M: 59.5% F: 40.5%	Prospective, Randomized, Controlled Trial	Portugal	Counseling	Anorexia and Diarrhea incidence PG-SGA (-)	Int=37 (N=74)
Ravasco et al. [24]	Head And Neck Cancer	Radiotherapy	DC	60 ± 11; M: 80.0% F:20.0%	Prospective, Randomized, Controlled Trial	Portugal	Counseling	Anorexia incidence (-)	Int = 25 (N=50)
Soto-Lugo et al. [35]	Gynecological Cancer	Surgery: Hysterectomy	DC	44.5; F: 100.0%	Single-Center, Randomized, Prospective Trial	Mexico	Low-FODMAP until end of radiation	Diarrhea, Constipation, Nausea, and Vomiting EORTC QLQ C-30 (NE)	Int=46 (N=89)
		Chemotherapy: Cisplatin							
		Radiotherapy: External Beam							
Abdollahi et al. [33]	Breast Cancer	Chemotherapy	DC	46.44 ± 10.71; F: 100.0%	Single-Center, Single-Controlled, And Randomized Trial	Iran	Diet modification:	Diarrhea, Constipation, Nausea, Vomiting, and Anorexia incidence ROM-III (-)	Int=73 (N=150)
							12–15% protein		
							30–35% fat 55–60% carbohydrates and nutrition education		
							1x/week		
							10 weeks		
Souza et al. [36]	Breast Cancer	Chemotherapy	DC	44.94±8.95; F: 100.0%	A Randomized Clinical Trial	Brazil	30 kcal/kg/day total + 1.5 g/kg/day of protein	Constipation, Diarrhea, Nausea, and Vomiting frequency EORTC QLQ (NE)	Int=19 (N=34)
							- for 42 days	Constipation, Diarrhea, Nausea, and Vomiting frequency EORTC QLQ (NE)	
							- for 63 days	Constipation, Diarrhea, Nausea, and Vomiting frequency EORTC QLQ (NE)	
							- for 21 days		
Salminen et al. [139]	Gynecological Cancer	Radiotherapy	NS	40-75; F: 100.0%	Pilot Clinical Trial	Finland	2 × 109 Lactobacillus Acidophilus	Diarrhea, Incidence (-); Anorexia Incidence (NE);	Int=11 (N=21)
							Daily		
							15 days		
Bozzetti et al. [48]	Breast Cancer	Chemotherapy: Leucovorin	NS	73.5; F: 100%	DoubleBlinded Randomized Study	Italy	10g Glutamine	Diarrhea, Incidence NCI (NE)	Int=33 (N=65)
							3x/Day		
							2 months		
Mahajan and Singh [114]	Cancer: Variable	Radiotherapy	NS	5-90; M:30.8% F: 69.2%	Randomized Prospective Study	India	100mg Vitamin B6	Anorexia, Vomiting, and Nausea Incidence (NE)	Int=52 (N=104)
							Daily		
							7 days		
Coghlin Dickson et al.									
[57]	Leukemia And Lymphoma	Transplant: Bone Marrow	NS	42.88±11.4; M:55.2% F: 44.8%	Prospective, Randomized, Double-Blinded Study	USA	30g Glutamine Daily	Mucositis incidence (NE)	Int=29(N=58)
							28 days		
Huang et al. [85]	Head And Neck Cancer	Radiation	NS	51.2 ± 10.4; M: 76.5% F: 23.5%	Pilot Randomized Trial	Taiwan	16g L-glutamine in saline (30mL) gargled for 3 min/day until 25th fraction of radiotherapy	Mucositis incidence/severity WHO (NE)	Int=8 (N=17)
Daniele et al. [59]	Colon Cancer	Chemotherapy	NS	35-76; M:58.1% F: 41.9%	A Double Blind, Two Arm, Parallel, Randomized Controlled Trial	Italy	6g Glutamine 3x/day	Diarrhea Incidence (NE)	Int=29 (N=62)
							15 days		
Asao et al. [43]	Colorectal Cancer	Surgery: Colon	NS	59.6 ± 7.7; M: 68.4% F: 31.6%	Randomized Controlled Trial	Japan	1x Gum	Flatus Latency (h) (–); Defecation Latency (h) (–)	Int= 10 (N=19)
							3x/day		
							Passage of first flatus		
Kokkonen et al. [100]	Cancer: Variable	Chemotherapy	NS	8.2±4.8; M: 50.0% F:50.0%	Prospective And Randomized Study	Finland	Vitamin A	Diarrhea, Constipation, Nausea, and Abdominal pain, incidence (NE)	Int=10 (N=20)
							-10mg/kg 3 weeks		
							-5mg/kg 3 weeks		
Ferreira et al. [72]	Oral Cavity And Oropharynx Cancer	Irradiation	NS	55.38±12.08; M: 88.9% F: 11.1%	Double-Blind Randomized Trial	Brazil	400mg ɑ-tocopherol	Mucositis incidence (-); Nausea and Vomiting Incidence (NE)	Int=28 (N=54)
							Daily		
							7 weeks		
Sieja and Talerczyk									
[144]	Ovarian Cancer	Chemotherapy	NS	51.05±12.75; F: 100.0%	Randomized And Double-Blind	Poland	100ug Selenium	Nausea, Vomiting, Abdominal Pain, Anorexia, Flatulence, and Mucositis Severity (-); Diarrhea Severity (NE)	Int=31 (N=62)
							4x/day		
							3 months		
Hirayama et al. [82]	Colorectal Cancer	Surgery: Colon	NS	58.5 ± 13.9; M: 54.2% F: 45.8%	Randomized Controlled Trial	Japan	30 min gum chewing sessions	Defecation Latency (h) (–); Flatus Latency (h) (–)	Int=10 (N=24)
							3x/day		
							Until passage of first flatus		
Li et al. [108]	Breast Cancer	Chemotherapy	NS	51.35±6.58; F: 100.0%	A Prospective Randomized trial	China	30g Glutamine	Mucositis and diarrhea Incidence, NCI (NE)	Int=30 (N=60)
							Daily		
							12 days		
Lin et al. [109]	Head And Neck Cancer	Radiotherapy	NS	50.5±11; M: 85.6% F:14.4%	Double Blind Randomized Study	Taiwan	25mg Zinc 3x/day	Mucositis Severity RTOG (-)	Int=50 (N=100)
							2 months		
Mücke et al. [124]	Gynecological Cancer	Radiation	NS	30-84; F: 100.0%	Prospective Randomized Observational Study	Germany	Sodium selenite:	Diarrhea incidence CTC (-)	Int=37 (N=77)
							-500ug day during radiation		
							-300ug day without radiation		
							Daily		
							5 weeks		
Quah et al. [130]	Colon And Rectal Cancer	Surgery: Colectomy	NS	67.5 ± 9.78; M: 65.8% F: 34.2%	Prospective Randomized Trial	United Kingdom	5min gum chewing	Flatus Latency (h) (NE); Defecation Latency (h) (NE)	Int= 19 (N=38)
							3x/day		
							Resumed solid diet		
Choi et al. [56]	Cancer: Variable	Chemotherapy: 5-Fluorouracil And Leucovorin	NS	25-67 M: 65.0% F: 35.0%	Open Label Study	Korea	10g glutamine 3x/day	Mucositits incidence (-)	Int=22 (N=51)
							15 days		
de Luis et al. [62]	Oral And Laryngeal Cancer	Surgery: Head And Neck	NS	61.8±13.3; M: 90.3% F:9.7%	Prospective Randomized Trial	Spain	17g Arginine	Diarrhea, incidence (NE)	Int=35 (N=75)
							Daily		
							16 days		
Delia et al. [63]	Sigmoid, Rectal, Cervical Cancer	Postoperative Radiation Therapy	NS	-	Double-Blind, Placebo-Controlled Trial	Italy	450 billion/g lyophilized bacteria	Diarrhea incidence (-) Defecation Frequency (-)	Int=243 (N=482)
							Daily		
							Unit end of radiation		
Gandemer et al. [74]	Cancer: Variable	Chemotherapy	NS	(5.2-18.7); M:64.0% F:36.0%	Multicenter Randomized Trial	France	20 min gum chewing	Mucositis incidence with intensive chemotherapy (-), non-intensive chemotherapy (NE)	Int=70(N=140)
							5x/day		
							3 weeks		
Giralt et al. [78]	Gynecological Cancer	Radiation: Pelvis	NS	60.15 ±12.33; F:100.0%	Multicenter, Randomized, Placebo-Controlled Nutritional Trial	Spain	96 mL liquid yogurt of 108 CFU/g probiotic administered for 5 min 3x/Day	Diarrhea incidence (NE)	Int=44(N=85)
							6 weeks		
Strasser et al. [148]	Cancer: Variable	Chemotherapy	NS	40-83; F: 68.3% M: 31.7%	Single-Center, Randomized, Double-Blind, Placebo Controlled, Two-Arm, Parallel Study	Switzerland	39g Glutamine	Nausea incidence (+); Anorexia, Vomiting, and Diarrhea Incidence CTCAE NCI (NE)	Int=21 (N=41)
							Daily		
							4 weeks		
Rashad et al. [132]	Head And Neck Cancer	Radio Chemotherapy	NS	48.2 ± 15.64; M: 77.5% F: 22.5%	Randomized Controlled Trial	Egypt	20mL honey 3x/day	Mucositis WHO (-)	Int=20 (N=40)
							5 weeks		
You et al. [156]	Head And Neck Cancer	Radiotherapy	NS	57.098 ±11.4765; M:90.0% F: 10.0%	Randomized Placebo Controlled Clinical Trial	Taiwan	0.5g Indirubin	Mucositis and Anorexia Incidence and Severity CTCAE NCI (-)	Int=11 (N=20)
							Daily		
							7 weeks		
Chitapanarux et al.									
[54]	Cervical Cancer	Radiotherapy [Pelvis] + Chemotherapy: Cisplatin (40mg/M2)	NS	(18-65); F: 100.0%	Double Blind Study	Thailand	250mg Lactobacillus	Diarrhea Incidence NCI CTC (-)	Int=32 (N=63)
							2x/day		
							7 weeks		
Li et al. [106]	Seminoma, Lymphoma	Radiotherapy, Abdomen,	NS	47.6; M: 66.7% F: 33.3%	Randomized Clinical Trial	China	300mg berberine 3x/Day	Vomiting and Diarrhea Incidence (-)	Int= 18 (N=36)
							1 week		
Li et al. [106]	Cervical Cancer	Radiotherapy, Abdomen,	NS	54.5; M: 0% F: 100%	Randomized Clinical Trial	China	300mg Berberine	Vomiting and Diarrhea Incidence (-)	Int=21 (N=42)
							3x/Day		
							2 weeks		
Mokhtar et al. [122]	Hematological Malignancies	Chemotherapy: Vincristine	NS	7.10±4.03; M: 71.3% F:28.7%	Pilot Study	Egypt	1.5g Glutamic acid	Constipation Incidence (NE)	Int= 54 (N=94)
							Daily		
							4 weeks		
Ikeguchi et al. [87]	Colorectal Cancer	Chemotherapy Variable	NS	70.45±8.17; M: 65.0% F: 35.0%	-	Japan	4.05g Fucoidan	Nausea, Diarrhea, and Mucositis incidence (NE)	Int=10 (N=20)
							Daily		
							6 months		
Liu et al. [112]	Colorectal Cancer	Surgery: Colectomy	NS	65.5± 10.45; M: 59.0% F: 41.0%	A Double-Blind Study	China	2g of 2.6 x 1014 CFU Probiotics	Defecation Latency (h) (-); Diarrhea incidence (-)	Int=50 (N=100)
							Daily		
							16 days		
Pillai et al. [129]	Bone Sarcoma	Chemotherapy	NS	8-21; M: 66.7% F:33.3%	Randomized Controlled Study	India	1,000-2,000mg depending on patient weight of ginger	Nausea and vomiting incidence/ severity (-)	Int=30 (N=60)
							Daily		
							10 Days		
Mansouri et al. [117]	Unknown	High Dose Chemotherapy; Hematopoietic Stem Cell Transplant	NS	29; M:66.7% F:33.3%	Double Blind Randomized Study	Iran	220 mg Zinc Sulfate	Mucositis Incidence / Severity WHO (NE)	Int=30 (N=60)
							2x/day		
							3 weeks		
Panahi et al. [127]	Breast Cancer	Chemotherapy	NS	51.83 ± 9.18; F: 100.0%	Pilot, Randomized, Open-Labeled, Clinical Trial	Iran	0.5g Ginger 3x/day	Nausea Incidence (NE)	Int=37 (N=78)
							4 days		
Rohr et al. [136]	Cancer: Variable	Radiation Therapy	NS	52.36±13.66; M:70.6% F:29.4%	Randomized, Double-Blind, Placebo-Controlled, Early-Phase Trial.	Germany	700 mg of soy isoflavones	Anorexia and Diarrhea Incidence (-)	Int=104 (N=168)
							Daily		
							3 months		
Sharma et al. [143]	Head And Neck Cancer	Chemotherapy	NS	51.23±9.78; M:92.5% F:7.5%	Randomized, Double-Blind, Single Center, Placebo-Controlled Study	India	2x109 of Lactobacillus Brevis	Mucositis Incidence and Severity, FACT-HN (-)	Int=86 (N=153)
							6x/Day		
							7 weeks		
Babaee et al. [45]	Head And Neck Cancer	Radiation	NS	52.5; M: 50.0% F:50.0%	Randomized Controlled Clinical Study	Iran	20g Calendula Officinalis	Mucositis Severity OMAS (-)	Int=20 (N=40)
							Daily		
							7 weeks		
Ertas et al. [69]	Gynecological Malignancies	Surgery	NS	54.05±10.69; F:100.0%	Randomized Intervention	Turkey	30 min gum sessions 3x/day	Ileus Incidence (-); Flatus Latency (h) and Defecation Latency (h) (-)	Int=74 (N=149)
							Until the use of antiemetic		
Hershman et al. [81]	Breast Cancer	Chemotherapy	NS	(26-80) F:100.0%	Randomized Double-Blind Placebo-Controlled Trial	USA	500mg Acetyl-L-Carnitine	Nausea, and Vomiting Incidence/Severity (NE)	Int=202 (n=396)
							6x/day		
							24 months		
Jahangard-Rafsanjani									
et al. [91]	Leukemia	High Dose Chemotherapy And Hematopoietic Stem Cell Transplant	NS	33.7; M: 77.8% F: 22.2%	Randomized Clinical Trial	Iran	20mcg Selenium 2x/day	Mucositis Incidence/ Severity WHO (NE)	Int=37 (N=54)
							Until 1 week post-transplant		
Kucuktulu et al. [102]	Cancer: Variable	Radiotherapy: Pelvis And Chemotherapy: 5-FU	NS	65.5; M: 69.4% F:30.6%	Intervention Study	Turkey	15g Glutamine 3x/day	Diarrhea Incidence (NE)	Int=23 (N=36)
							2 weeks		
Mahdavi et al. [115]	Rectal Cancer	Chemoradiotherapy	NS	60.25 ± 15.99; M: 58.1% F: 41.9%	Randomized Clinical Trial	Iran	3g Linoleic Acid 4x/day	Anorexia, Constipation, Nausea, and Vomiting Incidence EORTC QLQ-C30 (NE); Diarrhea Incidence EORTC QLQ-C30 (-)	Int=15 (N=31)
							6 weeks		
Sangthawan et al.									
[141]	Head And Neck Cancer	Radiation	NS	61±12.5; M: 86.8% F: 13.2%	Randomized, Double Blind, Placebo-Controlled Trial	Thailand	500mg Zinc	Mucositis Incidence (NE)	Int=72 (N=144)
							3x/day		
							5 weeks		
Valadares et al. [151]	Breast Cancer	Chemotherapy	NS	51.1; F:100.0%	A Randomized, Placebo-Controlled, Double-Blind Clinical Trial	India	2.1g of Agaricus Sylvaticus	NO P-values in publication	Int=23 (N=46)
							Daily		
							6 months		
Chattopadhyay et al.									
[52]	Head And Neck Cancer	Chemoradiotherapy	NS	(57.8±14.6); M: 71.4%	Prospective Randomized Study	India	10g Glutamine	Mucositis severity and Incidence (NE)	Int= 35 (N=70)
				F: 28.6%			Daily		
							End of Treatment		
Choi et al. [55]	Prostate Cancer	Surgery: Prostatectomy	NS	65.8 ± 7.01; M:100.0%	Prospective Randomized Study	Korea	30 min gum chewing sessions	Ileus Incidence (NE)	Int=18 (N=37)
							3x/day		
							Until the passage of flatus		
Demers et al. [64]	Pelvic Cancer	Radiation	NS	61; M:74.7% F: 25.3%	Prospective, Double Blinded Randomized Multi-Center Study	USA	1.3 billion CFU probiotics	Diarrhea Incidence WHO (NE)	Int=58 (N=114)
							2x/day		
							Until End of Treatment		
Elkerm and Tawashi									
[68]	Head And Neck Cancer	Radiation Or Chemoradiotherapy	NS	45.5; M: 55% F:45%	Pilot Study	Canada	2 g date palm pollen powder Daily	Oral Pain Severity VAS (-); Mucositis Severity OMAS (-)	Int=10 (N=200)
							29 Days		
Law et al. [105]	Breast Cancer	Chemotherapy	NS	50.2 ± 13.5 F: 100.0%	Prospective Study	Malaysia	10mL Virgin coconut oil 2x/Day	Diarrhea, Constipation, and Anorexia Severity EORTC QLQ-C30 (NE)	Int=30 (N=60)
							5 cycles of chemo		
Sanchez-Lara et al.									
[140]	Lung Cancer	Chemotherapy	NS	59.9±13.2; M: 46.7% F: 53.3%	Randomized Trial	Mexico	590 kcal EPA	Anorexia Severity EORTC-QLQC30 (-); Diarrhea Severity EORTC-QLQC30 (NE)	Int=46 (N=92)
							2x/day		
							-14 day		
							-21 day		
Arslan and Ozdemir									
[42]	Breast Cancer	Chemotherapy	NS	(49-58) F: 100%	Randomized, Controlled Trial	Turkey	500mg Ginger	Nausea, severity (-)	Int=30 (N=60)
							2x/day		
							3 days		
Dulskas et al. [159]	Colorectal Cancer	Surgery: Colon	NS	65.3±9.20; M: 53.3% F: 46.7%	Prospective, Single Center, Randomized Controlled Study	Lithuania	8g Coffee	Flatus Latency (h) (–), Defecation Latency (h) (+)	Int=30 (N=90)
							3x/day		
							Until first passage of bowel movement		
Islambulchilar et al.									
[88]	Leukemia	Chemotherapy	NS	19.2 ± 2.0; M: 56.3% F: 43.8%	Double-Blind, Placebo-Controlled Trial Study	Iran	1,000mg taurine twice 2x/day	Anorexia Incidence (NE)	Int=16 (N=32)
							6 months		
Itoh et al. [89]	Cervical Cancer	Chemoradiotherapy	NS	(30-72); F:100.0%	A Randomized, Double-Blind Pilot Trial	Japan	1g Hydrolyzed Rice Bran	Diarrhea and Nausea severity (NE)	Int=7 (N=14)
							3x/day		
							6 weeks		
Kobayashi et al. [99]	Colorectal Cancer	Surgery: Colon	NS	67.22 ± 11.78; M: 60.4% F: 39.6%	Randomized Clinical Trial	Japan	5 min gum chewing 3x/day until passage of first flatus	Flatus Latency (h) (NE); Defecation Latency (h) (NE)	Int=21 (N=43)
Liu et al. [111]	Colon Cancer	Surgery : Colectomy	NS	62.86±17.19; M:52.2% vs 47.8%	A Double-Center And Double-Blind Randomized Clinical Trial	China	2g/day of 2.6 × 1014 CFU Probiotics for 16 days	Defecation Latency (h) (-); Diarrhea incidence (-)	Int=66 (N=134)
Mego et al. [119]	Colorectal Cancer	Irinotecan	NS	42-81; M:56.5% F: 43.5%	A Randomized Double Blind, Placebo-Controlled Pilot Study	Slovakia	3 capsules of 10 × 109 CFU probiotics	Diarrhea and Bloating Incidence CTCAE (NE)	Int=23 (N=46)
							Daily		
							12 weeks		
Eghbali et al. [67]	Leukemia	Chemotherapy	NS	8.5 ± 2.5; M: 50.7% F:49.2%	Single Center, Randomized, Controlled Trial	Italy	30 min sessions of gum chewing	Mucositis Incidence WHO (-)	Int=65 (N=130)
							6x/day		
							15 days		
Garcia-Peris et al.									
[75]	Gynecological Cancer	Radiation: Abdominal The Total Prescribed Dose Was 52.2 Gy.	NS	60.3±11.8; F:100.0%	A Randomized, Double-Blind, Placebo-Controlled Trial	Spain	6g Fiber	Defecation Frequency (NE)	Int=20(N=38)
							Daily		
							4 weeks		
Jayalekshmi et al.									
[92]	Head And Neck Cancer	Radiotherapy	NS		Randomized Controlled Trial	India	15mL topical honey	Mucositis Incidence RTOG (-)	Int=14 (N=28)
							Daily		
							6 weeks		
Mansouri-Tehrani									
et al. [118]	Cancer: Variable	Radiation	NS	(62 ± 14.8); M:67.4% F:32.6%	A Randomized, Placebo-Controlled Study	Iran	2 capsules LactoCareO	Diarrhea Severity (-); Defecation frequency (-); Bloating Incidence (-)	Int=22 (N=46)
							Daily for		
							4 weeks		
Mutluay Yayla et al.									
[125]	Cancer: Variable	Chemotherapy	NS	NA; M: 36.7% F: 63.3%	Randomized Controlled Trial	Turkey	30 s rinse of 15mL sage-tea-thyme hydrosol 4 times/day for 2 wk.	Mucositis WHO (-)	Int=30 (N=60)
Tsuchiya et al. [150]	Gastrointestinal Cancer	Chemotherapy	NS	63.3±8.6; M:60.3% F: 39.7%	Prospective Randomized Trial	Japan	700mg cystine + 280mg theanine	Mucositis, Anorexia, Abdominal Pain, and Nausea incidence CTCAE (NE) Diarrhea Incidence CTCAE (-)	Int=32 (N=63)
							Daily		
							35 Days		
Zou et al. [158]	Leukemia	Chemotherapy	NS	45.8 ± 11.7; M: 55.0% F: 45.0%	Randomized, Controlled Trial	Taiwan	100g Sweet Potato	Constipation incidence ROM III (-); Defecation	
Latency (h) (–)	Int=57 (N=120)								
							2x/day		
							Until 5-day post-chemo		
Bossi et al. [47]	Lung, Head, And Neck Cancer	Chemotherapy: Cisplatin (Single Dose >50 Mg/M2)	NS	59.15 ±10.18 M: 65.6%, F:34.4%	Randomized, Double-Blind, Placebo-Controlled, Multicenter Study	Italy	160mg Ginger	Nausea Incidence VAS (NE)	Int=121 (N=244)
							Daily		
							673 days		
Faramarzi et al. [70]	Rectal Cancer	Chemoradiotherapy	NS	60.2± 15.9; M: 58.1% F: 41.9%	Randomized Clinical Trial	Iran	1000mg capsules of 3g Conjugated Linoleic Acid	Anorexia and Constipation Severity QLQ C-30 (NE); Diarrhea Severity QLQ C-30 (-)	Int=16 (N=33)
							4x/ day		
							6 weeks		
Ge et al. [76]	Gastric Cancer	Laparoscopic Surgery	NS	63.1 ± 12.3; M: 60% F:40%	Randomized Controlled Trial	China	15 min gum chewing sessions	Flatus Latency (h) (NE); Defecation Latency (h) (NE)	Int =38 (N=75)
							3 x/day		
							1 week		
Gholizadeh et al. [77]	Leukemia	Chemotherapy	NS	(31 ± 38.3); M: 61.4% F: 38.6%	Double-Blind, Randomized, Placebo Controlled	Iran	220mg Zinc	Mucositis Incidence WHO (-)	Int=70 (N=140)
							3x/day		
							4 weeks		
Gungordük et al. [79]	Gynecological Cancer	Surgery: Hysterectomy	NS	35.65 ± 8.45; F: 100.0%	A Randomized Controlled Trial	turkey	100g of coffee 3x/day	Flatus Latency (h) (–); Defecation Latency (h) (–); Ileus Incidence (-)	Int=58 (N=114)
							until time to first defecation		
Hashemipour et al.									
[80]	Leukemia Or Breast Cancer	Chemotherapy	NS	37.8 ±3.5 vs 38.4±2.7; M: 38.3% F: 61.7%	Double-Blind Randomized Clinical Trial	Iran	2600mg Omega-3	Mucositis Incidence WHO (-); Oral Pain Severity (-)	Int=30 (N=60)
							2/day		
							3 weeks		
Kim et al. [97]	Ovarian Cancer	Chemotherapy	NS	54.4±11.1; F:100.0%	A Randomized, Double Blind, Placebo-Controlled Trial	Korea	1000mg red ginseng	Nausea Incidence CTCAE (NE)	Int=15 (N=30)
							2x/day		
							3 months		
Kooshyar et al. [101]	Leukemia	Chemotherapy	NS	33±15.9; M: 60% F: 40%	Double Blind Placebo-Controlled Trial	Iran	250mg Quercetin 2x/day	Mucositis Severity WHO (NE)	Int=10 (N=20)
							Start to End of Chemotherapy		
Mocellin et al. [120]	Gastrointestinal Cancer	Chemotherapy	NS	54; M:55.6% F: 44.4%	A Triple Blind, Randomized Clinical Trial	Brazil	1.55g DHA+EPA	Anorexia and Constipation Severity EORTC-QLQ C-30 SD (-); Diarrhea Severity EORTC-QLQ C-30 SD (NE)	Int=28 (N=56)
							2x/day		
							9 weeks		
Motoori et al. [123]	Esophageal Cancer	Chemotherapy	NS	(63.9 ± 7.6); M: 91.8% F:8.2%	Open-Labeled Randomized Prospective Clinical Trial In A Single Center	Japan	1x 108	Diarrhea Incidence (-); Mucositis Incidence (NE)	Int=30 (N=61)
							living Bifidobacterium breve strain Yakult (B. breve strain Yakult) and		
							1x 108		
							living Lactobacillus casei strain Shirota (L. casei strain Shirota)/g, and galacto-oligosaccharides		
							Daily		
							6 weeks		
Zhao et al. [157]	Gastric Cancer	Surgery	NS	64.54±10.9; M:51.7% F:48.3%	Prospective Randomized-Controlled Trial.	China	30g fiber	Flatus Latency (h) (-); Abdominal pain Incidence (NE); Diarrhea incidence (-)	Int=40 (N=80)
							Daily		
							4 Days		
Aghamohammadi									
et al. [37]	Head And Neck Cancer	Radiotherapy And Chemotherapy	NS	61.02 ± 15.49; M: 71.2% F: 28.8%	A Randomized Double-Blind Clinical Trial	Iran	136mg zatarial multiflora 3x/day	Mucositis Severity WHO (-); Oral Pain Severity VAS (-)	Int=25 (N=52)
							6 weeks		
Charalambous et al.									
[51]	Head And Neck Cancer	Radiation	NS	61.53; M: 72.2% vs F: 27.8%	Randomized Controlled Trial	Cyprus	20mL thyme honey mixed in 100 mL water rinses for 15 min three times a day for 7 wk.	Mucositis Severity RTOG (-)	Int= 32 (N=64)
de Loera-Rodríguez									
et al. [61]	Cervical Cancer	Chemotherapy And Radiotherapy	NS	50.9±14.7; F:100.0%	Randomized, Double-Blind, Controlled Trial	Mexico	60g Symbiotics	Diarrhea and Constipation Incidence Bristol stool form scale (NE); Nausea and Vomiting Incidence NIC (-)	Int=35 (N=70)
							Daily		
							- 4 weeks		
							- 7 weeks		
Du et al. [65]	Central Nervous System Cancer	Craniospinal Irradiation	NS	(1.3-15.5); M:67.5% F:32.5%	Intervention	China	1 capsule Bacillus Licheniformis by ZSC	Diarrhea, Nausea, Vomiting, and Abdominal Pain Incidence CTCAE (-)	Int=80(N=160)
							3x/Day		
							End of Radiation		
Lages et al. [104]	Head And Neck Cancer	Surgery	NS	60.5 ± 11.12; M: 80.6% F:19.4%	Double-Blind, Randomized Trial	Brazil	1×109 CFU, 6 g fructooligosaccharides	Abdominal Pain Incidence (NE)	Int=18 (N=36)
							2x/Day		
							1 week		
Li et al. [107]	Lung Cancer	Cisplatin (Chemotherapy)	NS	57.49±7.53; M: 71.4% F: 28.6%	Randomized, Double-Blind, Placebo-Controlled Clinical Trial,	China	0.5g Ginger Root	Nausea and Vomiting Incidence (NE)	Int=71 (N=140)
							2x/day		
							5 days		
Lustberg et al. [113]	Breast Cancer	Aromatase Inhibitor	NS	59.5 ± 8.1; F: 100.0%	Randomized PlaceboControlled Pilot Trial	USA	4.3g of omega 3	Bloating and Diarrhea Incidence (NE); Abdominal Pain, Flatulence, Nausea, and Vomiting Severity FACT-ES (NE)	Int=22 (N=44)
							Daily		
							24 weeks		
Rambod et al. [131]	Leukemia	Chemotherapy	NS	36.73 ± 15.66; M: 56.6% F:43.4%	Randomized Controlled Trial	Iran	50mg zinc sulfate	Mucositis Severity (-)	Int=36 (N=72)
							Daily		
							2 weeks		
Solís-Martínez et al.									
[146]	Head And Neck Cancer	Surgery	NS	59±13.9; M:54.7% F: 45.3%	Randomized Single-Blind Placebo-Controlled Clinical Trial	Mexico	2g EPA	Diarrhea and Anorexia Severity QLQ-C30 (NE)	Int=32 (N=64)
		Radiotherapy					Daily		
		Chemotherapy					4 weeks		
		Combined							
Xie et al. [155]	Gastric Cancer	Surgery	NS	67.795±9.873; M: 47.9% F:52.1%	Prospective Randomized Controlled Trial	China	Probiotics	Diarrhea, incidence (-); Vomiting incidence (NE)	Int=70 (N=140)
							3x/day		
							1 wk post-treatement		
Aredes et al. [41]	Cervical Cancer	Chemotherapy	NS	44.53 ± 8.73 F: 100.0%	A Randomized, Triple-Blind, Clinical Trial	Brazil	2.5 g ѡ-3	Anorexia and Nausea Incidence PGSGA (-), Vomiting, Diarrhea, and Constipation Incidence PGSGA (NE)	Int=20 (N=40)
							Daily		
							45 days		
Camargo et al. [49]	Gastrointestinal Cancer	Chemotherapy	NS	(41-63); M: 52.9% F:47.1%	Randomized, Triple-Blind, Placebo-Controlled Clinical Trial	Brazil	1.55g ѡ-3	Constipation, Diarrhea, Nausea, Mucositis, and Anorexia Incidence CTCAE (NE),	Int=26(N=51)
							2x/day		
							9 weeks		
de la Rosa Oliva et al.									
[60]	Breast Cancer	Chemotherapy	NS	50.1 ± 2.17; F: 100%	Randomized, Controlled, Double-Blind Clinical Trial	Mexico	2.4g omega-3	Nausea, Diarrhea, Vomiting, ad Mucositis Incidence (NE)	Int=26 (N=52)
							Daily		
							6 months		
Huang et al. [84]	Head And Neck Cancer	Radiation	NS	52.4 ± 9.8 ; M: 92.2% F: 7.81%	Randomized, Double-Blind, And Controlled Clinical Trial.	Taiwan	10g L-glutamine	Mucositis severity CTCAE (-)	Int=31 (N=64)
							Daily		
							2 weeks		
Jiang et al. [93]	Nose And Pharynx Cancer	Chemoradiotherapy	NS	50.89 ± 10.05; M: 62.4% F: 37.6%	Randomized, Double-Blind, Placebo, Controlled Trial	Spain	3 capsules of probiotics Bifidobacterium longum, Lactobacillus lactis, and Enterococcus faecium from Shanghai Since Pharmaceutical	Mucositis Incidence/Severity CTCAE (-)	Int=58 (N=93)
							Daily		
							7 weeks		
Phutsisen et al. [128]	Gynecological Cancer	Surgery	NS	54.6 ± 13.9; F: 100.0%	Randomized Controlled Trial	Thailand	3g Cassia Alata	Ileus Incidence (NE)	Int= 45 (N=90)
							Daily		
							Until passage of first flatus		
Reyna-Figueroa et al.									
[134]	Leukemia	Chemotherapy	NS	10.75; M:63.3% F:36.7%	Randomized Pilot Study	Mexico	5×109 CFU of lactobacillus rhamnosus	Diarrhea Incidence (NE); Nausea and Constipation Incidence (-)	Int=30(N=60)
							2x/day for		
							1 week		
Tian et al. [160]	Lung Cancer	Chemotherapy	NS	55.5± 8.6; M: 58.8% F: 41.2%	Prospective Randomized Double Blind	China	420 mg C. butyricum	Nausea and Vomiting incidence CTCAE (NE); Diarrhea incidence CTCAE (-)	Int=25 (N=51)
							3x/day		
							3 weeks		
Anandhi et al. [40]	Oral Cancer	Radiotherapy And Chemotherapy: Cisplatin	NS	56.75 ± 6.5; M: 16.7% F: 83.3%	DoubleBlinded Randomized Controlled Study	India	150mg zinc sulfate	Mucositis Incidence Radiotherapy Oncology Group scoring criteria for acute radiation toxicity (-)	Int=60 (N=120)
							Daily		
							6 weeks		
Jafarimanesh et al.									
[90]	Breast Cancer	Chemotherapy	NS	10.65± 50.47; F: 100.0%	Randomized Controlled Trial	Iran	40 drops of peppermint in 20cc distilled water	Nausea, Vomiting, and Anorexia Severity VAS (-)	Int=42 (N=84)
							8 hours		
							Until Post-chemotherapy		
Kobayashi et al. [98]	Colorectal Cancer	Chemotherapy: Mfol-FOX6	NS	62.8 ± 2.2; M: 39.3% F:60.7%	Prospective Randomized Trial	Japan	700mg Cystine and 280mg Theanine	Constipation, Diarrhea, and Nausea, Incidence CTCAE (NE); Flatus Latency (h) (NE)	Int=14 (N=28)
							Daily		
							12 weeks		
Laali et al. [103]	Head And Neck Cancer	Radiotherapy	NS	53.44; M:70.4% F: 29.6%	Double Blind Placebo-Controlled Trial	Iran	200mcg selenium	Mucositis Incidence / Severity WHO (NE)	Int=33 (N=67)
							2x/Day		
							Until end of radiotherapy		
Rathe et al. [133]	Leukemia	Chemotherapy	NS	1-15; M: 51.6% F: 48.4%	Randomized, Double-Blind, Placebo-Controlled Trial	Denmark	Bovine Colostrum g for body weight:	Diarrhea Incidence/severity, Abdominal pain Incidence, and Mucositis Severity NCI-CTCAE (NE)	Int=30 (N=62)
							-7.5g for 0-15kg		
							-15g for 15.1-30kg		
							-22.5g for 30.1-45kg		
							-30g for >45kg		
							Daily		
							29 days		
Soltani et al. [147]	Head And Neck Cancer	Radiation	NS	56.47 ± 15.91; M: 68.2% F: 31.8%	Randomized, Double Blind, Placebo Controlled Clinical Trial	Iran	7.5 cc Plantago major	Mucositis Severity WHO (-); Oral Pain Severity VAS (-)	Int=22 (N=44)
							Daily		
							7 weeks		
Widjaja et al. [154]	Leukemia	Chemotherapy	NS	6.09 ± 3.70; M: 64.6% F:35.4%	Randomized Control Trial	Indonesia	400mg/kg Glutamine	Mucositis Severity WHO (-)	Int=24 (N=48)
							Daily		
							14 Days		
Al-Taie and Koseoglu									
[39]	Colon Cancer	Chemotherapy: 5 Fluorouracil	NS	50.57 ± 9.73; M: 51.7% F: 48.3%	Prospective Randomized Controlled Study	Turkey	10g glutamine suspension	Mucositis Incidence WHO (-)	Int=30 (N=60)
							3x/Day		
							3 months		
Aziz et al. [44]	Gastric Carcinoma	Chemotherapy: FLOT	NS	Variable, M:52.4% F:47.6%	Intervention Study	Egypt	2 g ѡ-3	Diarrhea, Nausea, and Vomiting Incidence CTCAE (-)	Int=21 (N=42)
							Daily		
							1 year		
Fukaya et al. [73]	Esophageal Cancer	Chemotherapy: Cisplatin, 5-Fluorouracil	NS	(44-77); M: 88.1% F: 11.9%	Randomized Controlled Trial	Japan	80mL Yakult + 100mL MILMIL-S + 15g Oligomate Daily	Anorexia Incidence (-); Nausea, Vomiting, Diarrhea, Constipation, and Mucositis Incidence (NE)	Int= 20 (N=42)
							7 days post 2nd cycle chemotherapy		
Kia et al. [96]	Head And Neck Cancer	Chemotherapy	NS	NA; M: 56% F: 44%	Randomized Clinical Trial	Iran	80mg Curcumin 2x/Day	Mucositis Severity WHO (-)	Int=25 (N=50)
							7 weeks		
Liu et al. [110]	Gastric Cancer	Surgery: Gastrectomy	NS	58.70 ± 8.65; M: 71.4% F: 28.5%	Randomized Controlled Trial	China	5g green tea	Flatus Latency (h) (–); Defecation Latency (h) (–)	Int=38 (N=77)
							Daily		
							Until Hospital discharge		
Oshvandi et al. [126]	Cancer: Variable	Chemotherapy	NS	46.22±2.36; M: 53.2% F: 46.8%	Randomized Controlled Trial	Iran	2 min gargline of 7.5mL zinc chloride	Mucositis Severity (-)	Int=48 (N=96)
							2x/Day		
							3 weeks		
Rodríguez-Padilla									
et al. [135]	Colorectal Cancer	Surgery: Ileostomy	NS	41-81; M: 69.6% F: 30.4%	Conducted A Prospective, Randomized, Multicenter, Double-Blind Experimental Study	Spain	4.5 × 1011 probiotics	Ileus incidence (NE)	Int=34 (N=69)
							Daily		
							3 days		
Rosli et al. [137]	Pelvic Cancer	Radiation Therapy	NS	56.2 ± 10.93; M:26.7% F: 73.3%	Randomized Controlled Trial	Malaysia	10g PHGG	Diarrhea frequency (days) (+); Diarrhea NCI-CTC grade (NE); Diarrhea Bristol stool chart grade (NE)	Int=11 (N=23)
							Daily		
							28 Days		
Carr et al. [50]	Hematopoietic Malignancies	Chemotherapy + Stem Cell Transplant [No Dose Description]	NS	(41-64); M:60.0% F:40.0%	Double-Blind Randomized, Placebo-Controlled Feasibility And Pilot Study	New Zealand	1g Vitamin C	Diarrhea 4-point Likert Scale (NE), Appetite loss 4-point Likert Scale (NE), Constipation 4-point Likert Scale (NE), Nausea/Vomiting EORTC QLQ-C30(NE)	Int=10 (N=20)
							2x/day		
							28 days		
Fernandes et al. [71]	Head And Neck Cancer	Radiation	NS	58.7 ±(9.9); M: 75% F: 25%	Randomized, Double-Blind, And Controlled Clinical Trial.	Brazil	30mL of 0.8% BOP	Oral mucositis WHO (-); Dysgeusia WHO (-); Dysphagia WHO (-)	Int= 32 (N=60)
							6 x/day		
							6 weeks		
Hsu and Szu [83]	Colorectal Cancer	Surgery: Abdomen	NS	58.85 ±9.46; M: 58.3% F: 41.7%	Randomized Clinical Trial	Taiwan	15 min gum chewing sessions	Time to first defecation (NE); Time to first flatus (-)	Int=30 (N=60)
							3x/day		
							Until passage of first flatus		
Karabey et al. [94]	Cancer: Variable	Chemotherapy: Methotrexate	NS	NA; M: 50.0% F:50.0%	Pilot Study	turkey	60 sec gargling of 5 mL of black mulberry extract with 9% fruit sugar	Mucositis RTOG (-)	Int=20 (N=40)
							Daily		
							15 days		
Manifar et al. [116]	Oral Cancer	Radiotherapy	NS	50.58±7.04 ; M:71.9% F: 28.1%	Double Blind Randomized Study	Iran	2x1010 CFUs Bifdobacterium breve, 7× 109	Mucositis WHO (-)	Int=32 (N=64)
							CFU		
							Bifdobacterium longum, 2 × 109		
							CFU Lactobacillus acidophilus, 7 × 109		
							CFU Lactobacillus casei, 2 × 108		
							CFU		
							Lactobacillus bulgaricus, 1.5 × 109		
							CFU Lactobacillus		
							rhamnosus, 1.5× 1010 CFU Streptococcus salivarius subspthermophiles) and 40 mg fructooligosaccharide as a prebiotic, lactose, magnesium stearate, and talc as carrier substances 3x/day		
							6 weeks		
Mohammadi et al.									
[121]	Cancer: Variable	Chemotherapy	NS	46.43 ± 2.42; M: 58.3% F: 41.7%	Randomized Controlled Trial	Iran	2 min gargling of 7.5mL zinc chloride	Mucositis WHO (-)	Int=48 (N=96)
							2x/Day		
							2 weeks		
Sim et al. [145]	Gastrointestinal Cancer	Chemotherapy	NS	64.58 ± 2.11; M: 80% F:20%	Randomized Controlled Trial	Korea	200mL omega 3	Anorexia, Constipation and Diarrhea Severity EORTC-QLQ (NE)	Int=22 (N=40)
							2x/day		
							8 weeks		
Werida et al. [153]	Breast Cancer	Chemotherapy	NS	48.875±8.49; F: 100.0%	A Randomized Controlled Trial	Egypt	600mg alpha-lipoid acid	Abdominal pain and Nausea Incidence NCI-CTCAE (NE)	Int=32 (N=64)
							Daily		
							6 months		
Al-Kharabsheh et al.									
[38]	Colorectal Cancer	Surgery: Colon	NS	52.43; M: 52.7% F: 47.3%	Randomized Controlled Trial	Jordan	1 hr Gum chewing	Flatus Latency (h) (–)	Int= 60 (N=129)
							Every 8 hours		
							Until passage of first flatus		
Badr et al. [46]	Leukemia	Chemotherapy: Intensive [High Dose]	NS	10.0 ± 4.06 M: 50% F: 50%	Single Blind Randomized Controlled Trial	Lebanon	1 min gargling of 2.5 cc honey gargling followed by swallowing.	Mucositis Severity WHO (-); Oral Pain Severity VAS (-)	Int= 17
							Daily		Int=13
							1 week		(N=42)
Badr et al. [46]	Leukemia	Chemotherapy: Intensive [High Dose]	NS	10.0 ± 4.06 M: 50% F: 50%	Single Blind Randomized Controlled Trial	Lebanon	1 min gargling 2.5 cc olive oil gargling followed by swallowing.	Mucositis Severity WHO (-); Oral Pain Severity VAS (-)	Int= 17
							Daily		Int=13
							1 week		(N=42)
Chen et al. [53]	Lung Cancer	Chemotherapy	NS	(61.1 ± 9.02); M: 91.7% F: 8.3%	Randomized Controlled Trial	China	2.5g JK5G postbiotics	Anorexia, Constipation, and Diarrhea Severity EORCT QLQ-C30 (NE).	Int= 30 (N=60)
							3x/day		
							12 weeks		
Crichton et al. [58]	Cancer: Variable	Chemotherapy	NS	59± 8; M: 32% F:68%	Multicenter, Double-Blind, Placebo-Controlled Randomized Trial	Australia	1.2g ginger root	Nausea Incidence/ Severity (-); Vomiting Incidence (-)	Int=51 (N=103)
							4x/day		
							1 Week		
Eghbali et al. [66]	Leukemia	Chemotherapy	NS	8.15± 2.13; M: 55.7% F: 44.3%	Double-Blind Randomized Clinical Trial	Iran	5×109 CFU of LactoCare 2x/day	Diarrhea Incidence (-); Constipation, Vomiting, and Nausea Incidence (NE)	Int=54 (N=106)
							1 week		
Hussein et al. [86]	Liver Cancer	Chemotherapy	NS	>30	Double-Blind Clinical Experiment	Iraq	3 g omega 3	Nausea Incidence (-); Vomiting and Diarrhea incidence (NE)	Int=28 (N=56)
							Daily		
							6 weeks		
Khazaei et al. [95]	Breast Cancer	Chemotherapy	NS	52.3 ± 11.9; F: 100%	Randomized, Placebo-Controlled Double-Blind Clinical Trial	Iran	1×109 CFU, Lactobacillus rhamnosus, Lactobacillus casei, Lactobacillus	Anorexia Severity (NE)	Int=34 (N=67)
							acidophilus, Lactobacillus bulgaricus, Bifidobacterium		
							breve, Bifidobacterium longum, Lactobacillus helveticus,		
							Lactobacillus lactis, Lactobacillus paraplantarum, Bifidobacterium bifidum, Streptococcus thermophilus and		
							Lactobacillus gasseri and 21 g fructooligosaccharides		
							2x/day		
							8 weeks		
Sahebnasagh et al.									
[138]	Head And Neck Cancer	Radiotherapy	NS	66.33 ± 10.53; M: 54.5% F; 45.5%	Double Blind Randomized Clinical Trial	Iran	60 s rinse of 5mL 1% zinc sulfate solution	Mucositis Severity WHO (-)	Int=17 (N=33)
							3x/Day		
							7 weeks		
Shah et al. [142]	Leukemia And Lymphoma	Intensified Chemotherapy	NS	7.5; NA	Randomized Double-Blind Placebo-Controlled Trial	India	1mg/kg Zinc	Mucositis Incidence / Severity WHO (NE)	Int=44 (N=90)
							Daily		
							2 weeks		
Thomas et al. [149]	Head And Neck Cancer	Radiation	NS	57.62 ± 11.62; M; 75% F: 25%	Randomized Controlled Trial	India	10mL rinse of turmeric	Mucositis Incidence WHO (-)	Int=46 (N=92)
							3x/day		
							7 weeks		
Wei et al. [152]	Lung Cancer	Chemotherapy	NS	59.5 ± 8.04; M: 69.2% F:30.8%	Randomized Placebo Controlled	China	2g Yiga Bio-technology probiotics	Anorexia, Constipation, and Diarrhea Incidence (-)	Int=42 (N=91)
							2x/day		
							2 chemo cycles		
Yanagimoto et al.									
[161]	Pancreatic Cancer	Chemotherapy	NS	67.0±9.61; M: 53.1% F: 46.9%	Randomized, Double Blind, Placebo Controlled Clinical Trial	Japan	6g Lentinula edodes mycelia extract	Diarrhea Incidence (NE)	Int=45 (N=84)
							Daily		
							9 weeks		

For effect on measurable outcomes, a positive or increased effect is denoted by (+), a negative or decreased effect is denoted by (-), and no effect is denoted by (NE).

Abbreviations: CFU, colony-forming unit; DC, dietary counseling; EORTC-QLQ; GI, gastrointestinal; NS, nutrient supplementation; ONS, oral nutrition supplements; PG-SGA, Patient-Generated Subjective Global Assessment; RTOG, Radiation Therapy Oncology Group; FACT-HN, Functional Assessment of Cancer Therapy – Head & Neck; FACT-ES, Functional Assessment of Cancer Therapy – Esophagitis.

Any articles containing the following were excluded: inappropriate control group, retrospective study, only data reported exceeded 2 wk after completion of cancer therapy or intervention, language other than English, not enough studies reporting this symptom to conduct a meta-analysis, no reporting of scaling system used, and dose of intervention not reported.

### Data extraction

A standardized data extraction form was used to collect the following methodological and outcome variables from each included study: author(s), publication year, study design, treatment type (chemotherapy, radiation therapy, surgical therapy, bone marrow transplant, and hormone therapy) sample size, participant characteristics (i.e., sex, age, and country), GI symptoms, and treatment effect (i.e., increase, decrease, or neutral change on physiological parameters). GI symptoms were assessed through the parameters identified during the data extraction process, including presence or severity of nausea, diarrhea, vomiting, constipation, ileus, flatulence, mucositis, abdominal pain, anorexia, and bloating, as well as flatus latency, defecation latency, or defecation frequency. Data were reported as either mean and standard deviation for continuous symptom data, or presence and absence for symptom incidence. Emails were sent out to authors of articles to request missing or unreported data. Articles with unresponsive authors were excluded from the analysis if no replies were received before manuscript submission.

### Quantitative data synthesis

A meta-analysis was performed to estimate the pooled effect size on GI symptoms, assessed by incidence and severity report. A priori subgroup analyses by treatment type (nutrient supplementation, ONS, or counseling) and GI side-effects were performed for all included studies where data from ≥3 studies were available. Study heterogeneity was assessed using the I2 index. The level of heterogeneity represented by I2 was interpreted as modest (I2 < 25%), moderate (25% < I2 < 50%), substantial (50% < I2 < 75%), or considerable (I2 > 75%). A fixed-effect model was estimated when modest to moderate heterogeneity was present, and a random-effect model was estimated when substantial to considerable heterogeneity was present. For meta-analyses that included 10 or fewer studies, a fixed-effect model was used by default, as the estimation of between-study variance in random-effect models is considered unreliable with a limited number of studies [163]. Although heterogeneity across studies was expected due to differences in intervention dose, treatment duration, and cancer type, the risk of inaccuracy from heterogeneity was considered more important [164,165]. Publication bias was assessed by a visual inspection of the funnel plot and Begg’s and Egger’s tests [166], as well as by the Cochrane bias assessment tool [167]. All statistical analyses were conducted using the Stata/MP 17.0 SE version (StataCorp). All analyses used 2-sided tests, and *P* < 0.05 was considered statistically significant. A log-odds ratio was used to analyze dichotomous outcomes reflected in the supplementary figures, which was then converted to an odds risk ratio (ORR) and reported as such in the summary graphs. A Hedge’s *G* was used to analyze severity which is a continuous outcome. The indicative effect size for the interpretation of Hedge’s G was defined as: small effect 0.20, medium effect 0.50, and large effect 0.80 [168]. Graphs were generated using GraphPad Prism version 10.0.0.

### Risk-of-bias assessment

Publication bias was assessed on meta-analyses conducted with 10 or more studies [166] based on symmetry of the funnel plots and Begg’s and Egger’s test where *P* < 0.05 indicated high risk of bias. Cochrane’s risk-of-bias tool was used to evaluate overall bias and within-study bias of the included studies through the evaluating 6 components: random sequence generation, allocation concealment, blinding of both participants and personnel, binding of outcome assessment, incomplete outcome data, and selective reporting. The bias was evaluated as high (presence of bias), low (no presence of bias), or unsure (no mention of the component within the individual study) [167].

## Results

### Study selection

**FIGURE 1:**
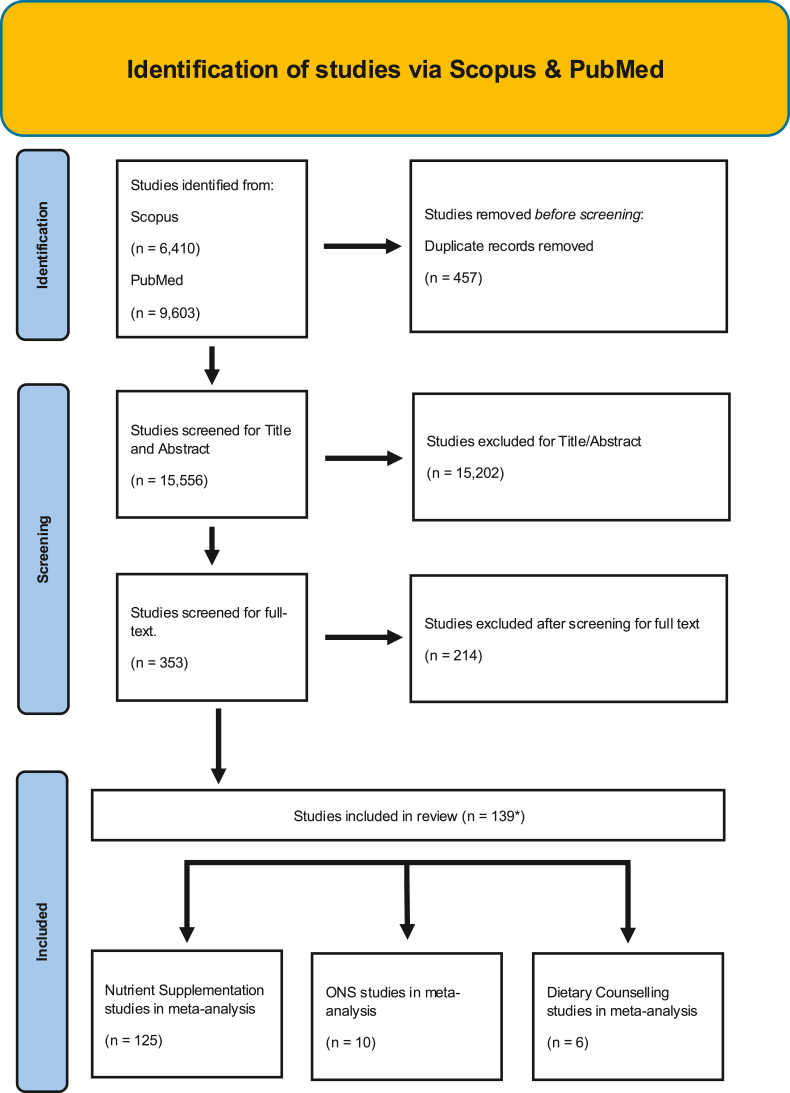
Study selection flow chart. ∗Two studies investigated both ONS and dietary counseling. ONS, oral nutrition supplementation.

**FIGURE 2:**
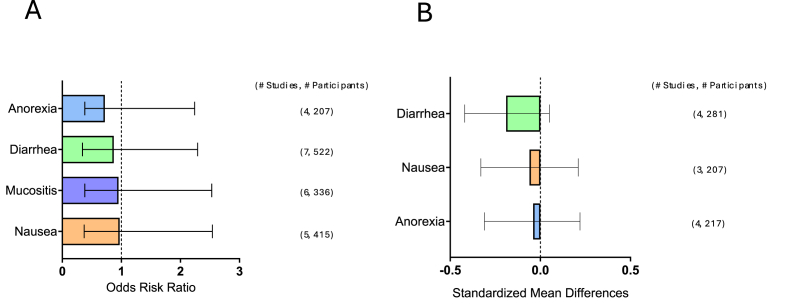
Effect of ONS on GI symptom incidence and severity. (A) ONS effects on GI symptom incidence. (B) ONS effects on GI symptom severity. Summary graph of meta-analysis results of the incidence (ORR) or severity (Hedge’s G) of GI symptoms. The dashed line indicates no effect. Bars represent the mean ORR or Hedge’s *G* with a 95% CI. Number of studies and participants are listed on the right for each symptom. CI, confidence interval; GI, gastrointestinal; ONS, oral nutrition supplementation; ORR, odds risk ratio. ∗ *P* < 0.05.

**FIGURE 3:**
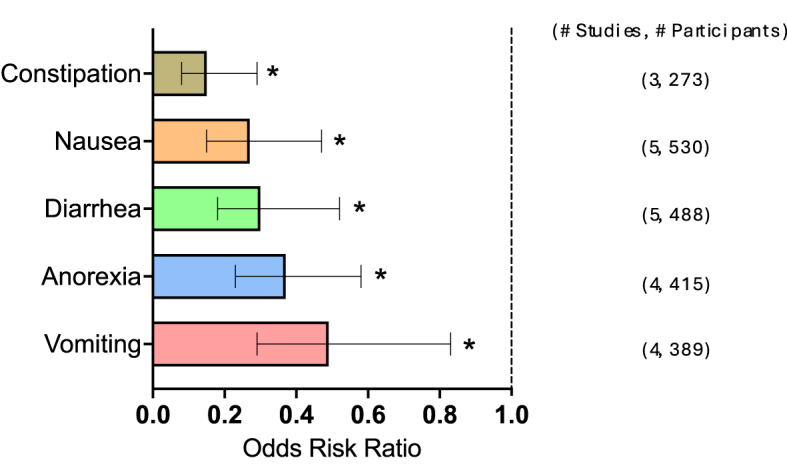
Dietary counseling on GI symptom incidence. Counseling effects on GI symptom incidence. Summary graph of meta-analysis results of the incidence (ORR). The dashed line indicates no effect. Bars represent the mean ORR with a 95% CI. Number of studies and participants are listed on the right for each symptom. CI, confidence interval; GI, gastrointestinal; ORR, odds risk ratio. ∗ *P* < 0.05.

**FIGURE 4:**
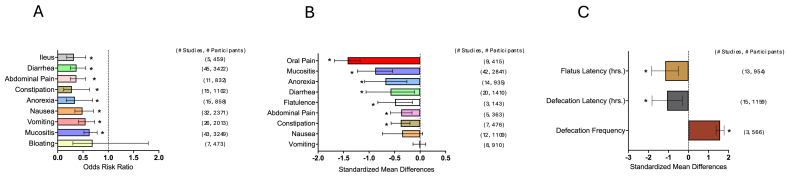
Nutrient supplementation on GI symptom incidence, GI symptom severity, and bowel timing (h). (A) Nutrient supplementation effects on symptom incidence. (B) Nutrient supplementation effects on symptom severity. (C) Nutrient supplementation effects on bowel timing (h). Summary graph of meta-analysis results of incidence (ORR), severity (Hedge’s G) and timing (h) (Hedge’s G). The dashed line indicates no effect. Bars represent the mean ORR and Hedge’s *G* with a 95% CI. Number of studies and participants are listed on the right for each symptom. CI, confidence interval; ORR, odds risk ratio. ∗ *P* < 0.05.

**FIGURE 5:**
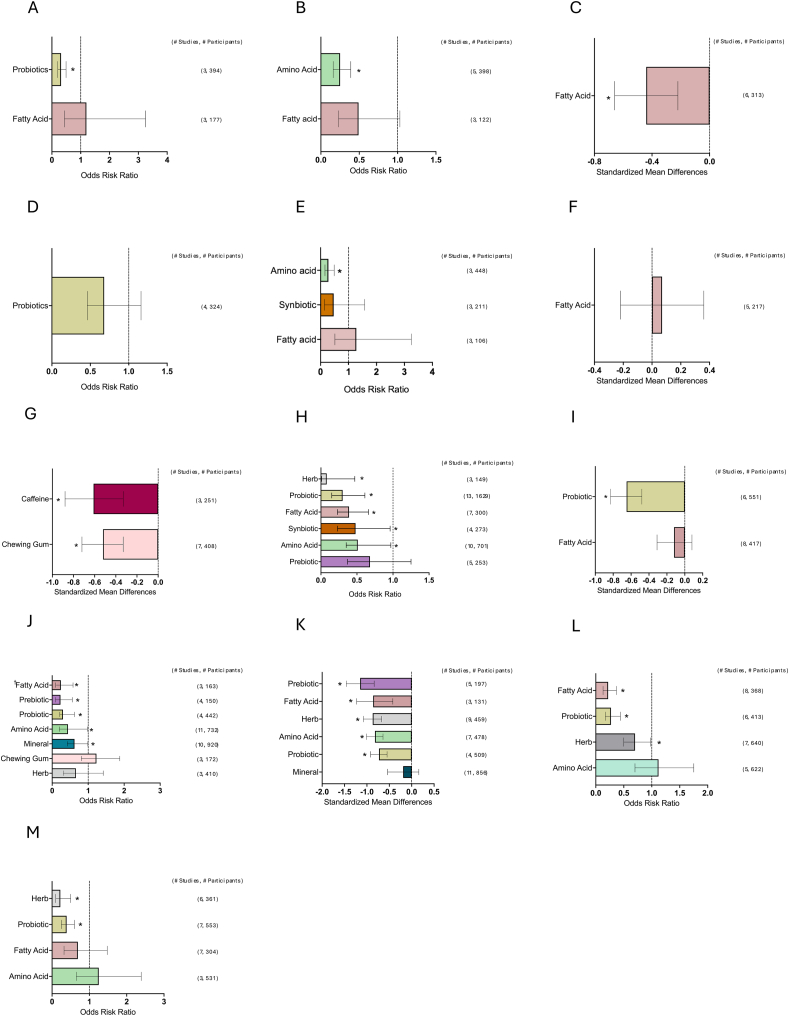
Effects of individual nutrient supplementation on GI symptom incidence and severity. (A) Abdominal pain incidence. (B) Anorexia incidence. (C) Anorexia severity. (D) Bloating incidence. (E) Constipation incidence. (F) Constipation severity. (G) Defecation latency (h). (H) Diarrhea incidence. (I) Diarrhea severity. (J) Mucositis incidence. (K) Mucositis severity. (L) Nausea incidence. (M) Vomiting incidence. Summary graph of meta-analysis results of the incidence (ORR) or severity (Hedge’s G) of GI symptoms. The dashed line indicates no effect. Bars represent the mean ORR or Hedge’s *G* with a 95% CI. Number of studies and participants are listed on the right for each nutrient supplementation. CI, confidence interval; ORR, odds risk ratio. † Indicates all fatty acids were omega-3. ∗ *P* < 0.05.

**FIGURE 6:**
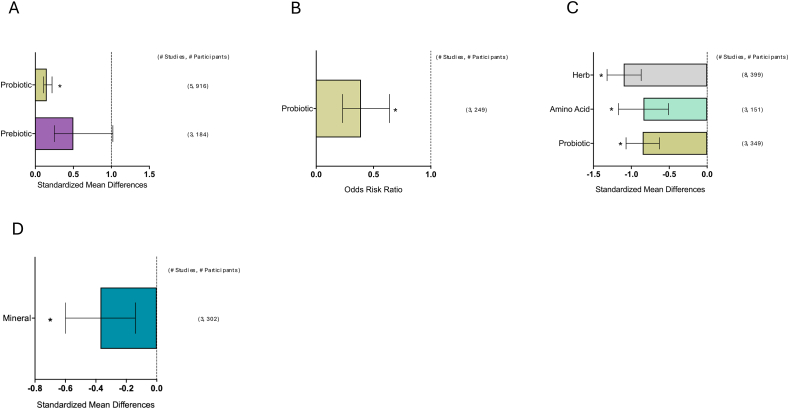
Effects of individual nutrient supplementation on GI symptom incidence and severity during variable cancer diagnoses. (A) Diarrhea incidence during colorectal cancer, (B) diarrhea incidence during GI cancers, and (C) mucositis severity during head and neck cancers. (D) Mucositis severity during leukemia. Summary graph of meta-analysis results of incidence (ORR) or severity (Hedge’s G) of GI symptoms during different cancer types. The dashed line indicates no effect. Bars represent the mean ORR or Hedge’s *G* with a 95% CI. Number of studies and participants are listed on the right for each nutrient supplementation. CI, confidence interval; GI, gastrointestinal; ORR, odds risk ratio; † Indicates all amino acids were glutamine. ∗ *P* < 0.05.

**FIGURE 7:**
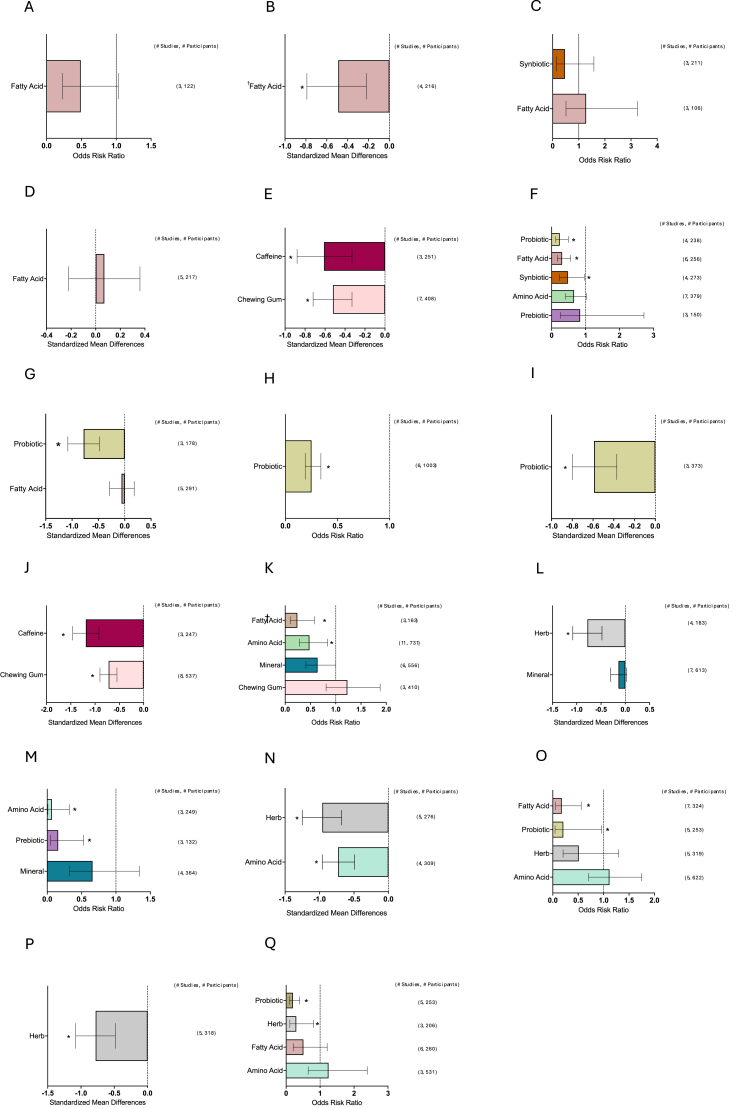
Effects of individual nutrient supplementation on GI symptom incidence and severity during variable cancer treatments. (A) Anorexia incidence during chemotherapy. (B) Anorexia severity during chemotherapy. (C) Constipation incidence during chemotherapy. (D) Constipation severity during chemotherapy. (E) Defecation latency (h) during surgical therapy. (F) Diarrhea incidence during chemotherapy. (G) Diarrhea severity during chemotherapy. (H) Diarrhea incidence during radiation therapy. (I) Diarrhea severity during radiation therapy. (J) Flatus latency (h) during surgical therapy. (K) Mucositis incidence during chemotherapy. (L) Mucositis severity during chemotherapy. (M) Mucositis incidence during radiation therapy. (N) Mucositis severity during radiation therapy. (O) Nausea incidence during chemotherapy. (P) Nausea severity during chemotherapy. (Q) Vomiting incidence during chemotherapy. Summary graph of meta-analysis results of the incidence (ORR) or severity (Hedge’s G) of GI symptoms during different treatments. The dashed line indicates no effect. Bars represent the mean ORR or Hedge’s *G* with a 95% CI. Number of studies and participants are listed on the right for each nutrient supplementation. CI, confidence interval; ORR, odds risk ratio. † Indicates all fatty acids were omega-3. ∗ *P* < 0.05.

**FIGURE 8:**
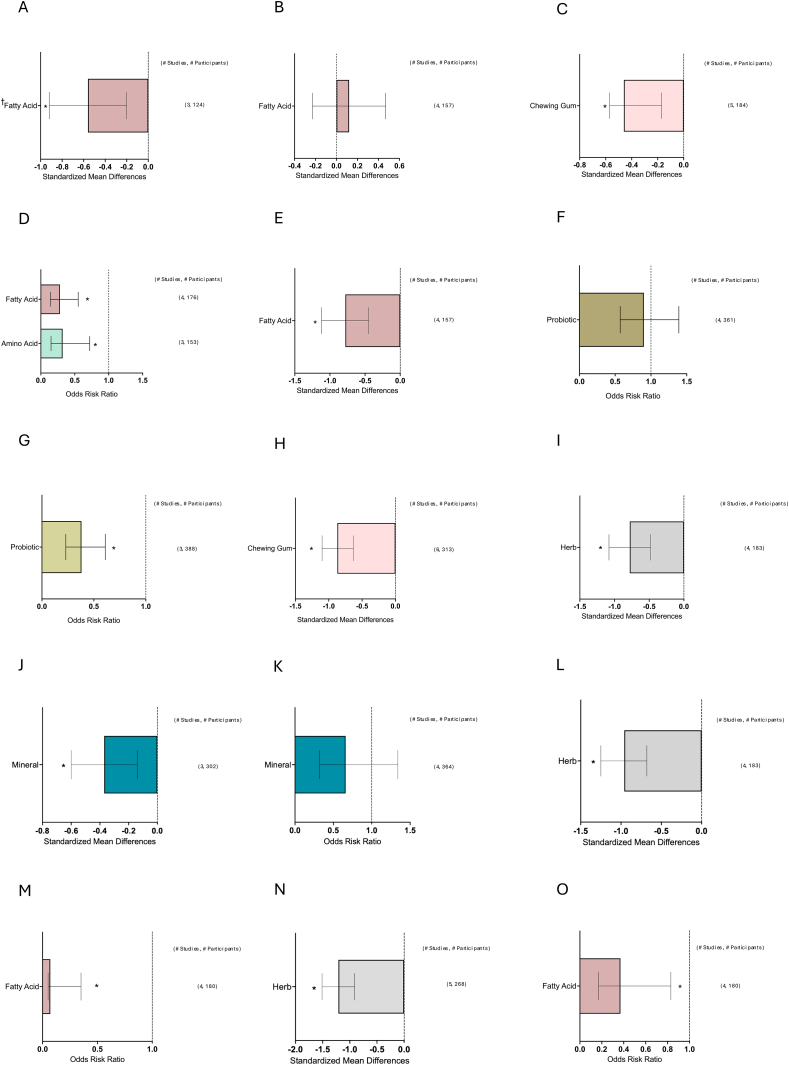
Effects of individual NS on GI symptom incidence and severity during variable cancer diagnoses and treatments. (A) Anorexia severity during GI cancers treated with chemotherapy. (B) Constipation severity during GI cancers treated with chemotherapy. (C) Defecation latency (h) during colorectal cancers treated with surgical therapy. (D) Diarrhea incidence during GI cancers treated with chemotherapy. (E) Diarrhea severity during GI cancers treated with chemotherapy. (F) Diarrhea incidence during gynecological cancers treated with radiation therapy, (G) diarrhea incidence during GI cancers treated with surgical therapy. (H) Flatus latency (h) during colorectal cancer treated with surgical therapy. (I) Mucositis severity during head and neck cancers treated with chemotherapy. (J) Mucositis severity during leukemia treated with chemotherapy. (K) Mucositis incidence during head and neck cancers treated with radiation therapy. (L) Mucositis severity during head and neck cancers treated with radiation therapy. (M) Nausea incidence during GI cancers treated with chemotherapy. (N) Oral pain during head and neck cancers treated with radiation therapy. (O) Vomiting incidence during head and neck cancers treated with chemotherapy. Summary graph of meta-analysis results of the incidence (ORR) or severity (Hedge’s G) of GI symptoms during different cancers and cancer treatments. The dashed line indicates no effect. Bars represent the mean ORR or Hedge’s *G* with a 95% CI. Number of studies and participants are listed on the right for each nutrient supplementation. CI, confidence interval; GI, gastrointestinal; ORR, odds risk ratio. † Indicates all fatty acids were omega-3. ∗ *P* < 0.05.

Figure 1 depicts the study selection flow chart. From a total of 15,556 unduplicated articles identified through the keyword and reference search, 15,202 were excluded through title and abstract screening. The remaining 354 articles were assessed in full text. Also, 211 articles were excluded after full-text review. Any studies reporting only symptoms that appeared in less than the minimum of 3 studies needed to conduct a meta-analysis were excluded (symptoms = proctitis, xerostomia, enteritis, and dysgeusia). Finally, 139 articles were included in the review: 126 nutrient supplementation, 9 ONS, and 6 dietary counseling. A total of 10,832 patients were included in total from all studies. The breakdown included: *n* = 9572 for nutrient supplementation, *n* = 722 for ONS studies, and *n* = 538 for dietary counseling. The number of studies and corresponding patient populations for each major meta-analysis are shown on the right-hand side of the forest plots in FIGURE 2, FIGURE 3, FIGURE 4, FIGURE 5, FIGURE 6, FIGURE 7, FIGURE 8.

### Basic characteristics of the selected studies

Table 2 reports the basic characteristics of the included studies. Both studies by Ravasco et al. [24,25] assessed oral nutrition supplementation and dietary counseling in comparison with their respective control groups. Each category was subdivided by GI symptom comparing latency (h), frequency (h), incidence, or severity: flatus latency, defecation latency, defecation frequency, abdominal pain, anorexia, bloating, constipation, diarrhea, ileus, mucositis, nausea, oral pain, and vomiting. Within those categories, sub-meta-analyses were conducted where ≥3 studies were available, divided as follows: cancer treatment type: radiation therapy, chemotherapy, and surgical therapy; cancer type: colorectal cancer (colon, rectum, or both), gynecological cancers (ovarian, pelvic, vulvar, vaginal, and cervical), hematological cancers (leukemia and lymphoma), GI cancers (esophageal, stomach, gastric, liver, pancreatic, colon, rectum, intestine) [169], head and neck (oral cancer, head and neck) and breast cancer; and by specific nutrient intervention: nutrient additions [probiotic, synbiotic, herbs (including: ginger), caffeine, gum, amino acid (including: glutamine), fatty acid (including: omega-3), polysaccharide (including: honey), and minerals (including: zinc sulfate)], ONS (including: Elental supplement), no further divisions were made for dietary counseling. Full descriptive statistics of each individual meta-analysis is reported in the Supplemental Figure 1.

### Effects of ONS on GI symptom severity and incidence

The results of the meta-analysis of ONS on GI symptom incidence and severity are displayed in Figure 2A and B [24,25,[26], [27], [28], [29], [30], [31], [32][162]]. ONS did not reduce the incidence of the following symptoms: anorexia: [95% confidence interval (CI): 0.34, 1.52; *P* = 0.39], diarrhea: (95% CI: 0.53, 1.42; *P* = 0.58), mucositis: (95% CI: 0.57, 1.58; *P* = 0.85), and nausea: (95% CI: 0.60, 1.57; *P* = 0.91). Furthermore, a subdivision of ONS, Elental [28,30,32], did not reduce the incidence of the following symptoms: mucositis (95% CI: –0.53, 0.68; *P* = 0.81).

ONS did not reduce the severity of the following symptoms: diarrhea: (95% CI: –0.42, 0.05; *P* = 0.12), nausea: (95% CI: –0.33, 0.21; *P* = 0.68), and anorexia: (95% CI: –0.31, 0.22; *P* = 0.74).

### Effects of nutrition counseling on GI symptom incidence

The results of meta-analysis for counseling on GI symptom incidence are displayed in Figure 3 [24,25,[33], [34], [35], [36]]. Counseling reduced the incidence of the following symptoms: constipation: ORR = 0.15 (95% CI: 0.08, 0.29; *P* < 0.001), nausea: ORR = 0.27 (95% CI: 0.15, 0.47; *P* < 0.001), diarrhea: ORR = 0.30 (95% CI: 0.18, 0.52; *P* < 0.001), anorexia: ORR = 0.37 (95% CI: 0.23, 0.58; *P* < 0.001), and vomiting: ORR = 0.49 (95% CI: –1.94, 0.44; *P* = 0.01).

Not enough studies captured the efficacy of counseling on GI symptom severity.

### Effects of nutrient supplementation on symptom incidence, severity, and latency (h)

The results of the meta-analysis for nutrient supplementation on GI symptom incidence, severity, and latency (h) are displayed in Figure 4A–C respectively [[37], [38], [39], [40], [41], [42], [43], [44], [45], [46], [47], [48], [49], [50], [51], [52], [53], [54], [55], [56], [57], [58], [59], [60], [61], [62], [63], [64], [65], [66], [67], [68], [69], [70], [71], [72], [73], [74], [75], [76], [77], [78], [79], [80], [81], [82], [83], [84], [85], [86], [87], [88], [89], [90], [91], [92], [93], [94], [95], [96], [97], [98], [99], [100], [101], [102], [103], [104], [105], [106], [107], [108], [109], [110], [111], [112], [113], [114], [115], [116], [117], [118], [119], [120], [121], [122], [123], [124], [125], [126], [127], [128], [129], [130], [131], [132], [133], [134], [135], [136], [137], [138], [139], [140], [141], [142], [143], [144], [145], [146], [147], [148], [149], [150], [151], [152], [153], [154], [155], [156], [157], [158], [159], [160][161]]. Nutrient supplementation reduced the incidence of the following symptoms: ileus: ORR = 0.32 (95% CI: 0.18, 0.55; *P* = 0.03), diarrhea: ORR = 0.37 (95% CI: 0.25, 0.55; *P* < 0.001), abdominal pain: ORR = 0.41 (95% CI: 0.29, 0.58; *P* < 0.001), constipation: ORR = 0.28 (95% CI: 0.12, 0.63; *P* < 0.001), anorexia: ORR = 0.34 (95% CI: 0.17, 0.69; *P* < 0.001), nausea: ORR = 0.49 (95% CI: 0.34, 0.71; *P* < 0.001), vomiting: ORR = 0.55 (95% CI: 0.41, 0.73; *P* < 0.001), and mucositis: ORR = 0.63 (95% CI: 0.52, 0.78; *P* < 0.001). However, nutrient supplementation did not reduce the incidence of the following symptoms: bloating: (ORR 95% CI: 0.30, 1.79; *P* = 0.10).

Nutrient supplementation reduced the severity of the following symptoms: oral pain: Hedge’s *G* = –1.42 (95% CI: –1.68, –1.18; *P* < 0.001), mucositis: Hedge’s *G* = –0.88 (95% CI: –1.23, –0.54; *P* < 0.001), anorexia: Hedge’s *G* = –0.68 (95% CI: –1.09, –0.26; *P* < 0.001), diarrhea: Hedge’s *G* = –0.58 (95% CI: –1.06, –0.11; *P* = 0.02), flatulence: Hedge’s *G* = –0.49 (95% CI: –0.83, –0.15; *P* < 0.001), abdominal pain: Hedge’s *G* = –0.68 (95% CI: –0.58, –0.16; *P* < 0.001), constipation: Hedge’s *G* = –0.38 (95% CI: –0.57, –0.20; *P* < 0.001). However, nutrient supplementation did not reduce the severity of the following symptoms: nausea: (95% CI: –0.74, 0.05; *P* = 0.09) and vomiting: (95% CI: –0.14, 0.11; *P* = 0.83).

Nutrient supplementation reduced the latency (h) of the following symptoms: flatus latency (h): Hedge’s *G* = –1.16 (95% CI: –1.84, –0.49; *P* < 0.001) and defecation latency (h): Hedge’s *G* = –1.07 (95% CI: –1.26, –0.31; *P* = 0.01). However, nutrient supplementation increased defecation frequency: Hedge’s *G* = 1.58 (95% CI: 1.38, 1.78; *P* < 0.001).

### Effects of specific nutrient supplementations on symptom incidence and severity

#### Abdominal pain

The results of the meta-analysis for specific nutrient supplementation on abdominal pain incidence are displayed in Figure 5A [65,[111], [112], [113],^135^,^153^]. The following nutrients reduced abdominal pain incidence: probiotic: abdominal pain: ORR = 0.32 (95% CI: 0.21, 0.49; *P* < 0.001). However, the following nutrients reduced abdominal pain incidence: fatty acids: (95% CI: 0.44, 3.25; *P* = 0.73).

Not enough studies captured abdominal pain severity.

#### Anorexia

The results of the meta-analysis for specific nutrient supplementation on anorexia incidence and severity are displayed in Figure 5B [41,49,88,115,122,136,148,150] and C [49,70,120,140,145,146], respectively. The following nutrients reduced anorexia incidence: amino acids: ORR = 0.25 (95% CI: 0.16, 0.39; *P* < 0.001). However, the following nutrients did not reduce anorexia incidence: fatty acids: (95% CI: 0.23, 1.03; *P* = 0.06).

The following nutrients reduced anorexia severity: fatty acids: Hedge’s *G* = –0.44 (95% CI: –0.66, –0.22; *P* < 0.001), specifically, omega-3 [51,122,142,147,1148]: Hedge’s *G* = –0.42 (95% CI: –0.65, –0.18; *P* < 0.001).

#### Bloating

The results of the meta-analysis for specific nutrient supplementation on bloating are displayed in Figure 5D [111,112,118,119]. The following nutrients did not reduce bloating incidence: probiotics: (95% CI: 0.46, 1.16; *P* = 0.19).

Not enough studies captured bloating severity.

#### Constipation

The results of the meta-analysis for specific nutrient supplementation on constipation incidence and severity are displayed in Figure 5E [41,49,61,66,73,98,115,122,136] and F [49,70,105,120,145], respectively. The following nutrients reduced constipation incidence: amino acids: ORR = 0.28 (95% CI: 0.16, 0.49; *P* < 0.001). However, the following nutrients did not reduce constipation incidence: synbiotics: (95% CI: 0.14, 1.58; *P* = 0.22) and fatty acids: (95% CI: 0.51, 3.25; *P* = 0.60).

The following nutrients did not reduce constipation severity: fatty acids: (95% CI: –0.22, 0.36; *P* = 0.63).

#### *Defecation latency (h)*

The results of the meta-analysis for specific nutrient supplementation on defecation latency (h) are displayed in Figure 5G [44,63,67,76,79,82,83,99,111,112,118,130,159]. The following nutrients reduced defecation latency (h): chewing gum: Hedge’s *G* = –0.52 (95% CI: –0.72, –0.33; *P* < 0.001) and caffeine: Hedge’s *G* = –0.61 (95% CI: –0.88, –0.33; *P* < 0.001).

#### Diarrhea

The results of the meta-analysis for specific nutrient supplementation on diarrhea incidence and severity are displayed in Figure 5H [44,48,49,54,[59], [60], [61], [62], [63], [64], [65], [66],^73^,^78^,^86^,^87^,^98^,^102^,^106^,^108^,[110], [111], [112], [113],^115^,^119^,^123^,^133^,^134^,^136^,^137^,^139^,^148^,[150], [151], [152],^155^,^157^,^160^,^161^] and I [49,64,65,70,105,113,[118], [119], [120],^140^,^145^,^146^,^152^,^160^], respectively. The following nutrients reduced diarrhea incidence: herbs: ORR = 0.08 (95% CI: 0.01, 0.45; *P* = 0.04), probiotics: ORR = 0.30 (95% CI: 0.15, 0.61; *P* <0.001), fatty acids: ORR = 0.39 (95% CI: 0.23, 0.66; *P* < 0.001), synbiotics: ORR = 0.48 (95% CI: 0.23, 0.96; *P* = 0.04), and amino acids: ORR = 0.51 (95% CI: 0.35, 0.97; *P* < 0.001). However, the following nutrients did not reduce diarrhea incidence prebiotics: (95% CI: 0.37, 1.25; *P* = 0.19).

The following nutrients reduced diarrhea severity: probiotics: Hedge’s *G* = –0.65 (95% CI: –0.83, –0.48; *P* < 0.001). However, the following nutrients did not reduce diarrhea severity: fatty acids (95% CI: –0.31, 0.08; *P* = 0.24), specifically omega-3 fatty acids [49,113,120,140,145,146]: (95% CI: –0.28, 0.16; *P* = 0.58).

#### Mucositis

The results of the meta-analysis for nutrient supplementation on mucositis incidence and severity are depicted in Figure 5J [39,40,49,52,56,57,60,65,67,[71], [72], [73], [74],^77^,^80^,^84^,^85^,^87^,[91], [92], [93],^101^,^103^,^108^,^116^,^117^,^121^,^123^,^125^,^126^,^132^,^133^,^136^,^138^,[141], [142], [143],^149^,^150^,^154^,^156^] and K [37,45,46,49,51,52,56,65,67,68,71,77,80,84,85,[91], [92], [93], [94],^103^,^109^,^116^,^117^,^121^,^122^,^125^,^126^,[131], [132], [133],^136^,^138^,[142], [143], [144],^147^,^149^,^154^,^156^], respectively. The following nutrients reduced mucositis incidence: fatty acids, specifically omega-3: (95% CI: 0.10, 0.58; *P* < 0.001), prebiotics ORR = 0.23 (95% CI: 0.09, 0.56; *P* < 0.001), probiotics: ORR = 0.30 (95% CI: 0.21, 0.62; *P* < 0.001), amino acids: ORR = 0.44 (95% CI: 0.20, 0.98; *P* = 0.04), specifically glutamine [39,52,56,57,84,85,108,154]: ORR = 0.54 (95% CI: 0.32, 0.94; *P* = 0.03), minerals: ORR = 0.62 (95% CI: 0.42, 0.99; *P* = 0.01). However, the following nutrients did not reduce mucositis incidence: a sub division of minerals—zinc sulfate [40,117,138,141]: (95% CI: 0.43, 1.55; *P* = 0.54), herbs: (95% CI: 0.43, 1.55; *P* = 0.54), and chewing gum: (95% CI: 0.81, 1.88; *P* = 0.33).

The following nutrients reduced mucositis severity: prebiotics: Hedge’s *G* = –1.51 (95% CI: –1.46, –0.84; *P* < 0.001), specifically honey [46,92,132]: Hedge’s *G* = –1.63 (95% CI: –2.09, –1.17; *P* < 0.001), fatty acids: Hedge’s *G* = –0.86 (95% CI: –1.23, –0.48; *P* < 0.001), herbs: Hedge’s *G* = –0.87 (95% CI: –1.08, –0.67; *P* < 0.001), amino acids: Hedge’s *G* = –0.82 (95% CI: –1.01, –0.64; *P* < 0.001), specifically glutamine [52,56,84,85,154]: Hedge’s *G* = –0.97 (95% CI: –1.23, –0.71; *P* < 0.001), probiotics: Hedge’s *G* = –0.73 (95% CI: –0.92, –0.55; *P* < 0.001), and a subdivision of minerals - zinc [77,109,142]: Hedge’s *G* = –0.37 (95% CI: –0.58, –0.15; *P* < 0.001) and zinc sulfate [117,131,138]: Hedge’s *G* = –0.44 (95% CI: –0.77, –0.12 ; *P* = 0.01). However, the following nutrients did not reduce mucositis severity: minerals: (95% CI: –0.54, 0.16; *P* = 0.29), and a subdivision of minerals—selenium [91,103,144]: (95% CI: –0.08, 0.48; *P* = 0.15).

#### Nausea

The results of the meta-analysis for nutrient supplementation on nausea incidence are depicted in Figure 5L [41,44,47,49,58,60,61,66,73,81,86,97,98,107,110,113,115,122,129,134,148,149,151,153,160]. The following nutrients reduced nausea incidence: fatty acids: ORR = 0.22 (95% CI: 0.13, 0.37; *P* < 0.001), specifically omega-3 [41,44,49,60,86,113]: ORR = 0.21 (95% CI: 0.12, 0.37; *P* < 0.001) probiotics: ORR = 0.27 (95% CI: 0.17, 0.44; *P* < 0.001), and herbs: ORR = 0.70 (95% CI: 0.50, 0.98; *P* = 0.04). However, amino acids: (95% CI: 0.70, 1.75; *P* = 0.63) and a subdivision of herbs— ginger [47,58,107,127,129]: (95% CI: 0.50, 1.01; *P* = 0.06) did not reduce nausea incidence.

Not enough studies captured nausea severity.

#### Vomiting

The results of the meta-analysis for nutrient supplementation on vomiting incidence are depicted in Figure 5M [41,44,49,58,60,61,66,73,81,86,106,107,110,113,125,122,129,134,148,151,155,160]. The following nutrients reduced vomiting incidence: herbs: ORR = 0.22 (95% CI: 0.10, 0.50; *P* < 0.001) and probiotics: ORR = 0.39 (95% CI: 0.25, 0.60; *P* < 0.001). However, fatty acids: (95% CI: 0.32, 1.48; *P* = 0.34), specifically omega-3 [41,44,49,60,86,113]: (95% CI: 0.22, 1.17; *P* = 0.11) and amino acids: (95% CI: 0.65, 2.39; *P* = 0.50) did not reduce vomiting incidence.

Not enough studies captured vomiting severity.

### Symptom incidence and severity by cancer type

#### Effects of nutrient supplementation on diarrhea during GI cancers

The results of the meta-analysis for nutrient supplementation on diarrhea incidence during GI cancers are depicted in Figure 6A [63,87,111,112,119,155,157,161]. The following nutrients reduced diarrhea incidence during GI cancers: probiotics: ORR = 0.15 (95% CI: 0.11, 0.22; *P* < 0.001). However, the following nutrients did not reduce diarrhea incidence during GI cancers: prebiotics: (95% CI: 0.25, 1.02; *P* = 0.06).

Not enough studies captured diarrhea severity during GI cancers.

#### Effects of nutrient supplementation on diarrhea during colorectal cancer

The results of the meta-analysis for nutrient supplementation on diarrhea incidence during colorectal cancers are depicted in Figure 6B [111,112,119]. The following nutrients reduced diarrhea incidence during colorectal cancer: probiotics: ORR = 0.39 (95% CI: 0.23, 0.64; *P* < 0.001).

Not enough studies captured diarrhea severity during colorectal cancers.

#### Effects of nutrient supplementation on mucositis during head and neck cancers

The results of the meta-analysis for nutrient supplementation on mucositis severity during head and neck cancers are depicted in Figure 6C [37,45,52,68,71,84,85,93,96,116,143,147,149,156]. The following nutrients reduced mucositis severity during head and neck cancers: herbs: Hedge’s *G* = –1.10 (95% CI: –1.32, –0.87; *P* < 0.001), amino acids, specifically glutamine: Hedge’s *G* = –0.84 (95% CI: –1.17, –0.51; *P* < 0.001), and probiotics: Hedge’s *G* = –0.85 (95% CI: –1.07, –0.63; *P* < 0.001).

Not enough studies captured mucositis incidence and head and neck cancers.

#### Effects of nutrient supplementation on mucositis during leukemia

The results of the meta-analysis for nutrient supplementation on mucositis severity during leukemia are depicted in Figure 6D [77,131,142]. The following nutrients reduced mucositis severity during leukemia: minerals: Hedge’s *G* = –0.37 (95% CI: –0.60, –0.14; *P* < 0.001).

Not enough studies captured mucositis incidence and leukemia.

### Symptom incidence and severity by treatment type

#### Effects of nutrient supplementation on anorexia during chemotherapy

The results of the meta-analysis for nutrient supplementation on anorexia incidence and severity during chemotherapy therapy are depicted in Figure 7A [41,49,115] and B [49,120,140,145], respectively. The following nutrients did not reduce anorexia incidence during chemotherapy: fatty acids: (95% CI: 0.23, 1.03; *P* = 0.06).

The following nutrients reduced anorexia severity during chemotherapy: fatty acids specifically, omega–3: Hedge’s *G* = –0.49 (95% CI: –0.79, –0.22; *P* < 0.001).

#### Effects of nutrient supplementation on constipation during chemotherapy

The results of the meta-analysis for nutrient supplementation on constipation incidence and severity during chemotherapy are depicted in Figure 7C [41,49,61,66,73,115] and D [49,70,105,120,145], respectively. The following nutrients did not reduce constipation incidence during chemotherapy: synbiotics: (95% CI: 0.14, 1.58; *P* = 0.22) and fatty acids: (95% CI: 0.51, 3.25; *P* = 0.68).

Similarly, the following nutrients did not reduce constipation severity during chemotherapy: fatty acids: (95% CI: –0.22, 0.36; *P* = 0.63).

#### Effects of nutrient supplementation on defecation latency (h) during surgical therapy

The results of the meta-analysis for nutrient supplementation on defecation latency (h) during surgical therapy are depicted in Figure 7E [43,69,76,79,82,83,99,130,159]. The following nutrients reduced defecation latency (h) during surgical therapy: caffeine: Hedge’s *G* = –0.61 (95% CI: –0.88, –0.33; *P* < 0.001) and chewing gum: Hedge’s *G* = –0.52 (95% CI: –0.72, –0.33; *P* < 0.001).

#### Effects of nutrient supplementation on diarrhea during chemotherapy

The results of the meta-analysis for nutrient supplementation on diarrhea incidence and severity during chemotherapy are depicted in Figure 7F [33,40,44,48,49,[59], [60], [61],^66^,^73^,^86^,^87^,^98^,^108^,^116^,^119^,^123^,^133^,^134^,^148^,[150], [151], [152],^160^,^161^] and G [49,105,119,120,140,145,152,160], respectively. The following nutrients reduced diarrhea incidence during chemotherapy: probiotics: ORR = 0.24 (95% CI: 0.12, 0.49; *P* < 0.001), fatty acids: ORR = 0.31 (95% CI: 0.17, 0.56; *P* = 0.06), specifically omega–3 [41,44,49,60,86]: ORR = 0.38 (95% CI: 0.20, 0.72; *P* < 0.001), and synbiotics: ORR = 0.48 (95% CI: 0.23, 0.96; *P* = 0.04). However, the following nutrients did not reduce diarrhea incidence during chemotherapy therapy: amino acids: (95% CI: 0.41, 1.03; *P* = 0.07), specifically glutamine [48,59,108,148]: (95% CI: 0.36, 1.09; *P* = 0.10), and prebiotics: (95% CI: 0.26, 2.72; *P* = 0.78).

The following nutrients reduced diarrhea severity during chemotherapy: probiotics: Hedge’s *G* = –0.78 (95% CI: –1.08, –0.48; *P* < 0.001). However, the following nutrients did not reduce diarrhea severity during chemotherapy: fatty acids: (95% CI: –0.29, 0.18; *P* = 0.63).

#### Effects of nutrient supplementation on diarrhea during radiation therapy

The results of the meta-analysis for nutrient supplementation on diarrhea incidence and severity during radiation therapy are depicted in Figure 7H [54,[63], [64], [65],^78^,^139^] and I [64,65,118]. The following nutrients reduced diarrhea incidence during radiation therapy: probiotics: ORR = 0.25 (95% CI: 0.19, 0.34; *P* < 0.001).

The following nutrients reduced diarrhea severity during radiation therapy: probiotics: Hedge’s *G* = –0.59 (95% CI: –0.08, –0.37; *P* < 0.001).

#### Effects of nutrient supplementation on flatus latency (h) during surgical therapy

The results of the meta-analysis for nutrient supplementation on flatus latency (h) during surgical therapy are depicted in Figure 7J [38,43,69,76,79,82,83,99,110,130,159]. The following nutrients reduced flatus latency (h) during surgical therapy: caffeine: Hedge’s *G* = –1.19 (95% CI: –1.46, –0.92; *P* < 0.001) and chewing gum: Hedge’s *G* = –0.72 (95% CI: –0.90, –0.50; *P* < 0.001).

#### Effects of nutrient supplementation on mucositis during chemotherapy

The results of the meta-analysis for nutrient supplementation on mucositis incidence and severity during chemotherapy are depicted in Figure 7K [39,49,60,67,74,77,80,91,108,117,121, 126,133,142,150,154] and L [37,45,68,77,96,103,121,126,131,142,144], respectively. The following nutrients reduced mucositis incidence during chemotherapy: fatty acids, specifically omega–3: ORR = 0.66 (95% CI: 0.10, 0.58; *P* < 0.001), and amino acids: ORR = 0.48 (95% CI: 0.28, 0.84; *P* = 0.01). However, the following nutrients did not reduce mucositis incidence during chemotherapy: minerals: (95% CI: 0.41, 1.00; *P* = 0.05) and chewing gum: (95% CI: 0.81, 1.88; *P* = 0.33).

The following nutrients reduced mucositis severity during chemotherapy: herbs: Hedge’s *G* = –0.78 (95% CI: –1.08, –0.48; *P* < 0.001). However, the following nutrients did not reduce mucositis severity during chemotherapy: minerals: (95% CI: –0.30, 0.03; *P* = 0.10).

#### Effects of nutrient supplementation on mucositis during radiation

The results of the meta-analysis for nutrient supplementation on mucositis incidence and severity during radiation therapy are depicted in Figure 7M [40,51,84,85,92,103,132,136,138,141] and N [71,84,85,125,133,136,147,149,156], respectively. The following nutrients reduced mucositis incidence during radiation therapy: amino acids: ORR = 0.07 (95% CI: 0.01, 0.32; *P* < 0.001) and prebiotics: ORR= 0.16 (95% CI: 0.05, 0.53; *P* < 0.001). However, the following nutrients reduced mucositis incidence during radiation therapy: minerals: (95% CI: 0.32, 1.34; *P* = 0.25), specifically zinc sulfate [40,138,141]: (95% CI: 0.32, 1.40; *P* = 0.30).

The following nutrients reduced mucositis severity during radiation therapy: herbs: Hedge’s *G* = –0.96 (95% CI: –1.25, –0.68; *P* = 0.12) and amino acids: Hedge’s *G* = –0.73 (95% CI: –0.96, –0.49; *P* < 0.001).

#### Effects of nutrient supplementation on nausea during chemotherapy

The results of the meta-analysis for nutrient supplementation on nausea incidence and severity during chemotherapy are depicted in Figure 7O [40,44,49,58,60,61,73,81,86,97,98,107,115,122,127,129,134,148,150,151,153,160] and P [42,58,89,90,129], respectively. The following nutrients reduced nausea incidence during chemotherapy: fatty acids: ORR = 0.85 (95% CI: 0.10, 0.31; *P* < 0.001), specifically omega–3 [41,44,49,60,86]: ORR = 0.15 (95% CI: 0.08, 0.30; *P* < 0.001), probiotics: ORR = 0.19 (95% CI: 0.10, 0.38; *P* < 0.001), and herbs: ORR = 0.52 (95% CI: 0.32, 0.85; *P* = 0.01), specifically ginger [58,107,127,129]: ORR = 0.52 (95% CI: 0.32, 0.89; *P* = 0.01). However, the following nutrients did not reduce nausea incidence during chemotherapy: amino acids: (95% CI: 0.70, 1.75; *P* = 0.64).

The following nutrients reduced nausea severity during chemotherapy: herbs: Hedge’s *G* = –0.27 (95% CI: –0.50, –0.04; *P* = 0.02), specifically ginger [42,58,129]: Hedge’s *G* = –0.38 (95% CI: –0.65, –0.10; *P* = 0.01).

#### Effects of nutrient supplementation on vomiting during chemotherapy

The results of the meta-analysis for nutrient supplementation on vomiting incidence during chemotherapy are depicted in Figure 7Q [41,44,49,58,60,61,73,81,86,107,105,122,129,134,148,151,160]. The following nutrients reduced vomiting incidence during chemotherapy: probiotics: ORR = 0.20 (95% CI: 0.10, 0.40; *P* < 0.001), herbs, specifically ginger: ORR = 0.30 (95% CI: 0.11, 0.81; *P* = 0.02), and a subanalysis of fatty acids, omega-3 [41,44,52,61,86]: ORR = 0.30 (95% CI: 0.11, 0.82; *P* = 0.02). However, the following nutrients did not reduce vomiting incidence during chemotherapy: fatty acids: (95% CI: 0.21, 1.21; *P* = 0.13) and amino acids: (95% CI: 0.65, 2.39; *P* = 0.50).

Not enough studies captured vomiting severity and chemotherapy.

### Symptom incidence and severity depending on nutrient, cancer type, and cancer treatment

#### Effects of nutrient supplementation on anorexia during GI cancers treated with chemotherapy

The results of the meta-analysis for nutrient supplementation on anorexia severity during GI cancers treated with chemotherapy are depicted in Figure 8A [49,120,145]. The following nutrients reduced anorexia severity during GI cancers treated with chemotherapy: fatty acids, specifically omega-3: Hedge’s *G* = –0.56 (95% CI: –0.92, –0.20; *P* < 0.001).

Not enough studies captured anorexia incidence during GI cancers treated with chemotherapy.

#### Effects of nutrient supplementation on constipation during GI cancers treated with chemotherapy

The results of the meta-analysis for nutrient supplementation on constipation severity during GI cancers chemotherapy is depicted in Figure 8B [49,70,120,145]. The following nutrients reduced constipation severity during GI cancers chemotherapy: fatty acids: (95% CI: –0.23, 0.47; *P* = 0.51), specifically omega-3 [49,120,145]: (95% CI: –0.68, 0.07; *P* = 0.11).

Not enough studies captured constipation incidence during GI cancers treated with chemotherapy.

#### Effects of nutrient supplementation on defecation latency (h) during colorectal cancer and surgical therapy

The results of the meta-analysis for nutrient supplementation on defecation latency (h) during colorectal cancer and surgical therapy are depicted in Figure 8C [43,82,99,130]. The following nutrients reduced defecation latency (h) during colorectal cancer and surgical therapy: chewing gum: Hedge’s *G* = –0.46 (95% CI: –0.57, –0.17; *P* < 0.001).

#### Effects of nutrient supplementation on diarrhea during GI cancers treated with chemotherapy

The results of the meta-analysis for nutrient supplementation on diarrhea incidence and severity during GI cancers treated with chemotherapy are depicted in Figure 8D [44,49,59,86,98,115,150] and E [49,70,120,145], respectively. The following nutrients reduced diarrhea incidence during GI cancers treated with chemotherapy: fatty acids: ORR = 0.28 (95% CI: 0.14, 0.55; *P* < 0.001), specifically omega-3 [44,49,86]: ORR = 0.37 (95% CI: 0.18, 0.76; *P* = 0.01), and amino acids: ORR = 0.32 (95% CI: 0.15, 0.72; *P* = 0.01).

Similarly, the following nutrients reduced diarrhea severity during GI cancers treated with chemotherapy: fatty acids: Hedge’s *G* = –0.78 (95% CI: –1.12, –0.45; *P* < 0.001), specifically omega-3 [49,120,145]: Hedge’s *G* = –0.51 (95% CI: –0.86, –0.15; *P* = 0.01).

#### Effects of nutrient supplementation on diarrhea during gynecological cancers treated with radiation therapy

The results of the meta-analysis for nutrient supplementation on diarrhea incidence during gynecological cancers during radiation therapy are depicted in Figure 8F [54,64,78,139]. The following nutrients did not reduce diarrhea incidence during gynecological cancers during radiation therapy: probiotics: (95% CI: 0.57, 1.39; *P* = 0.62).

Not enough studies captured diarrhea severity during gynecological cancers treated with radiation therapy.

#### Effects of nutrient supplementation on diarrhea during GI cancers with surgical therapy

The results of the meta-analysis for nutrient supplementation on diarrhea incidence during GI cancers and surgical therapy are depicted in Figure 8G [111,112,155]. The following nutrients reduced diarrhea incidence during GI cancers and surgical therapy: probiotics: ORR = 0.38 (95% CI: 0.23, 0.61; *P* < 0.001).

Not enough studies captured diarrhea severity during GI cancers treated with surgical therapy.

#### Effects of nutrient supplementation on flatus latency (h) during colorectal cancers during surgical therapy

The results of the meta-analysis for nutrient supplementation on flatus latency (h) during colorectal cancers during surgical therapy are depicted in Figure 8H [38,43,82,83,99,130]. The following nutrients reduced flatus latency (h) during colorectal cancers during surgical therapy: chewing gum: Hedge’s *G* = –0.87 (95% CI: –1.10, –0.63; *P* < 0.001).

#### Effects of nutrient supplementation on mucositis during head and neck cancers and chemotherapy

The results of the meta-analysis for nutrient supplementation on mucositis severity during head and neck cancers and chemotherapy are depicted in Figure 8I [37,45,68,96]. The following nutrients reduced mucositis severity during head and neck cancers and chemotherapy: herbs: Hedge’s *G* = –0.78 (95% CI: –1.081, –0.48; *P* < 0.001).

Not enough studies captured mucositis incidence during head and neck cancers treated with chemotherapy.

#### Effects of nutrient supplementation on mucositis during leukemia and chemotherapy

The results of the meta-analysis for nutrient supplementation on mucositis severity during leukemia and chemotherapy are depicted in Figure 8J [77,131,142]. The following nutrients reduced mucositis severity during leukemia and chemotherapy: minerals: Hedge’s *G* = –0.37 (95% CI: –0.60, –0.14; *P* < 0.001).

Not enough studies captured mucositis incidence during leukemia treated with chemotherapy.

#### Effects of nutrient supplementation on mucositis during head and neck cancers treated with radiation therapy

The results of the meta-analysis for nutrient supplementation on mucositis incidence and severity during head and neck cancers treated with radiation therapy are depicted in Figure 8K [40,103,138,141] and L [71,147,149,156], respectively. The following nutrients reduced mucositis incidence during head and neck cancers treated with radiation therapy: minerals: (95% CI: 0.29, 1.20; *P* = 0.14). However, the following nutrients did not reduce mucositis incidence during head and neck cancers treated with radiation therapy: a subdivision of minerals—zinc sulfate [45,143,146]: (95% CI: 0.32, 1.40; *P* = 0.30).

The following nutrients reduced mucositis severity during head and neck cancers treated with radiation therapy: herbs: Hedge’s *G* = –0.96 (95% CI: –1.25, –0.68; *P* < 0.001).

#### Effects of nutrient supplementation on nausea during GI cancers and chemotherapy

The results of the meta-analysis for nutrient supplementation on nausea incidence during GI cancers and chemotherapy are depicted in Figure 8M [44,49,86,115]. The following nutrients reduced nausea incidence during GI cancers and chemotherapy: fatty acids: ORR = 0.07 (95% CI: 0.05, 0.35; *P* < 0.001), specifically omega–3 [44,49,86]: ORR = 0.11 (95% CI: 0.05, 0.24; *P* < 0.001).

Not enough studies captured nausea severity during GI cancers treated with chemotherapy.

#### Effects of nutrient supplementation on oral pain during head and neck cancers and radiation therapy

The results of the meta-analysis for nutrient supplementation on oral pain severity during head and neck cancers and radiation therapy are depicted in Figure 8N [37,68,71,147,149]. The following nutrients reduced oral pain severity during head and neck cancers and radiation therapy: herbs: Hedge’s *G* = –1.21 (95% CI: –1.51, –0.91; *P* < 0.001).

Not enough studies captured oral pain incidence during head and neck cancers and radiation therapy.

#### Effects of nutrient supplementation on vomiting during GI cancers and chemotherapy

The results of the meta-analysis for nutrient supplementation on vomiting incidence during GI cancers and chemotherapy are depicted in Figure 8O [44,49,86,115]. The following nutrients reduced vomiting incidence during GI cancers and chemotherapy: fatty acids: ORR = 0.37 (95% CI: 0.17, 0.83; *P* < 0.001), specifically omega-3 [44,49,86]: ORR = 0.28 (95% CI: 0.09, 0.90; *P* = 0.03).

Not enough studies captured vomiting severity during GI cancers treated with chemotherapy.

### Risk-of-bias assessment

The Cochrane bias assessment tool was used to evaluate overall bias and within-study bias of the included studies. The general and within-study risks of bias are shown in Supplemental Figure 2. Individual within-study bias can be found in Supplemental Table 3. Half of the studies (51.0%) adequately described randomization; for (49.0%) of the studies, the randomization process was not clearly described or omitted. Methods of allocation concealment were described in (33.8%) of the studies, and most of them described a blinding method in the study design (60.4%). This is a notable strength for these meta-analyses as over half of the studies included patients blinded to the treatment group, which helps reduce bias in self-reported outcomes, particularly relevant for subjective GI symptoms. Less than half of the studies (41.0%) provided sufficient but not extensive information regarding the blinding of outcome assessment. Given the quantitative nature of the outcome variables, it is possible that the outcome measurement would be influenced by the blinding of participants. Incomplete outcome data or attrition bias was considered low if the studies had no dropouts or dropout rates did not exceed 20% of total participants. Most studies (85.6%) included in the analysis had low attrition bias. Low selective reporting (26.6%) was based on a description of outcomes as well as access to registered protocols. Highly selective reporting was true if neither description of outcomes, nor trial registration were available. Furthermore, the funnel plot and Begg’s and Egger’s test, used to assess statistically significant bias, was conducted in 20 of the 151 meta-analyses, which indicated the presence of statistical bias in 8 of the meta-analyses conducted. This raises concern for the validity of the results from the meta-analyses as this could indicate an unbalanced representation of the effects of MNT on cancer therapy-induced GI symptoms. This further skew the overall findings and compromises the validity of the conclusions.

## Discussion

**FIGURE 9:**
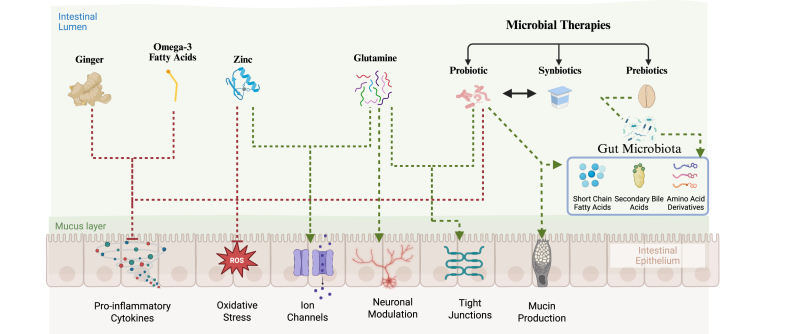
Overview—therapies in alleviating cancer-induced GI symptoms. The intestinal effects of ginger, omega-3 fatty acids, and prebiotics were attributed to reduced proinflammatory cytokine production. Zinc reduced ROS production, whereas both zinc and glutamine enhanced ion channel expression. Additionally, glutamine supported neuronal modulation. Glutamine, probiotics, and synbiotics enhanced tight junction expression, whereas probiotics and synbiotics also stimulated mucin production. Probiotics, synbiotics, and prebiotics acted through the gut microbiota, leading to increased production of short-chain fatty acids, secondary bile acids, and amino acid derivatives. GI, gastrointestinal; ROS, reactive oxygen species. Green arrows indicate processes that are enhanced, red arrows denote processes which are diminished.

This study systematically reviewed and quantitatively synthesized scientific evidence regarding nutritional therapies (nutrient supplementation, ONS, and nutrition counseling) in treating GI symptoms during cancer treatment. A total of 10,832 patients from 139 studies were included in the 151 total meta-analyses. A summary of all the statistical measures used for the meta-analyses, corresponding to FIGURE 2, FIGURE 3, FIGURE 4, FIGURE 5, FIGURE 6, FIGURE 7, FIGURE 8, is provided in Table 3. A pictorial summary of these results and their effects within the intestine is displayed in Figure 9.

**TABLE 3:** Summary of meta-analyses statistics.

Meta-analyses	Symptom	Nutrition intervention	Effect size	Lower CI	Upper CI	No. of studies	No. of participants	Statistical model	*P* value
All ONS All cancer treatments (Incidence)	Anorexia	ONS	0.72	0.34	1.52	4	207	Fixed effect	0.39
All ONS All cancer treatments (Incidence)	Diarrhea	ONS	0.87	0.53	1.42	7	522	Fixed effect	0.58
All ONS All cancer treatments (Incidence)	Mucositis	ONS	0.95	0.57	1.58	6	336	Fixed effect	0.85
All ONS All cancer treatments (Incidence)	Nausea	ONS	0.97	0.60	1.57	5	415	Fixed effect	0.91
All ONS All cancer treatments (Severity)	Diarrhea	ONS	–0.19	–0.42	0.05	4	281	Fixed effect	0.12
All ONS All cancer treatments (Severity)	Nausea	ONS	–0.06	–0.33	0.21	3	207	Fixed effect	0.68
All ONS All cancer treatments (Severity)	Anorexia	ONS	–0.04	–0.31	0.22	4	217	Fixed effect	0.74
All counseling All cancer treatments (Incidence)	Constipation	Counseling	0.15	0.08	0.29	3	273	Fixed effect	<0.001
All counseling All cancer treatments (Incidence)	Nausea	Counseling	0.27	0.15	0.47	5	530	Fixed effect	<0.001
All counseling All cancer treatments (Incidence)	Diarrhea	Counseling	0.30	0.18	0.52	5	488	Fixed effect	<0.001
All counseling All cancer treatments (Incidence)	Anorexia	Counseling	0.37	0.23	0.58	4	415	Fixed effect	<0.001
All counseling All cancer treatments (Incidence)	Vomiting	Counseling	0.49	0.29	0.83	4	389	Fixed effect	0.01
All nutrients All cancer treatments (Incidence)	Ileus	Nutrient supplementation	0.32	0.18	0.55	5	459	Fixed effect	<0.001
All nutrients All cancer treatments (Incidence)	Diarrhea	Nutrient supplementation	0.37	0.25	0.55	45	3422	Random effect	<0.001
All nutrients All cancer treatments (Incidence)	Abdominal pain	Nutrient supplementation	0.41	0.29	0.58	11	832	Fixed effect	<0.001
All nutrients All cancer treatments (Incidence)	Constipation	Nutrient supplementation	0.28	0.12	0.63	15	1102	Random effect	<0.001
All nutrients All cancer treatments (Incidence)	Anorexia	Nutrient supplementation	0.34	0.17	0.69	15	858	Random effect	<0.001
All nutrients All cancer treatments (Incidence)	Nausea	Nutrient supplementation	0.49	0.34	0.71	32	2371	Random effect	<0.001
All nutrients All cancer treatments (Incidence)	Vomiting	Nutrient supplementation	0.55	0.41	0.73	26	2013	Fixed effect	<0.001
All nutrients All cancer treatments (Incidence)	Mucositis	Nutrient supplementation	0.63	0.52	0.78	43	3249	Fixed effect	<0.001
All nutrients All cancer treatments (Incidence)	Bloating	Nutrient supplementation	0.69	0.30	1.79	7	473	Fixed effect	0.1
All nutrients All cancer treatments (Severity)	Oral Pain	Nutrient supplementation	–1.42	–1.68	–1.18	9	415	Fixed effect	<0.001
All nutrients All cancer treatments (Severity)	Mucositis	Nutrient supplementation	–0.88	–1.23	–0.54	42	2841	Random effect	<0.001
All nutrients All cancer treatments (Severity)	Anorexia	Nutrient supplementation	–0.68	–1.09	–0.26	14	935	Random effect	<0.001
All nutrients All cancer treatments (Severity)	Diarrhea	Nutrient supplementation	–0.58	–1.06	–0.11	20	1410	Random effect	0.02
All nutrients All cancer treatments (Severity)	Flatulence	Nutrient supplementation	–0.49	–0.83	–0.15	3	143	Fixed effect	<0.001
All nutrients All cancer treatments (Severity)	Abdominal pain	Nutrient supplementation	–0.37	–0.58	–0.16	5	363	Fixed effect	<0.001
All nutrients All cancer treatments (Severity)	Constipation	Nutrient supplementation	–0.38	–0.57	–0.20	7	476	Fixed effect	<0.001
All nutrients All cancer treatments (Severity)	Nausea	Nutrient supplementation	–0.35	–0.74	0.05	12	1109	Random effect	0.09
All nutrients All cancer treatments (Severity)	Vomiting	Nutrient supplementation	–0.01	–0.14	0.11	8	910	Fixed effect	0.83
All nutrients All cancer treatments	Flatus latency (h)	Nutrient supplementation	–1.16	–1.84	–0.49	13	954	Random effect	<0.001
All nutrients All cancer treatments	Defecation latency (h)	Nutrient supplementation	–1.07	–1.86	–0.31	15	1159	Random effect	0.01
All nutrients All cancer treatments	Defecation frequency	Nutrient supplementation	1.58	1.38	1.78	3	566	Fixed effect	<0.001
Abdominal pain incidence All cancer treatments	Abdominal pain	Probiotic	0.32	0.21	0.49	3	394	Fixed effect	<0.001
Abdominal pain incidence All cancer treatments	Abdominal pain	Fatty acid	1.20	0.44	3.25	3	177	Fixed effect	0.73
Anorexia incidence All cancer treatments	Anorexia	Amino acid	0.25	0.16	0.39	5	398	Fixed effect	<0.001
Anorexia incidence All cancer treatments	Anorexia	Fatty acid	0.49	0.23	1.03	3	122	Fixed effect	0.06
Anorexia severity All cancer treatments	Anorexia	Fatty acid	–0.44	–0.66	–0.22	6	313	Fixed effect	<0.001
Bloating incidence All cancer treatments	Bloating	Probiotic	0.68	0.46	1.16	4	324	Fixed effect	0.19
Constipation incidence All cancer treatments	Constipation	Amino acid	0.28	0.16	0.49	3	448	Fixed effect	<0.001
Constipation incidence All cancer treatments	Constipation	Synbiotic	0.47	0.14	1.58	3	211	Fixed effect	0.22
Constipation incidence All cancer treatments	Constipation	Fatty acid	1.28	0.51	3.25	3	106	Fixed effect	0.6
Constipation severity All cancer treatments	Constipation	Fatty acid	0.07	–0.22	0.36	5	217	Fixed effect	0.63
Defecation latency (h) All cancer treatments	Defecation latency (h)	Caffeine	–0.61	–0.88	–0.33	3	251	Fixed effect	<0.001
Defecation latency (h) All cancer treatments	Defecation latency (h)	Chewing gum	–0.52	–0.72	–0.33	7	408	Fixed effect	<0.001
Diarrhea incidence All cancer treatments	Diarrhea	Herb	0.08	0.01	0.47	3	149	Fixed effect	<0.001
Diarrhea incidence All cancer treatments	Diarrhea	Probiotic	0.30	0.15	0.61	13	1629	Random effect	<0.001
Diarrhea incidence All cancer treatments	Diarrhea	Fatty acid	0.39	0.23	0.66	7	300	Fixed effect	<0.001
Diarrhea incidence All cancer treatments	Diarrhea	Synbiotic	0.48	0.23	0.96	4	273	Fixed effect	0.04
Diarrhea incidence All cancer treatments	Diarrhea	Amino acid	0.51	0.35	0.97	10	701	Fixed effect	<0.001
Diarrhea incidence All cancer treatments	Diarrhea	Prebiotic	0.68	0.37	1.25	5	253	Fixed effect	0.19
Diarrhea severity All cancer treatment	Diarrhea	Probiotic	–0.65	–0.83	–0.48	6	551	Fixed effect	<0.001
Diarrhea severity All cancer treatment	Diarrhea	Fatty acid	–0.12	–0.31	0.08	8	417	Fixed effect	0.24
Mucositis incidence All cancer treatments	Mucositis	Fatty Acid	0.24	0.10	0.58	3	163	Fixed effect	<0.001
Mucositis incidence All cancer treatments	Mucositis	Prebiotic	0.23	0.09	0.56	4	150	Fixed effect	<0.001
Mucositis incidence All cancer treatments	Mucositis	Probiotic	0.30	0.21	0.62	4	442	Fixed effect	<0.001
Mucositis incidence All cancer treatments	Mucositis	Amino acid	0.44	0.20	0.98	11	732	Fixed effect	0.04
Mucositis incidence All cancer treatments	Mucositis	Mineral	0.62	0.42	0.99	10	920	Fixed effect	0.01
Mucositis incidence All cancer treatments	Mucositis	Chewing gum	1.23	0.81	1.88	3	410	Fixed effect	0.33
Mucositis incidence All cancer treatments	Mucositis	Herb	0.66	0.31	1.42	3	172	Fixed effect	0.29
Mucositis severity All cancer treatment	Mucositis	Prebiotic	–1.15	–1.46	–0.84	5	197	Fixed effect	<0.001
Mucositis severity All cancer treatment	Mucositis	Fatty acid	–0.86	–1.23	–0.42	3	131	Fixed effect	<0.001
Mucositis severity All cancer treatment	Mucositis	Herb	–0.87	–1.08	–0.67	9	459	Fixed effect	<0.001
Mucositis severity All cancer treatment	Mucositis	Amino acid	–0.82	–1.01	–0.64	7	478	Fixed effect	<0.001
Mucositis severity All cancer treatment	Mucositis	Probiotic	–0.73	–0.92	–0.55	4	509	Fixed effect	<0.001
Mucositis severity All cancer treatment	Mucositis	Mineral	–0.19	–0.54	0.16	11	856	Random effect	0.29
Nausea incidence All cancer treatments	Nausea	Fatty acid	0.22	0.13	0.37	8	368	Fixed effect	<0.001
Nausea incidence All cancer treatments	Nausea	Probiotic	0.27	0.17	0.44	6	413	Fixed effect	<0.001
Nausea incidence All cancer treatments	Nausea	Herb	0.70	0.50	0.98	7	640	Fixed effect	0.04
Nausea incidence All cancer treatments	Nausea	Amino acid	1.12	0.70	1.75	5	622	Fixed effect	0.63
Vomiting incidence All cancer treatments	Vomiting	Herb	0.22	0.10	0.50	6	361	Fixed effect	<0.001
Vomiting incidence All cancer treatments	Vomiting	Probiotic	0.39	0.25	0.60	7	553	Fixed effect	<0.001
Vomiting incidence All cancer treatments	Vomiting	Fatty acid	0.69	0.32	1.48	7	304	Fixed effect	0.34
Vomiting incidence All cancer treatments	Vomiting	Amino acid	1.25	0.65	2.39	3	531	Fixed effect	0.5
Diarrhea incidence gastrointestinal cancer	Diarrhea	Probiotic	0.15	0.11	0.22	5	916	Fixed effect	<0.001
Diarrhea incidence gastrointestinal cancer	Diarrhea	Prebiotic	0.50	0.25	1.02	3	184	Fixed effect	0.06
Diarrhea incidence colorectal cancer	Diarrhea	Probiotic	0.39	0.23	0.64	3	249	Fixed effect	<0.001
Mucositis severity head and neck cancer	Mucositis	Herb	–1.10	–1.32	–0.87	8	399	Fixed effect	<0.001
Mucositis severity head and neck cancer	Mucositis	Amino acid	–0.84	–1.17	–0.51	3	151	Fixed effect	<0.001
Mucositis severity head and neck cancer	Mucositis	Probiotic	–0.85	–1.07	–0.63	3	349	Fixed effect	<0.001
Mucositis severity leukemia	Mucositis	Mineral	–0.37	–0.60	–0.14	3	302	Fixed effect	<0.001
Anorexia incidence chemotherapy	Anorexia	Fatty acid	0.49	0.23	1.03	3	122	Fixed effect	0.06
Anorexia severity chemotherapy	Anorexia	Fatty acid	–0.49	–0.79	–0.22	4	216	Fixed effect	<0.001
Constipation incidence chemotherapy	Constipation	Synbiotic	0.47	0.14	1.58	3	211	Fixed effect	0.22
Constipation incidence chemotherapy	Constipation	Fatty acid	1.28	0.51	3.25	3	106	Fixed effect	0.68
Constipation severity chemotherapy	Constipation	Fatty acid	0.07	–0.22	0.36	5	217	Fixed effect	0.63
Defecation latency (h) surgical therapy	Defecation latency (h)	Caffeine	–0.61	–0.88	–0.33	3	251	Fixed effect	<0.001
Defecation latency (h) surgical therapy	Defecation latency (h)	Chewing gum	–0.52	–0.72	–0.33	7	408	Fixed effect	<0.001
Diarrhea incidence chemotherapy	Diarrhea	Probiotic	0.24	0.12	0.49	4	238	Fixed effect	<0.001
Diarrhea incidence chemotherapy	Diarrhea	Fatty acid	0.31	0.17	0.56	6	256	Fixed effect	<0.001
Diarrhea incidence chemotherapy	Diarrhea	Synbiotic	0.48	0.23	0.96	4	273	Fixed effect	0.04
Diarrhea incidence chemotherapy	Diarrhea	Amino acid	0.66	0.41	1.03	7	379	Fixed effect	0.07
Diarrhea incidence chemotherapy	Diarrhea	Prebiotic	0.84	0.26	2.72	3	150	Fixed effect	0.78
Diarrhea severity chemotherapy	Diarrhea	Probiotic	–0.78	–1.08	–0.48	3	178	Fixed effect	<0.001
Diarrhea severity chemotherapy	Diarrhea	Fatty acid	–0.06	–0.29	0.18	5	291	Fixed effect	0.63
Diarrhea incidence radiation therapy	Diarrhea	Probiotic	0.25	0.19	0.34	6	1003	Fixed effect	<0.001
Diarrhea severity radiation therapy	Diarrhea	Probiotic	–0.59	–0.80	–0.37	3	373	Fixed effect	<0.001
Flatus latency (h) surgical therapy	Flatus latency (h)	Caffeine	–1.19	–1.46	–0.92	3	247	Fixed effect	<0.001
Flatus latency (h) surgical therapy	Flatus latency (h)	Chewing gum	–0.72	–0.90	–0.55	8	537	Fixed effect	<0.001
Mucositis incidence chemotherapy	Mucositis	Fatty acid	0.24	0.10	0.58	3	163	Fixed effect	<0.001
Mucositis incidence chemotherapy	Mucositis	Amino acid	0.48	0.28	0.84	11	731	Fixed effect	0.01
Mucositis incidence chemotherapy	Mucositis	Mineral	0.64	0.41	1.00	6	556	Fixed effect	0.05
Mucositis incidence chemotherapy	Mucositis	Chewing gum	1.23	0.81	1.88	3	410	Fixed effect	0.33
Mucositis severity chemotherapy	Mucositis	Herb	–0.78	–1.08	–0.48	4	183	Fixed effect	<0.001
Mucositis severity chemotherapy	Mucositis	Mineral	–0.14	–0.30	0.03	7	613	Fixed effect	0.1
Mucositis incidence radiation therapy	Mucositis	Amino Acid	0.07	0.01	0.32	3	249	Fixed effect	<0.001
Mucositis incidence radiation therapy	Mucositis	Prebiotic	0.16	0.05	0.53	3	132	Fixed effect	<0.001
Mucositis incidence radiation therapy	Mucositis	Mineral	0.66	0.32	1.34	4	364	Fixed effect	0.25
Mucositis severity radiation therapy	Mucositis	Herb	–0.96	–1.25	–0.68	5	276	Fixed effect	<0.001
Mucositis severity radiation therapy	Mucositis	Amino acid	–0.73	–0.96	–0.49	4	309	Fixed effect	<0.001
Nausea incidence chemotherapy	Nausea	Fatty acid	0.17	0.10	0.31	7	324	Fixed effect	<0.001
Nausea incidence chemotherapy	Nausea	Probiotic	0.19	0.10	0.38	5	253	Fixed effect	<0.001
Nausea incidence chemotherapy	Nausea	Herb	0.52	0.32	0.85	5	319	Fixed effect	0.01
Nausea incidence chemotherapy	Nausea	Amino acid	1.12	0.70	1.75	5	622	Fixed effect	0.63
Nausea severity chemotherapy	Nausea	Herb	–0.27	–0.50	–0.04	5	318	Fixed effect	0.02
Vomiting incidence chemotherapy	Vomiting	Probiotic	0.20	0.10	0.40	5	253	Fixed effect	<0.001
Vomiting incidence chemotherapy	Vomiting	Herb	0.30	0.11	0.81	3	206	Fixed effect	0.02
Vomiting incidence chemotherapy	Vomiting	Fatty acid	0.51	0.21	1.21	6	260	Fixed effect	0.13
Vomiting incidence chemotherapy	Vomiting	Amino acid	1.25	0.65	2.39	3	531	Fixed effect	0.5
Anorexia severity GI cancer chemotherapy	Anorexia	Fatty acid	–0.56	–0.92	–0.20	3	124	Fixed effect	<0.001
Constipation severity GI cancer chemotherapy	Constipation	Fatty acid	0.12	–0.23	0.47	4	157	Fixed effect	0.51
Defecation latency (h) colorectal cancer surgical therapy	Defecation latency (h)	Chewing gum	–0.46	–0.57	–0.17	5	184	Fixed effect	<0.001
Diarrhea incidence GI cancer chemotherapy	Diarrhea	Fatty acid	0.28	0.14	0.55	4	176	Fixed effect	<0.001
Diarrhea incidence GI cancer chemotherapy	Diarrhea	Amino acid	0.32	0.15	0.72	3	153	Fixed effect	0.01
Diarrhea severity GI cancer chemotherapy	Diarrhea	Fatty acid	–0.78	–1.12	–0.45	4	157	Fixed effect	<0.001
Diarrhea incidence gynecological cancer radiation therapy	Diarrhea	Probiotic	0.90	0.57	1.39	4	361	Fixed effect	0.62
Diarrhea incidence GI cancer surgical therapy	Diarrhea	Probiotic	0.38	0.23	0.61	3	388	Fixed effect	<0.001
Flatus latency (h) colorectal cancer surgical therapy	Flatus latency (h)	Chewing gum	–0.87	–1.10	–0.63	6	383	Fixed effect	<0.001
Mucositis severity head and neck cancer chemotherapy	Mucositis	Herb	–0.78	–1.08	–0.48	4	183	Fixed effect	<0.001
Mucositis severity leukemia chemotherapy	Mucositis	Mineral	–0.37	–0.60	–0.14	3	302	Fixed effect	<0.001
Mucositis incidence head and neck cancer radiation therapy	Mucositis	Mineral	0.66	0.32	1.34	4	364	Fixed effect	0.14
Mucositis severity head and neck cancer radiation therapy	Mucositis	Herb	–0.96	–1.25	–0.68	4	183	Fixed effect	<0.001
Nausea incidence GI cancer chemotherapy	Nausea	Fatty acid	0.07	0.05	0.35	4	180	Fixed effect	<0.001
Oral pain head and neck cancer radiation therapy	Oral Pain	Herb	–1.21	–1.51	–0.91	5	268	Fixed effect	<0.001
Vomiting incidence GI cancer chemotherapy	Vomiting	Fatty acid	0.37	0.17	0.83	4	180	Fixed effect	<0.001

Table including the quantitative data underlying the custom summary graphs shown in FIGURE 2, FIGURE 3, FIGURE 4, FIGURE 5, FIGURE 6, FIGURE 7, FIGURE 8. These values support the visual trends presented in the summary figures. Each row represents a meta-analysis examining the relationship between a specific nutrient and a symptom. Columns include the meta-analysis, symptom, intervention, estimated mean effect size, 95% confidence interval (lower and upper bounds), number of studies, total number of participants, and corresponding *P* value.

Abbreviations: CI, confidence interval; GI, gastrointestinal; No., number; ONS, oral nutrition supplements.

ONS was not effective at reducing any GI symptoms included in these analyses. However, the low number of qualifying studies evaluated (*n* = 8 studies with *n* = 722 participants total) indicate that ONS is understudied in the context of cancer therapy-induced GI symptoms. The main forms of ONS captured in this study were Enterade, Elental, and Pepti–2000, all of which target immunomodulation. Therefore, the potential efficacy of more specialized ONS specifically targeting GI symptoms should not be ruled out at this point.

Dietary counseling was effective at reducing diarrhea, constipation, nausea, anorexia, and vomiting incidence. No sub-meta-analyses could be conducted for dietary counseling due to the highly variable dietary recommendations: low fermentable oligosaccharides, disaccharides, monosaccharides, and polyols are short-chain carbohydrates, low-lactose, immunomodulatory, and individualized recommendations with variable number of counseling sessions and intervention durations. As counseling is the most personalized nutrition intervention type, future studies could leverage the positive results of the nutrient supplementation meta-analyses to produce more effective outcomes in counseling settings.

In contrast to other approaches, specific nutrient supplementation was often effective at reducing cancer therapy-induced GI symptoms. In subanalyzes conducted according to type of nutrient, cancer, and cancer treatment—amino acid, fatty acid, probiotic, synbiotic, prebiotic, herbal, and mineral supplementations were found effective at treating a variety of GI symptoms.

Amino acid supplementation reduced mucositis, anorexia, constipation, and diarrhea. These results are widely supported by the broader scientific literature, in which amino acids are demonstrated to support gut health via several mechanisms. Amino acids such as glutamine play an important role in maintaining intestinal structure and function by serving as a primary energy source for enterocytes and enhancing cell proliferation and differentiation [170]. Glutamine was the most studied amino acid, representing 14 out of 19 total amino acid studies. Glutamine is recognized to reduce diarrhea through regulating ion channels related to water absorption in the intestine, reducing inflammation through regulation of NF-kB, and increasing tight junction protein expression [170,171]. Furthermore, the only subanalysis of amino acid supplementation that could be conducted was in the context of leukemia. Collectively, amino acid supplementation was effective at reducing mucositis during leukemia, despite wide variation in the amino acids implemented. Overall, supplementation of amino acids, particularly glutamine, seems like a promising therapy for reducing most GI symptoms during cancer treatment.

Therapies designed to modulate the gut microbiota—specifically prebiotics, probiotics, and synbiotics—reduced mucositis, diarrhea, nausea, and vomiting. However, it is difficult to attribute any of these benefits to 1 specific mechanism, given that no 2 studies implemented the same probiotic, prebiotic, or synbiotic combination. Microbial therapies elicit positive effects on the GI tract via microbial synthesis of small molecules including short-chain fatty acids, secondary bile acids, amino acid metabolites, and others in a continually growing list [172,173]. Probiotics reduced the incidence and severity of diarrhea. Probiotics exert many positive effects on the GI tract, such as promoting barrier function via increasing tight junction protein expression, increasing mucin synthesis, and suppressing proinflammatory cytokine secretion [173]. Although the dosage and strain used in each probiotic supplementation study varied widely, many included classic probiotics like *Lactobacillus spp.* and *Bifidobacterium spp*. However, low variability in effect sizes within and across studies may suggest that supplementing beneficial, live bacteria is a highly viable strategy to provide GI symptom relief during cancer therapy. Prebiotics can elicit similar effects by preferentially feeding specific, beneficial gut microbial species, whereas synbiotics provide both these live microbes and their preferred substrates simultaneously. Synbiotics reflected similar effects to those of probiotics in reducing diarrhea incidence. Conversely, prebiotics were only effective at treating mucositis incidence and severity (3 of 6 total meta-analyses). Many studies have characterized how cancer and its treatment disrupt the gut microbiota. Given the benefits of microbial therapies to reduce cancer-therapy induced GI symptoms highlighted by this analysis, future studies should mechanistically detail how these therapies reduce the incidence and severity of these symptoms.

Herbal supplementation reduced vomiting, diarrhea, mucositis, and nausea whereas mineral supplementation reduced mucositis. Ginger was the most studied herb representing about one-fourth of all herbal intervention studies (6 out of 21). Ginger consumption is recognized to modulate enteric nervous system signaling through stimulation of cholinergic and –5HT3 receptors, increasing gastric emptying (thereby reducing vomiting) [174]. Ginger also strengthens intestinal barrier function through activation of Nrf2, reducing inflammation and diarrhea [174,175]. Other herbal supplements such as curcumin, although widely studied in other contexts, were not studied enough to support a meta-analysis. Minerals including zinc sulfate can reduce mucositis by inhibiting production of reactive oxygen species through the protection of sulfhydryl groups by transition metals [176]. Additionally, zinc is important in regulating ion exchange across the cell membrane [177]. Although ginger and zinc supplementation seem effective, other herbal and mineral supplements require further investigation for their abilities to reduce cancer treatment-induced GI side-effects.

Essential fatty acids, such as omega-3 fatty acids, were demonstrated to reduce anorexia and nausea. This is unsurprising, as they are recognized to regulate immune responses via decreased synthesis of proinflammatory cytokines such as TNF, IL-1β, and IL-6 [178]. Similarly, essential fatty acids modulate structure and function of the gut microbiota, enhancing *Bifidobacterium*, reducing LPS-producing bacteria, and increasing butyrate-producers [179]. Furthermore, increased omega-3 consumption has been associated with lower serum zonulin concentrations, a marker of intestinal barrier disruption [179]. All things considered, consumption of essential fatty acids, particularly omega-3 fatty acids, could work well in combination with other nutrition therapies by reducing inflammation in the intestine.

Overall, this systematic review highlights the efficacy of MNT to relieve GI symptoms during cancer treatment and highlights the potential of implementing these therapies more-widely. Dietitians should prescribe dietary modifications that target the underlying physiology of these GI symptoms in combination with weight-sustaining approaches. Other symptoms including proctitis, enteritis, and dysgeusia lacked the necessary 3 or more studies to evaluate via meta-analysis, indicating the need for further research in these areas. A continuing challenge in implementing these strategies is highlighted by the large variability within patients across studies. Personal differences between participants, including genetics, gut microbial composition, and lifestyle factors, likely influence individual responses to these interventions. This indicates that continued patient monitoring and personalized nutrition strategies are likely needed in the context of cancer treatment, where both systemic and demographic disadvantages are recognized to influence treatment outcomes [11].

## Author contributions

The authors’ responsibilities were as follows – ZA: analyzed data and performed statistical analysis, drafted first manuscript; and all authors: designed research, conducted research, approved the final version of manuscript, edited and revised the manuscript.

## Funding

ZA was supported by a Jonathan Baldwin Turner Graduate Fellowship through the University of Illinois at Urbana-Champaign Division of Nutritional Sciences.

## Conflict of interest

ZA reports financial support was provided by College of ACES, University of Illinois at Urbana-Champaign, Jonathan Baldwin Turner Fellowship. If there are other authors, they declare that they have no known competing financial interests or personal relationships that could have appeared to influence the work reported in this paper.
