# Supplementary material for: Nutrition Interventions in the Treatment of Gastrointestinal Symptoms during Cancer Therapy: A Systematic Review and Meta-analysis
Source: Adv Nutr. 2025 Jul 22;16(9):100485. doi: 10.1016/j.advnut.2025.100485 (PMC12489516; doi:10.1016/j.advnut.2025.100485)
Supplement: Multimedia component 1 [file mmc1.pdf]

Nutrition Interventions in the Treatment of Gastrointestinal Symptoms during Cancer Therapy: A Systematic Review and Meta-Analysis Zainab Alzoubi et al. 2025

Table S1. Search Algorithm used in Scopus:

Cancer Treatment:
[“Treated For Cancer” OR (“Postoperative” AND “Cancer”) OR “Cancer Treatment” OR “Cancer Therapy” OR “Chemotherapy” OR “Radiotherapy” OR “Radiation Therapy” OR “Radiofrequency Ablation” OR “Tumor Resection” OR “Tumour Resection” OR “Immunotherapy” OR “Neoadjuvant” OR “Pelvic Radiation” OR “Pelvis Radiation” OR “Pelvic Irradiation” OR “Pelvis Irradiation” OR “Oncology Treatment”]

AND

Gastrointestinal Side-Effects:
[“Diarrhea” OR “Diarrhoea” OR “Constipation” OR “Bile Acid Malabsorption” OR “Bile Acid Diarrhea” OR “Bile Acid Diarrhoea” OR “Fat Malabsorption” OR “Steatorrhea” OR “Watery Stool” OR “Loose Stool” OR “Fecal Incontinence” OR “Faecal Incontinence” OR “Functional Gastrointestinal Disorder” OR “Gastroparesis” OR “Ileus” OR “Nausea” OR “Vomiting” OR “Enterotoxicity” OR “Gastritis” OR “Colitis” OR “Enteritis” OR “Bloating” OR “Abdominal Distention” OR “Acid Reflux” OR “Gastric Reflux” OR “Gerd” OR “Abdominal Cramp” OR “Loss Of Appetite” OR “Appetite Loss” OR “Anorexia” OR “Dysbiosis” OR “bowel obstruction” OR “motility issue” OR “motility disorder” Or “Bowel
Symptom” OR “Intestinal Motility” OR “Gastrointestinal Toxicity” OR “Dysgeusia” OR “Mucositis” OR “Dyspepsia” OR “Intestinal Permeability” OR “Xerostomia” OR “Cheilitis” OR “Gastroesophageal Reflux Disease”]

AND

Nutrient
[“Nutrition” OR “Nutrient” OR “Nutritional” OR “Diet” OR “Dietary” OR “Dietary Fiber” OR “Dietary Fibre” OR “Lignan” OR “Psyllium” OR “Fructooligosaccharide” OR “Glucan” OR “Raffinose” OR “Stachyose” OR “Dextran” OR “Probiotic” OR “Prebiotic” OR “Synbiotic” OR “Postbiotic” OR “Fat” OR “Lipids” OR “Triglyceride” OR “Triacylglycerol” OR “Diglyceride” OR “Diacylglycerol” OR “Monoglyceride” OR “Monoacylglycerol” OR “Glycerol” OR “Glyceride” OR “Omega-3” OR “Omega-6” OR “Linoleic Acid” OR “Eicosapentaenoic Acid” OR “Epa” OR “Docosahexaenoic Acid” OR “Dha” OR “Arachidonic Acid” OR “Eicosanoids” OR “Fatty Acid” OR “Saturated Fat” OR “Unsaturated Fat” OR “Monounsaturated Fat” OR “Polyunsaturated Fat” OR “Cholesterol” OR “Protein” OR “Amino Acid” OR “Polypeptide” OR “Carbohydrate” OR “Glucose” OR “Galactose” OR “Fructose” OR “Mannose” OR “Lactose” OR “Dextrose” OR “Sucrose” OR “Sugar” OR “Starch” OR “Polysaccharide” OR “Macronutrient” OR “Calories” OR “Oral Nutrition Supplement” OR “Parenteral Nutrition” OR “Enteral Nutrition” OR “Phytochemical” OR “Polyphenol” OR “Organosulfide” OR “Saponin” OR “Carotenoid” OR “Capsaicin” OR “Indole” OR “Isothiocyanate” OR “Phytosterol” OR “Flavanoid” OR “Flavonoid” OR “Isoflavone” OR “Catechine” OR “Catechin” OR “Ellagic Acid” OR “Anthocyanoside” OR “Anthocyanin” OR “Resveratrol” OR “Vitamin” OR “Thiamin” OR “Thiamine” OR “Retinol” OR “Riboflavin” OR “Ergocalciferol” OR “Alpha-Tocopherol” OR “Niacin” OR “Pantothenic Acid” OR “Phytonadione” OR “Phylloquinone”

OR “Biotin” OR “Pyridoxine” OR “Cobalamin” OR “Folate” OR “Ascorbic Acid” OR “Mineral” OR “Chromium” OR “Copper” OR “Fluoride” OR “Iodide” OR “Iron” OR “Manganese” OR “Molybdenum” OR “Selenium” OR “Zinc” OR “Calcium” OR “Potassium” OR “Sodium” OR “Chloride” OR “Phosphorus” OR "Herb" OR "Phytotherapy" OR "Root" OR "Bran"]

AND

Clinical
[“clinical” OR “human” OR “patient”]

AND NOT:

["Osteoporosis" OR "Diabetes" OR "Review" OR "Germline" OR "ulcerative colitis" OR "crohn's disease" OR "Vaccine" OR "Arrhythmia" OR "Concussion" OR "Asthma" OR "Pneumonia" OR "Drug Development" OR "Allergy" OR "Meta-analysis" OR "Heart Disease" OR "Transfection" OR "Arthritis" OR "Pharmacogenetics" OR "Insulin" OR "Encephalopathy" OR "encephalitis" OR "Hypertension" OR "Epilepsy" OR "Deep Vein Thrombosis" OR "Tuberculosis" OR "Coma" OR "Edema" OR "rickets" OR "organoid" OR "mouse" OR "rat" OR "preliminary" OR "case report" OR "case presentation" OR "postmortem" OR "Retrospective" OR "Phase 0" OR "Phase I" OR "Phase II" OR "Phase III" OR "Phase 1" OR "Phase 2" OR "Phase 3" OR "Celiac" OR "Feasibility Study" OR "Cell Culture" OR "Human Immunodeficiency Virus"]

Limit to:
Article
1961< and >2024

Table S2. Search Algorithm used in PubMed:

Cancer Treatment:
"Chemotherapy, Adjuvant"[Mesh] OR "Immunotherapy"[Mesh] OR "Radiotherapy"[Mesh] OR "Antineoplastic Protocols"[Mesh] OR “Treated For Cancer”[Tiab] OR “Cancer Treatment”[Tiab] OR “Cancer Therapy”[Tiab] OR Chemotherapy[Tiab] OR Radiotherapy[Tiab] OR “Radiation Therapy”[Tiab] OR “Radiofrequency Ablation”[Tiab] OR “Tumor Resection”[Tiab] OR “Tumour Resection”[Tiab] OR Immunotherapy[Tiab] OR Neoadjuvant[Tiab] OR "Postoperative Cancer"[Tiab:~10000] OR "Preoperative Cancer"[Tiab:~10000] OR "Operative Cancer"[Tiab:~10000] OR “Chemoradiotherapy”[Tiab] OR Neoplasm/therapy[Mesh] OR “Pelvic Radiation”[Tiab] OR “Pelvic Irradiation”[Tiab] or “Oncology Treatment”[Tiab]

AND

Gastrointestinal Side-Effects:
"Signs and Symptoms, Digestive"[Mesh] OR "Gastroenteritis"[Mesh] OR "Cachexia"[Mesh] OR "Digestive System Physiological Phenomena"[Mesh] OR "Taste Disorders"[Mesh] OR “Malnutrition”[Mesh] OR Diarrhea[Tiab] OR Diarrhoea[Tiab] OR Constipation[Tiab] OR “Bile Acid Malabsorption”[Tiab] OR “Bile Acid Diarrhea”[Tiab] OR “Bile Acid Diarrhoea”[Tiab] OR “Fat Malabsorption”[Tiab] OR Steatorrhea[Tiab] OR “Watery Stool”[Tiab] OR “Loose Stool”[Tiab] OR “Fecal Incontinence”[Tiab] OR “Faecal Incontinence”[Tiab] OR “Functional Gastrointestinal Disorder”[Tiab] OR Gastroparesis[Tiab] OR Ileus[Tiab] OR Nausea[Tiab] OR Vomiting[Tiab] OR Enterotoxicity[Tiab] OR Gastritis[Tiab] OR Colitis[Tiab] OR Enteritis[Tiab] OR Bloating[Tiab] OR “Abdominal Distention”[Tiab] OR “Acid Reflux”[Tiab] OR “Gastric Reflux”[Tiab] OR Gerd[Tiab] OR “Abdominal Cramp”[Tiab] OR “Loss Of Appetite”[Tiab] OR “Appetite Loss”[Tiab] OR Anorexia[Tiab] OR Dysbiosis[Tiab] OR “bowel obstruction”[Tiab] OR “motility disorder”[Tiab] OR mucositis[Tiab] OR dysgeusia[Tiab] OR “intestinal permeability”[Tiab] OR xerostomia[Tiab] OR dyspepsia[Tiab] OR “gastroesophageal reflux disease”[Tiab] OR cheilitis[Tiab]

AND

Nutrient
“Nutrition Therapy”[Mesh] OR “Phytotherapy”[Mesh] OR "Diet, Food, and Nutrition"[Mesh] OR “Lipids”[Mesh] OR “Carbohydrates”[Mesh] OR "Amino Acids, Peptides, and Proteins"[Mesh] OR “Minerals”[Mesh] OR “Vitamins”[Mesh] OR Nutrition[Tiab] OR Nutrient[Tiab] OR Nutritional[Tiab] OR Diet[Tiab] OR Dietary[Tiab] OR “Dietary Fiber”[Tiab] OR “Dietary Fibre”[Tiab] OR Lignan[Tiab] OR Psyllium[Tiab] OR Fructooligosaccharide[Tiab] OR Glucan[Tiab] OR Raffinose[Tiab] OR Stachyose[Tiab] OR Dextran[Tiab] OR Probiotic[Tiab] OR Prebiotic[Tiab] OR Synbiotic[Tiab] OR Postbiotic[Tiab] OR Fat[Tiab] OR Lipids[Tiab] OR Triglyceride[Tiab] OR Triacylglycerol[Tiab] OR Diglyceride[Tiab] OR Diacylglycerol[Tiab] OR Monoglyceride[Tiab] OR Monoacylglycerol[Tiab] OR Glycerol[Tiab] OR Glyceride[Tiab] OR Omega-3[Tiab] OR Omega-6[Tiab] OR “Linoleic Acid”[Tiab] OR “Eicosapentaenoic Acid”[Tiab] OR Epa[Tiab] OR “Docosahexaenoic Acid”[Tiab] OR Dha[Tiab] OR “Arachidonic Acid”[Tiab] OR Eicosanoids[Tiab] OR “Fatty Acid”[Tiab] OR “Saturated Fat”[Tiab] OR “Unsaturated Fat”[Tiab] OR “Monounsaturated Fat”[Tiab] OR “Polyunsaturated Fat”[Tiab] OR Cholesterol[Tiab] OR Protein[Tiab] OR “Amino Acid”[Tiab] OR Polypeptide[Tiab] OR Carbohydrate[Tiab] OR Glucose[Tiab] OR Galactose[Tiab] OR Fructose[Tiab] OR Mannose[Tiab] OR Lactose[Tiab] OR Dextrose[Tiab] OR Sucrose[Tiab] OR Sugar[Tiab] OR Starch[Tiab] OR Polysaccharide[Tiab] OR Macronutrient[Tiab] OR Calories[Tiab] OR “Oral Nutrition Supplement”[Tiab] OR “Enteral Nutrition”[Tiab] OR Phytochemical[Tiab] OR Polyphenol[Tiab] OR Organosulfide[Tiab] OR Saponin[Tiab] OR Carotenoid[Tiab] OR Capsaicin[Tiab] OR Indole[Tiab] OR Isothiocyanate[Tiab] OR Phytosterol[Tiab] OR Flavanoid[Tiab] OR Flavonoid[Tiab] OR Isoflavone[Tiab] OR Catechine[Tiab] OR Catechin[Tiab] OR “Ellagic Acid”[Tiab] OR Anthocyanoside[Tiab] OR Anthocyanin[Tiab] OR Resveratrol[Tiab] OR Vitamin[Tiab] OR Thiamin[Tiab] OR Thiamine[Tiab] OR Retinol[Tiab] OR Riboflavin[Tiab] OR Ergocalciferol[Tiab] OR Alpha-Tocopherol[Tiab] OR

Niacin[Tiab] OR “Pantothenic Acid”[Tiab] OR Phytonadione[Tiab] OR Phylloquinone[Tiab] OR Biotin[Tiab] OR Pyridoxine[Tiab] OR Cobalamin[Tiab] OR Folate[Tiab] OR “Ascorbic Acid”[Tiab] OR Mineral[Tiab] OR Chromium[Tiab] OR Copper[Tiab] OR Fluoride[Tiab] OR Iodide[Tiab] OR Iron[Tiab] OR Manganese[Tiab] OR Molybdenum[Tiab] OR Selenium[Tiab] OR Zinc[Tiab] OR Calcium[Tiab] OR Potassium[Tiab] OR Sodium[Tiab] OR Chloride[Tiab] OR Phosphorus[Tiab] OR bran[Tiab] OR “Plant Extracts”[Mesh] OR Herb[Tiab] OR Root[Tiab]

AND:

Clinical

Clinical[Text Word] OR Human*[Text word] OR Patient*[Text word]

AND NOT:

Osteoporosis[Tiab] OR Diabetes[Tiab] OR Review[Tiab] OR Germline[Tiab] OR “ulcerative colitis”[Tiab] OR “crohn’s disease”[Tiab] OR “inflammatory bowel disease”[Tiab] OR Vaccine[Tiab] OR Arrhythmia[Tiab] OR Concussion[Tiab] OR Asthma[Tiab] OR Pneumonia[Tiab] OR “Drug Development”[Tiab] OR Allergy[Tiab] OR Meta-analysis[Tiab] OR “Heart Disease”[Tiab] OR Arthritis[Tiab] OR Pharmacogenetics[Tiab] OR Insulin[Tiab] OR Encephalopathy[Tiab] OR encephalitis[Tiab] OR Hypertension[Tiab] OR Epilepsy OR “Deep Vein Thrombosis” [Tiab] OR Tuberculosis[Tiab] OR Coma[Tiab] OR Edema[Tiab] OR rickets[Tiab] OR organoid[Tiab] OR mouse[Tiab] OR rat[Tiab] OR Mice[Tiab] OR retrospective[Tiab] OR preliminary[Tiab] OR “case report”[Tiab] OR “case presentation”[Tiab] OR postmortem[Tiab] OR fasting[Tiab] OR parenteral[Tiab] OR “Crossover”[Tiab] OR “Meta-analysis”[Publication Type] OR Review[Publication Type] OR “Systematic Review”[Publication Type] OR "Clinical Trial, Phase I" [Publication Type] OR "Clinical Trial, Phase II" [Publication Type] OR "Clinical Trial, Phase III" [Publication Type] OR "Clinical Trial, Phase IV" [Publication Type] OR "Case Reports" [Publication Type] OR "Letter" [Publication Type] OR “Pharmacokinetics”[Tiab] OR “Prognostic”[Tiab] OR “Agonist”[Tiab] OR “Antagonist”[Tiab] OR "Cross-Over Studies"[Mesh] OR Transfection[Tiab] OR "Feasibility Study"[Tiab] OR "Cell Culture"[Tiab] OR "Human Immunodeficiency Virus"[Tiab]

Limit to:
English, Exclude Preprints,
1946-2023

TableS3. General risk of bias.

	Random Sequence Generation	Allocation Concealment	Blinding of participants and personnel	Blinding of outcome assessment	Incomplete Outcome Data	Selective Reporting
Abdollahi, R., et al. (2019).	+	-	-	-	+	+
Aredes, M. A., et al. (2019).	-	-	+	+	+	?
Arslan, M. and L. Ozdemir (2015).	-	?	-	-	+	-
Aziz, L. M. A., et al. (2021)	?	?	-	-	+	?
Bossi, P., et al. (2017).	-	+	+	?	-	-
Bye, A., et al. (1992).	?	?	-	-	+	-
Camargo, C. D. Q., et al. (2019).	+	+	+	+	+	?
Carr, A. C., et al. (2022).	+	+	+	-	+	?
Chattopadhyay, S., et al. (2014).	?	?	-	-	+	-
Chen, M., et al. (2023).	+	+	+	+	+	?
Chitapanarux, I., et al. (2010).	+	?	+	?	+	?
Coghlin Dickson, T. M., et al. (2000).	+	?	+	+	+	?
de Loera-Rodríguez, L. H., et al. (2018).	+	?	+	?	+	-
de Luis, D. A., et al. (2007).	?	?	+	?	+	-

TableS3. Continued.

	Random Sequence Generation	Allocation Concealment	Blinding of participants and personnel	Blinding of outcome assessment	Incomplete Outcome Data	Selective Reporting
de Souza, A. P. S., et al. (2021).	+	?	-	-	+	+
Delia, P., et al. (2007).	?	?	+	?	+	-
Demers, M., et al. (2014).	+	?	+	+	+	+
Du, S. X., et al. (2018).	?	?	?	?	+	-
Ertas, I. E., et al. (2013).	+	+	-	-	-	+
Ferreira, P. R., et al. (2004).	-	+	+	?	+	-
Filipp, Z. D., et al. (2021).	?	?	+	?	+	+
Fukaya, M., et al. (2021).	+	?	-	-	+	?
Gandemer, V., et al. (2007).	+	?	+	-	+	?
Garcia-Peris, P., et al. (2016).	?	?	+	+	+	?
Giger-Pabst, U., et al. (2013).	?	+	+	-	+	?
Giralt, J., et al. (2008).	+	?	+	?	-	?
Güngördük, K., et al. (2017).	-	+	-	+	+	+
Hershman, D. L., et al. (2013).	?	?	+	?	+	?

TableS3. Continued.

	Random Sequence Generation	Allocation Concealment	Blinding of participants and personnel	Blinding of outcome assessment	Incomplete Outcome Data	Selective Reporting
Hussein, R. M., et al. (2023).	?	?	+	+	?	?
Ikeguchi, M., et al. (2011).	?	?	-	-	+	?
Islambulchilar, M., et al. (2015)	?	?	+	?	+	-
Itoh, Y., et al. (2015).	?	?	+	?	+	+
Jiang, W., et al. (2018).	+	?	-	+	+	?
Katada, C., et al. (2021).	?	?	?	?	+	+
Kim, H. S., et al. (2017).	?	?	+	?	+	+
Kobayashi, M., et al. (2020).	?	+	-	-	+	+
Kokkonen, J., et al. (2002).	?	+	-	-	+	-
Kucuktulu, E., et al. (2013).	?	?	+	?	+	-
Law, K. S., et al. (2014).	?	?	-	-	+	-
Li, G. H., et al. (2010).	-	?	?	?	+	-
Li, X., et al. (2018).	-	+	+	-	+	?
Li, Y., et al. (2006).	?	?	+	?	+	-

TableS3. Continued.

	Random Sequence Generation	Allocation Concealment	Blinding of participants and personnel	Blinding of outcome assessment	Incomplete Outcome Data	Selective Reporting
Liu, Z., et al. (2015).	+	+	+	+	+	-
Liu, Z., et al. (2011).	+	+	+	?	+	-
Lustberg, M. B., et al. (2018).	+	?	+	?	+	?
Mahajan, M. K. and V. Singh (1998).	?	?	+	?	+	-
Mahdavi, R., et al. (2013).	?	?	+	?	+	-
Mansouri-Tehrani, H. S., et al. (2016).	-	?	-	-	+	+
Mego, M., et al. (2015).	-	?	+	+	+	?
Mocellin, M. C., et al. (2017).	+	?	+	+	+	+
Motoori, M., et al. (2017).	+	-	-	-	+	+
Mücke, R., et al. (2006).	?	?	-	-	+	?
Pillai, A. K., et al. (2011).	+	?	+	?	?	+
Rathe, M., et al. (2020).	+	+	+	?	+	+
Ravasco, P., et al. (2005).	+	+	-	+	+	-
Ravasco, P., et al. (2005).	+	+	-	+	+	-

TableS3. Continued.

	Random Sequence Generation	Allocation Concealment	Blinding of participants and personnel	Blinding of outcome assessment	Incomplete Outcome Data	Selective Reporting
Reyna-Figueroa, J., et al. (2019).	+	?	-	-	+	?
Rodríguez-Padilla, Á., et al. (2021).	+	?	+	?	+	-
Rohr, U. D., et al. (2012).	?	?	+	?	+	-
Rosli, D., et al. (2021).	+	?	+	+	-	?
Salminen, E., et al. (1988).	?	?	-	-	+	-
Sánchez-Lara, K., et al. (2014).	+	+	+	+	-	?
Sangthawan, D., et al. (2013).	+	-	+	?	+	?
Sharma, A., et al. (2012).	+	-	+	?	-	+
Sieja, K. and M. Talerczyk (2004).	?	?	+	?	+	-
Sim, E., et al. (2022).	?	?	-	-	-	-
Smit, J. M., et al. (1986).	-	-	-	-	+	-
Solís-Martínez, O., et al. (2018).	?	?	-	-	+	-
Soto-Lugo, J. H., et al. (2017).	?	?	-	-	+	-
Strasser, F., et al. (2008).	+	?	+	+	-	+

TableS3. Continued.

	Random Sequence Generation	Allocation Concealment	Blinding of participants and personnel	Blinding of outcome assessment	Incomplete Outcome Data	Selective Reporting
Tian, Y., et al. (2019).	+	?	+	+	+	+
Toyomasu, Y., et al. (2019).	+	?	-	+	-	?
Tsuchiya, T., et al. (2016).	?	-	-	-	+	-
Valadares, F., et al. (2013).	+	?	+	?	+	-
Werida, R. H., et al. (2022).	?	+	+	?	+	+
Xie, H., et al. (2018).	?	?	?	?	+	-
You, W. C., et al. (2009).	-	?	?	?	-	-
Zhao, R., et al. (2017).	?	+	-	-	+	-
Zou, J. Y., et al. (2016).	?	?	-	+	+	-
Aghamohammadi, A., et al. (2018).	+	?	+	+	+	?
Al-Kharabsheh, M., et al. (2023).	+	?	-	-	+	?
Al-Taie, A. and A. Koseoglu (2021).	?	+	-	-	-	-
Anandhi, P., et al. (2020).	?	+	+	+	+	-
Asao, T., et al. (2002).	-	-	-	-	+	-

TableS3. Continued.

	Random Sequence Generation	Allocation Concealment	Blinding of participants and personnel	Blinding of outcome assessment	Incomplete Outcome Data	Selective Reporting
Babaee, N., et al. (2013).	-	-	+	+	-	-
Badr, L. K., et al. (2023).	-	-	+	-	+	+
Bozzetti, F., et al. (1997).	-	+	+	+	+	-
Charalambous, M., et al. (2018).	+	+	-	-	+	?
Choi, H., et al. (2014).	-	-	-	-	+	?
Choi, K., et al. (2007).	+	-	-	-	+	-
Crichton, M., et al. (2023).	?	+	+	+	+	+
Daniele, B., et al. (2001).	+	+	+	+	+	?
de la Rosa Oliva, F., et al. (2019).	?	?	+	+	+	-
Dulskas, A., et al. (2015).	+	+	-	-	+	?
Eghbali, A., et al. (2016).	?	-	-	-	+	-
Eghbali, A., et al. (2023).	+	-	+	+	+	?
Elkerm, Y. and R. Tawashi (2014).	-	-	-	-	+	?
Faramarzi, E., et al. (2017).	+	+	+	+	+	-

TableS3. Continued.

	Random Sequence Generation	Allocation Concealment	Blinding of participants and personnel	Blinding of outcome assessment	Incomplete Outcome Data	Selective Reporting
Fernandes, P. M., et al. (2022).	+	-	+	+	+	?
Ge, B., et al. (2017).	+	-	-	-	+	+
Harada, K., et al. (2019).	?	?	-	-	?	?
Hashemipour, M. A., et al. (2017).	+	-	+	+	+	-
Hirayama, I., et al. (2006).	?	?	-	-	+	-
Hsu, Y.-C. and S.-Y. Szu (2022).	+	-	+	-	+	?
Huang, C.-J., et al. (2019).	+	-	+	+	+	+
Huang, E. Y., et al. (2000).	?	-	+	+	+	-
Jafarimanesh, H., et al. (2020).	+	+	+	+	+	?
Jahangard-Rafsanjani, Z., et al. (2013).	?	?	+	+	+	?
Jayalekshmi, J. L., et al. (2016).	?	+	+	-	+	-
Jiang, C., et al. (2019).	+	+	+	+	+	?
Karabey, T., et al. (2022).	-	-	-	-	+	-
Khazaei, Y., et al. (2023).	+	+	+	+	+	+

TableS3. Continued.

	Random Sequence Generation	Allocation Concealment	Blinding of participants and personnel	Blinding of outcome assessment	Incomplete Outcome Data	Selective Reporting
Kia, S. J., et al. (2021).	+	+	+	+	+	?
Kobayashi, T., et al. (2015).	?	-	-	-	-	?
Kooshyar, M. M., et al. (2017).	+	-	+	+	+	-
Laali, E., et al. (2020).	?	-	+	+	-	?
Lages, P. C., et al. (2018).	+	+	+	+	+	+
Lin, L.-C., et al. (2006).	+	?	+	+	+	-
Liu, D., et al. (2021).	+	+	-	-	+	+
Manifar, S., et al. (2022).	+	+	+	+	-	?
Mansouri, A., et al. (2012).	+	+	+	+	+	?
Mohammadi, F., et al. (2022).	+	+	+	+	+	?
Mokhtar, G. M., et al. (2010).	-	-	+	+	+	-
Mutluay Yayla, E., et al. (2016).	-	-	-	-	+	?
Oshvandi, K., et al. (2021).	+	+	+	+	-	+
Panahi, Y., et al. (2012).	+	+	-	-	-	?

TableS3. Continued.

	Random Sequence Generation	Allocation Concealment	Blinding of participants and personnel	Blinding of outcome assessment	Incomplete Outcome Data	Selective Reporting
Phutsisen, J., et al. (2019).	+	+	-	+	+	+
Quah, H. M., et al. (2006).	+	+	-	-	+	?
Rambod, M., et al. (2018).	+	+	+	+	+	+
Rashad, U. M., et al. (2009).	?	-	-	-	+	-
Sahebnasagh, M., et al. (2023).	+	+	+	+	+	+
Shah, D., et al. (2023).	+	+	+	+	+	+
Sittitrai, P., et al. (2021).	+	-	+	+	+	+
Soltani, G. M., et al. (2020).	+	?	+	+	+	+
Thomas, P. L., et al. (2023).	+	+	-	-	+	+
Widjaja, N. A., et al. (2020).	?	-	+	+	+	-
Yanagimoto, H., et al. (2023).	+	+	+	+	+	+
Gholizadeh, N., et al. (2017).	?	?	+	+	+	+
Wei, H., et al. (2023).	+	+	+	+	+	+

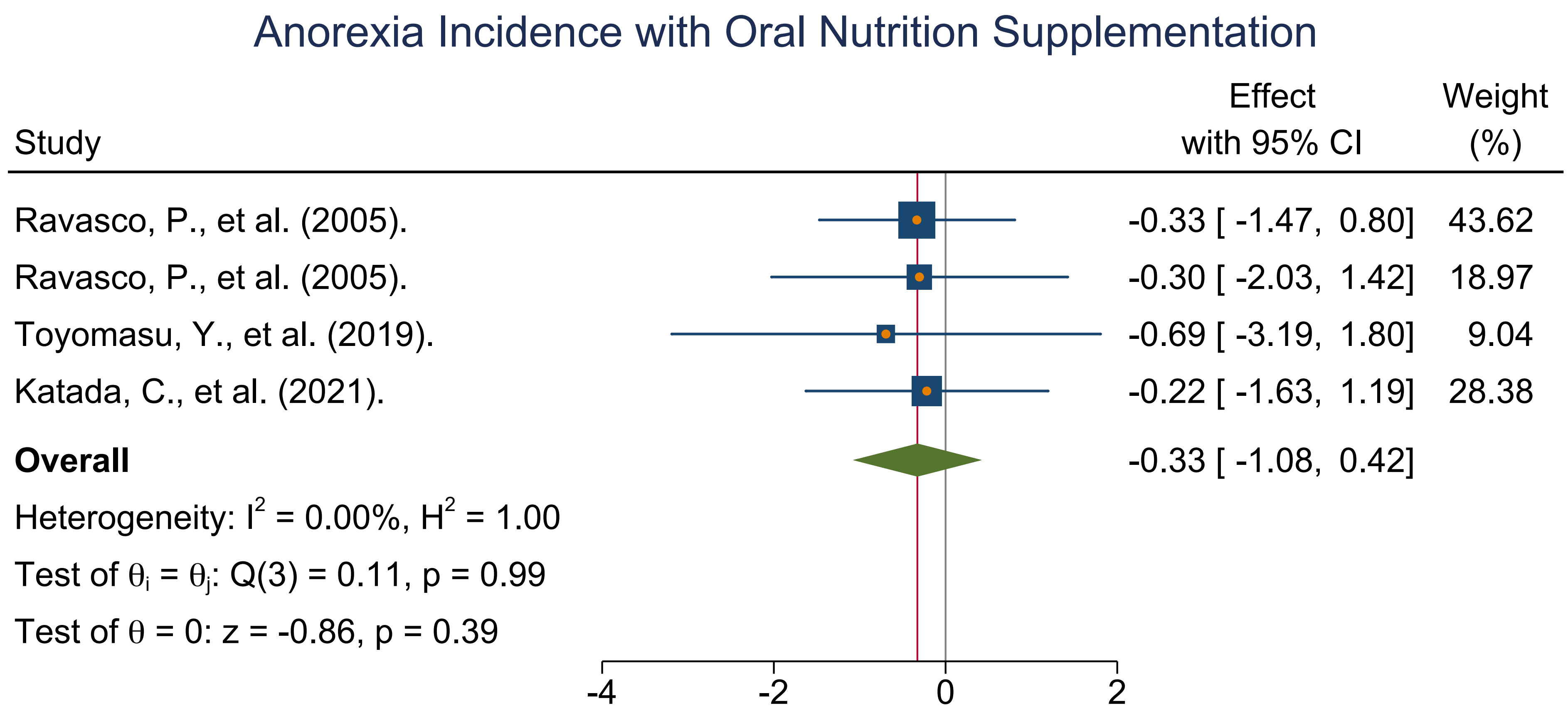
Anorexia Incidence with Oral Nutrition Supplementation
Study
Effect with 95% CI
Weight (%)
Ravasco, P., et al. (2005). -0.33 [-1.47, 0.80] 43.62
Ravasco, P., et al. (2005). -0.30 [-2.03, 1.42] 18.97
Toyomasu, Y., et al. (2019). -0.69 [-3.19, 1.80] 9.04
Katada, C., et al. (2021). -0.22 [-1.63, 1.19] 28.38
Overall -0.33 [-1.08, 0.42]
Heterogeneity: I^2 = 0.00%, H^2 = 1.00
Test of $\theta_i = \theta_j$: Q(3) = 0.11, p = 0.99
Test of $\theta = 0$: z = -0.86, p = 0.39
-4
-2
0
2

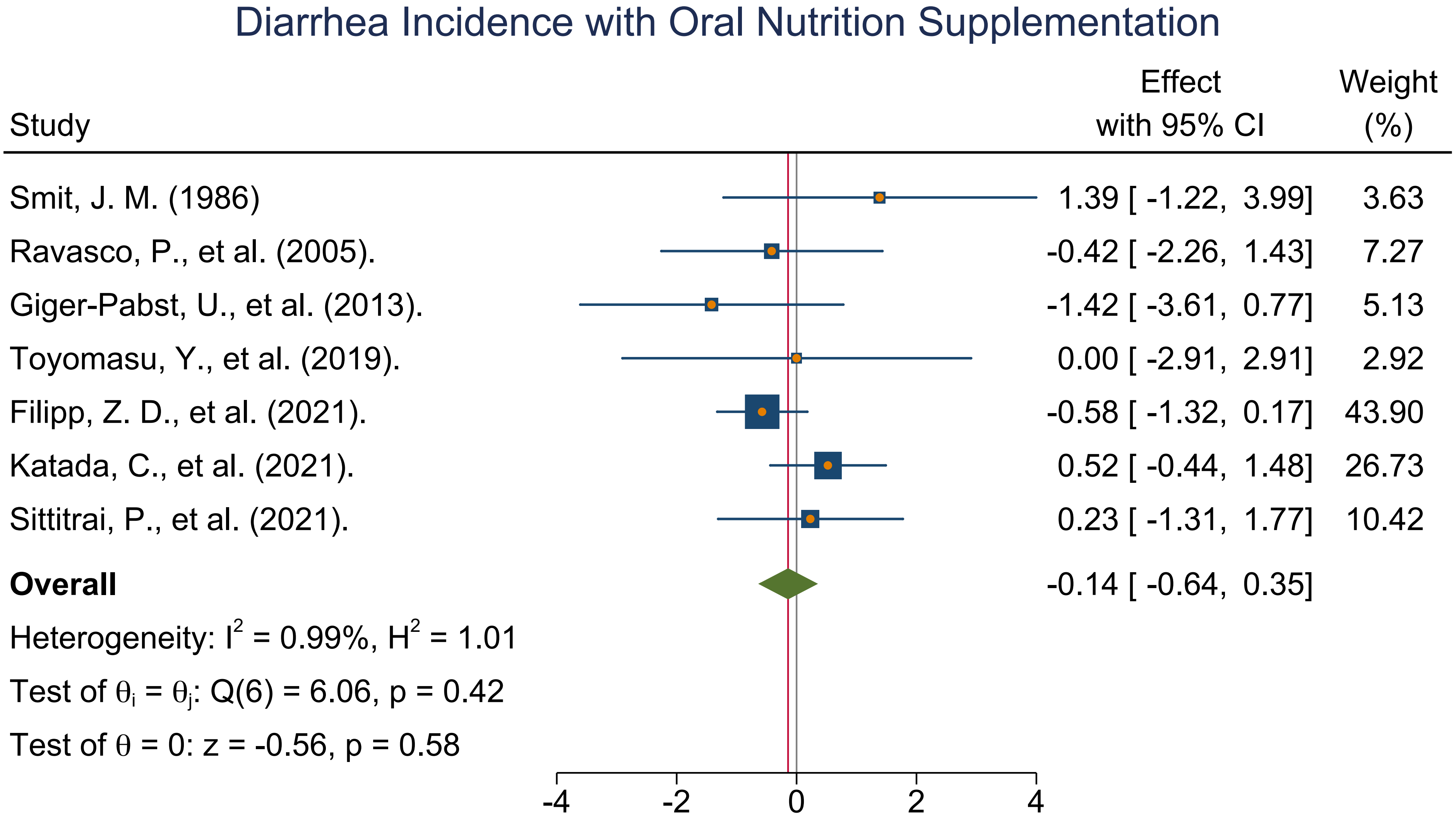
Diarrhea Incidence with Oral Nutrition Supplementation
Study
Effect with 95% CI
Weight (%)
Smit, J. M. (1986) 1.39 [-1.22, 3.99] 3.63
Ravasco, P., et al. (2005). -0.42 [-2.26, 1.43] 7.27
Giger-Pabst, U., et al. (2013). -1.42 [-3.61, 0.77] 5.13
Toyomasu, Y., et al. (2019). 0.00 [-2.91, 2.91] 2.92
Filipp, Z. D., et al. (2021). -0.58 [-1.32, 0.17] 43.90
Katada, C., et al. (2021). 0.52 [-0.44, 1.48] 26.73
Sittitrai, P., et al. (2021). 0.23 [-1.31, 1.77] 10.42
Overall -0.14 [-0.64, 0.35]
Heterogeneity: I^2 = 0.99%, H^2 = 1.01
Test of $\theta_i = \theta_j$: Q(6) = 6.06, p = 0.42
Test of θ = 0: z = -0.56, p = 0.58
-4
-2
0
2
4

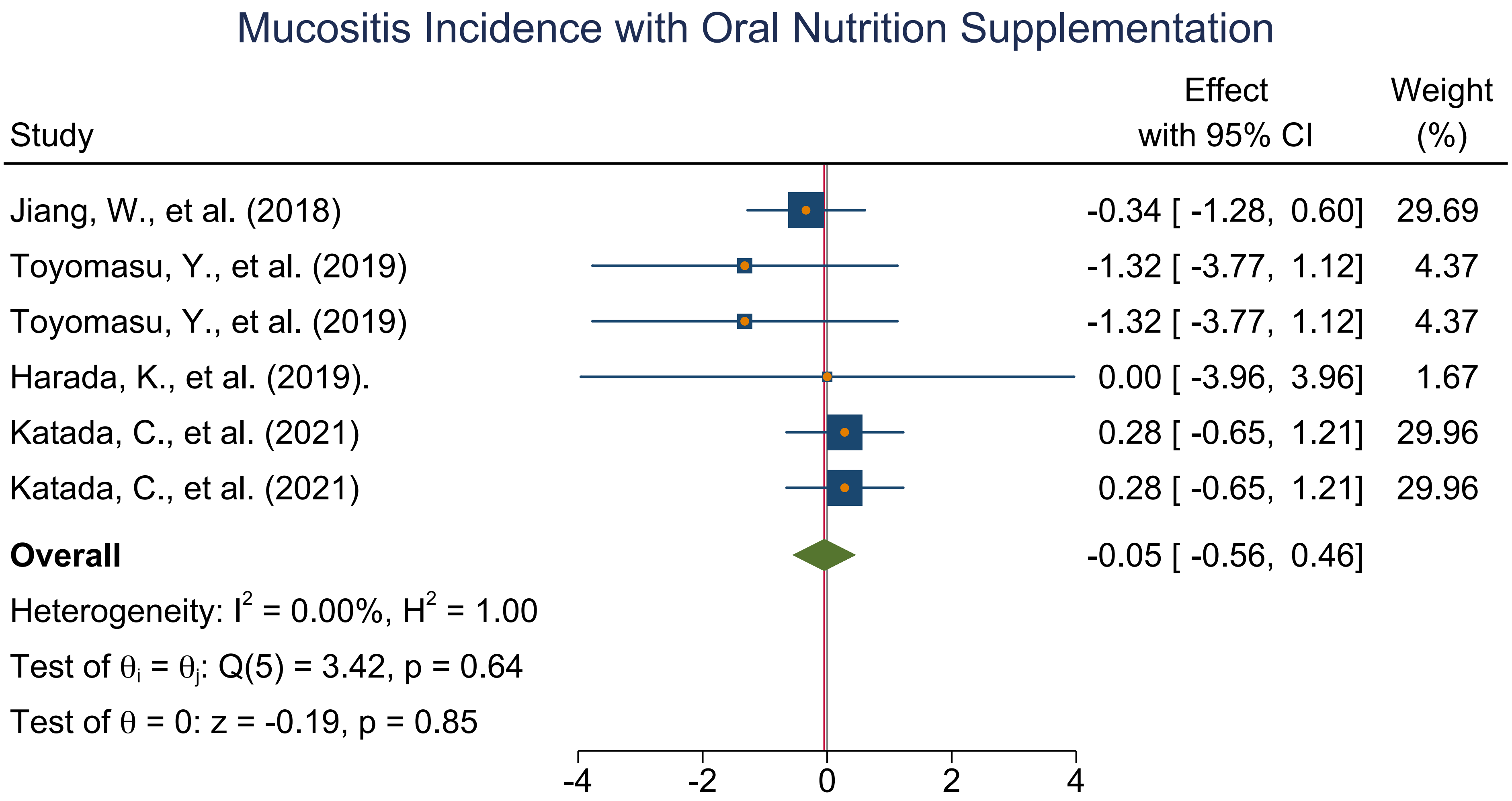
Mucositis Incidence with Oral Nutrition Supplementation
Study
Effect with 95% CI
Weight (%)
Jiang, W., et al. (2018) -0.34 [-1.28, 0.60] 29.69
Toyomasu, Y., et al. (2019) -1.32 [-3.77, 1.12] 4.37
Toyomasu, Y., et al. (2019) -1.32 [-3.77, 1.12] 4.37
Harada, K., et al. (2019). 0.00 [-3.96, 3.96] 1.67
Katada, C., et al. (2021) 0.28 [-0.65, 1.21] 29.96
Katada, C., et al. (2021) 0.28 [-0.65, 1.21] 29.96
Overall -0.05 [-0.56, 0.46]
Heterogeneity: I^2 = 0.00%, H^2 = 1.00
Test of $\theta_i = \theta_j$: Q(5) = 3.42, p = 0.64
Test of θ = 0: z = -0.19, p = 0.85
-4
-2
0
2
4

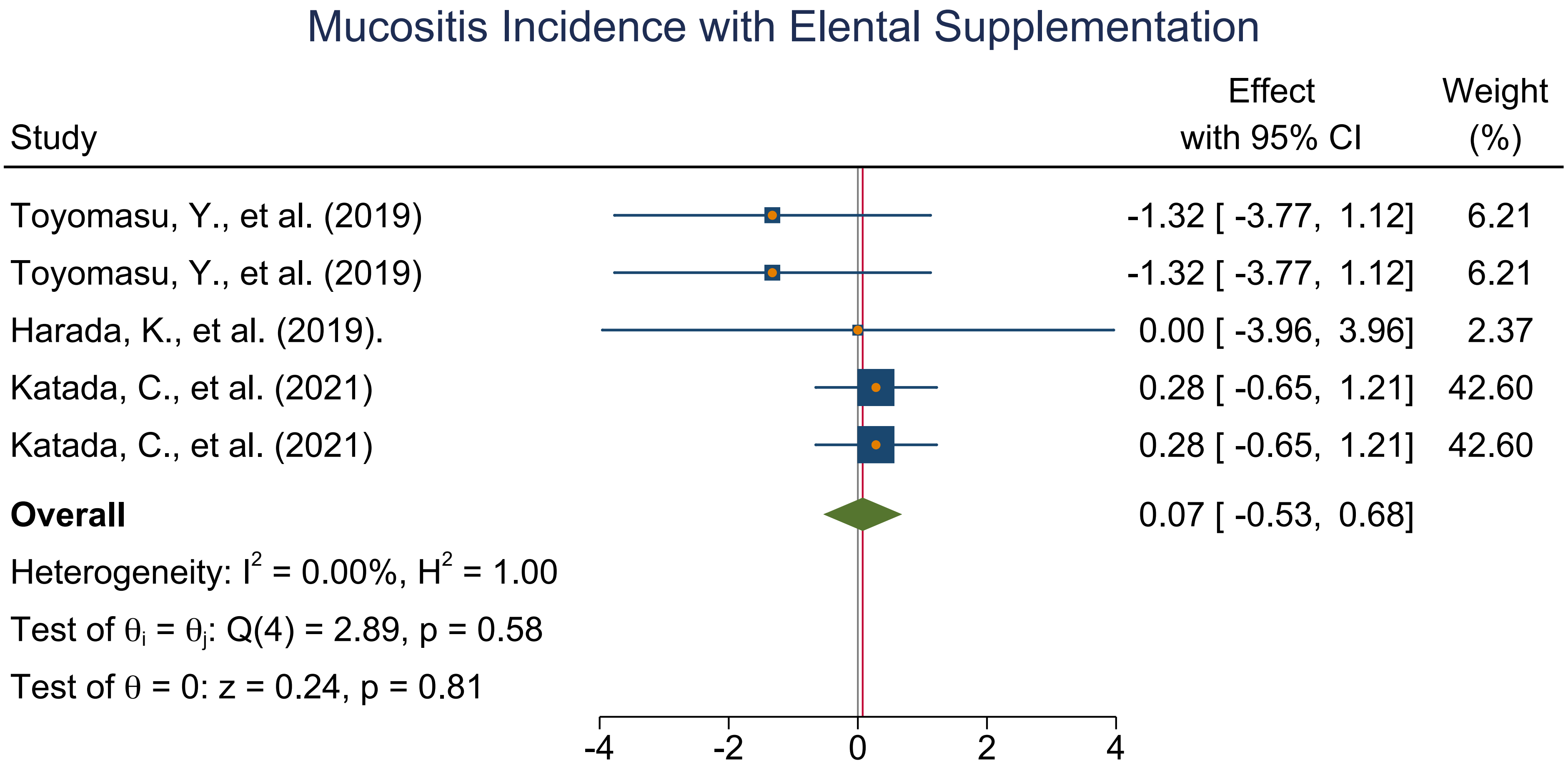
Mucositis Incidence with Elental Supplementation
Study
Effect with 95% CI
Weight (%)
Toyomasu, Y., et al. (2019) -1.32 [-3.77, 1.12] 6.21
Toyomasu, Y., et al. (2019) -1.32 [-3.77, 1.12] 6.21
Harada, K., et al. (2019). 0.00 [-3.96, 3.96] 2.37
Katada, C., et al. (2021) 0.28 [-0.65, 1.21] 42.60
Katada, C., et al. (2021) 0.28 [-0.65, 1.21] 42.60
Overall 0.07 [-0.53, 0.68]
Heterogeneity: I^2 = 0.00%, H^2 = 1.00
Test of $\theta_i = \theta_j$: Q(4) = 2.89, p = 0.58
Test of θ = 0: z = 0.24, p = 0.81
-4
-2
0
2
4

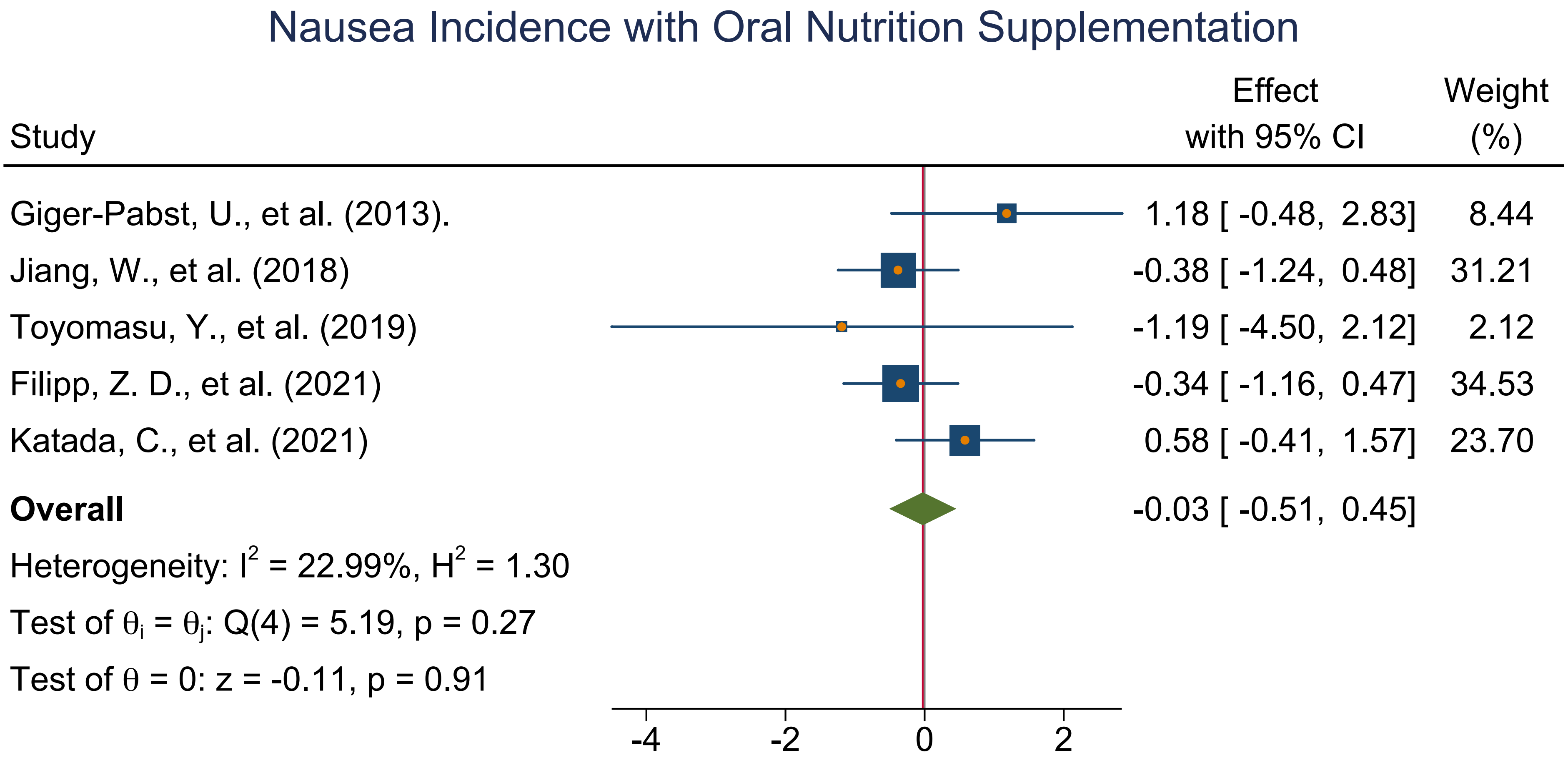

Nausea Incidence with Oral Nutrition Supplementation
Study
Effect with 95% CI
Weight (%)
Giger-Pabst, U., et al. (2013). 1.18 [-0.48, 2.83] 8.44
Jiang, W., et al. (2018) -0.38 [-1.24, 0.48] 31.21
Toyomasu, Y., et al. (2019) -1.19 [-4.50, 2.12] 2.12
Filipp, Z. D., et al. (2021) -0.34 [-1.16, 0.47] 34.53
Katada, C., et al. (2021) 0.58 [-0.41, 1.57] 23.70
Overall -0.03 [-0.51, 0.45]
Heterogeneity: I^2 = 22.99%, H^2 = 1.30
Test of $\theta_i = \theta_j$: Q(4) = 5.19, p = 0.27
Test of θ = 0: z = -0.11, p = 0.91
-4
-2
0
2

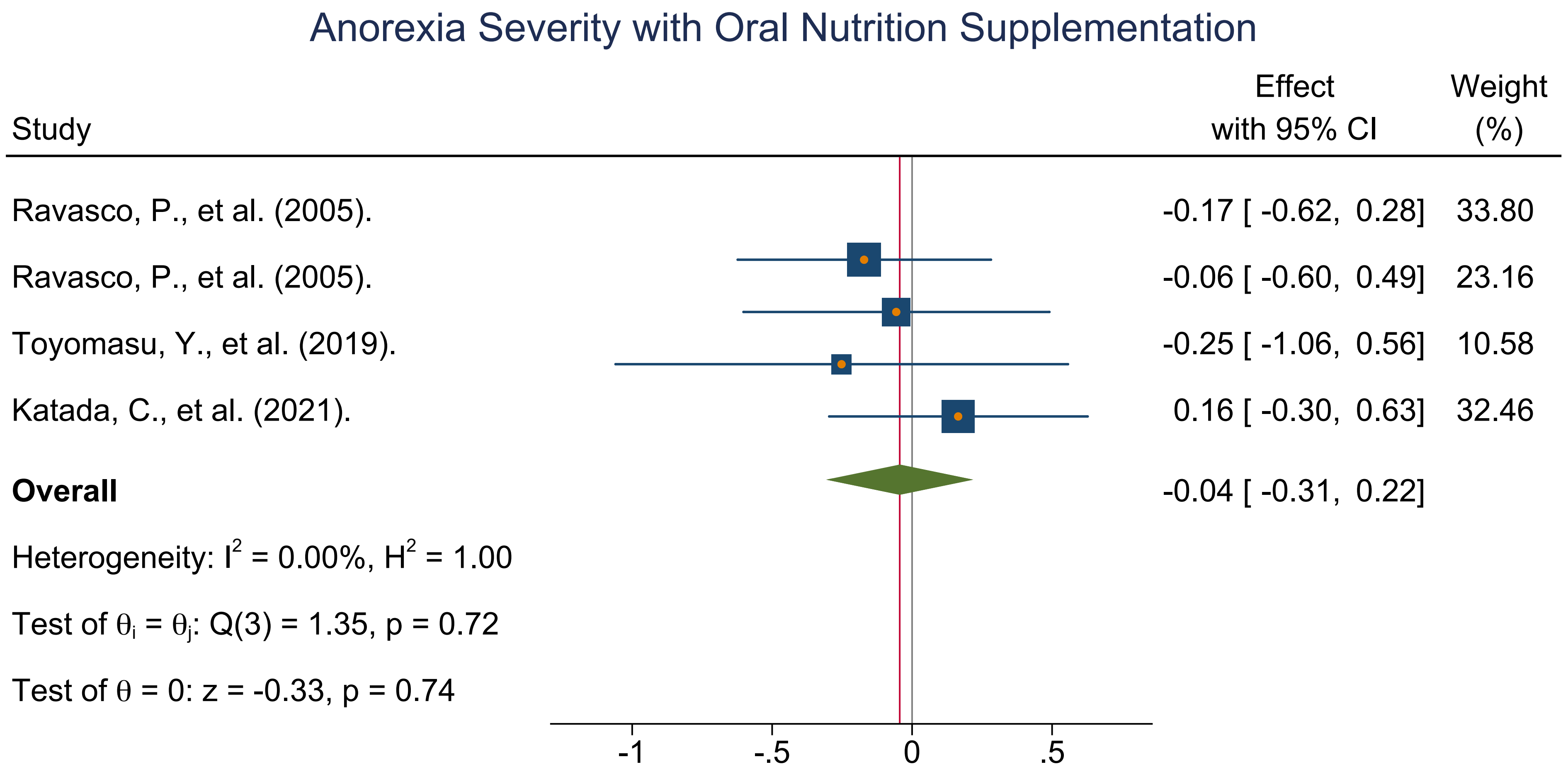
Anorexia Severity with Oral Nutrition Supplementation
Study
Effect with 95% CI
Weight (%)
Ravasco, P., et al. (2005). -0.17 [-0.62, 0.28] 33.80
Ravasco, P., et al. (2005). -0.06 [-0.60, 0.49] 23.16
Toyomasu, Y., et al. (2019). -0.25 [-1.06, 0.56] 10.58
Katada, C., et al. (2021). 0.16 [-0.30, 0.63] 32.46
Overall -0.04 [-0.31, 0.22]
Heterogeneity: I^2 = 0.00%, H^2 = 1.00
Test of $\theta_i = \theta_j$: Q(3) = 1.35, p = 0.72
Test of θ = 0: z = -0.33, p = 0.74
-1
-.5
0
.5

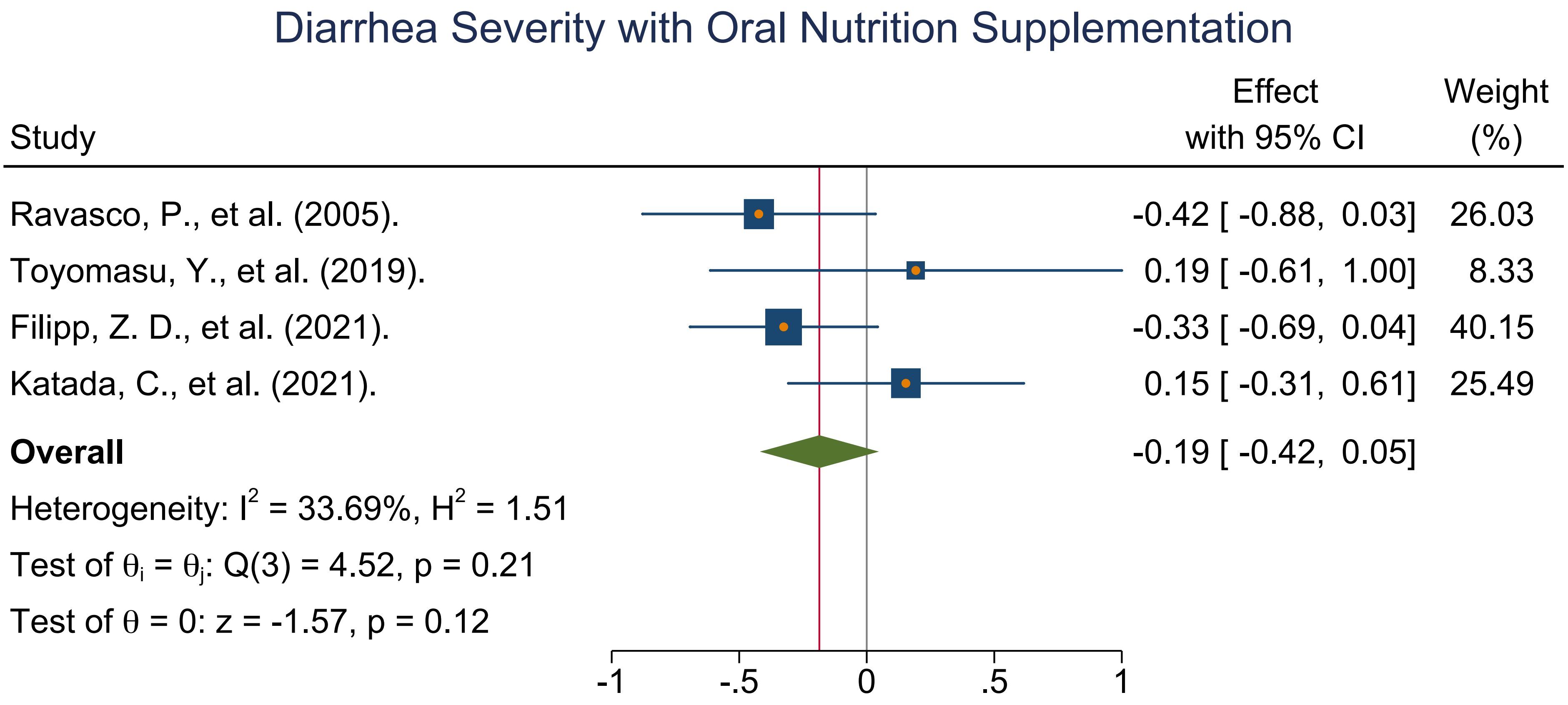
Diarrhea Severity with Oral Nutrition Supplementation
Study
Effect with 95% CI
Weight (%)
Ravasco, P., et al. (2005). -0.42 [-0.88, 0.03] 26.03
Toyomasu, Y., et al. (2019). 0.19 [-0.61, 1.00] 8.33
Filipp, Z. D., et al. (2021). -0.33 [-0.69, 0.04] 40.15
Katada, C., et al. (2021). 0.15 [-0.31, 0.61] 25.49
Overall -0.19 [-0.42, 0.05]
Heterogeneity: I^2 = 33.69%, H^2 = 1.51
Test of $\theta_i = \theta_j$: Q(3) = 4.52, p = 0.21
Test of θ = 0: z = -1.57, p = 0.12
-1
-.5
0
.5
1

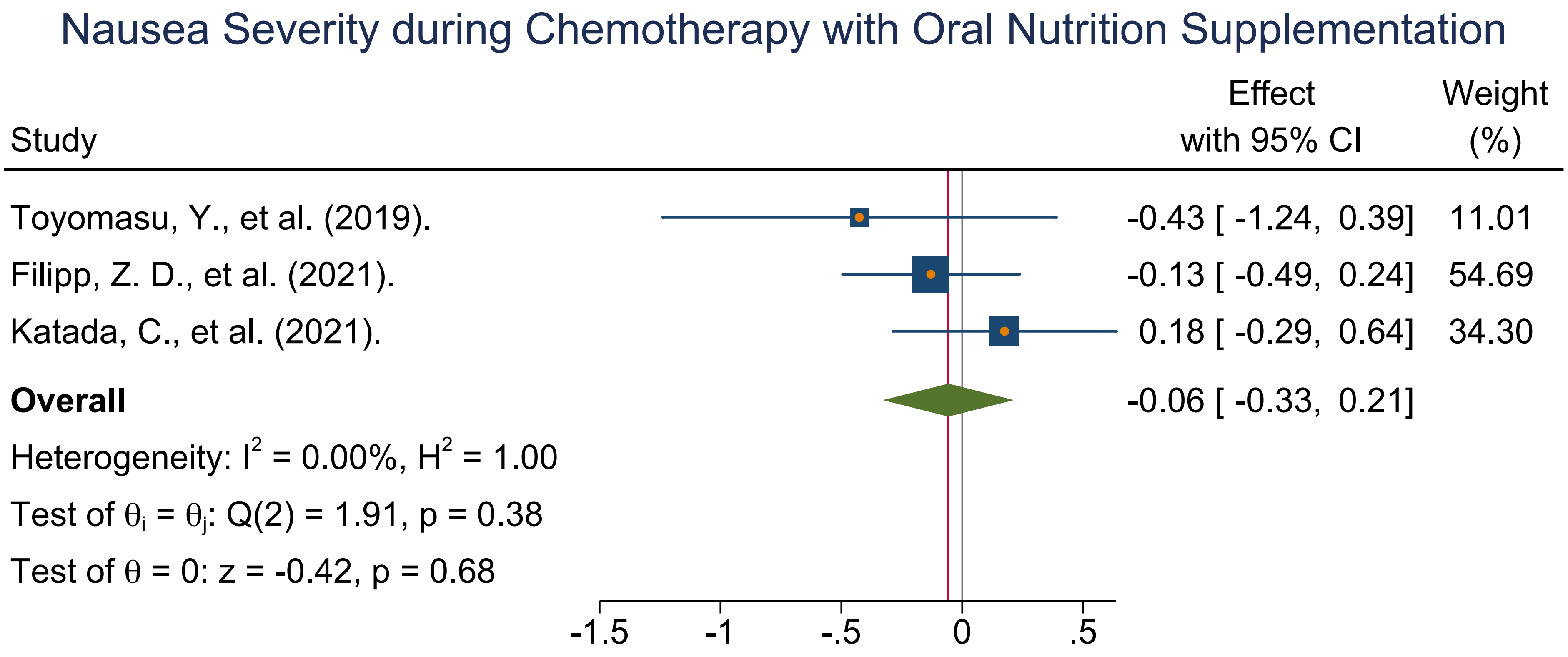
Nausea Severity during Chemotherapy with Oral Nutrition Supplementation
Study
Effect with 95% CI
Weight (%)
Toyomasu, Y., et al. (2019). -0.43 [-1.24, 0.39] 11.01
Filipp, Z. D., et al. (2021). -0.13 [-0.49, 0.24] 54.69
Katada, C., et al. (2021). 0.18 [-0.29, 0.64] 34.30
Overall -0.06 [-0.33, 0.21]
Heterogeneity: I2 = 0.00%, H2 = 1.00
Test of θi = θj: Q(2) = 1.91, p = 0.38
Test of θ = 0: z = -0.42, p = 0.68
-1.5
-1
-.5
0
.5

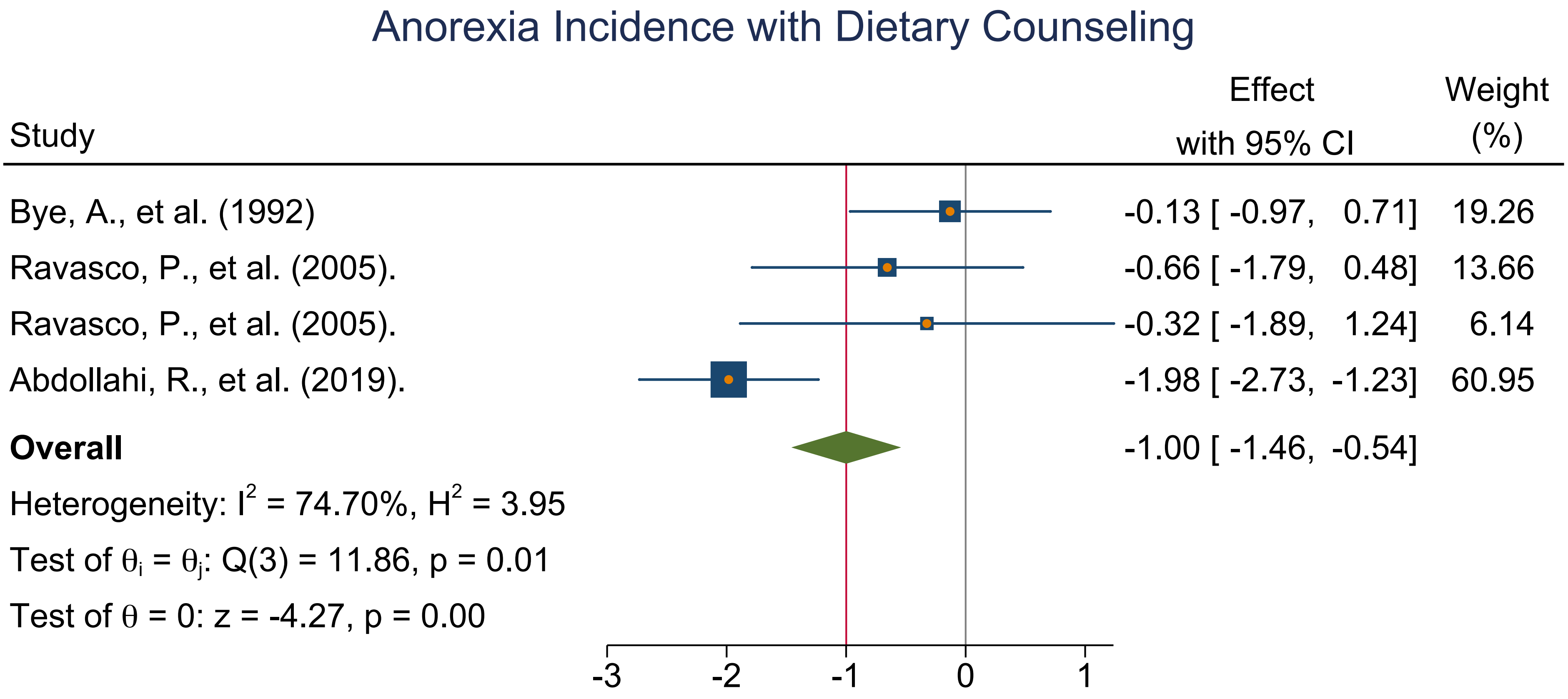
Anorexia Incidence with Dietary Counseling
Study
Effect with 95% CI
Weight (%)
Bye, A., et al. (1992) -0.13 [-0.97, 0.71] 19.26
Ravasco, P., et al. (2005). -0.66 [-1.79, 0.48] 13.66
Ravasco, P., et al. (2005). -0.32 [-1.89, 1.24] 6.14
Abdollahi, R., et al. (2019). -1.98 [-2.73, -1.23] 60.95
Overall -1.00 [-1.46, -0.54]
Heterogeneity: I^2 = 74.70%, H^2 = 3.95
Test of $\theta_i = \theta_j$: Q(3) = 11.86, p = 0.01
Test of θ = 0: z = -4.27, p = 0.00
-3
-2
-1
0
1

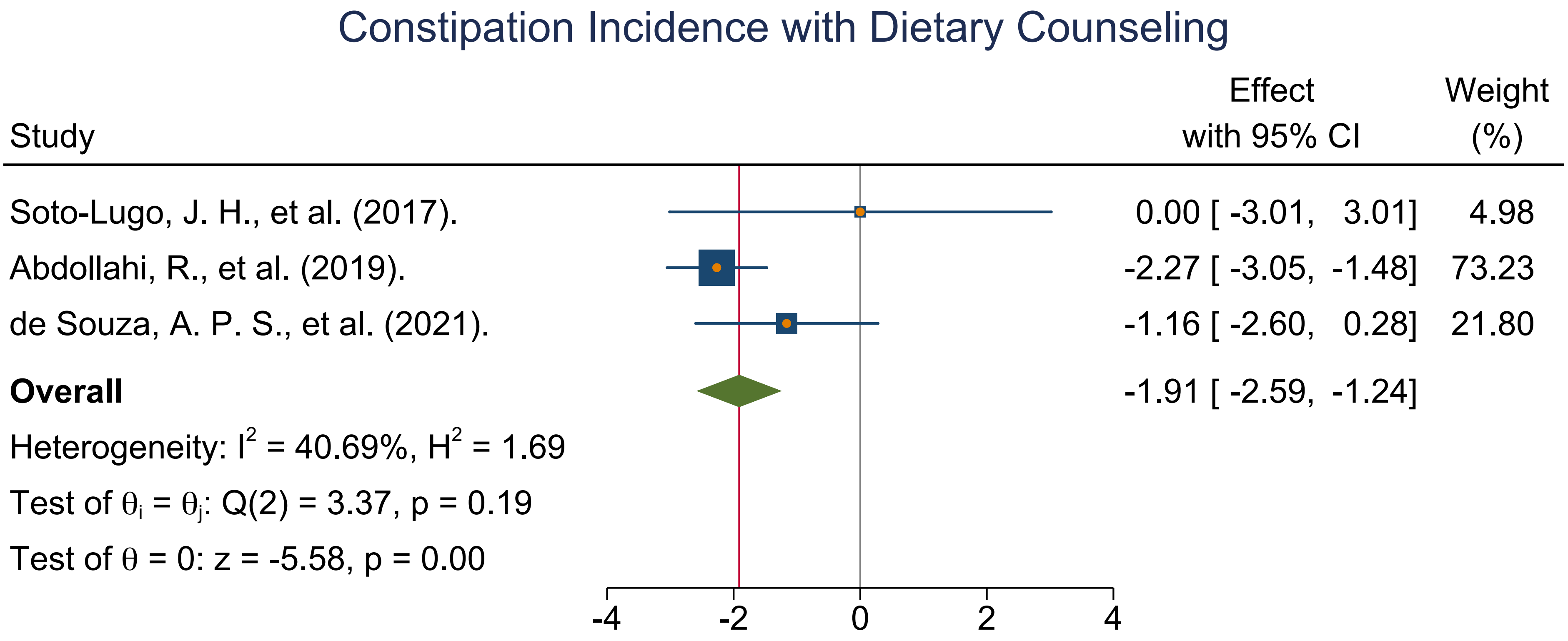

Constipation Incidence with Dietary Counseling
Study
Effect with 95% CI
Weight (%)
Soto-Lugo, J. H., et al. (2017). 0.00 [-3.01, 3.01] 4.98
Abdollahi, R., et al. (2019). -2.27 [-3.05, -1.48] 73.23
de Souza, A. P. S., et al. (2021). -1.16 [-2.60, 0.28] 21.80
Overall -1.91 [-2.59, -1.24]
Heterogeneity: I^2 = 40.69%, H^2 = 1.69
Test of $\theta_i = \theta_j$: Q(2) = 3.37, p = 0.19
Test of θ = 0: z = -5.58, p = 0.00
-4
-2
0
2
4

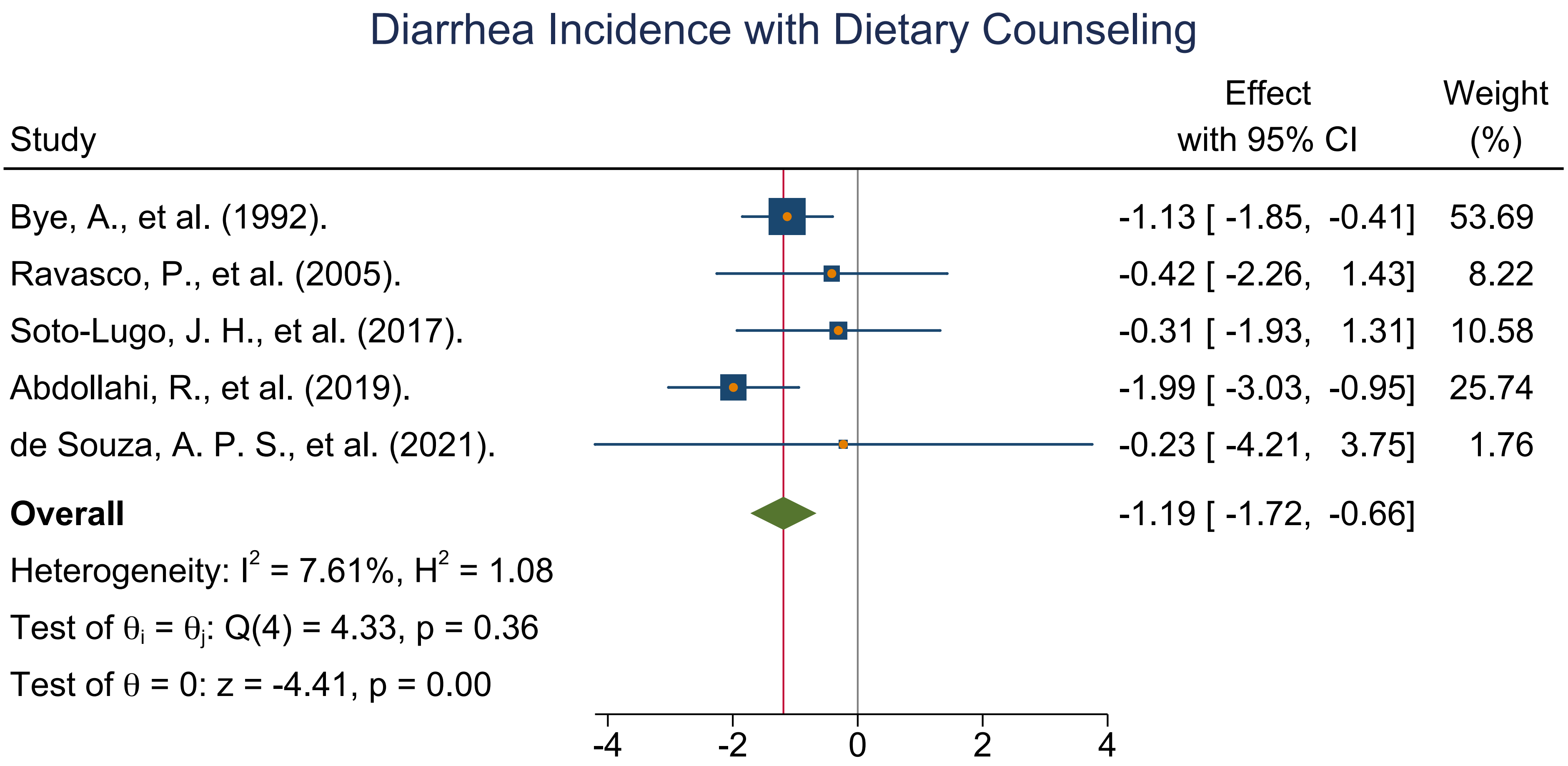
Diarrhea Incidence with Dietary Counseling
Study
Effect with 95% CI
Weight (%)
Bye, A., et al. (1992).
-1.13 [-1.85, -0.41]
53.69
Ravasco, P., et al. (2005).
-0.42 [-2.26, 1.43]
8.22
Soto-Lugo, J. H., et al. (2017).
-0.31 [-1.93, 1.31]
10.58
Abdollahi, R., et al. (2019).
-1.99 [-3.03, -0.95]
25.74
de Souza, A. P. S., et al. (2021).
-0.23 [-4.21, 3.75]
1.76
Overall
-1.19 [-1.72, -0.66]
Heterogeneity: I^2 = 7.61%, H^2 = 1.08
Test of $\theta_i = \theta_j$: Q(4) = 4.33, p = 0.36
Test of θ = 0: z = -4.41, p = 0.00
-4
-2
0
2
4

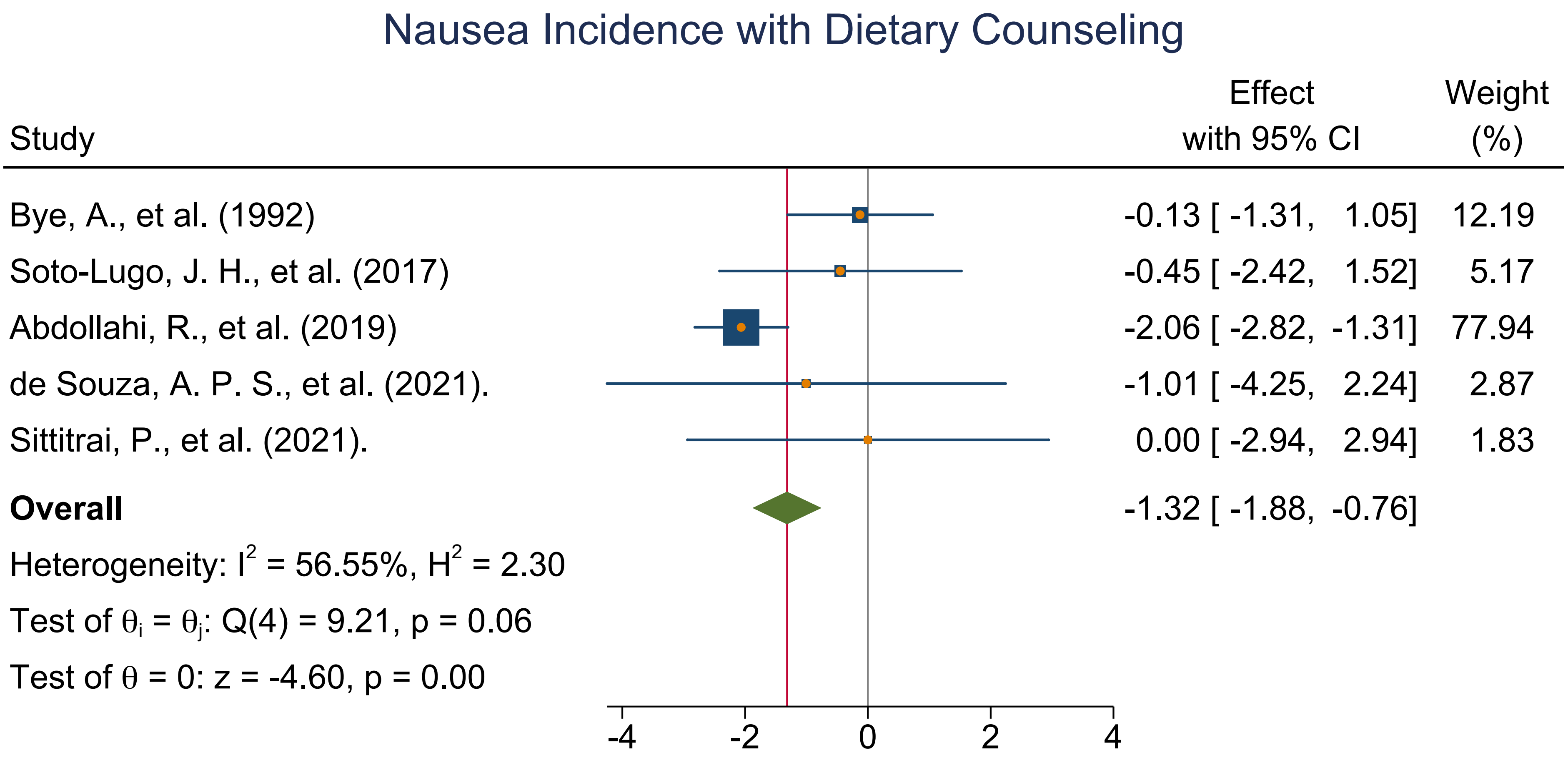
Nausea Incidence with Dietary Counseling
Study
Effect with 95% CI
Weight (%)
Bye, A., et al. (1992) -0.13 [-1.31, 1.05] 12.19
Soto-Lugo, J. H., et al. (2017) -0.45 [-2.42, 1.52] 5.17
Abdollahi, R., et al. (2019) -2.06 [-2.82, -1.31] 77.94
de Souza, A. P. S., et al. (2021). -1.01 [-4.25, 2.24] 2.87
Sittitrai, P., et al. (2021). 0.00 [-2.94, 2.94] 1.83
Overall -1.32 [-1.88, -0.76]
Heterogeneity: I² = 56.55%, H² = 2.30
Test of θi = θj: Q(4) = 9.21, p = 0.06
Test of θ = 0: z = -4.60, p = 0.00
-4
-2
0
2
4

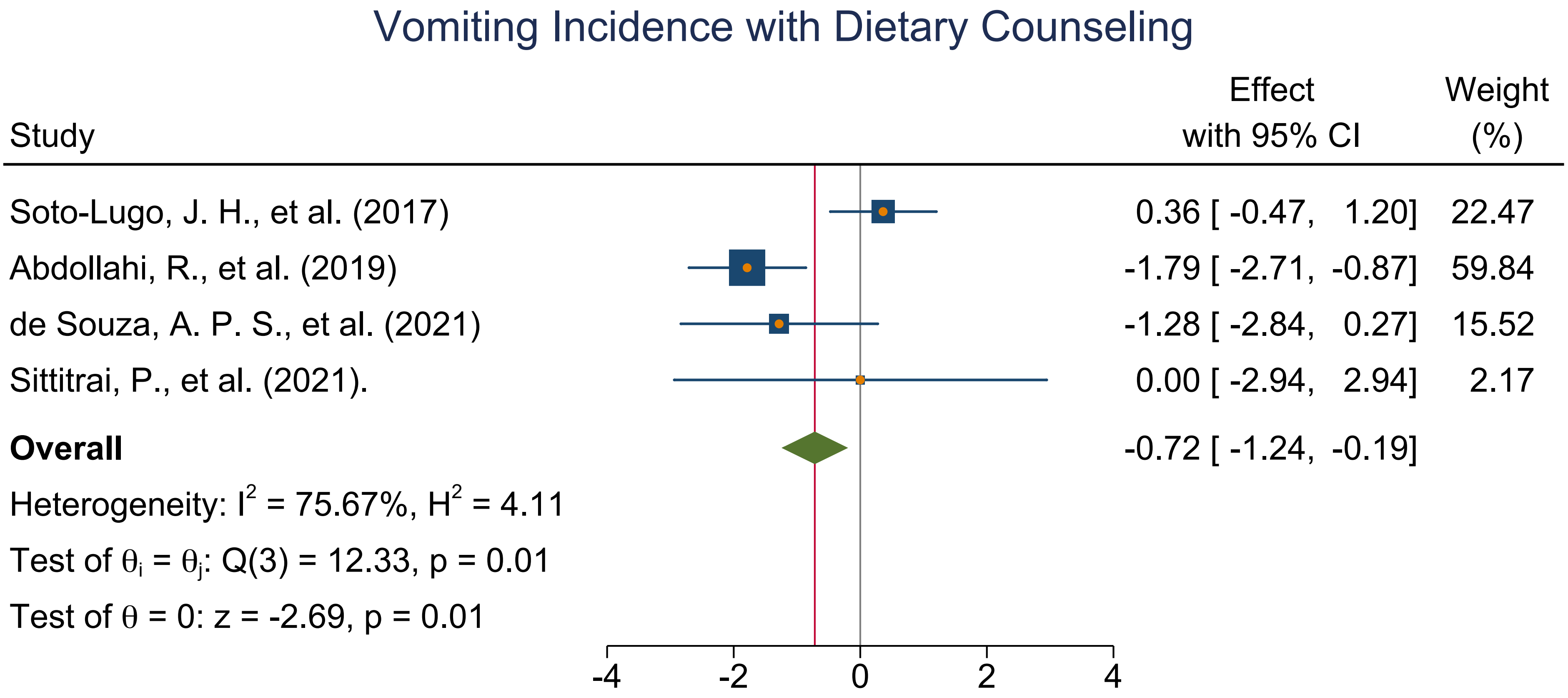

Vomiting Incidence with Dietary Counseling
Study
Effect with 95% CI
Weight (%)
Soto-Lugo, J. H., et al. (2017) 0.36 [-0.47, 1.20] 22.47
Abdollahi, R., et al. (2019) -1.79 [-2.71, -0.87] 59.84
de Souza, A. P. S., et al. (2021) -1.28 [-2.84, 0.27] 15.52
Sittitrai, P., et al. (2021). 0.00 [-2.94, 2.94] 2.17
Overall -0.72 [-1.24, -0.19]
Heterogeneity: I2 = 75.67%, H2 = 4.11
Test of θi = θj: Q(3) = 12.33, p = 0.01
Test of θ = 0: z = -2.69, p = 0.01
-4
-2
0
2
4

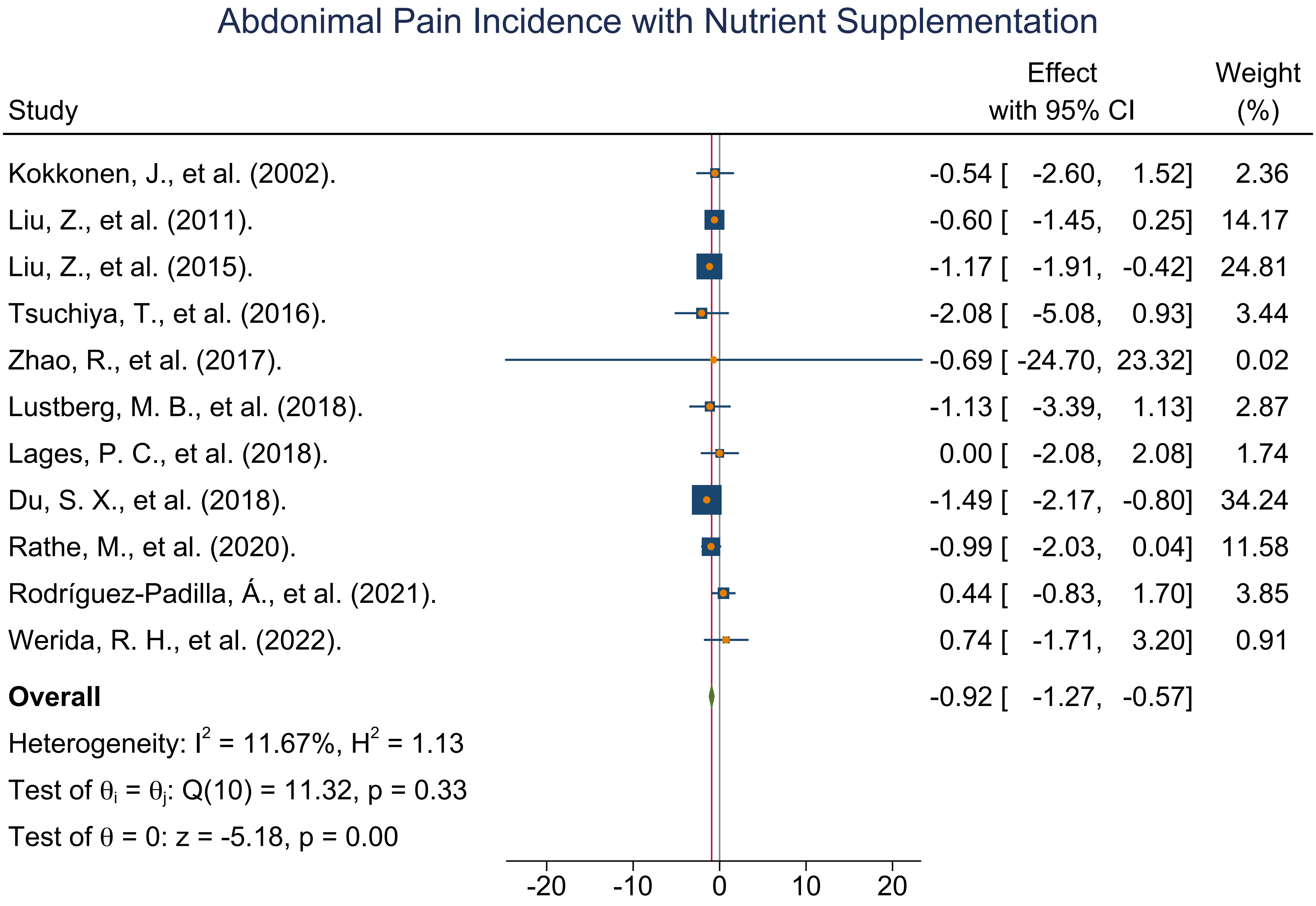
Abdonimal Pain Incidence with Nutrient Supplementation
Study
Effect with 95% CI
Weight (%)
Kokkonen, J., et al. (2002). -0.54 [-2.60, 1.52] 2.36
Liu, Z., et al. (2011). -0.60 [-1.45, 0.25] 14.17
Liu, Z., et al. (2015). -1.17 [-1.91, -0.42] 24.81
Tsuchiya, T., et al. (2016). -2.08 [-5.08, 0.93] 3.44
Zhao, R., et al. (2017). -0.69 [-24.70, 23.32] 0.02
Lustberg, M. B., et al. (2018). -1.13 [-3.39, 1.13] 2.87
Lages, P. C., et al. (2018). 0.00 [-2.08, 2.08] 1.74
Du, S. X., et al. (2018). -1.49 [-2.17, -0.80] 34.24
Rathe, M., et al. (2020). -0.99 [-2.03, 0.04] 11.58
Rodríguez-Padilla, Á., et al. (2021). 0.44 [-0.83, 1.70] 3.85
Werida, R. H., et al. (2022). 0.74 [-1.71, 3.20] 0.91
Overall -0.92 [-1.27, -0.57]
Heterogeneity: I^2 = 11.67%, H^2 = 1.13
Test of $\theta_i = \theta_j$: Q(10) = 11.32, p = 0.33
Test of θ = 0: z = -5.18, p = 0.00
-20
-10
0
10
20

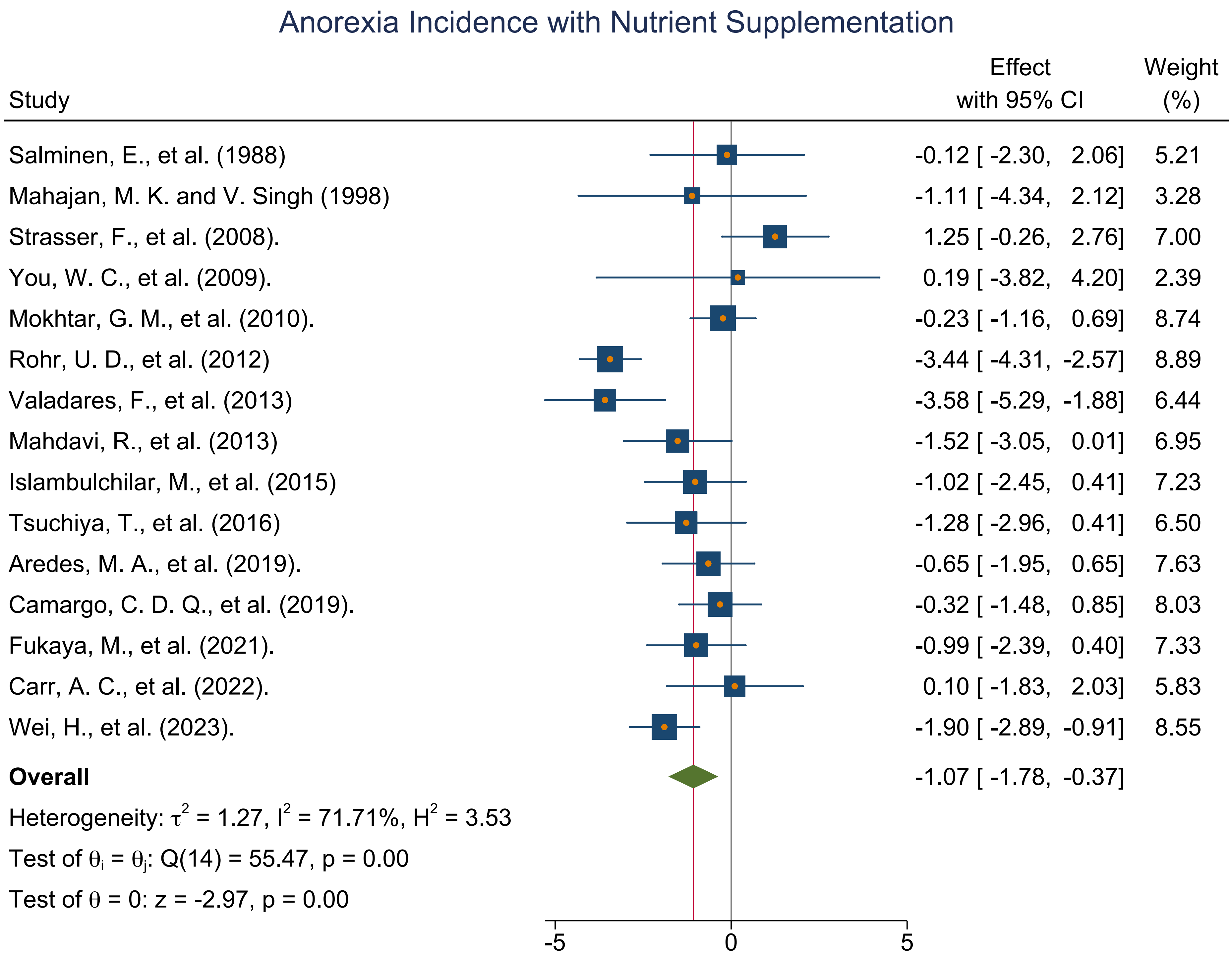
Anorexia Incidence with Nutrient Supplementation
Study
Effect with 95% CI
Weight (%)
Salminen, E., et al. (1988) -0.12 [-2.30, 2.06] 5.21
Mahajan, M. K. and V. Singh (1998) -1.11 [-4.34, 2.12] 3.28
Strasser, F., et al. (2008). 1.25 [-0.26, 2.76] 7.00
You, W. C., et al. (2009). 0.19 [-3.82, 4.20] 2.39
Mokhtar, G. M., et al. (2010). -0.23 [-1.16, 0.69] 8.74
Rohr, U. D., et al. (2012) -3.44 [-4.31, -2.57] 8.89
Valadares, F., et al. (2013) -3.58 [-5.29, -1.88] 6.44
Mahdavi, R., et al. (2013) -1.52 [-3.05, 0.01] 6.95
Islambulchilar, M., et al. (2015) -1.02 [-2.45, 0.41] 7.23
Tsuchiya, T., et al. (2016) -1.28 [-2.96, 0.41] 6.50
Aredes, M. A., et al. (2019). -0.65 [-1.95, 0.65] 7.63
Camargo, C. D. Q., et al. (2019). -0.32 [-1.48, 0.85] 8.03
Fukaya, M., et al. (2021). -0.99 [-2.39, 0.40] 7.33
Carr, A. C., et al. (2022). 0.10 [-1.83, 2.03] 5.83
Wei, H., et al. (2023). -1.90 [-2.89, -0.91] 8.55
Overall -1.07 [-1.78, -0.37]
Heterogeneity: τ² = 1.27, I² = 71.71%, H² = 3.53
Test of θi = θj: Q(14) = 55.47, p = 0.00
Test of θ = 0: z = -2.97, p = 0.00
-5
0
5

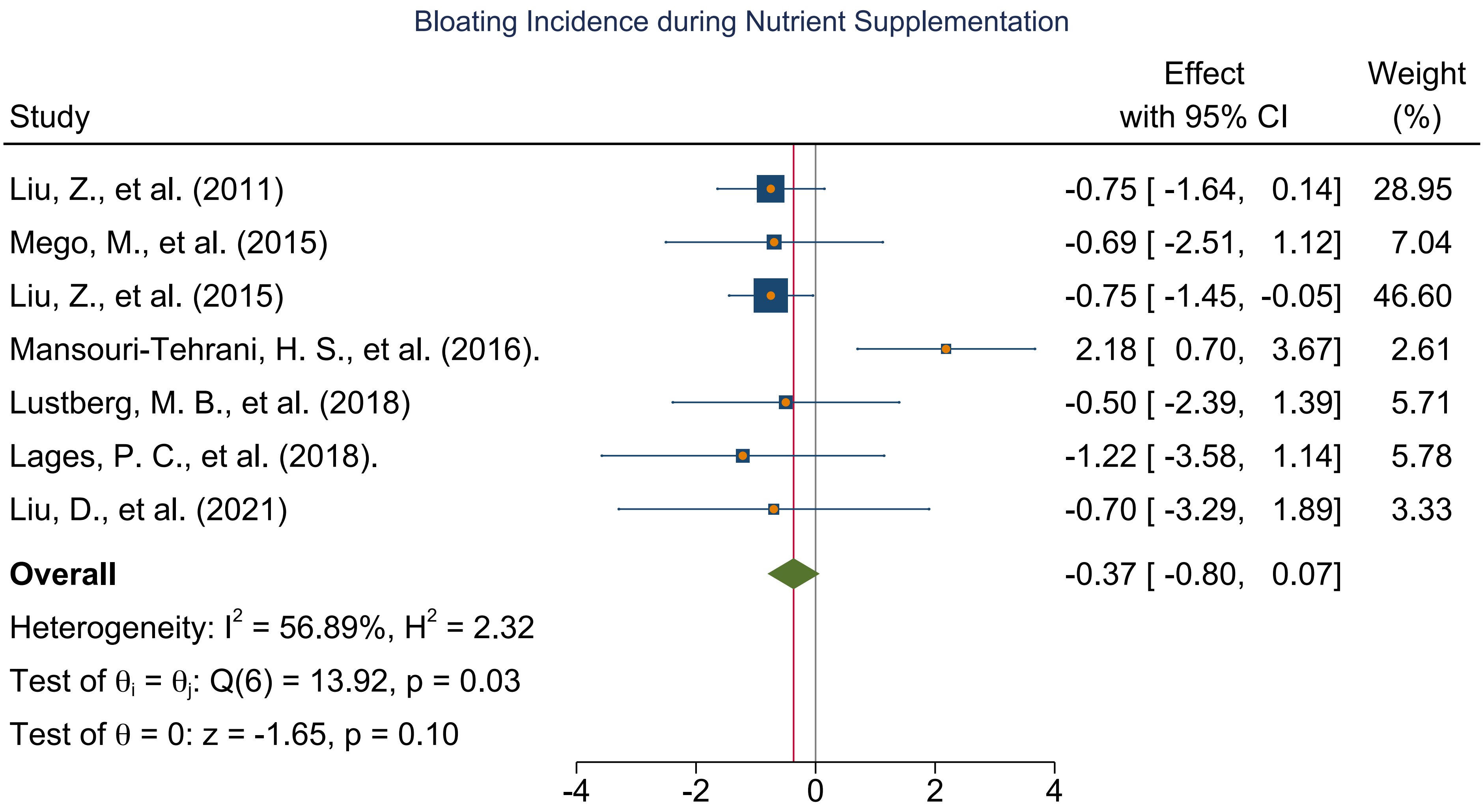
Bloating Incidence during Nutrient Supplementation
Study
Effect with 95% CI
Weight (%)
Liu, Z., et al. (2011) -0.75 [-1.64, 0.14] 28.95
Mego, M., et al. (2015) -0.69 [-2.51, 1.12] 7.04
Liu, Z., et al. (2015) -0.75 [-1.45, -0.05] 46.60
Mansouri-Tehrani, H. S., et al. (2016). 2.18 [0.70, 3.67] 2.61
Lustberg, M. B., et al. (2018) -0.50 [-2.39, 1.39] 5.71
Lages, P. C., et al. (2018). -1.22 [-3.58, 1.14] 5.78
Liu, D., et al. (2021) -0.70 [-3.29, 1.89] 3.33
Overall -0.37 [-0.80, 0.07]
Heterogeneity: I^2 = 56.89%, H^2 = 2.32
Test of $\theta_i = \theta_j$: Q(6) = 13.92, p = 0.03
Test of θ = 0: z = -1.65, p = 0.10
-4
-2
0
2
4

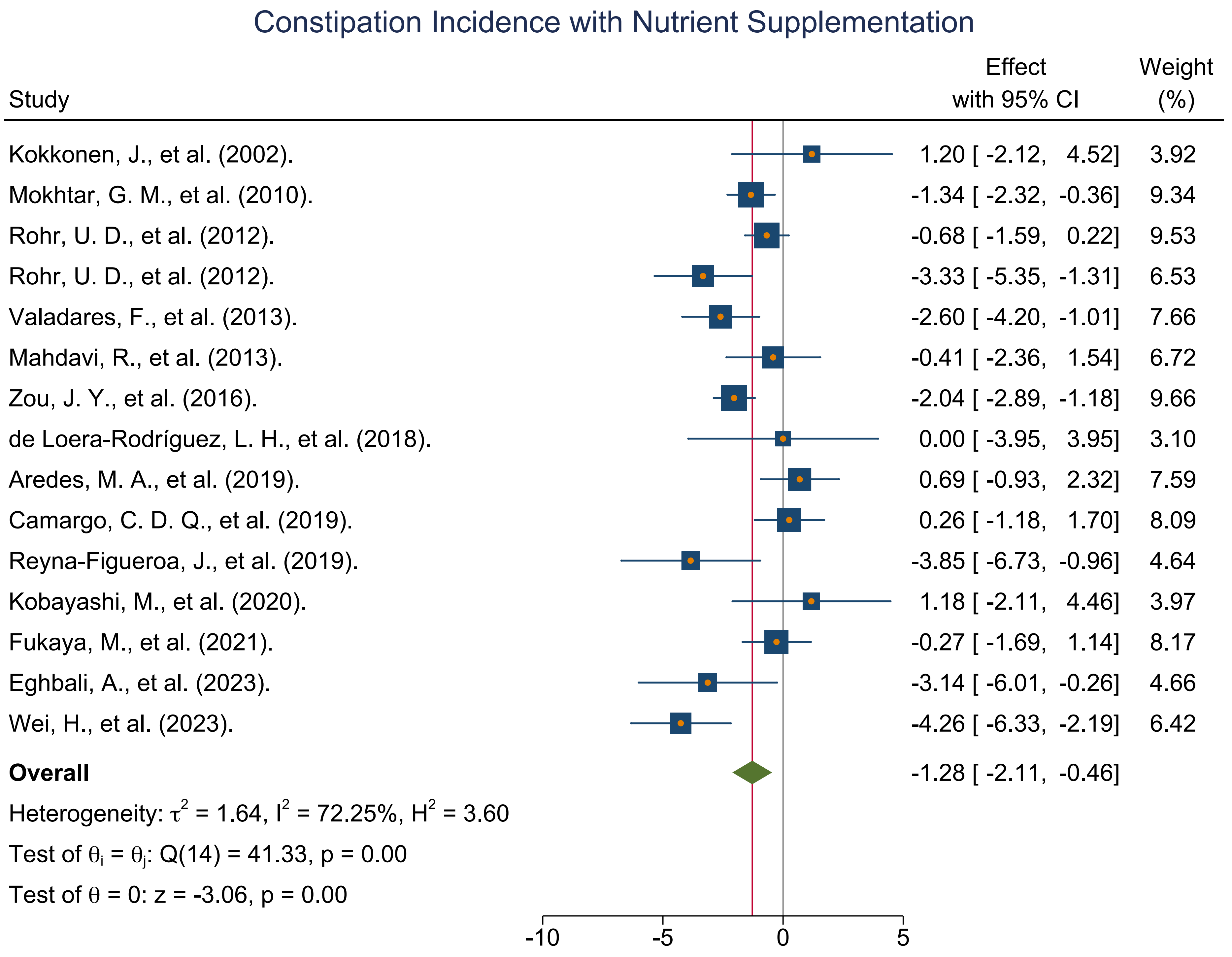
Constipation Incidence with Nutrient Supplementation
Study
Effect with 95% CI
Weight (%)
Kokkonen, J., et al. (2002). 1.20 [-2.12, 4.52] 3.92
Mokhtar, G. M., et al. (2010). -1.34 [-2.32, -0.36] 9.34
Rohr, U. D., et al. (2012). -0.68 [-1.59, 0.22] 9.53
Rohr, U. D., et al. (2012). -3.33 [-5.35, -1.31] 6.53
Valadares, F., et al. (2013). -2.60 [-4.20, -1.01] 7.66
Mahdavi, R., et al. (2013). -0.41 [-2.36, 1.54] 6.72
Zou, J. Y., et al. (2016). -2.04 [-2.89, -1.18] 9.66
de Loera-Rodríguez, L. H., et al. (2018). 0.00 [-3.95, 3.95] 3.10
Aredes, M. A., et al. (2019). 0.69 [-0.93, 2.32] 7.59
Camargo, C. D. Q., et al. (2019). 0.26 [-1.18, 1.70] 8.09
Reyna-Figueroa, J., et al. (2019). -3.85 [-6.73, -0.96] 4.64
Kobayashi, M., et al. (2020). 1.18 [-2.11, 4.46] 3.97
Fukaya, M., et al. (2021). -0.27 [-1.69, 1.14] 8.17
Eghbali, A., et al. (2023). -3.14 [-6.01, -0.26] 4.66
Wei, H., et al. (2023). -4.26 [-6.33, -2.19] 6.42
Overall -1.28 [-2.11, -0.46]
Heterogeneity: τ^2 = 1.64, I^2 = 72.25%, H^2 = 3.60
Test of $\theta_i = \theta_j$: Q(14) = 41.33, p = 0.00
Test of θ = 0: z = -3.06, p = 0.00
-10
-5
0
5

Diarrhea Incidence with Nutrient Supplementation

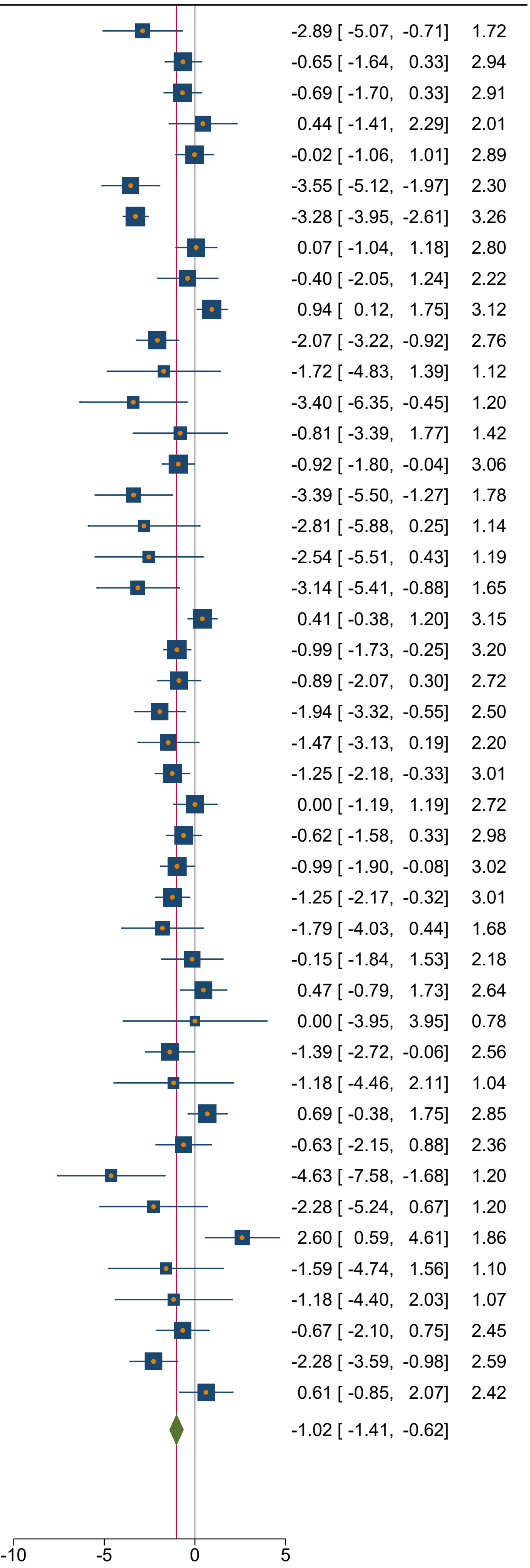

Study	Effect with 95% CI	Weight (%)
Salminen, E., et al. (1988).	-2.89 [-5.07, -0.71]	1.72
Bozzetti et al. (1997)	-0.65 [-1.64, 0.33]	2.94
Daniele, B., et al. (2001).	-0.69 [-1.70, 0.33]	2.91
Kokkonen, J., et al. (2002).	0.44 [-1.41, 2.29]	2.01
Li, Y. (2006)	-0.02 [-1.06, 1.01]	2.89
Mücke, R., et al. (2006).	-3.55 [-5.12, -1.97]	2.30
Delia, P., et al. (2007).	-3.28 [-3.95, -2.61]	3.26
de Luis, D. A., et al. (2007).	0.07 [-1.04, 1.18]	2.80
Strasser, F., et al. (2008).	-0.40 [-2.05, 1.24]	2.22
Giralt, J., et al. (2008).	0.94 [0.12, 1.75]	3.12
Chitapanarux, I., et al. (2010).	-2.07 [-3.22, -0.92]	2.76
Li, G. H., et al. (2010).	-1.72 [-4.83, 1.39]	1.12
Li, G. H., et al. (2010).	-3.40 [-6.35, -0.45]	1.20
Ikeguchi, M., et al. (2011).	-0.81 [-3.39, 1.77]	1.42
Liu, Z., et al. (2011).	-0.92 [-1.80, -0.04]	3.06
Rohr, U. D., et al. (2012).	-3.39 [-5.50, -1.27]	1.78
Kucuktulu, E., et al. (2013).	-2.81 [-5.88, 0.25]	1.14
Valadares, F., et al. (2013).	-2.54 [-5.51, 0.43]	1.19
Mahdavi, R., et al. (2013).	-3.14 [-5.41, -0.88]	1.65
Demers, M., et al. (2014).	0.41 [-0.38, 1.20]	3.15
Liu, Z., et al. (2015).	-0.99 [-1.73, -0.25]	3.20
Mego, M., et al. (2015).	-0.89 [-2.07, 0.30]	2.72
Tsuchiya, T., et al. (2016).	-1.94 [-3.32, -0.55]	2.50
Motoori, M., et al. (2017).	-1.47 [-3.13, 0.19]	2.20
Zhao, R., et al. (2017).	-1.25 [-2.18, -0.33]	3.01
Lustberg, M. B., et al. (2018).	0.00 [-1.19, 1.19]	2.72
de Loera-Rodríguez, L. H., et al. (2018).	-0.62 [-1.58, 0.33]	2.98
Xie, H., et al. (2018).	-0.99 [-1.90, -0.08]	3.02
Du, S. X., et al. (2018).	-1.25 [-2.17, -0.32]	3.01
de la Rosa Oliva, F., et al. (2019).	-1.79 [-4.03, 0.44]	1.68
Aredes, M. A., et al. (2019).	-0.15 [-1.84, 1.53]	2.18
Camargo, C. D. Q., et al. (2019).	0.47 [-0.79, 1.73]	2.64
Reyna-Figueroa (2019)	0.00 [-3.95, 3.95]	0.78
Tian, Y., et al. (2019).	-1.39 [-2.72, -0.06]	2.56
Kobayashi, M., et al. (2020).	-1.18 [-4.46, 2.11]	1.04
Rathe, M., et al. (2020).	0.69 [-0.38, 1.75]	2.85
Fukaya, M., et al. (2021).	-0.63 [-2.15, 0.88]	2.36
Aziz, L. M. A., et al. (2021).	-4.63 [-7.58, -1.68]	1.20
Liu D. et al. (2021)	-2.28 [-5.24, 0.67]	1.20
Rosli, D., et al. (2021).	2.60 [0.59, 4.61]	1.86
Carr et al. (2022)	-1.59 [-4.74, 1.56]	1.10
Eghbali, A., et al. (2023).	-1.18 [-4.40, 2.03]	1.07
Hussein, R. M., et al. (2023).	-0.67 [-2.10, 0.75]	2.45
Wei, H., et al. (2023).	-2.28 [-3.59, -0.98]	2.59
Yanagimoto, H., et al. (2023).	0.61 [-0.85, 2.07]	2.42
**Overall**	-1.02 [-1.41, -0.62]	

Heterogeneity: $\tau^2 = 1.13$, $I^2 = 72.44\%$, $H^2 = 3.63$

Test of $\theta_i = \theta_j$: $Q(44) = 168.20$, $p = 0.00$

Test of $\theta = 0$: $z = -5.03$, $p = 0.00$

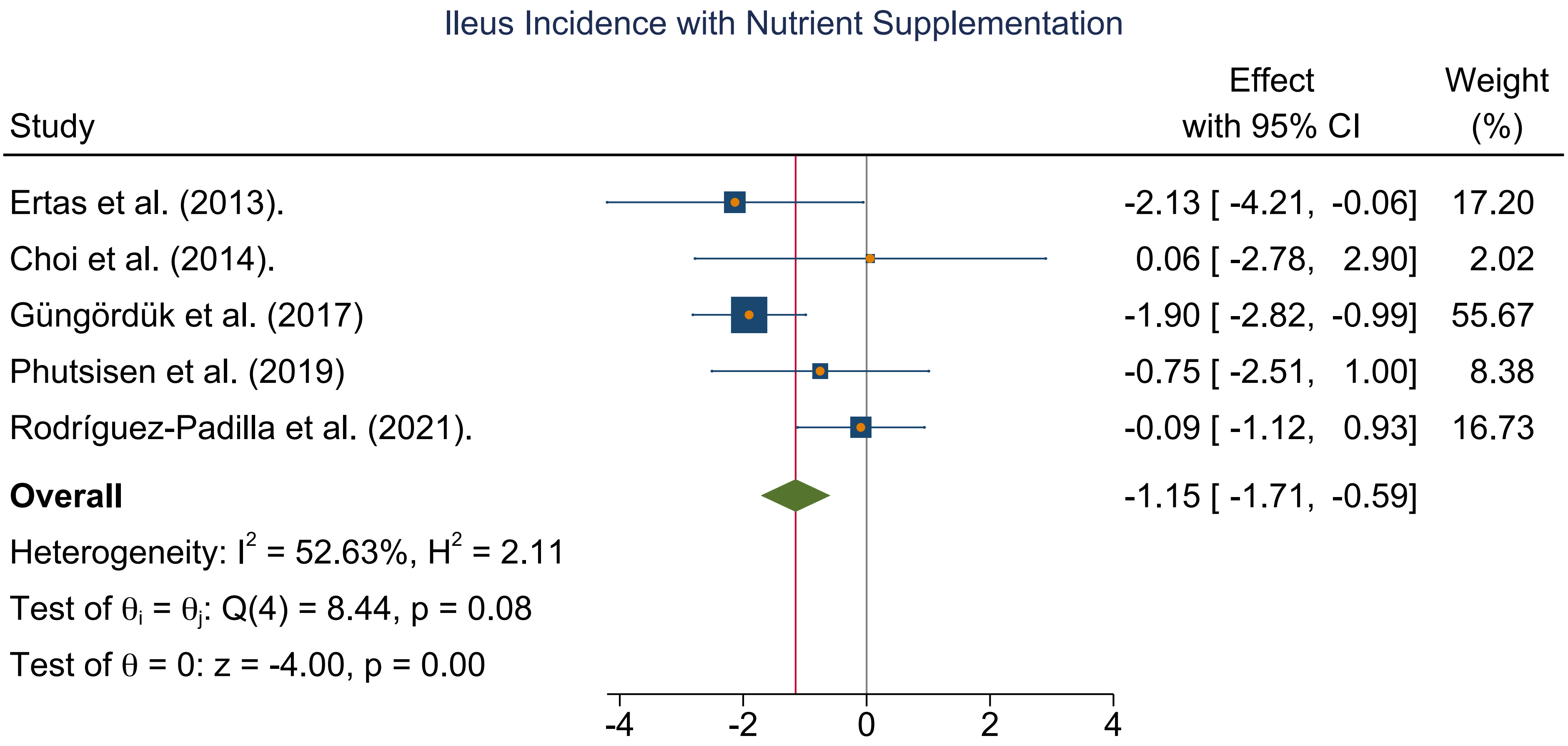
Ileus Incidence with Nutrient Supplementation
Study
Effect with 95% CI
Weight (%)
Ertas et al. (2013). -2.13 [-4.21, -0.06] 17.20
Choi et al. (2014). 0.06 [-2.78, 2.90] 2.02
Güngördük et al. (2017) -1.90 [-2.82, -0.99] 55.67
Phutsisen et al. (2019) -0.75 [-2.51, 1.00] 8.38
Rodríguez-Padilla et al. (2021). -0.09 [-1.12, 0.93] 16.73
Overall -1.15 [-1.71, -0.59]
Heterogeneity: I^2 = 52.63%, H^2 = 2.11
Test of $\theta_i = \theta_j$: Q(4) = 8.44, p = 0.08
Test of θ = 0: z = -4.00, p = 0.00
-4
-2
0
2
4

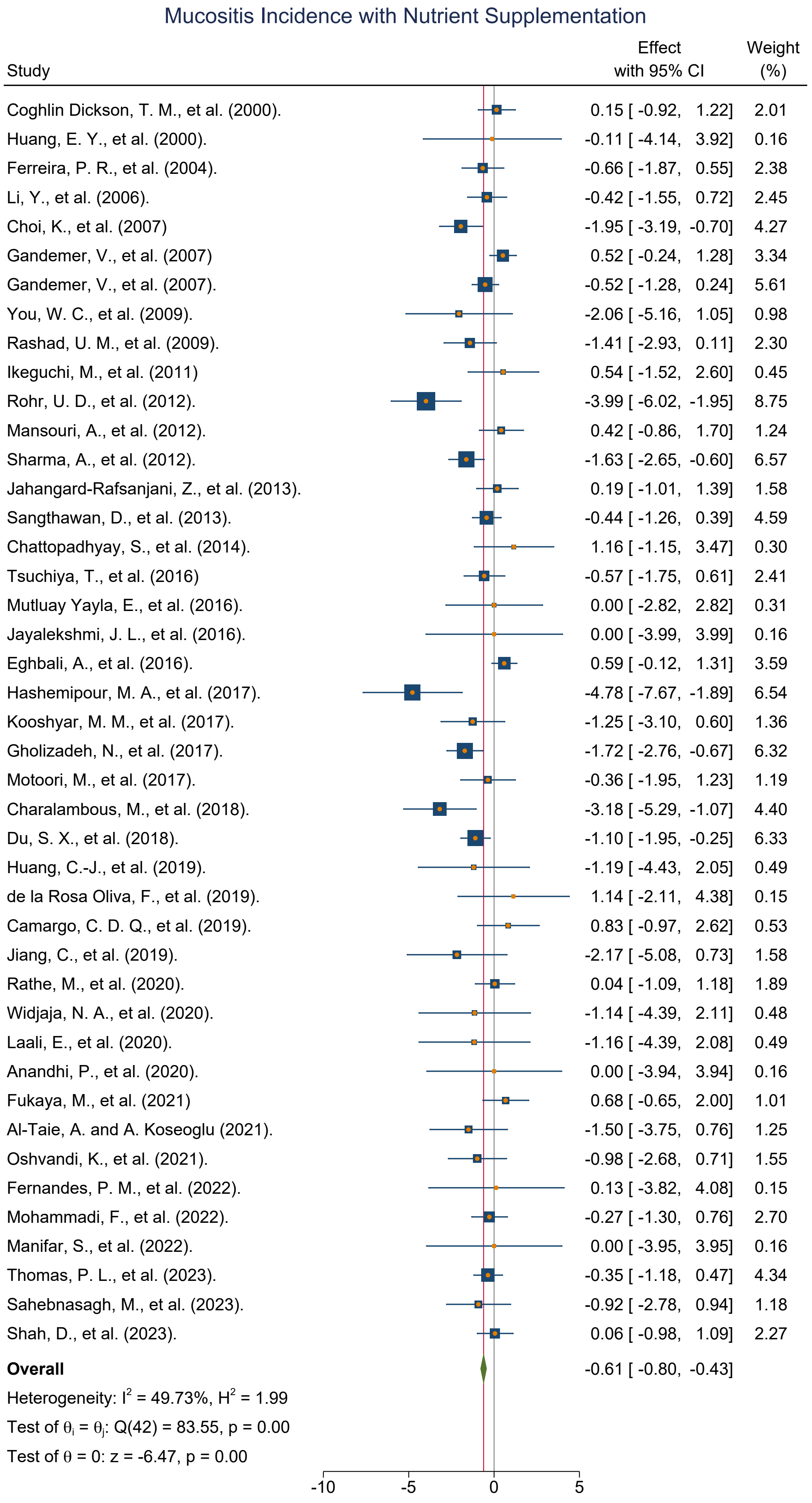
Mucositis Incidence with Nutrient Supplementation
Study
Effect with 95% CI
Weight (%)
Coghlin Dickson, T. M., et al. (2000). 0.15 [-0.92, 1.22] 2.01
Huang, E. Y., et al. (2000). -0.11 [-4.14, 3.92] 0.16
Ferreira, P. R., et al. (2004). -0.66 [-1.87, 0.55] 2.38
Li, Y., et al. (2006). -0.42 [-1.55, 0.72] 2.45
Choi, K., et al. (2007) -1.95 [-3.19, -0.70] 4.27
Gandemer, V., et al. (2007) 0.52 [-0.24, 1.28] 3.34
Gandemer, V., et al. (2007). -0.52 [-1.28, 0.24] 5.61
You, W. C., et al. (2009). -2.06 [-5.16, 1.05] 0.98
Rashad, U. M., et al. (2009). -1.41 [-2.93, 0.11] 2.30
Ikeguchi, M., et al. (2011) 0.54 [-1.52, 2.60] 0.45
Rohr, U. D., et al. (2012). -3.99 [-6.02, -1.95] 8.75
Mansouri, A., et al. (2012). 0.42 [-0.86, 1.70] 1.24
Sharma, A., et al. (2012). -1.63 [-2.65, -0.60] 6.57
Jahangard-Rafsanjani, Z., et al. (2013). 0.19 [-1.01, 1.39] 1.58
Sangthawan, D., et al. (2013). -0.44 [-1.26, 0.39] 4.59
Chattopadhyay, S., et al. (2014). 1.16 [-1.15, 3.47] 0.30
Tsuchiya, T., et al. (2016) -0.57 [-1.75, 0.61] 2.41
Mutluay Yayla, E., et al. (2016). 0.00 [-2.82, 2.82] 0.31
Jayalekshmi, J. L., et al. (2016). 0.00 [-3.99, 3.99] 0.16
Eghbali, A., et al. (2016). 0.59 [-0.12, 1.31] 3.59
Hashemipour, M. A., et al. (2017). -4.78 [-7.67, -1.89] 6.54
Kooshyar, M. M., et al. (2017). -1.25 [-3.10, 0.60] 1.36
Gholizadeh, N., et al. (2017). -1.72 [-2.76, -0.67] 6.32
Motoori, M., et al. (2017). -0.36 [-1.95, 1.23] 1.19
Charalambous, M., et al. (2018). -3.18 [-5.29, -1.07] 4.40
Du, S. X., et al. (2018). -1.10 [-1.95, -0.25] 6.33
Huang, C.-J., et al. (2019). -1.19 [-4.43, 2.05] 0.49
de la Rosa Oliva, F., et al. (2019). 1.14 [-2.11, 4.38] 0.15
Camargo, C. D. Q., et al. (2019). 0.83 [-0.97, 2.62] 0.53
Jiang, C., et al. (2019). -2.17 [-5.08, 0.73] 1.58
Rathe, M., et al. (2020). 0.04 [-1.09, 1.18] 1.89
Widjaja, N. A., et al. (2020). -1.14 [-4.39, 2.11] 0.48
Laali, E., et al. (2020). -1.16 [-4.39, 2.08] 0.49
Anandhi, P., et al. (2020). 0.00 [-3.94, 3.94] 0.16
Fukaya, M., et al. (2021) 0.68 [-0.65, 2.00] 1.01
Al-Taie, A. and A. Koseoglu (2021). -1.50 [-3.75, 0.76] 1.25
Oshvandi, K., et al. (2021). -0.98 [-2.68, 0.71] 1.55
Fernandes, P. M., et al. (2022). 0.13 [-3.82, 4.08] 0.15
Mohammadi, F., et al. (2022). -0.27 [-1.30, 0.76] 2.70
Manifar, S., et al. (2022). 0.00 [-3.95, 3.95] 0.16
Thomas, P. L., et al. (2023). -0.35 [-1.18, 0.47] 4.34
Sahebnasagh, M., et al. (2023). -0.92 [-2.78, 0.94] 1.18
Shah, D., et al. (2023). 0.06 [-0.98, 1.09] 2.27
Overall -0.61 [-0.80, -0.43]
Heterogeneity: I² = 49.73%, H² = 1.99
Test of θi = θj: Q(42) = 83.55, p = 0.00
Test of θ = 0: z = -6.47, p = 0.00
-10
-5
0
5

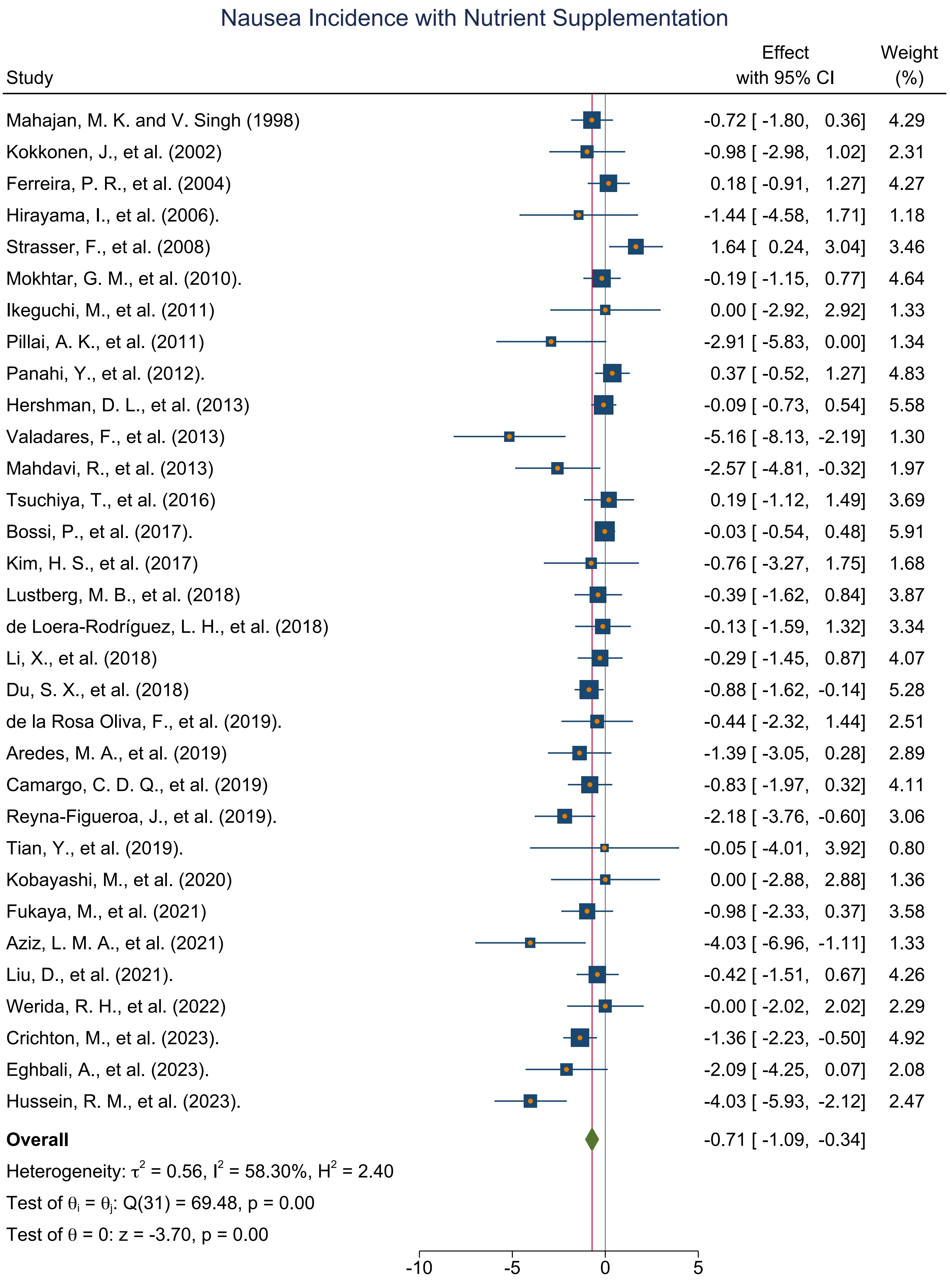

Nausea Incidence with Nutrient Supplementation
Study
Effect with 95% CI
Weight (%)
Mahajan, M. K. and V. Singh (1998) -0.72 [-1.80, 0.36] 4.29
Kokkonen, J., et al. (2002) -0.98 [-2.98, 1.02] 2.31
Ferreira, P. R., et al. (2004) 0.18 [-0.91, 1.27] 4.27
Hirayama, I., et al. (2006). -1.44 [-4.58, 1.71] 1.18
Strasser, F., et al. (2008) 1.64 [0.24, 3.04] 3.46
Mokhtar, G. M., et al. (2010). -0.19 [-1.15, 0.77] 4.64
Ikeguchi, M., et al. (2011) 0.00 [-2.92, 2.92] 1.33
Pillai, A. K., et al. (2011) -2.91 [-5.83, 0.00] 1.34
Panahi, Y., et al. (2012). 0.37 [-0.52, 1.27] 4.83
Hershman, D. L., et al. (2013) -0.09 [-0.73, 0.54] 5.58
Valadares, F., et al. (2013) -5.16 [-8.13, -2.19] 1.30
Mahdavi, R., et al. (2013) -2.57 [-4.81, -0.32] 1.97
Tsuchiya, T., et al. (2016) 0.19 [-1.12, 1.49] 3.69
Bossi, P., et al. (2017). -0.03 [-0.54, 0.48] 5.91
Kim, H. S., et al. (2017) -0.76 [-3.27, 1.75] 1.68
Lustberg, M. B., et al. (2018) -0.39 [-1.62, 0.84] 3.87
de Loera-Rodríguez, L. H., et al. (2018) -0.13 [-1.59, 1.32] 3.34
Li, X., et al. (2018) -0.29 [-1.45, 0.87] 4.07
Du, S. X., et al. (2018) -0.88 [-1.62, -0.14] 5.28
de la Rosa Oliva, F., et al. (2019). -0.44 [-2.32, 1.44] 2.51
Aredes, M. A., et al. (2019) -1.39 [-3.05, 0.28] 2.89
Camargo, C. D. Q., et al. (2019) -0.83 [-1.97, 0.32] 4.11
Reyna-Figueroa, J., et al. (2019). -2.18 [-3.76, -0.60] 3.06
Tian, Y., et al. (2019). -0.05 [-4.01, 3.92] 0.80
Kobayashi, M., et al. (2020) 0.00 [-2.88, 2.88] 1.36
Fukaya, M., et al. (2021) -0.98 [-2.33, 0.37] 3.58
Aziz, L. M. A., et al. (2021) -4.03 [-6.96, -1.11] 1.33
Liu, D., et al. (2021). -0.42 [-1.51, 0.67] 4.26
Werida, R. H., et al. (2022) -0.00 [-2.02, 2.02] 2.29
Crichton, M., et al. (2023). -1.36 [-2.23, -0.50] 4.92
Eghbali, A., et al. (2023). -2.09 [-4.25, 0.07] 2.08
Hussein, R. M., et al. (2023). -4.03 [-5.93, -2.12] 2.47
Overall -0.71 [-1.09, -0.34]
Heterogeneity: τ² = 0.56, I² = 58.30%, H² = 2.40
Test of θi = θj: Q(31) = 69.48, p = 0.00
Test of θ = 0: z = -3.70, p = 0.00
-10
-5
0
5

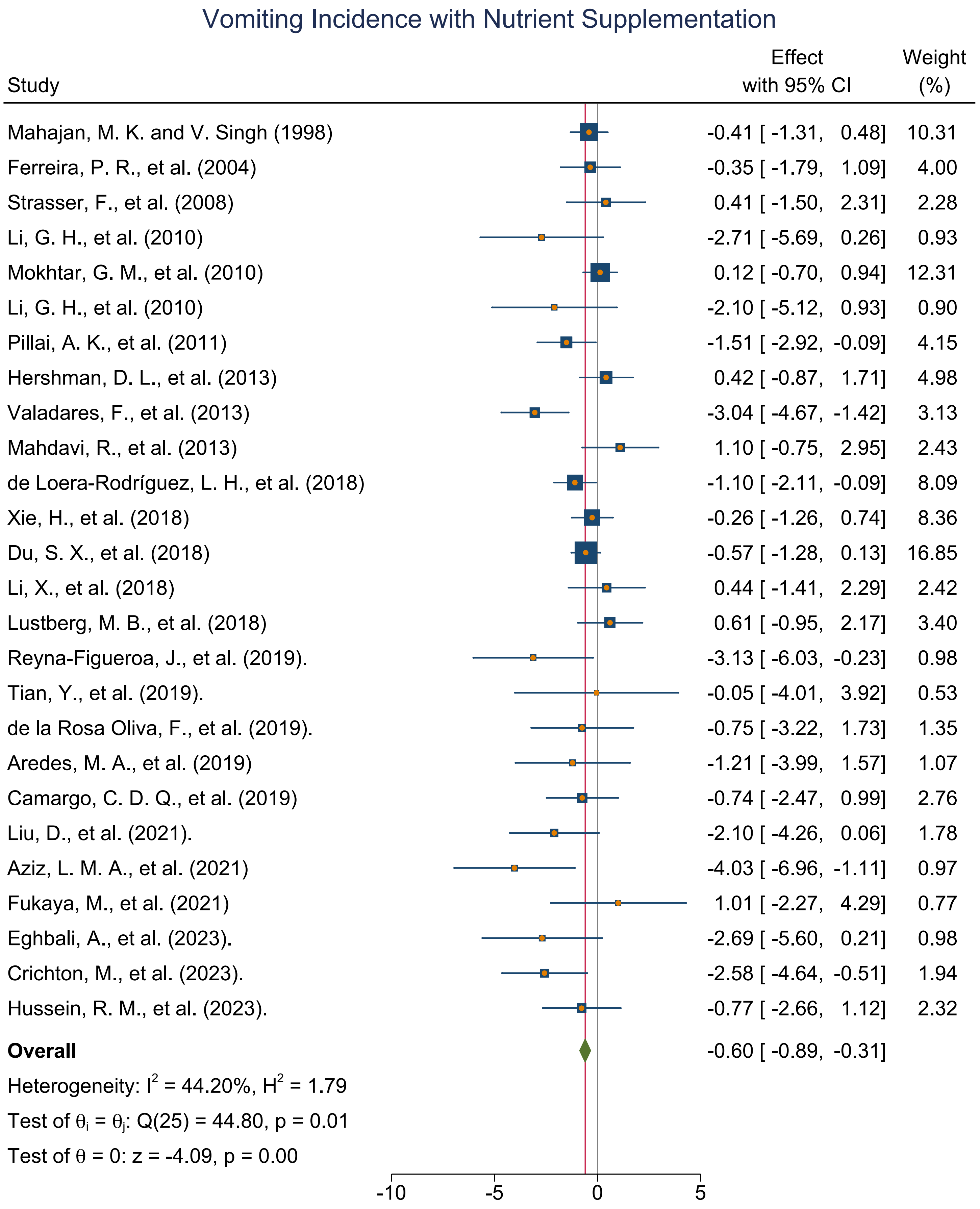
Vomiting Incidence with Nutrient Supplementation
Study
Effect with 95% CI
Weight (%)
Mahajan, M. K. and V. Singh (1998) -0.41 [-1.31, 0.48] 10.31
Ferreira, P. R., et al. (2004) -0.35 [-1.79, 1.09] 4.00
Strasser, F., et al. (2008) 0.41 [-1.50, 2.31] 2.28
Li, G. H., et al. (2010) -2.71 [-5.69, 0.26] 0.93
Mokhtar, G. M., et al. (2010) 0.12 [-0.70, 0.94] 12.31
Li, G. H., et al. (2010) -2.10 [-5.12, 0.93] 0.90
Pillai, A. K., et al. (2011) -1.51 [-2.92, -0.09] 4.15
Hershman, D. L., et al. (2013) 0.42 [-0.87, 1.71] 4.98
Valadares, F., et al. (2013) -3.04 [-4.67, -1.42] 3.13
Mahdavi, R., et al. (2013) 1.10 [-0.75, 2.95] 2.43
de Loera-Rodríguez, L. H., et al. (2018) -1.10 [-2.11, -0.09] 8.09
Xie, H., et al. (2018) -0.26 [-1.26, 0.74] 8.36
Du, S. X., et al. (2018) -0.57 [-1.28, 0.13] 16.85
Li, X., et al. (2018) 0.44 [-1.41, 2.29] 2.42
Lustberg, M. B., et al. (2018) 0.61 [-0.95, 2.17] 3.40
Reyna-Figueroa, J., et al. (2019). -3.13 [-6.03, -0.23] 0.98
Tian, Y., et al. (2019). -0.05 [-4.01, 3.92] 0.53
de la Rosa Oliva, F., et al. (2019). -0.75 [-3.22, 1.73] 1.35
Aredes, M. A., et al. (2019) -1.21 [-3.99, 1.57] 1.07
Camargo, C. D. Q., et al. (2019) -0.74 [-2.47, 0.99] 2.76
Liu, D., et al. (2021). -2.10 [-4.26, 0.06] 1.78
Aziz, L. M. A., et al. (2021) -4.03 [-6.96, -1.11] 0.97
Fukaya, M., et al. (2021) 1.01 [-2.27, 4.29] 0.77
Eghbali, A., et al. (2023). -2.69 [-5.60, 0.21] 0.98
Crichton, M., et al. (2023). -2.58 [-4.64, -0.51] 1.94
Hussein, R. M., et al. (2023). -0.77 [-2.66, 1.12] 2.32
Overall -0.60 [-0.89, -0.31]
Heterogeneity: I^2 = 44.20%, H^2 = 1.79
Test of $\theta_i = \theta_j$: Q(25) = 44.80, p = 0.01
Test of θ = 0: z = -4.09, p = 0.00
-10
-5
0
5

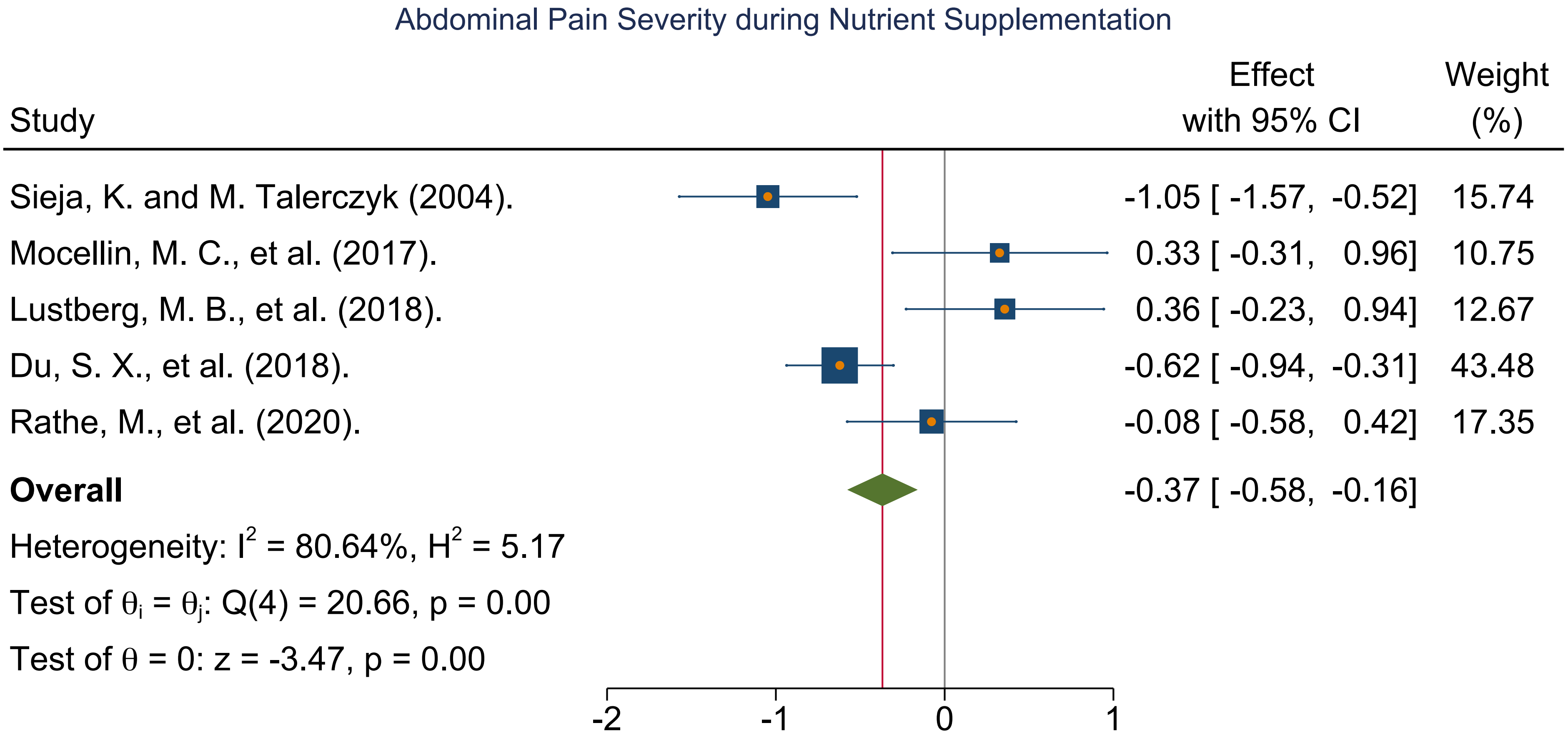
Abdominal Pain Severity during Nutrient Supplementation
Study
Effect with 95% CI
Weight (%)
Sieja, K. and M. Talerczyk (2004). -1.05 [-1.57, -0.52] 15.74
Mocellin, M. C., et al. (2017). 0.33 [-0.31, 0.96] 10.75
Lustberg, M. B., et al. (2018). 0.36 [-0.23, 0.94] 12.67
Du, S. X., et al. (2018). -0.62 [-0.94, -0.31] 43.48
Rathe, M., et al. (2020). -0.08 [-0.58, 0.42] 17.35
Overall -0.37 [-0.58, -0.16]
Heterogeneity: I^2 = 80.64%, H^2 = 5.17
Test of $\theta_i = \theta_j$: Q(4) = 20.66, p = 0.00
Test of θ = 0: z = -3.47, p = 0.00
-2
-1
0
1

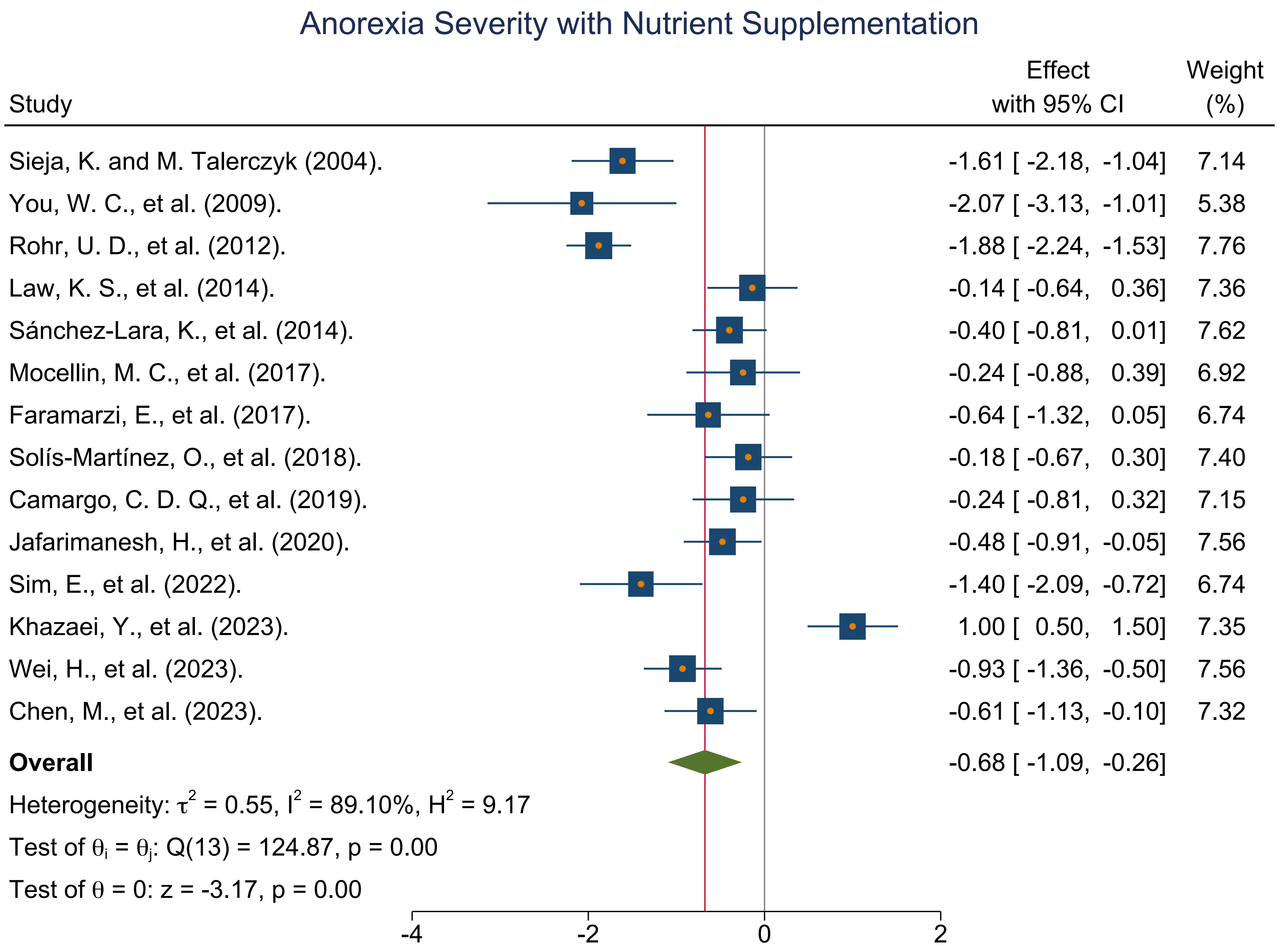
Anorexia Severity with Nutrient Supplementation
Study
Effect with 95% CI
Weight (%)
Sieja, K. and M. Talerczyk (2004). -1.61 [-2.18, -1.04] 7.14
You, W. C., et al. (2009). -2.07 [-3.13, -1.01] 5.38
Rohr, U. D., et al. (2012). -1.88 [-2.24, -1.53] 7.76
Law, K. S., et al. (2014). -0.14 [-0.64, 0.36] 7.36
Sánchez-Lara, K., et al. (2014). -0.40 [-0.81, 0.01] 7.62
Mocellin, M. C., et al. (2017). -0.24 [-0.88, 0.39] 6.92
Faramarzi, E., et al. (2017). -0.64 [-1.32, 0.05] 6.74
Solís-Martínez, O., et al. (2018). -0.18 [-0.67, 0.30] 7.40
Camargo, C. D. Q., et al. (2019). -0.24 [-0.81, 0.32] 7.15
Jafarimanesh, H., et al. (2020). -0.48 [-0.91, -0.05] 7.56
Sim, E., et al. (2022). -1.40 [-2.09, -0.72] 6.74
Khazaei, Y., et al. (2023). 1.00 [0.50, 1.50] 7.35
Wei, H., et al. (2023). -0.93 [-1.36, -0.50] 7.56
Chen, M., et al. (2023). -0.61 [-1.13, -0.10] 7.32
Overall -0.68 [-1.09, -0.26]
Heterogeneity: τ^2 = 0.55, I^2 = 89.10%, H^2 = 9.17
Test of $\theta_i = \theta_j$: Q(13) = 124.87, p = 0.00
Test of θ = 0: z = -3.17, p = 0.00
-4
-2
0
2

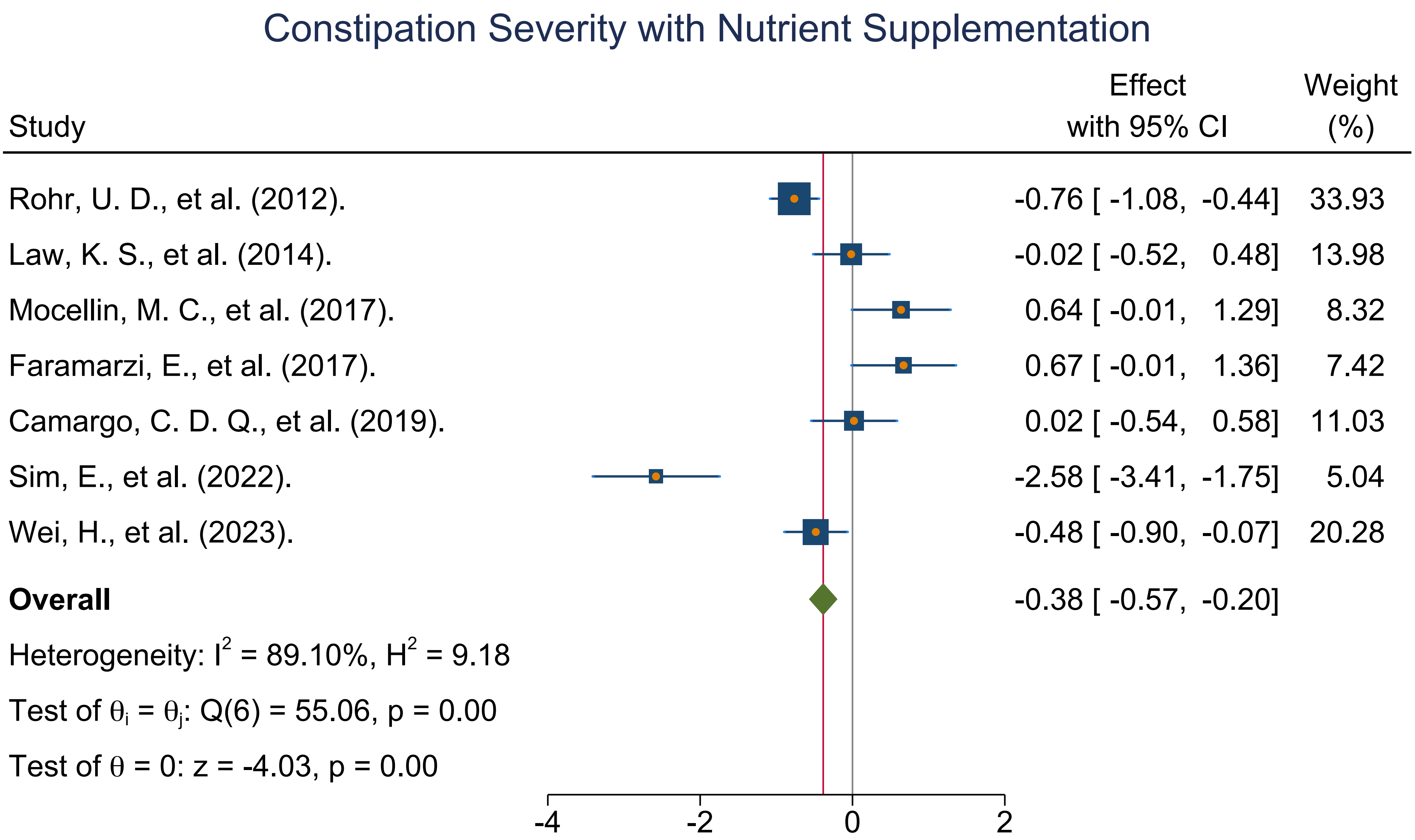
Constipation Severity with Nutrient Supplementation
Study
Effect with 95% CI
Weight (%)
Rohr, U. D., et al. (2012). -0.76 [-1.08, -0.44] 33.93
Law, K. S., et al. (2014). -0.02 [-0.52, 0.48] 13.98
Mocellin, M. C., et al. (2017). 0.64 [-0.01, 1.29] 8.32
Faramarzi, E., et al. (2017). 0.67 [-0.01, 1.36] 7.42
Camargo, C. D. Q., et al. (2019). 0.02 [-0.54, 0.58] 11.03
Sim, E., et al. (2022). -2.58 [-3.41, -1.75] 5.04
Wei, H., et al. (2023). -0.48 [-0.90, -0.07] 20.28
Overall -0.38 [-0.57, -0.20]
Heterogeneity: I^2 = 89.10%, H^2 = 9.18
Test of $\theta_i = \theta_j$: Q(6) = 55.06, p = 0.00
Test of θ = 0: z = -4.03, p = 0.00
-4
-2
0
2

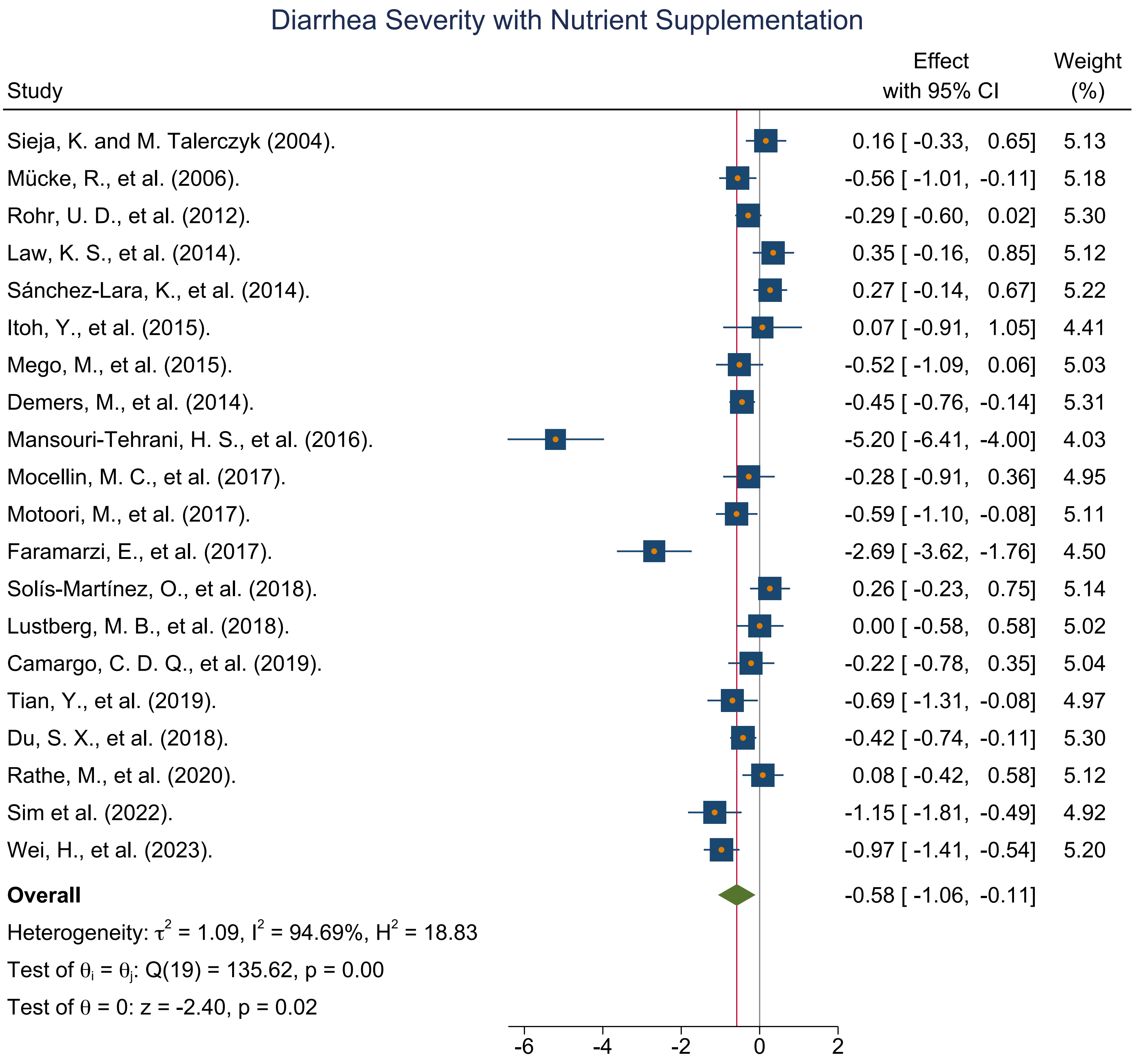

Diarrhea Severity with Nutrient Supplementation
Study
Effect with 95% CI
Weight (%)
Sieja, K. and M. Talerczyk (2004). 0.16 [-0.33, 0.65] 5.13
Mücke, R., et al. (2006). -0.56 [-1.01, -0.11] 5.18
Rohr, U. D., et al. (2012). -0.29 [-0.60, 0.02] 5.30
Law, K. S., et al. (2014). 0.35 [-0.16, 0.85] 5.12
Sánchez-Lara, K., et al. (2014). 0.27 [-0.14, 0.67] 5.22
Itoh, Y., et al. (2015). 0.07 [-0.91, 1.05] 4.41
Mego, M., et al. (2015). -0.52 [-1.09, 0.06] 5.03
Demers, M., et al. (2014). -0.45 [-0.76, -0.14] 5.31
Mansouri-Tehrani, H. S., et al. (2016). -5.20 [-6.41, -4.00] 4.03
Mocellin, M. C., et al. (2017). -0.28 [-0.91, 0.36] 4.95
Motoori, M., et al. (2017). -0.59 [-1.10, -0.08] 5.11
Faramarzi, E., et al. (2017). -2.69 [-3.62, -1.76] 4.50
Solís-Martínez, O., et al. (2018). 0.26 [-0.23, 0.75] 5.14
Lustberg, M. B., et al. (2018). 0.00 [-0.58, 0.58] 5.02
Camargo, C. D. Q., et al. (2019). -0.22 [-0.78, 0.35] 5.04
Tian, Y., et al. (2019). -0.69 [-1.31, -0.08] 4.97
Du, S. X., et al. (2018). -0.42 [-0.74, -0.11] 5.30
Rathe, M., et al. (2020). 0.08 [-0.42, 0.58] 5.12
Sim et al. (2022). -1.15 [-1.81, -0.49] 4.92
Wei, H., et al. (2023). -0.97 [-1.41, -0.54] 5.20
Overall -0.58 [-1.06, -0.11]
Heterogeneity: τ^2 = 1.09, I^2 = 94.69%, H^2 = 18.83
Test of $\theta_i = \theta_j$: Q(19) = 135.62, p = 0.00
Test of θ = 0: z = -2.40, p = 0.02
-6
-4
-2
0
2

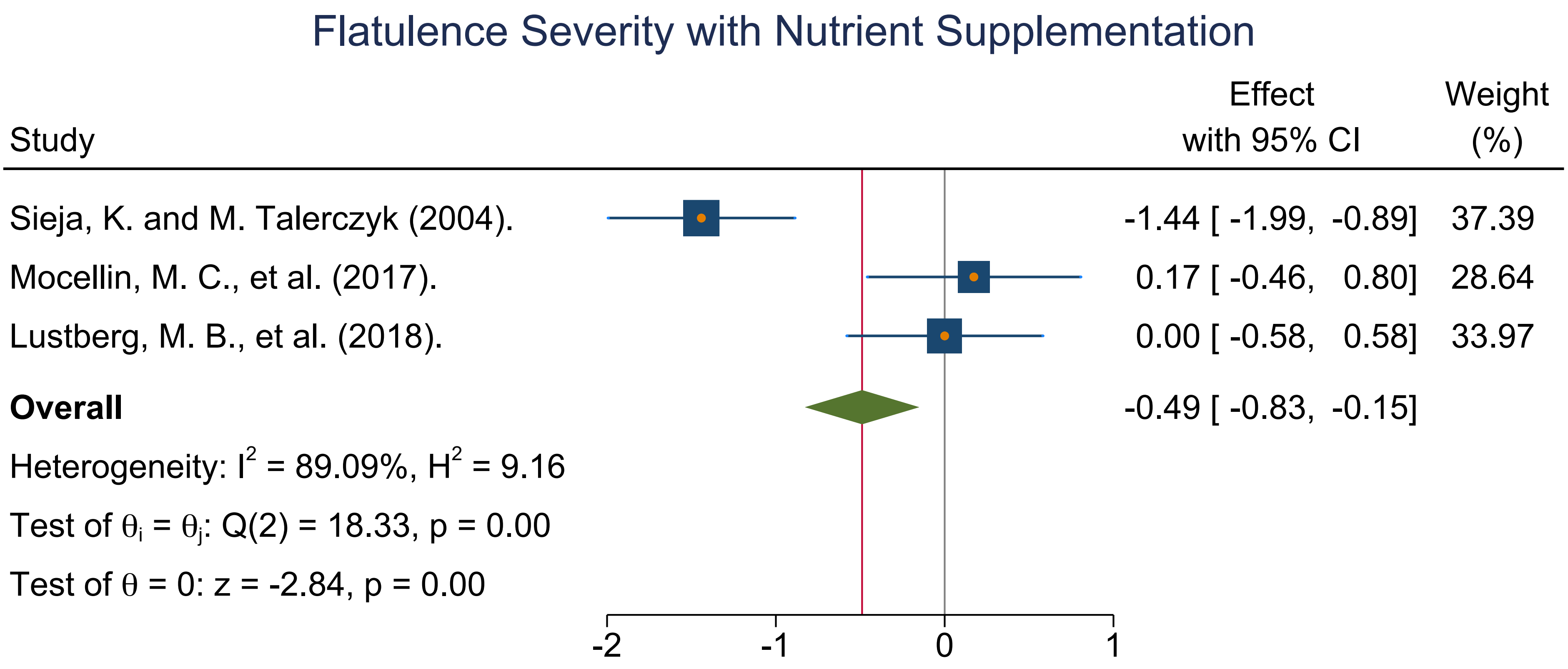
Flatulence Severity with Nutrient Supplementation
Study
Effect with 95% CI
Weight (%)
Sieja, K. and M. Talerczyk (2004).
-1.44 [-1.99, -0.89]
37.39
Mocellin, M. C., et al. (2017).
0.17 [-0.46, 0.80]
28.64
Lustberg, M. B., et al. (2018).
0.00 [-0.58, 0.58]
33.97
Overall
-0.49 [-0.83, -0.15]
Heterogeneity: I^2 = 89.09%, H^2 = 9.16
Test of $\theta_i = \theta_j$: Q(2) = 18.33, p = 0.00
Test of θ = 0: z = -2.84, p = 0.00
-2
-1
0
1

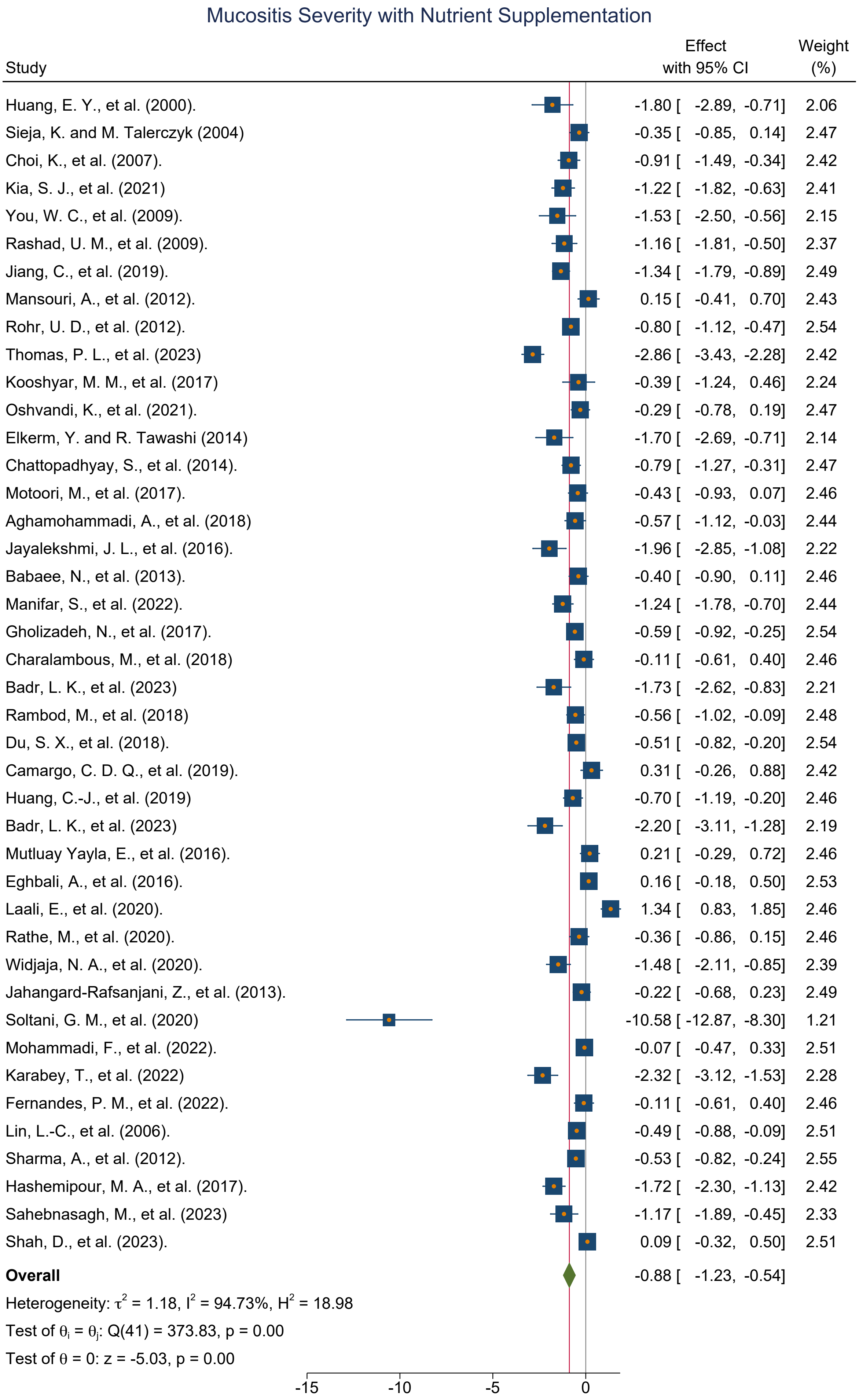
Mucositis Severity with Nutrient Supplementation
Study
Effect with 95% CI
Weight (%)
Huang, E. Y., et al. (2000). -1.80 [-2.89, -0.71] 2.06
Sieja, K. and M. Talerczyk (2004) -0.35 [-0.85, 0.14] 2.47
Choi, K., et al. (2007). -0.91 [-1.49, -0.34] 2.42
Kia, S. J., et al. (2021) -1.22 [-1.82, -0.63] 2.41
You, W. C., et al. (2009). -1.53 [-2.50, -0.56] 2.15
Rashad, U. M., et al. (2009). -1.16 [-1.81, -0.50] 2.37
Jiang, C., et al. (2019). -1.34 [-1.79, -0.89] 2.49
Mansouri, A., et al. (2012). 0.15 [-0.41, 0.70] 2.43
Rohr, U. D., et al. (2012). -0.80 [-1.12, -0.47] 2.54
Thomas, P. L., et al. (2023) -2.86 [-3.43, -2.28] 2.42
Kooshyar, M. M., et al. (2017) -0.39 [-1.24, 0.46] 2.24
Oshvandi, K., et al. (2021). -0.29 [-0.78, 0.19] 2.47
Elkerm, Y. and R. Tawashi (2014) -1.70 [-2.69, -0.71] 2.14
Chattopadhyay, S., et al. (2014). -0.79 [-1.27, -0.31] 2.47
Motoori, M., et al. (2017). -0.43 [-0.93, 0.07] 2.46
Aghamohammadi, A., et al. (2018) -0.57 [-1.12, -0.03] 2.44
Jayalekshmi, J. L., et al. (2016). -1.96 [-2.85, -1.08] 2.22
Babaee, N., et al. (2013). -0.40 [-0.90, 0.11] 2.46
Manifar, S., et al. (2022). -1.24 [-1.78, -0.70] 2.44
Gholizadeh, N., et al. (2017). -0.59 [-0.92, -0.25] 2.54
Charalambous, M., et al. (2018) -0.11 [-0.61, 0.40] 2.46
Badr, L. K., et al. (2023) -1.73 [-2.62, -0.83] 2.21
Rambod, M., et al. (2018) -0.56 [-1.02, -0.09] 2.48
Du, S. X., et al. (2018). -0.51 [-0.82, -0.20] 2.54
Camargo, C. D. Q., et al. (2019). 0.31 [-0.26, 0.88] 2.42
Huang, C.-J., et al. (2019) -0.70 [-1.19, -0.20] 2.46
Badr, L. K., et al. (2023) -2.20 [-3.11, -1.28] 2.19
Mutluay Yayla, E., et al. (2016). 0.21 [-0.29, 0.72] 2.46
Eghbali, A., et al. (2016). 0.16 [-0.18, 0.50] 2.53
Laali, E., et al. (2020). 1.34 [0.83, 1.85] 2.46
Rathe, M., et al. (2020). -0.36 [-0.86, 0.15] 2.46
Widjaja, N. A., et al. (2020). -1.48 [-2.11, -0.85] 2.39
Jahangard-Rafsanjani, Z., et al. (2013). -0.22 [-0.68, 0.23] 2.49
Soltani, G. M., et al. (2020) -10.58 [-12.87, -8.30] 1.21
Mohammadi, F., et al. (2022). -0.07 [-0.47, 0.33] 2.51
Karabey, T., et al. (2022) -2.32 [-3.12, -1.53] 2.28
Fernandes, P. M., et al. (2022). -0.11 [-0.61, 0.40] 2.46
Lin, L.-C., et al. (2006). -0.49 [-0.88, -0.09] 2.51
Sharma, A., et al. (2012). -0.53 [-0.82, -0.24] 2.55
Hashemipour, M. A., et al. (2017). -1.72 [-2.30, -1.13] 2.42
Sahebnasagh, M., et al. (2023) -1.17 [-1.89, -0.45] 2.33
Shah, D., et al. (2023). 0.09 [-0.32, 0.50] 2.51
Overall -0.88 [-1.23, -0.54]
Heterogeneity: τ^2 = 1.18, I^2 = 94.73%, H^2 = 18.98
Test of $\theta_i = \theta_j$: Q(41) = 373.83, p = 0.00
Test of θ = 0: z = -5.03, p = 0.00
-15
-10
-5
0

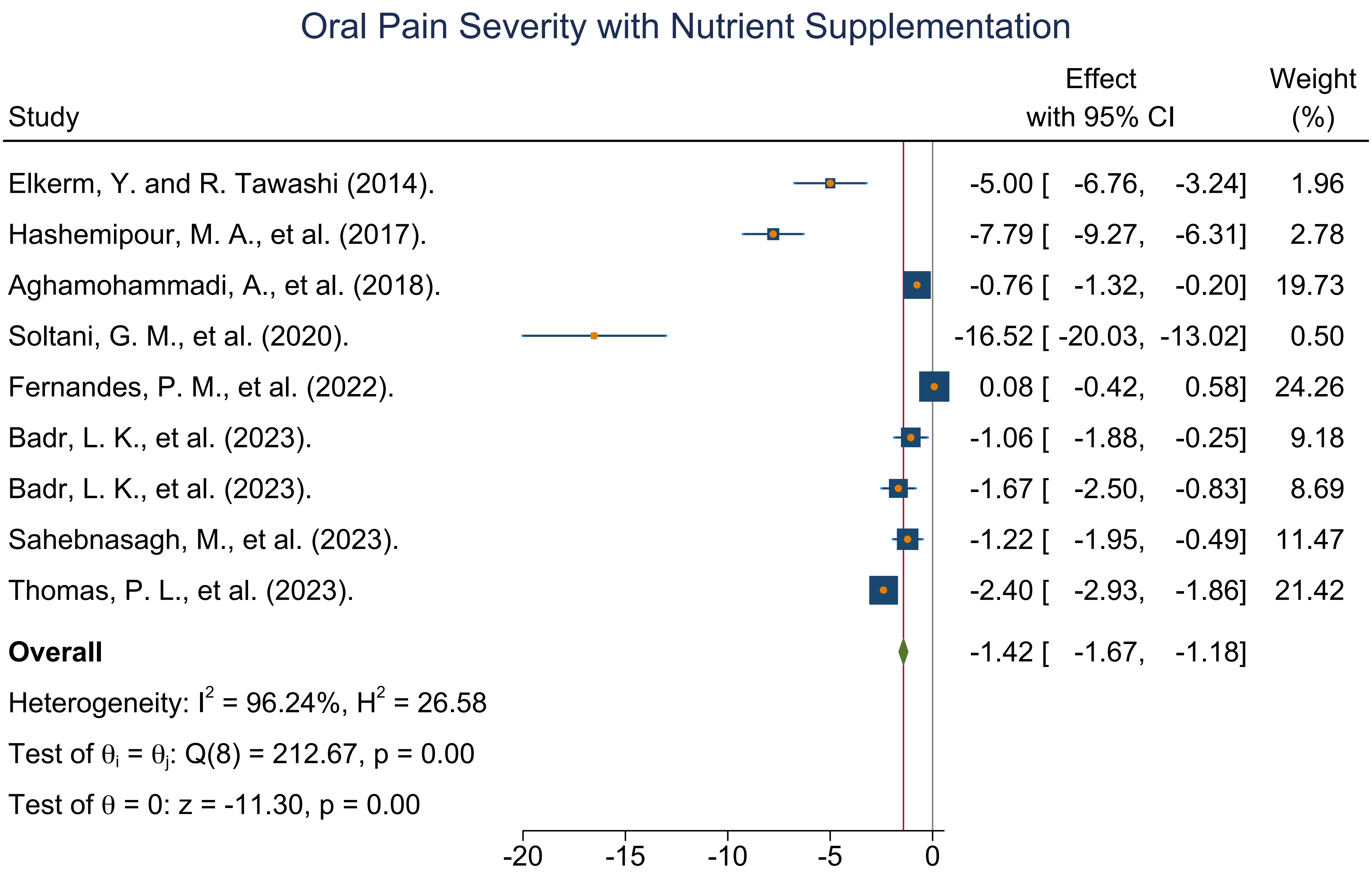

Oral Pain Severity with Nutrient Supplementation
Study
Effect with 95% CI
Weight (%)
Elkerm, Y. and R. Tawashi (2014). -5.00 [-6.76, -3.24] 1.96
Hashemipour, M. A., et al. (2017). -7.79 [-9.27, -6.31] 2.78
Aghamohammadi, A., et al. (2018). -0.76 [-1.32, -0.20] 19.73
Soltani, G. M., et al. (2020). -16.52 [-20.03, -13.02] 0.50
Fernandes, P. M., et al. (2022). 0.08 [-0.42, 0.58] 24.26
Badr, L. K., et al. (2023). -1.06 [-1.88, -0.25] 9.18
Badr, L. K., et al. (2023). -1.67 [-2.50, -0.83] 8.69
Sahebnasagh, M., et al. (2023). -1.22 [-1.95, -0.49] 11.47
Thomas, P. L., et al. (2023). -2.40 [-2.93, -1.86] 21.42
Overall -1.42 [-1.67, -1.18]
Heterogeneity: I^2 = 96.24%, H^2 = 26.58
Test of $\theta_i = \theta_j$: Q(8) = 212.67, p = 0.00
Test of θ = 0: z = -11.30, p = 0.00
-20
-15
-10
-5
0

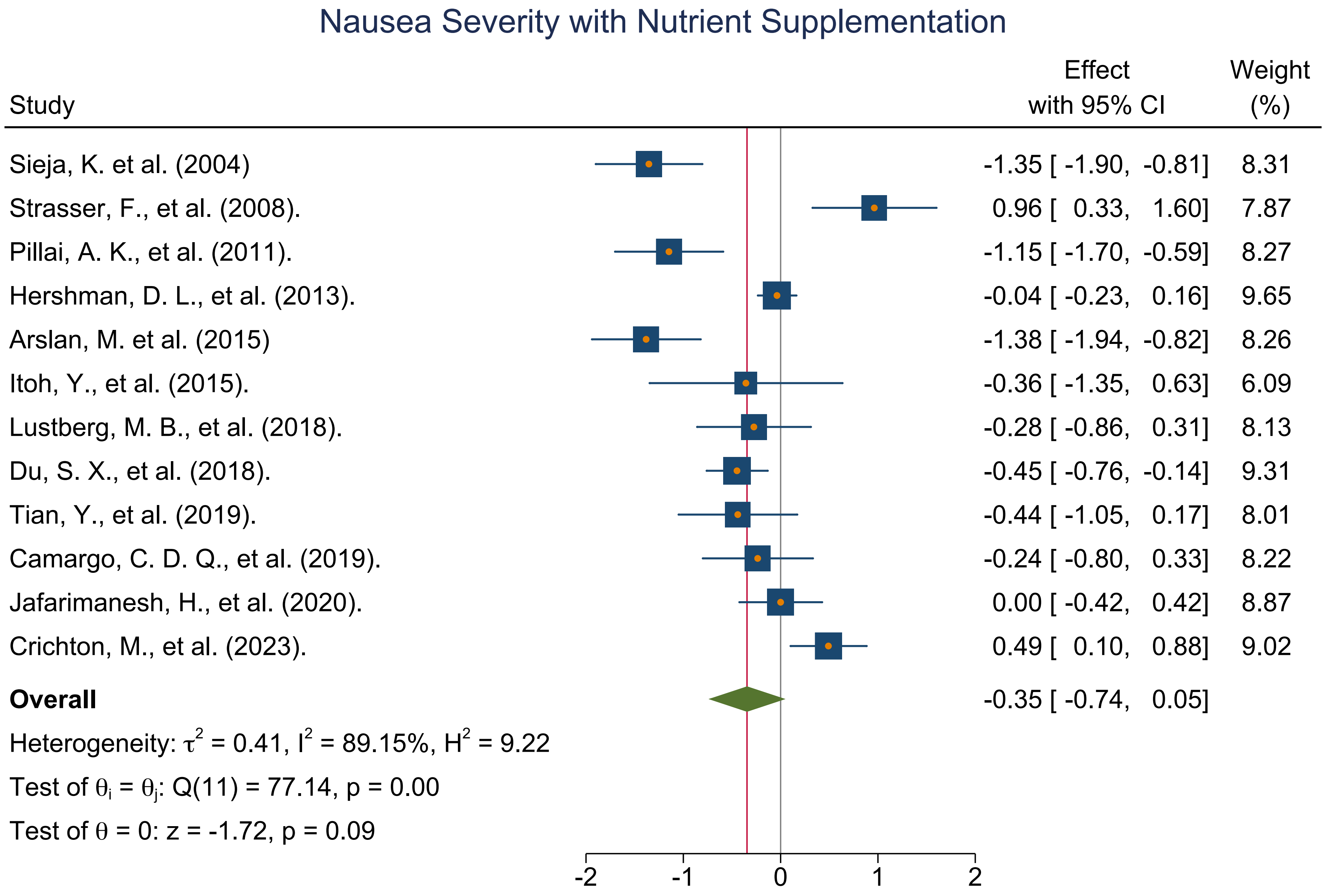

Nausea Severity with Nutrient Supplementation
Study
Effect with 95% CI
Weight (%)
Sieja, K. et al. (2004) -1.35 [-1.90, -0.81] 8.31
Strasser, F., et al. (2008). 0.96 [0.33, 1.60] 7.87
Pillai, A. K., et al. (2011). -1.15 [-1.70, -0.59] 8.27
Hershman, D. L., et al. (2013). -0.04 [-0.23, 0.16] 9.65
Arslan, M. et al. (2015) -1.38 [-1.94, -0.82] 8.26
Itoh, Y., et al. (2015). -0.36 [-1.35, 0.63] 6.09
Lustberg, M. B., et al. (2018). -0.28 [-0.86, 0.31] 8.13
Du, S. X., et al. (2018). -0.45 [-0.76, -0.14] 9.31
Tian, Y., et al. (2019). -0.44 [-1.05, 0.17] 8.01
Camargo, C. D. Q., et al. (2019). -0.24 [-0.80, 0.33] 8.22
Jafarimanesh, H., et al. (2020). 0.00 [-0.42, 0.42] 8.87
Crichton, M., et al. (2023). 0.49 [0.10, 0.88] 9.02
Overall -0.35 [-0.74, 0.05]
Heterogeneity: $\tau^2 = 0.41$, $I^2 = 89.15\%$, $H^2 = 9.22$
Test of $\theta_i = \theta_j$: Q(11) = 77.14, p = 0.00
Test of $\theta = 0$: z = -1.72, p = 0.09
-2 -1 0 1 2

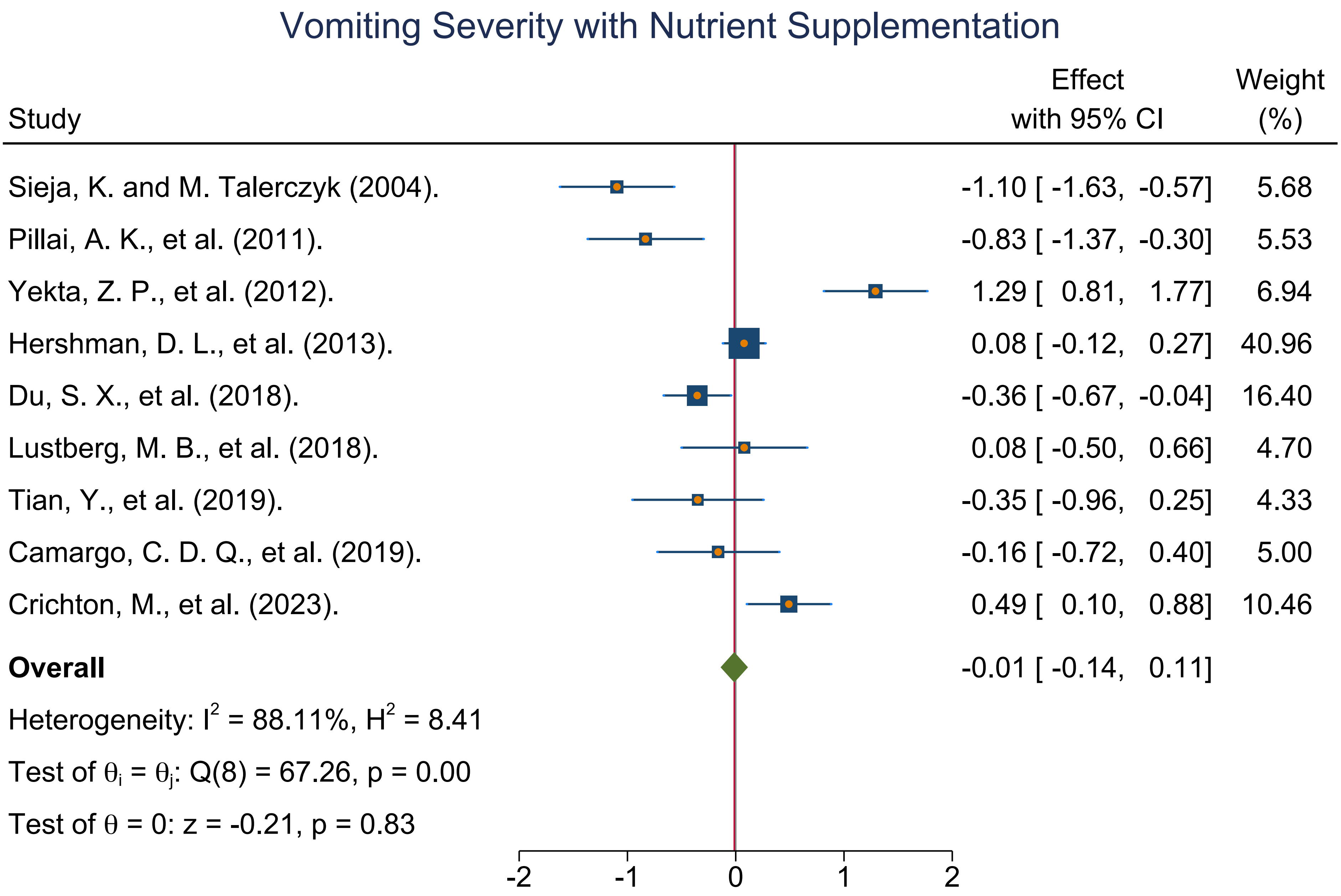
Vomiting Severity with Nutrient Supplementation
Study
Effect with 95% CI
Weight (%)
Sieja, K. and M. Talerczyk (2004). -1.10 [-1.63, -0.57] 5.68
Pillai, A. K., et al. (2011). -0.83 [-1.37, -0.30] 5.53
Yekta, Z. P., et al. (2012). 1.29 [0.81, 1.77] 6.94
Hershman, D. L., et al. (2013). 0.08 [-0.12, 0.27] 40.96
Du, S. X., et al. (2018). -0.36 [-0.67, -0.04] 16.40
Lustberg, M. B., et al. (2018). 0.08 [-0.50, 0.66] 4.70
Tian, Y., et al. (2019). -0.35 [-0.96, 0.25] 4.33
Camargo, C. D. Q., et al. (2019). -0.16 [-0.72, 0.40] 5.00
Crichton, M., et al. (2023). 0.49 [0.10, 0.88] 10.46
Overall -0.01 [-0.14, 0.11]
Heterogeneity: I^2 = 88.11%, H^2 = 8.41
Test of $\theta_i = \theta_j$: Q(8) = 67.26, p = 0.00
Test of θ = 0: z = -0.21, p = 0.83
-2
-1
0
1
2

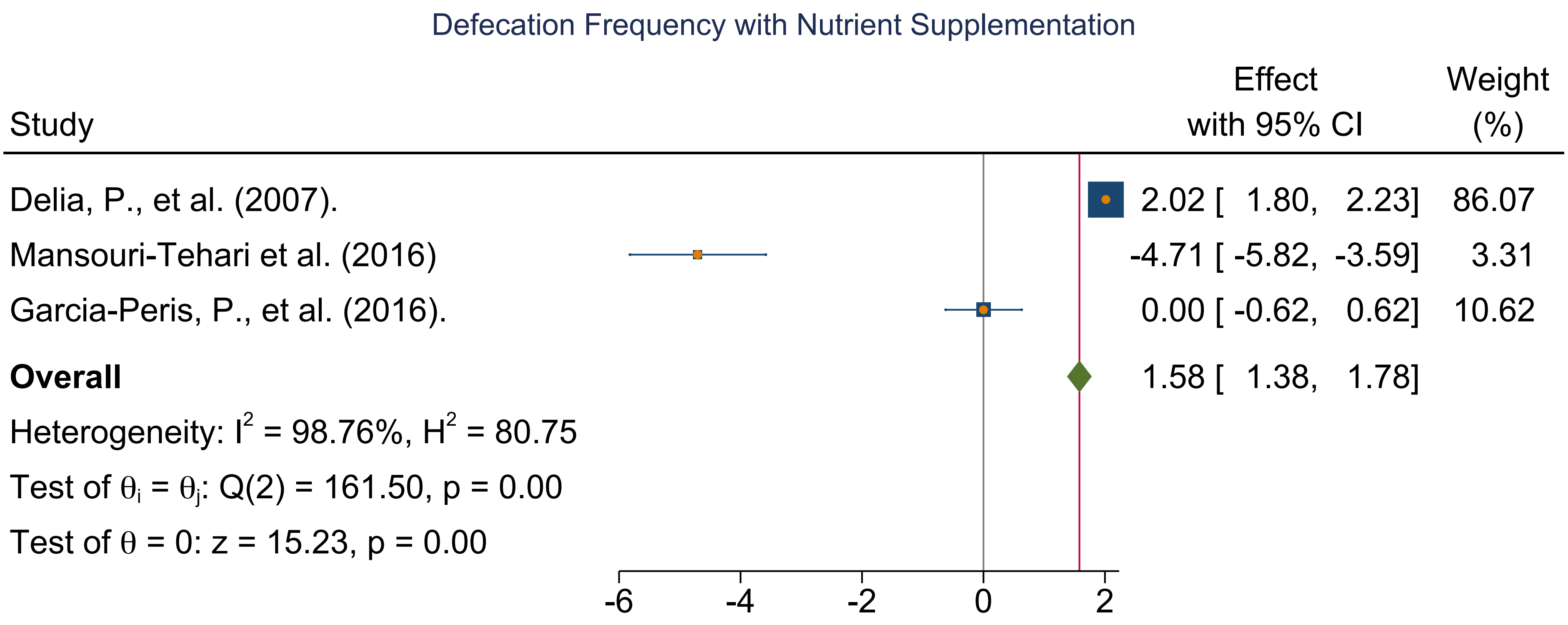

Defecation Frequency with Nutrient Supplementation
Study
Effect with 95% CI
Weight (%)
Delia, P., et al. (2007). 2.02 [1.80, 2.23] 86.07
Mansouri-Tehari et al. (2016) -4.71 [-5.82, -3.59] 3.31
Garcia-Peris, P., et al. (2016). 0.00 [-0.62, 0.62] 10.62
Overall 1.58 [1.38, 1.78]
Heterogeneity: I^2 = 98.76%, H^2 = 80.75
Test of $\theta_i = \theta_j$: Q(2) = 161.50, p = 0.00
Test of θ = 0: z = 15.23, p = 0.00
-6
-4
-2
0
2

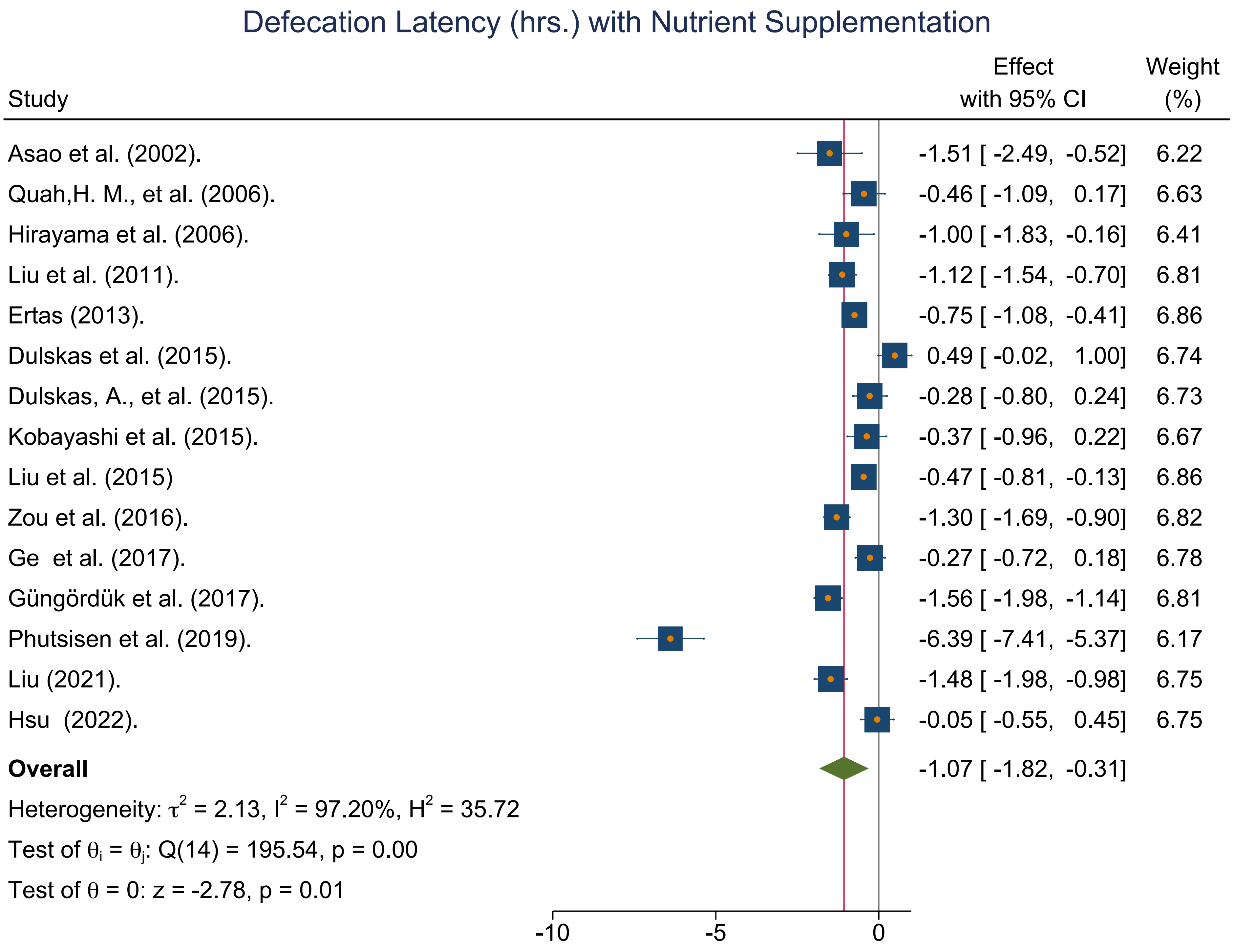

Defecation Latency (hrs.) with Nutrient Supplementation
Study
Effect with 95% CI
Weight (%)
Asao et al. (2002). -1.51 [-2.49, -0.52] 6.22
Quah,H. M., et al. (2006). -0.46 [-1.09, 0.17] 6.63
Hirayama et al. (2006). -1.00 [-1.83, -0.16] 6.41
Liu et al. (2011). -1.12 [-1.54, -0.70] 6.81
Ertas (2013). -0.75 [-1.08, -0.41] 6.86
Dulskas et al. (2015). 0.49 [-0.02, 1.00] 6.74
Dulskas, A., et al. (2015). -0.28 [-0.80, 0.24] 6.73
Kobayashi et al. (2015). -0.37 [-0.96, 0.22] 6.67
Liu et al. (2015) -0.47 [-0.81, -0.13] 6.86
Zou et al. (2016). -1.30 [-1.69, -0.90] 6.82
Ge et al. (2017). -0.27 [-0.72, 0.18] 6.78
Güngördük et al. (2017). -1.56 [-1.98, -1.14] 6.81
Phutsisen et al. (2019). -6.39 [-7.41, -5.37] 6.17
Liu (2021). -1.48 [-1.98, -0.98] 6.75
Hsu (2022). -0.05 [-0.55, 0.45] 6.75
Overall -1.07 [-1.82, -0.31]
Heterogeneity: τ² = 2.13, I² = 97.20%, H² = 35.72
Test of θi = θj: Q(14) = 195.54, p = 0.00
Test of θ = 0: z = -2.78, p = 0.01
-10
-5
0

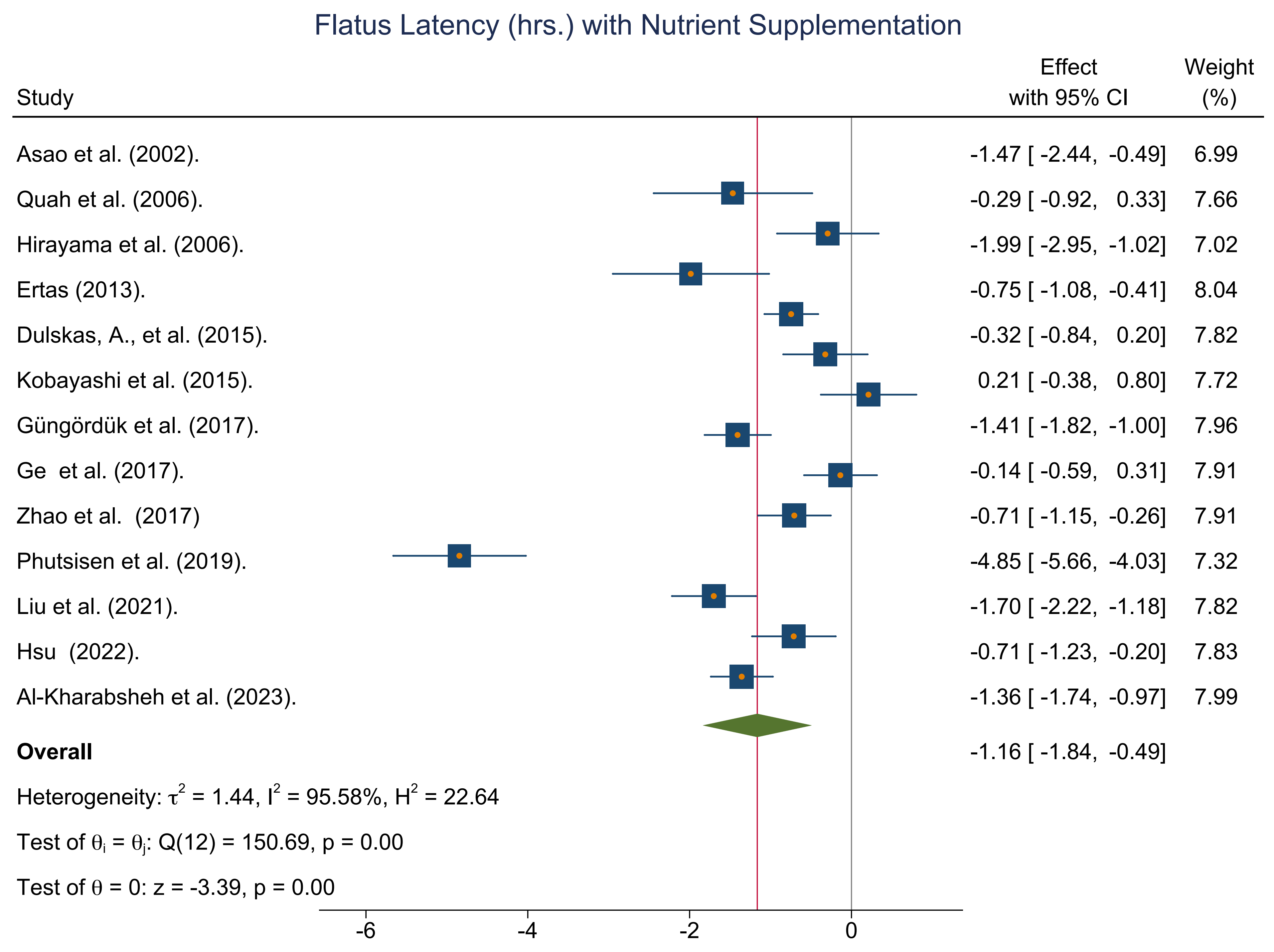
Flatus Latency (hrs.) with Nutrient Supplementation
Study
Effect with 95% CI
Weight (%)
Asao et al. (2002). -1.47 [-2.44, -0.49] 6.99
Quah et al. (2006). -0.29 [-0.92, 0.33] 7.66
Hirayama et al. (2006). -1.99 [-2.95, -1.02] 7.02
Ertas (2013). -0.75 [-1.08, -0.41] 8.04
Dulskas, A., et al. (2015). -0.32 [-0.84, 0.20] 7.82
Kobayashi et al. (2015). 0.21 [-0.38, 0.80] 7.72
Güngördük et al. (2017). -1.41 [-1.82, -1.00] 7.96
Ge et al. (2017). -0.14 [-0.59, 0.31] 7.91
Zhao et al. (2017) -0.71 [-1.15, -0.26] 7.91
Phutsisen et al. (2019). -4.85 [-5.66, -4.03] 7.32
Liu et al. (2021). -1.70 [-2.22, -1.18] 7.82
Hsu (2022). -0.71 [-1.23, -0.20] 7.83
Al-Kharabsheh et al. (2023). -1.36 [-1.74, -0.97] 7.99
Overall -1.16 [-1.84, -0.49]
Heterogeneity: τ^2 = 1.44, I^2 = 95.58%, H^2 = 22.64
Test of $\theta_i = \theta_j$: Q(12) = 150.69, p = 0.00
Test of θ = 0: z = -3.39, p = 0.00
-6
-4
-2
0

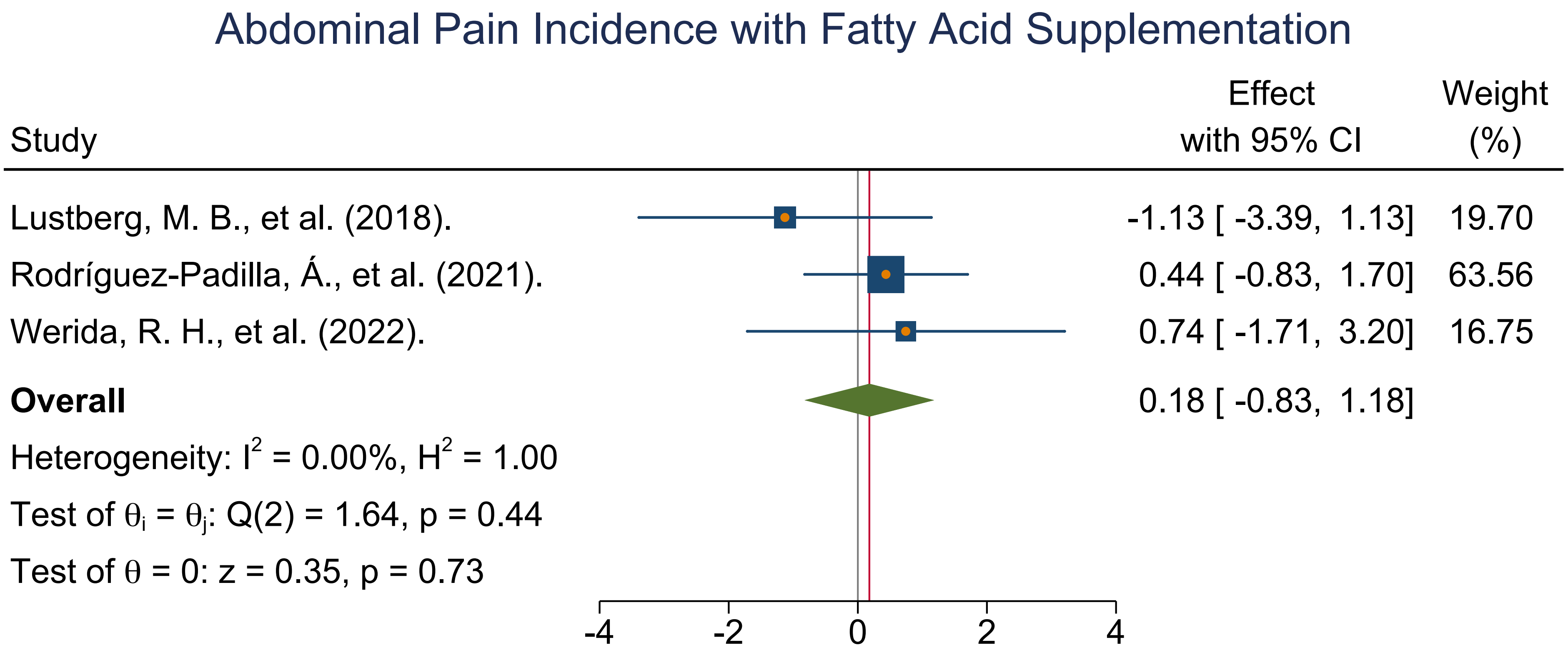
Abdominal Pain Incidence with Fatty Acid Supplementation
Study
Effect with 95% CI
Weight (%)
Lustberg, M. B., et al. (2018).
-1.13 [-3.39, 1.13]
19.70
Rodríguez-Padilla, Á., et al. (2021).
0.44 [-0.83, 1.70]
63.56
Werida, R. H., et al. (2022).
0.74 [-1.71, 3.20]
16.75
Overall
0.18 [-0.83, 1.18]
Heterogeneity: I² = 0.00%, H² = 1.00
Test of θi = θj: Q(2) = 1.64, p = 0.44
Test of θ = 0: z = 0.35, p = 0.73
-4
-2
0
2
4

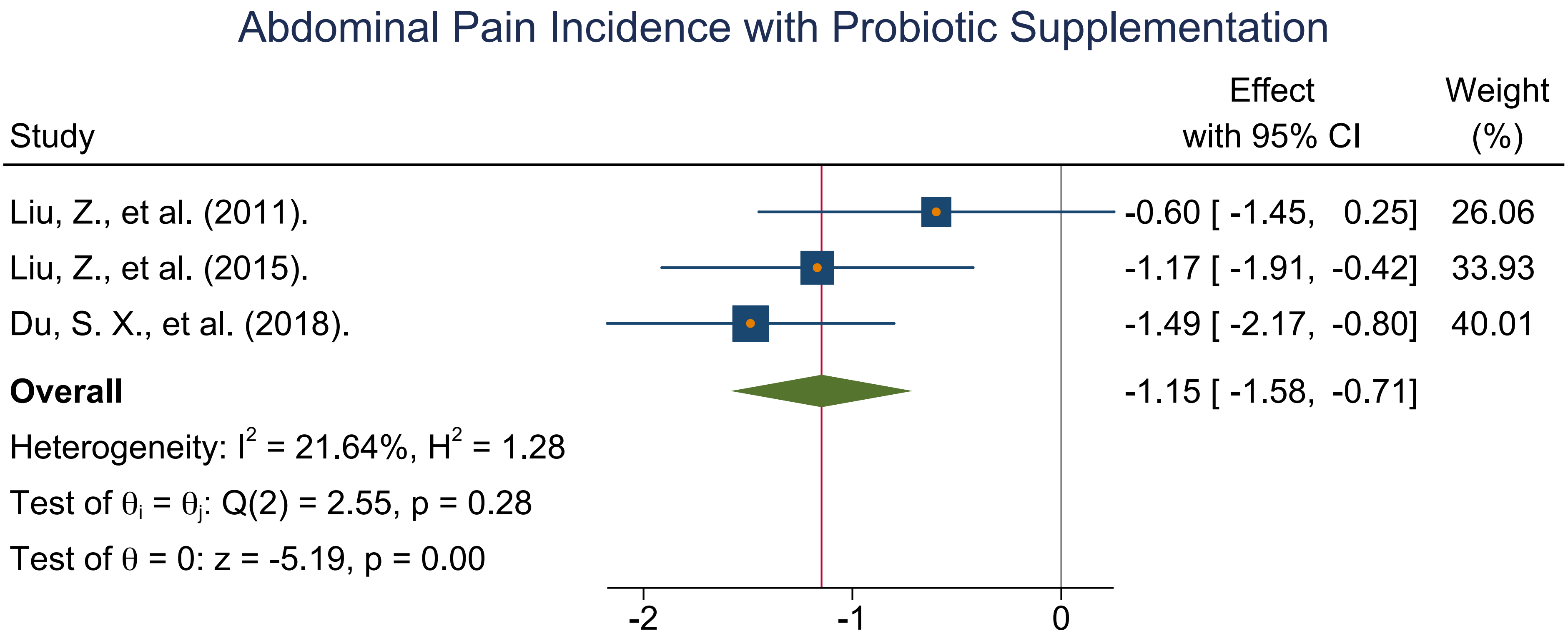
Abdominal Pain Incidence with Probiotic Supplementation
Study
Effect with 95% CI
Weight (%)
Liu, Z., et al. (2011). -0.60 [-1.45, 0.25] 26.06
Liu, Z., et al. (2015). -1.17 [-1.91, -0.42] 33.93
Du, S. X., et al. (2018). -1.49 [-2.17, -0.80] 40.01
Overall -1.15 [-1.58, -0.71]
Heterogeneity: I^2 = 21.64%, H^2 = 1.28
Test of $\theta_i = \theta_j$: Q(2) = 2.55, p = 0.28
Test of θ = 0: z = -5.19, p = 0.00
-2
-1
0

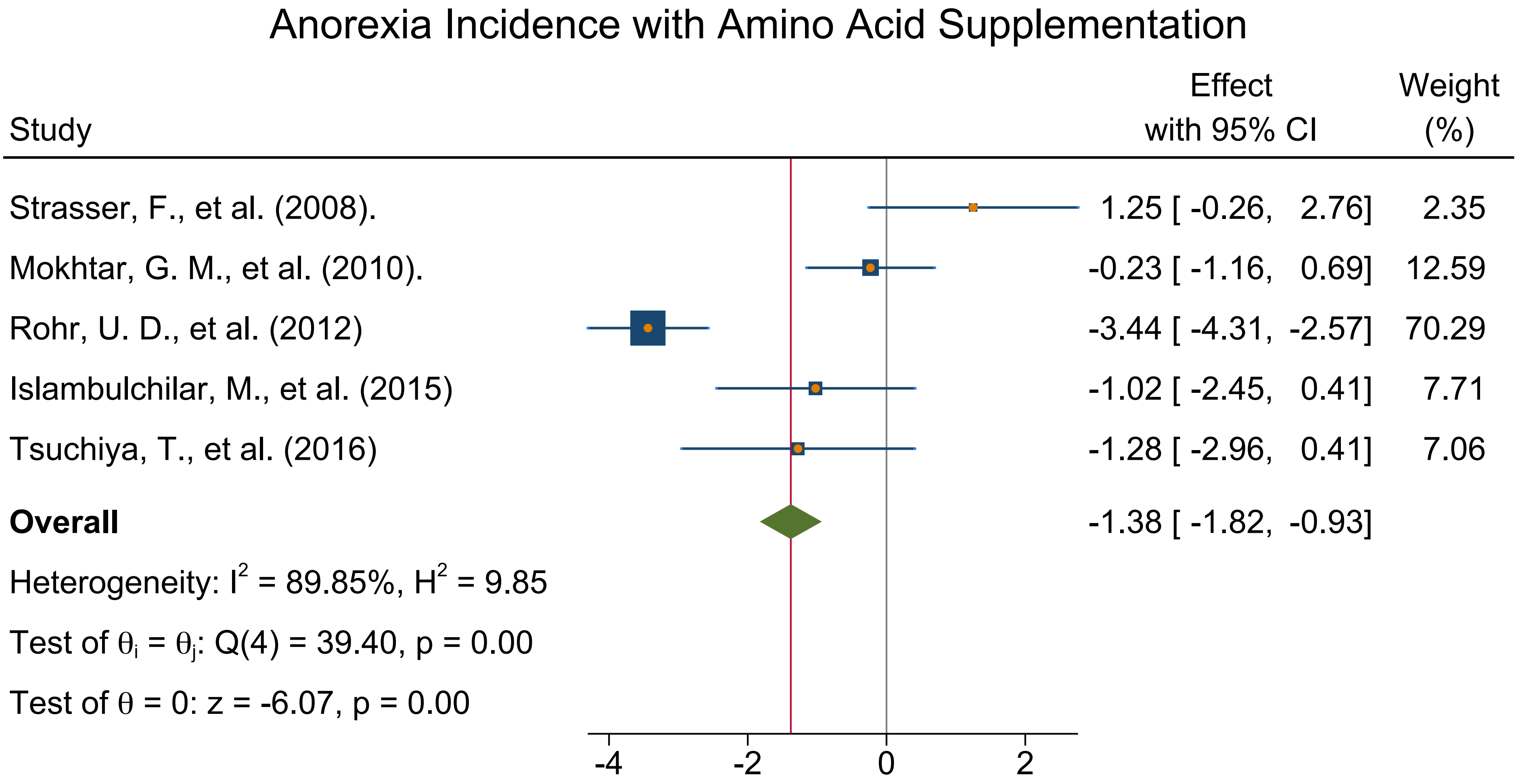
Anorexia Incidence with Amino Acid Supplementation
Study
Effect with 95% CI
Weight (%)
Strasser, F., et al. (2008). 1.25 [-0.26, 2.76] 2.35
Mokhtar, G. M., et al. (2010). -0.23 [-1.16, 0.69] 12.59
Rohr, U. D., et al. (2012) -3.44 [-4.31, -2.57] 70.29
Islambulchilar, M., et al. (2015) -1.02 [-2.45, 0.41] 7.71
Tsuchiya, T., et al. (2016) -1.28 [-2.96, 0.41] 7.06
Overall -1.38 [-1.82, -0.93]
Heterogeneity: I² = 89.85%, H² = 9.85
Test of θi = θj: Q(4) = 39.40, p = 0.00
Test of θ = 0: z = -6.07, p = 0.00
-4
-2
0
2

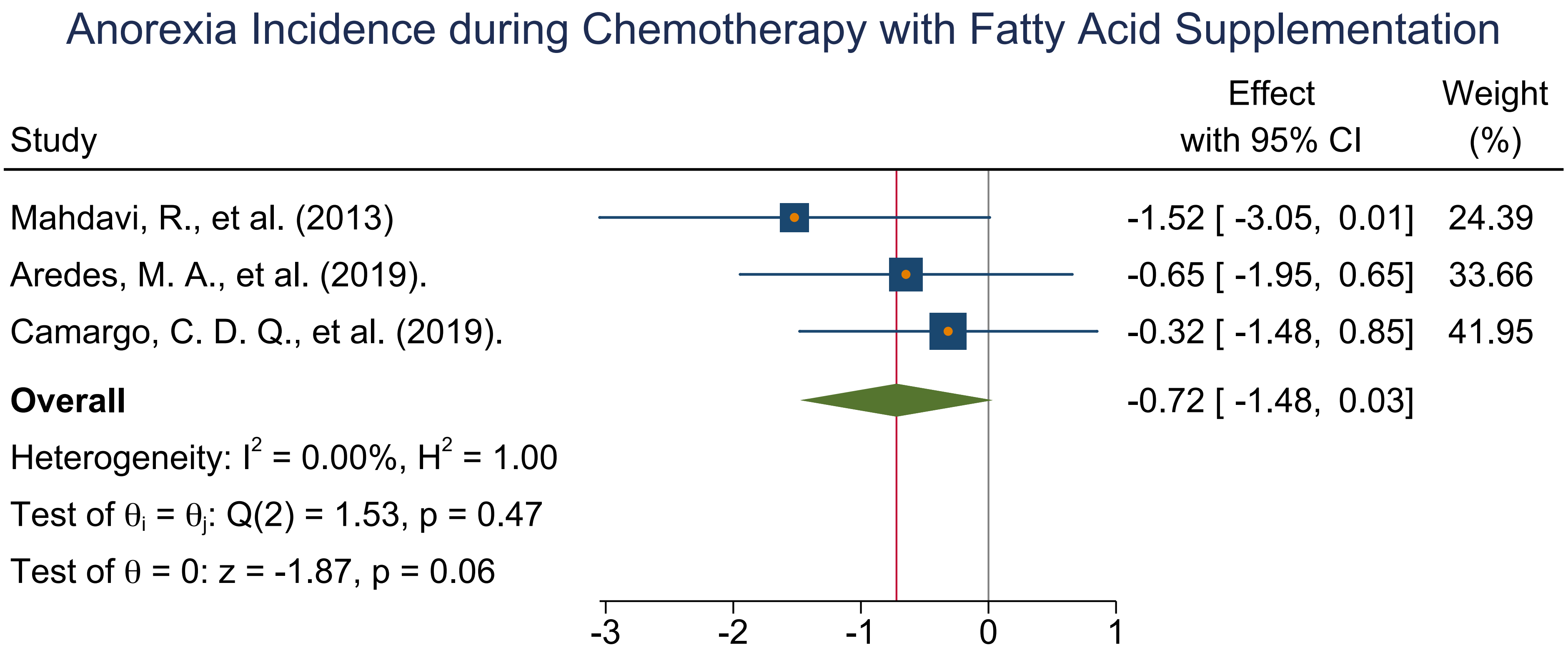
Anorexia Incidence during Chemotherapy with Fatty Acid Supplementation
Study
Effect with 95% CI
Weight (%)
Mahdavi, R., et al. (2013)
-1.52 [-3.05, 0.01]
24.39
Aredes, M. A., et al. (2019).
-0.65 [-1.95, 0.65]
33.66
Camargo, C. D. Q., et al. (2019).
-0.32 [-1.48, 0.85]
41.95
Overall
-0.72 [-1.48, 0.03]
Heterogeneity: I2 = 0.00%, H2 = 1.00
Test of θi = θj: Q(2) = 1.53, p = 0.47
Test of θ = 0: z = -1.87, p = 0.06
-3
-2
-1
0
1

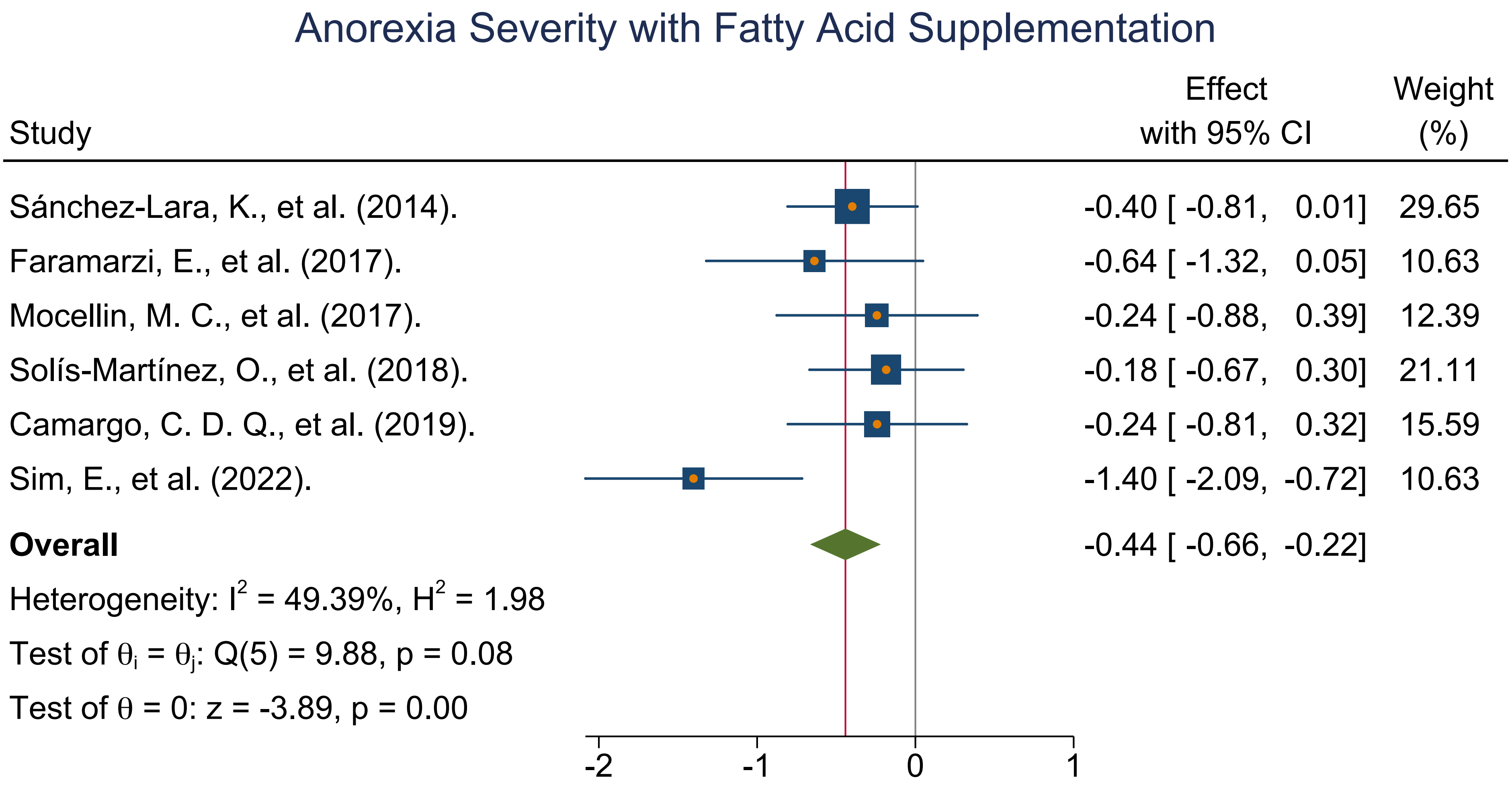
Anorexia Severity with Fatty Acid Supplementation
Study
Effect with 95% CI
Weight (%)
Sánchez-Lara, K., et al. (2014). -0.40 [-0.81, 0.01] 29.65
Faramarzi, E., et al. (2017). -0.64 [-1.32, 0.05] 10.63
Mocellin, M. C., et al. (2017). -0.24 [-0.88, 0.39] 12.39
Solís-Martínez, O., et al. (2018). -0.18 [-0.67, 0.30] 21.11
Camargo, C. D. Q., et al. (2019). -0.24 [-0.81, 0.32] 15.59
Sim, E., et al. (2022). -1.40 [-2.09, -0.72] 10.63
Overall -0.44 [-0.66, -0.22]
Heterogeneity: I^2 = 49.39%, H^2 = 1.98
Test of $\theta_i = \theta_j$: Q(5) = 9.88, p = 0.08
Test of θ = 0: z = -3.89, p = 0.00
-2
-1
0
1

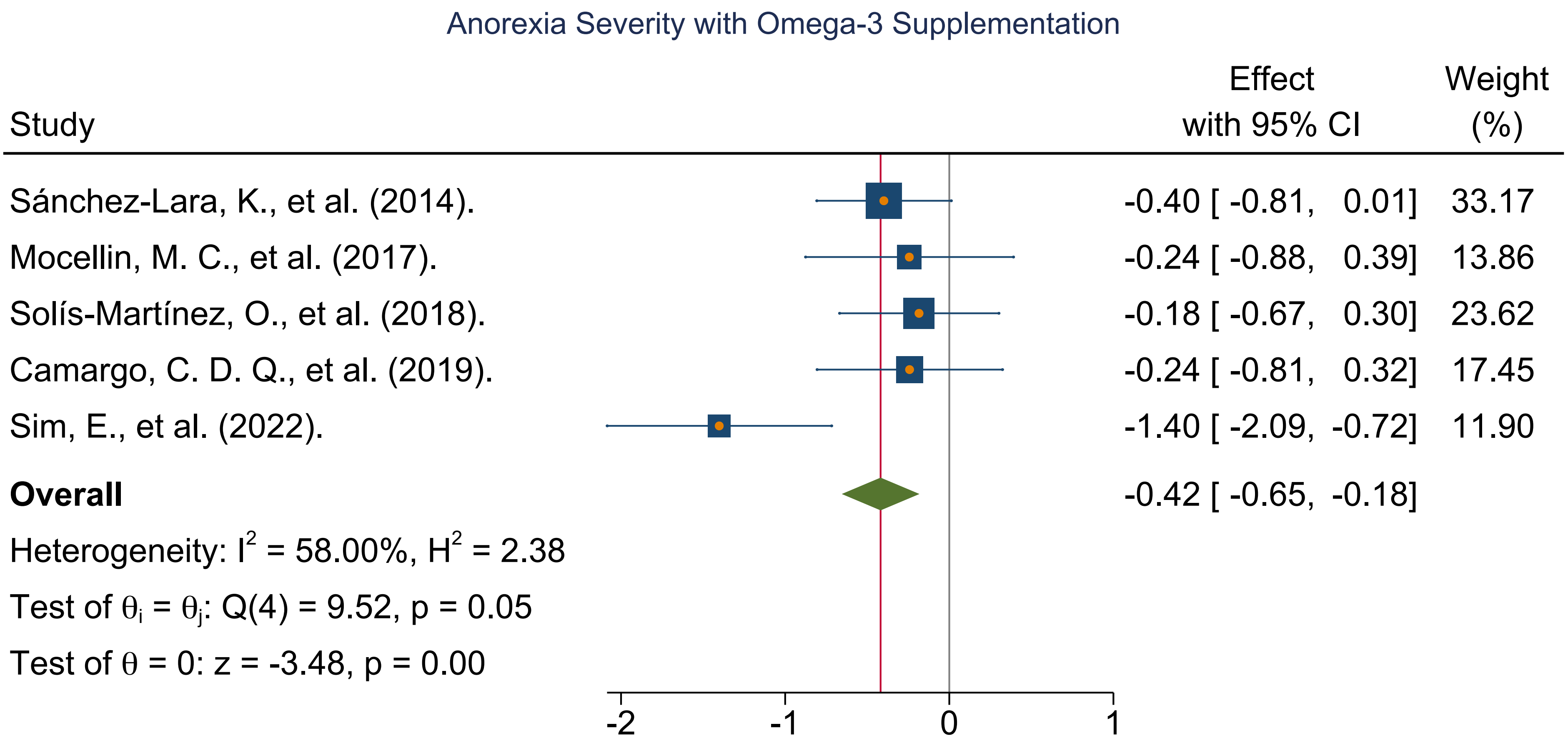
Anorexia Severity with Omega-3 Supplementation
Study
Effect with 95% CI
Weight (%)
Sánchez-Lara, K., et al. (2014). -0.40 [-0.81, 0.01] 33.17
Mocellin, M. C., et al. (2017). -0.24 [-0.88, 0.39] 13.86
Solís-Martínez, O., et al. (2018). -0.18 [-0.67, 0.30] 23.62
Camargo, C. D. Q., et al. (2019). -0.24 [-0.81, 0.32] 17.45
Sim, E., et al. (2022). -1.40 [-2.09, -0.72] 11.90
Overall -0.42 [-0.65, -0.18]
Heterogeneity: I^2 = 58.00%, H^2 = 2.38
Test of $\theta_i = \theta_j$: Q(4) = 9.52, p = 0.05
Test of θ = 0: z = -3.48, p = 0.00
-2
-1
0
1

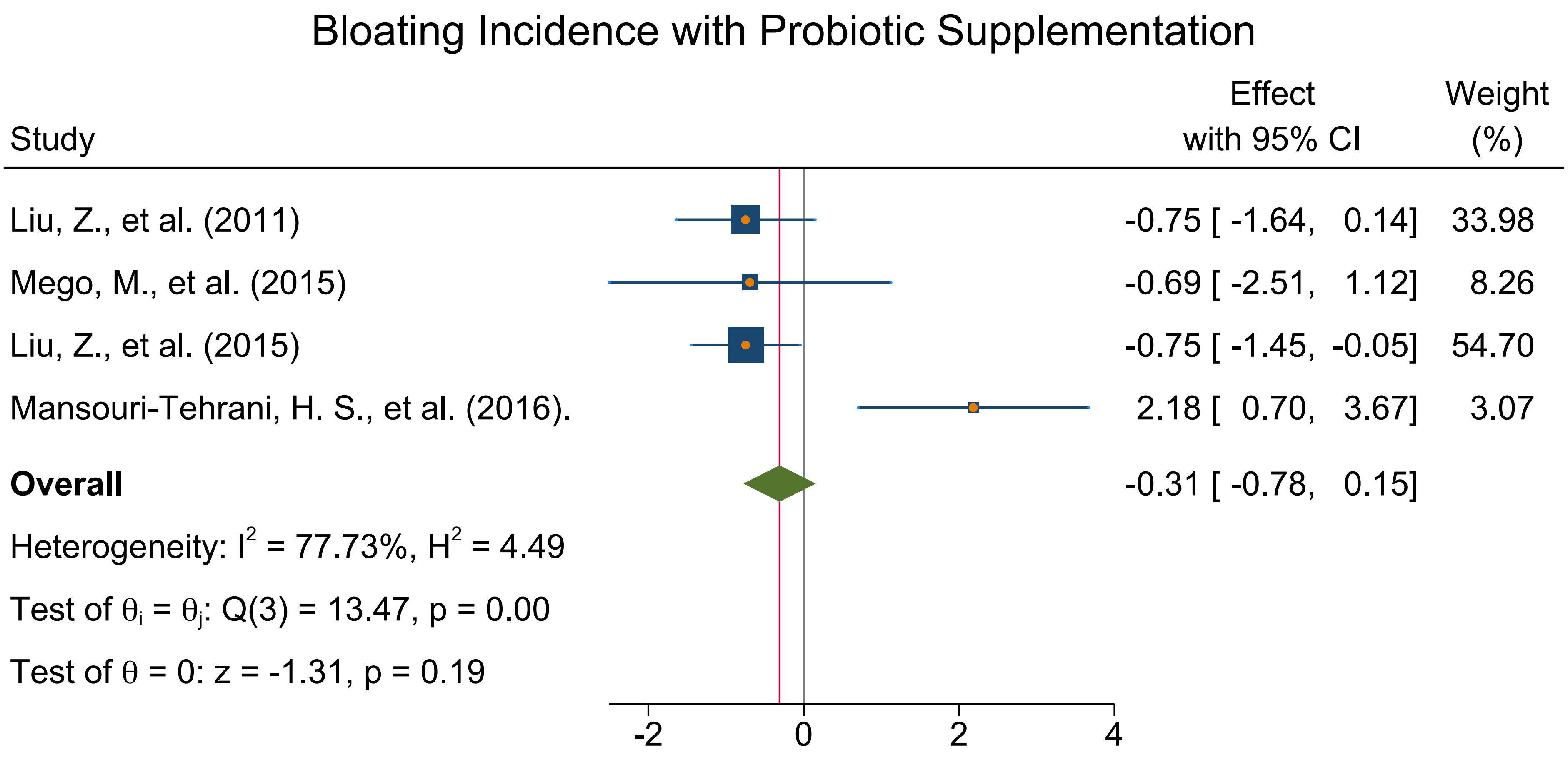
Bloating Incidence with Probiotic Supplementation
Study
Effect with 95% CI
Weight (%)
Liu, Z., et al. (2011) -0.75 [-1.64, 0.14] 33.98
Mego, M., et al. (2015) -0.69 [-2.51, 1.12] 8.26
Liu, Z., et al. (2015) -0.75 [-1.45, -0.05] 54.70
Mansouri-Tehrani, H. S., et al. (2016). 2.18 [0.70, 3.67] 3.07
Overall -0.31 [-0.78, 0.15]
Heterogeneity: I^2 = 77.73%, H^2 = 4.49
Test of $\theta_i = \theta_j$: Q(3) = 13.47, p = 0.00
Test of θ = 0: z = -1.31, p = 0.19
-2
0
2
4

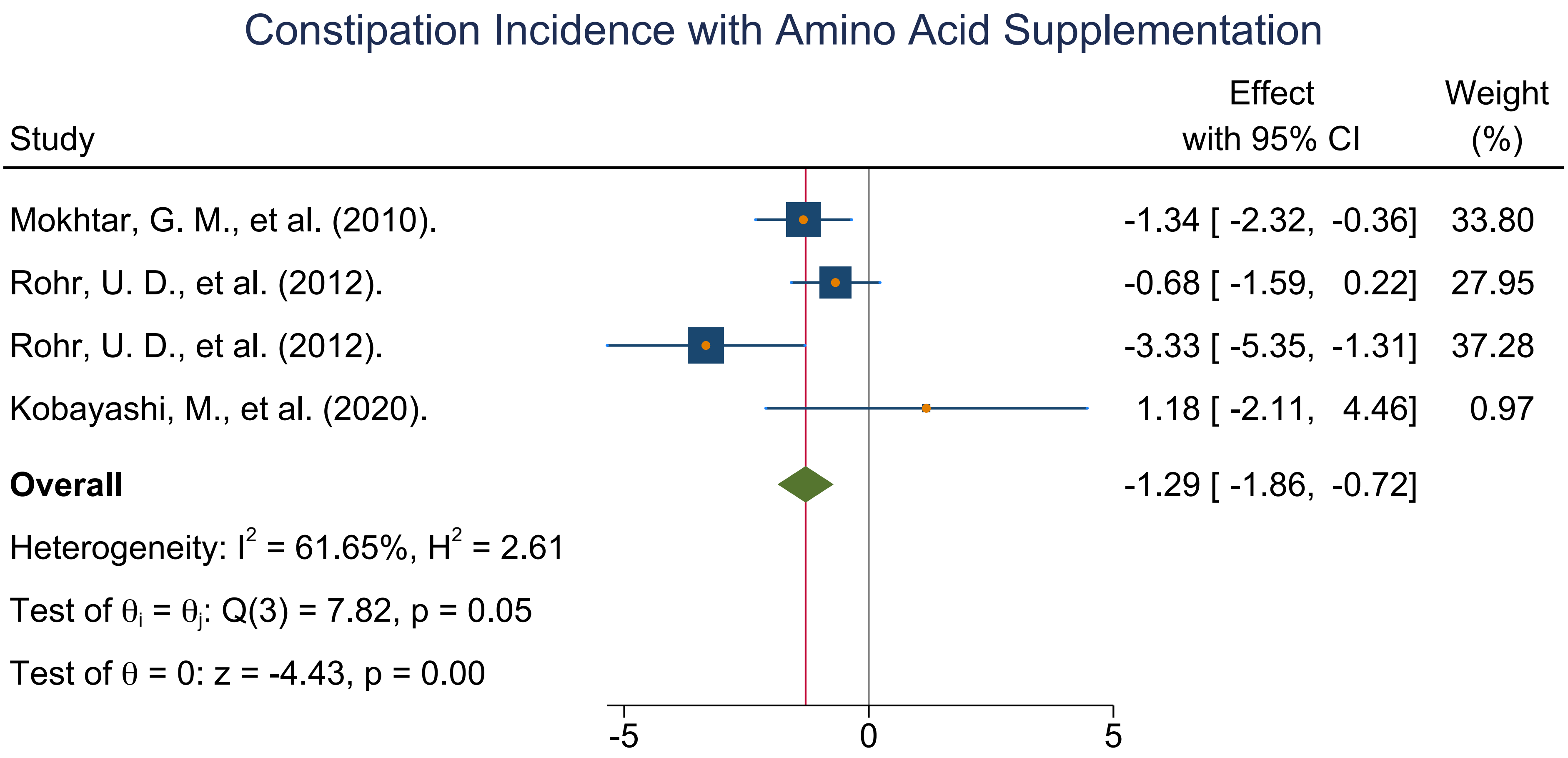
Constipation Incidence with Amino Acid Supplementation
Study
Effect with 95% CI
Weight (%)
Mokhtar, G. M., et al. (2010).
-1.34 [-2.32, -0.36]
33.80
Rohr, U. D., et al. (2012).
-0.68 [-1.59, 0.22]
27.95
Rohr, U. D., et al. (2012).
-3.33 [-5.35, -1.31]
37.28
Kobayashi, M., et al. (2020).
1.18 [-2.11, 4.46]
0.97
Overall
-1.29 [-1.86, -0.72]
Heterogeneity: I2 = 61.65%, H2 = 2.61
Test of θi = θj: Q(3) = 7.82, p = 0.05
Test of θ = 0: z = -4.43, p = 0.00
-5
0
5

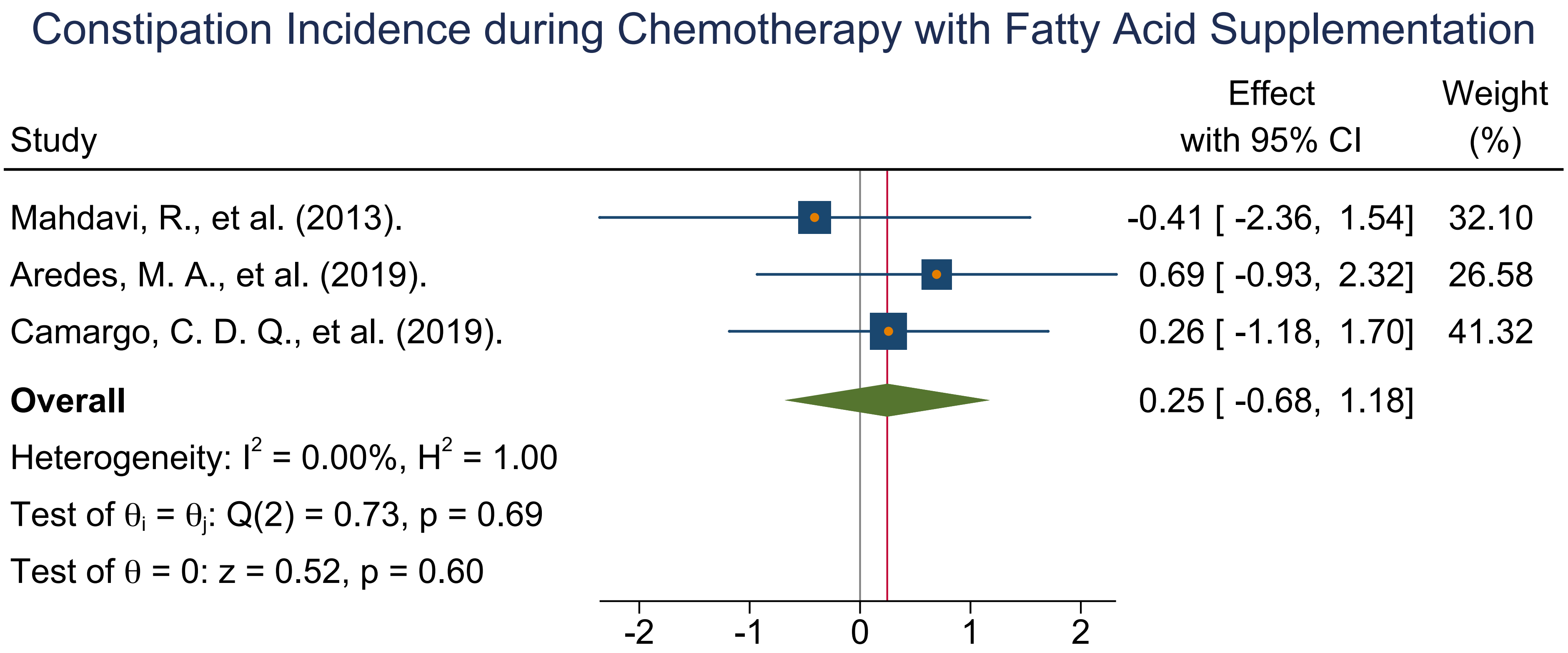
Constipation Incidence during Chemotherapy with Fatty Acid Supplementation
Study
Effect with 95% CI
Weight (%)
Mahdavi, R., et al. (2013).
-0.41 [-2.36, 1.54]
32.10
Aredes, M. A., et al. (2019).
0.69 [-0.93, 2.32]
26.58
Camargo, C. D. Q., et al. (2019).
0.26 [-1.18, 1.70]
41.32
Overall
0.25 [-0.68, 1.18]
Heterogeneity: I2 = 0.00%, H2 = 1.00
Test of θi = θj: Q(2) = 0.73, p = 0.69
Test of θ = 0: z = 0.52, p = 0.60
-2
-1
0
1
2

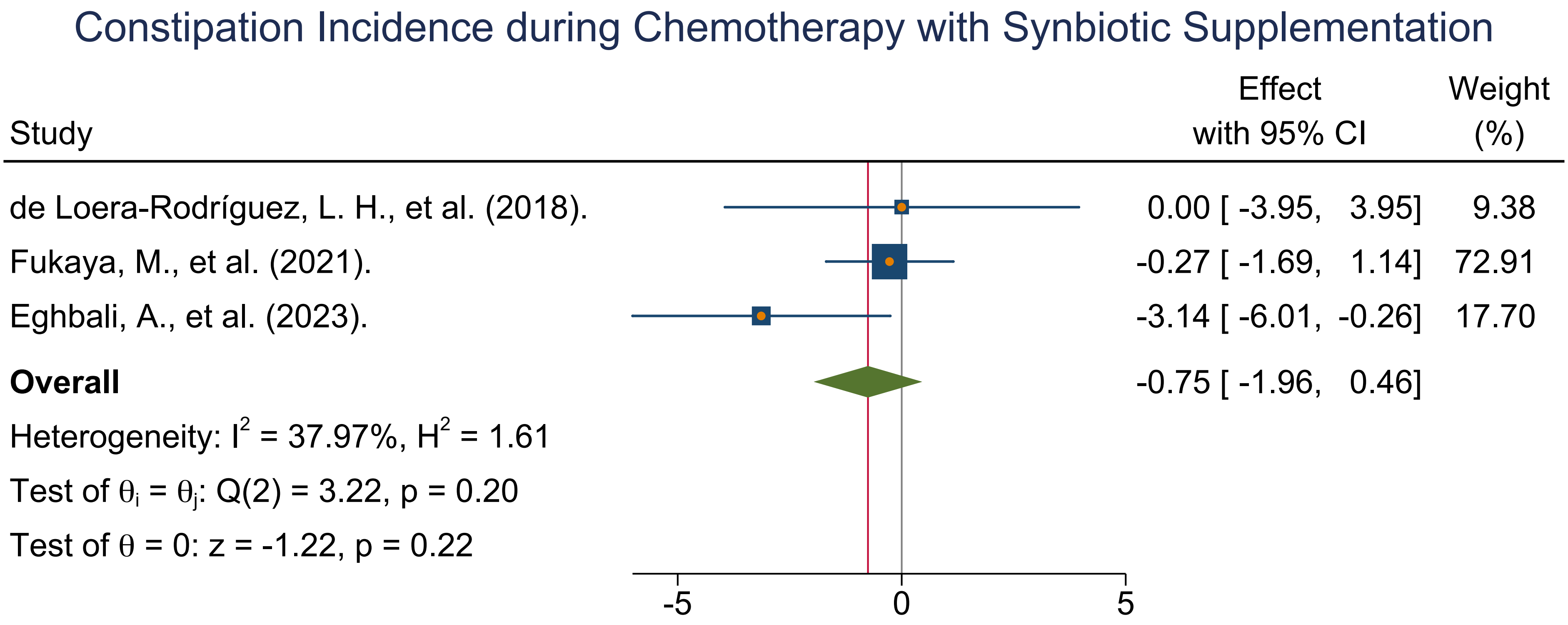
Constipation Incidence during Chemotherapy with Synbiotic Supplementation
Study
Effect with 95% CI
Weight (%)
de Loera-Rodríguez, L. H., et al. (2018).
0.00 [-3.95, 3.95] 9.38
Fukaya, M., et al. (2021).
-0.27 [-1.69, 1.14] 72.91
Eghbali, A., et al. (2023).
-3.14 [-6.01, -0.26] 17.70
Overall
-0.75 [-1.96, 0.46]
Heterogeneity: I2 = 37.97%, H2 = 1.61
Test of θi = θj: Q(2) = 3.22, p = 0.20
Test of θ = 0: z = -1.22, p = 0.22
-5
0
5

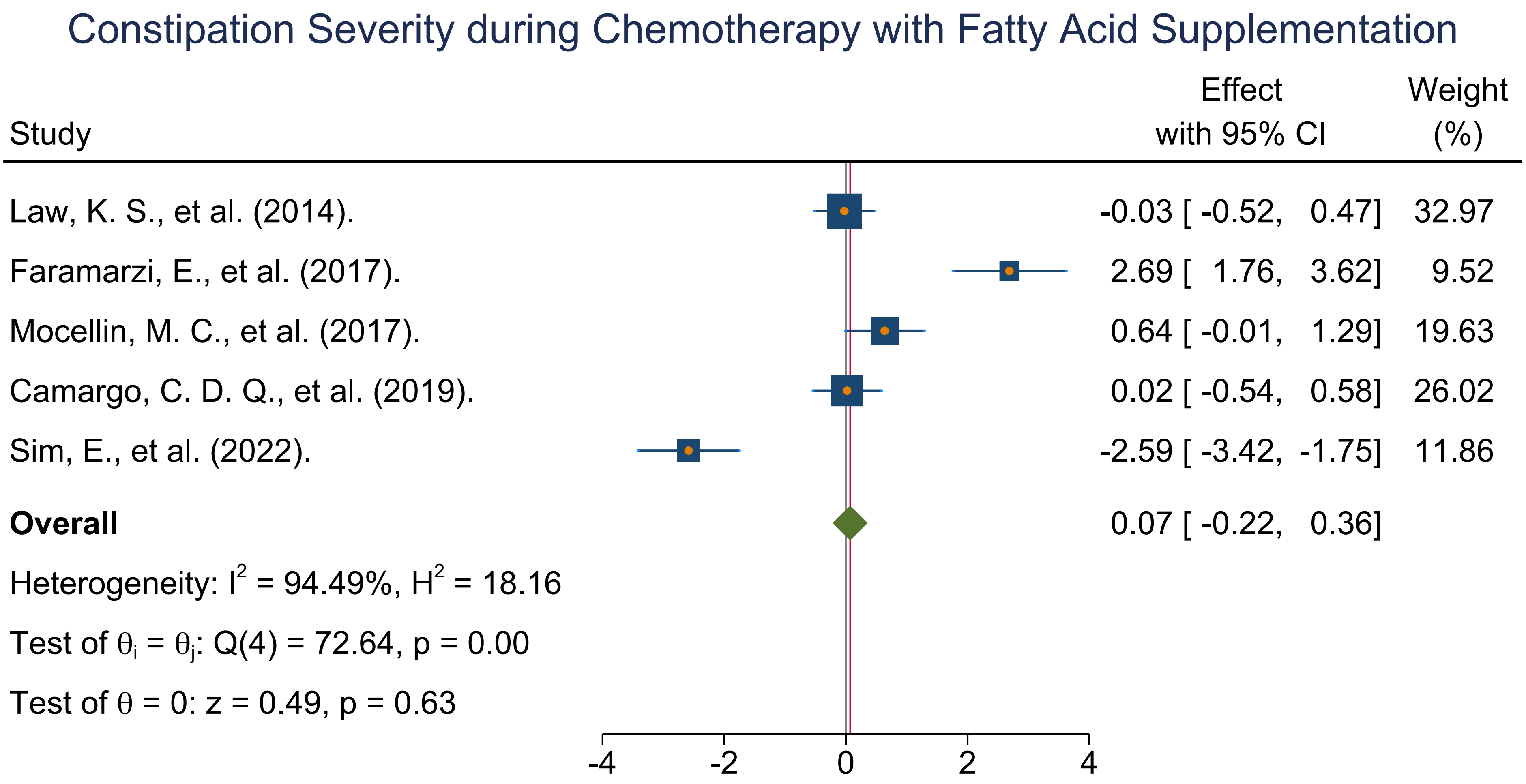
Constipation Severity during Chemotherapy with Fatty Acid Supplementation
Study
Effect with 95% CI
Weight (%)
Law, K. S., et al. (2014). -0.03 [-0.52, 0.47] 32.97
Faramarzi, E., et al. (2017). 2.69 [1.76, 3.62] 9.52
Mocellin, M. C., et al. (2017). 0.64 [-0.01, 1.29] 19.63
Camargo, C. D. Q., et al. (2019). 0.02 [-0.54, 0.58] 26.02
Sim, E., et al. (2022). -2.59 [-3.42, -1.75] 11.86
Overall 0.07 [-0.22, 0.36]
Heterogeneity: I^2 = 94.49%, H^2 = 18.16
Test of $\theta_i = \theta_j$: Q(4) = 72.64, p = 0.00
Test of $\theta = 0$: z = 0.49, p = 0.63
-4
-2
0
2
4

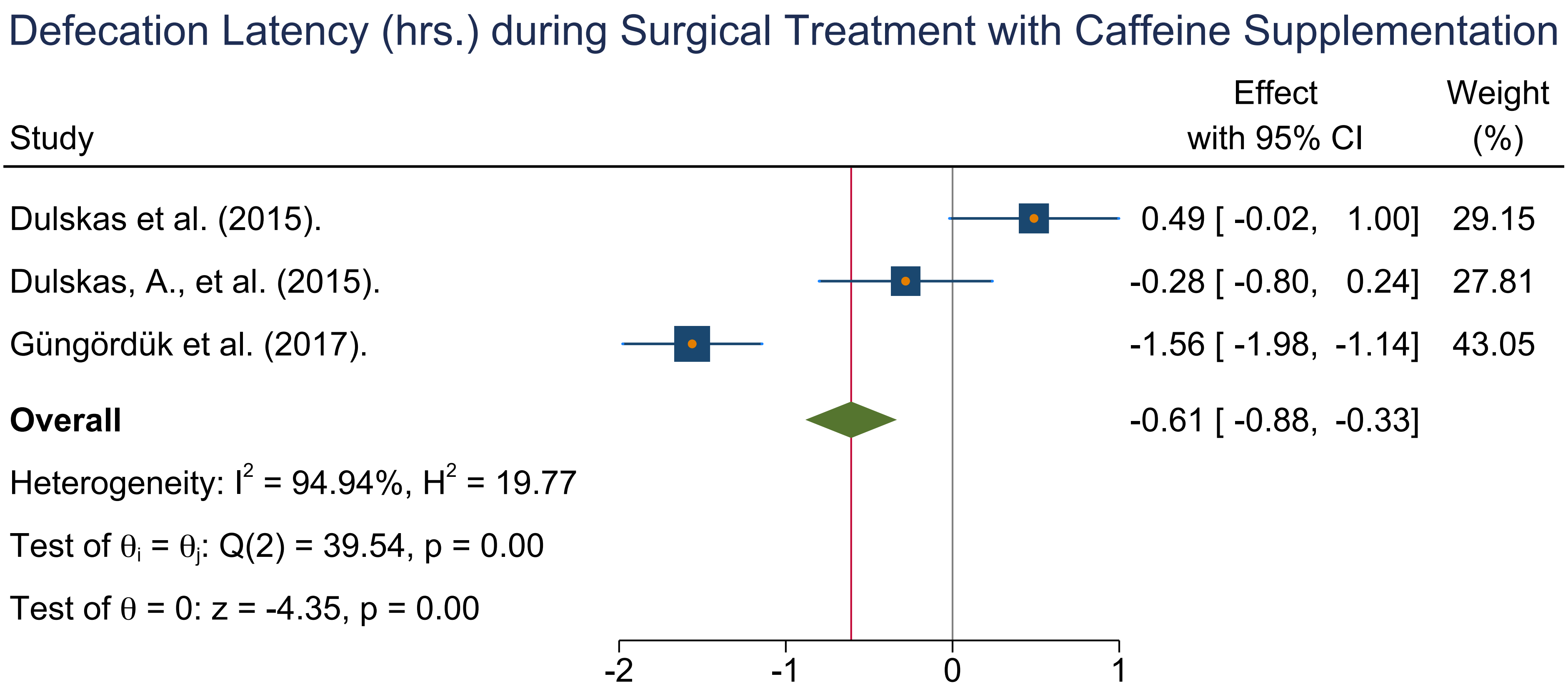
Defecation Latency (hrs.) during Surgical Treatment with Caffeine Supplementation
Study
Effect with 95% CI
Weight (%)
Dulskas et al. (2015).
0.49 [-0.02, 1.00]
29.15
Dulskas, A., et al. (2015).
-0.28 [-0.80, 0.24]
27.81
Güngördük et al. (2017).
-1.56 [-1.98, -1.14]
43.05
Overall
-0.61 [-0.88, -0.33]
Heterogeneity: I^2 = 94.94%, H^2 = 19.77
Test of $\theta_i = \theta_j$: Q(2) = 39.54, p = 0.00
Test of θ = 0: z = -4.35, p = 0.00
-2
-1
0
1

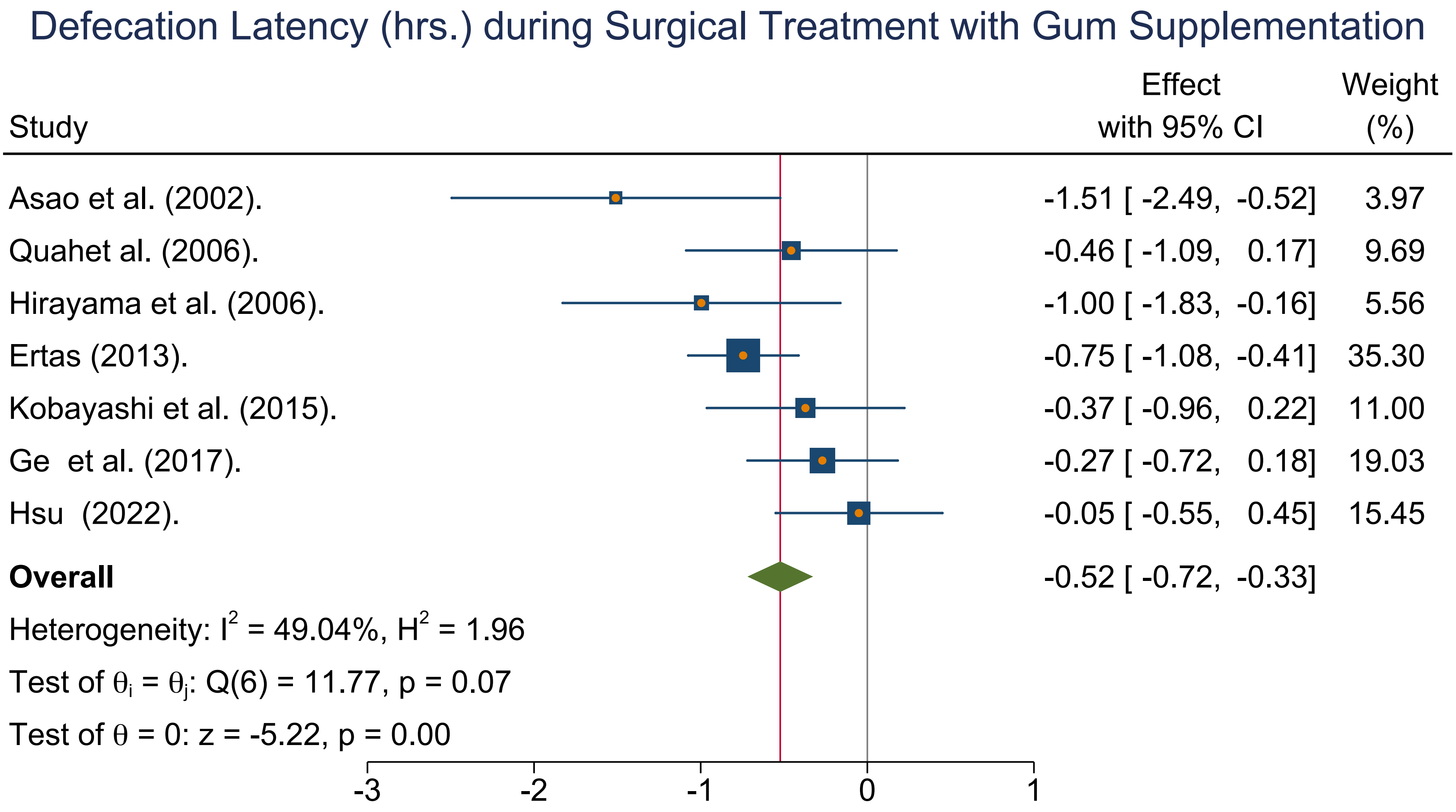
Defecation Latency (hrs.) during Surgical Treatment with Gum Supplementation
Study
Effect with 95% CI
Weight (%)
Asao et al. (2002). -1.51 [-2.49, -0.52] 3.97
Quahet al. (2006). -0.46 [-1.09, 0.17] 9.69
Hirayama et al. (2006). -1.00 [-1.83, -0.16] 5.56
Ertas (2013). -0.75 [-1.08, -0.41] 35.30
Kobayashi et al. (2015). -0.37 [-0.96, 0.22] 11.00
Ge et al. (2017). -0.27 [-0.72, 0.18] 19.03
Hsu (2022). -0.05 [-0.55, 0.45] 15.45
Overall -0.52 [-0.72, -0.33]
Heterogeneity: I^2 = 49.04%, H^2 = 1.96
Test of $\theta_i = \theta_j$: Q(6) = 11.77, p = 0.07
Test of θ = 0: z = -5.22, p = 0.00
-3
-2
-1
0
1

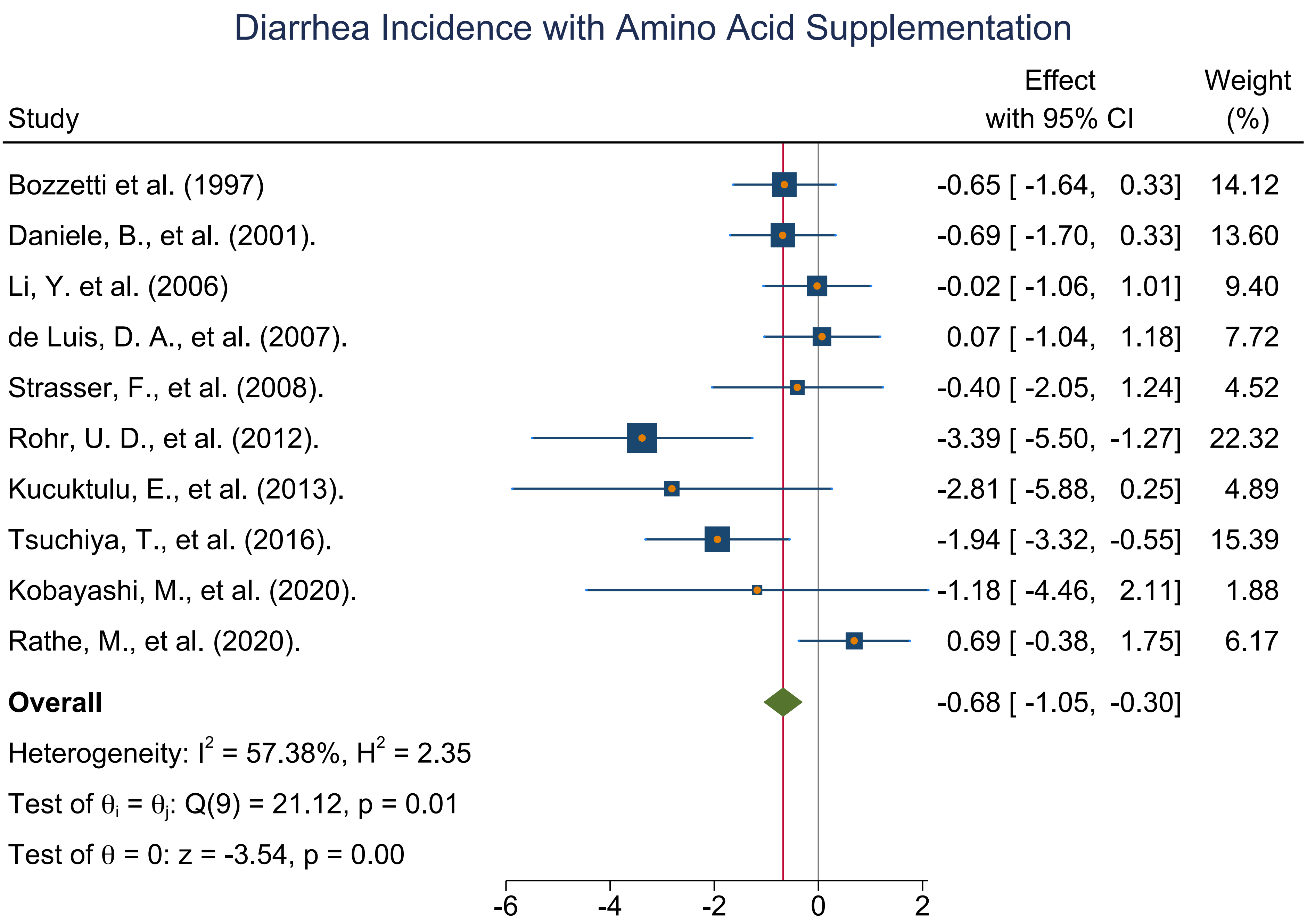

Diarrhea Incidence with Amino Acid Supplementation
Study
Effect with 95% CI
Weight (%)
Bozzetti et al. (1997) -0.65 [-1.64, 0.33] 14.12
Daniele, B., et al. (2001). -0.69 [-1.70, 0.33] 13.60
Li, Y. et al. (2006) -0.02 [-1.06, 1.01] 9.40
de Luis, D. A., et al. (2007). 0.07 [-1.04, 1.18] 7.72
Strasser, F., et al. (2008). -0.40 [-2.05, 1.24] 4.52
Rohr, U. D., et al. (2012). -3.39 [-5.50, -1.27] 22.32
Kucuktulu, E., et al. (2013). -2.81 [-5.88, 0.25] 4.89
Tsuchiya, T., et al. (2016). -1.94 [-3.32, -0.55] 15.39
Kobayashi, M., et al. (2020). -1.18 [-4.46, 2.11] 1.88
Rathe, M., et al. (2020). 0.69 [-0.38, 1.75] 6.17
Overall -0.68 [-1.05, -0.30]
Heterogeneity: I^2 = 57.38%, H^2 = 2.35
Test of $\theta_i = \theta_j$: Q(9) = 21.12, p = 0.01
Test of θ = 0: z = -3.54, p = 0.00
-6
-4
-2
0
2

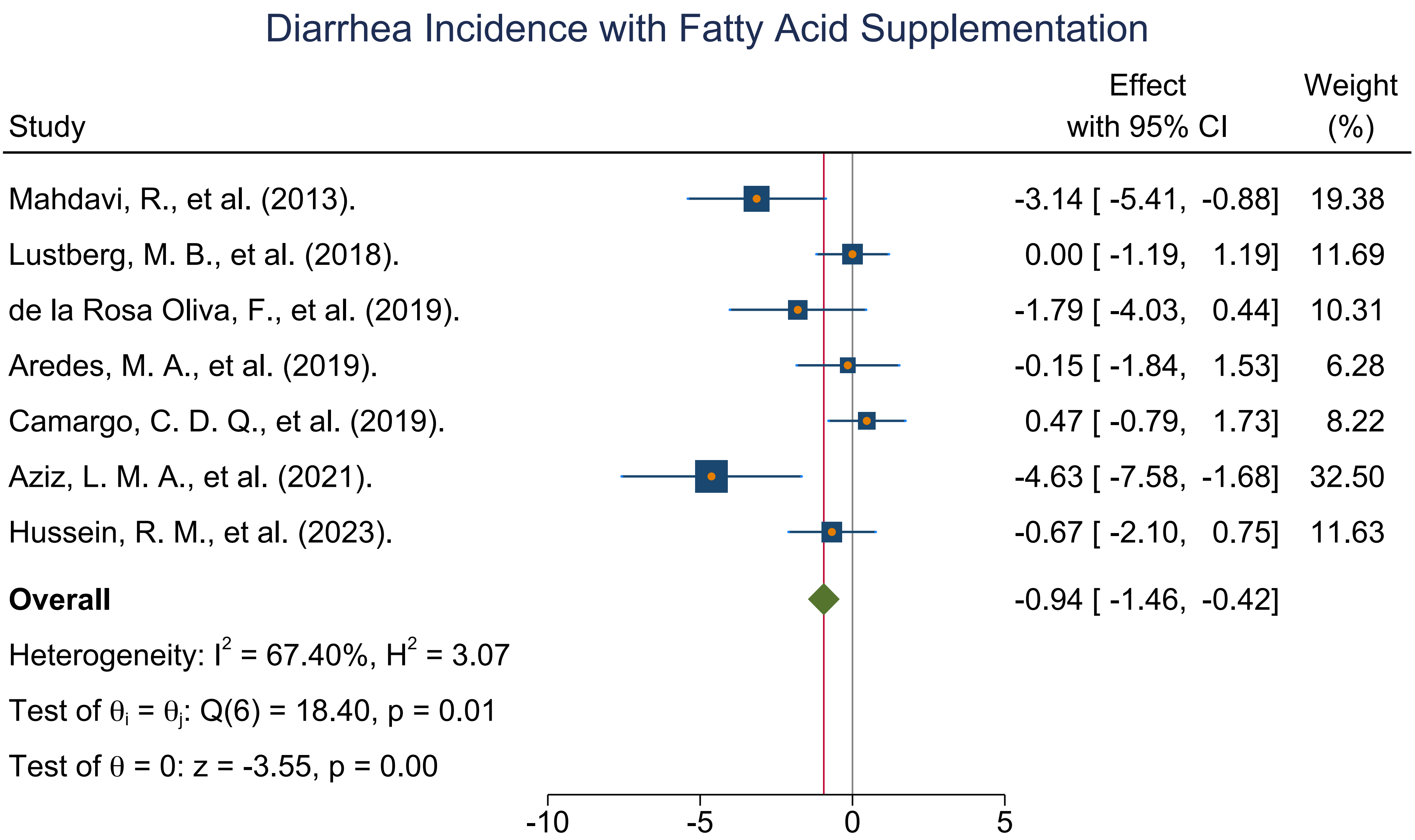
Diarrhea Incidence with Fatty Acid Supplementation
Study
Effect with 95% CI
Weight (%)
Mahdavi, R., et al. (2013). -3.14 [-5.41, -0.88] 19.38
Lustberg, M. B., et al. (2018). 0.00 [-1.19, 1.19] 11.69
de la Rosa Oliva, F., et al. (2019). -1.79 [-4.03, 0.44] 10.31
Aredes, M. A., et al. (2019). -0.15 [-1.84, 1.53] 6.28
Camargo, C. D. Q., et al. (2019). 0.47 [-0.79, 1.73] 8.22
Aziz, L. M. A., et al. (2021). -4.63 [-7.58, -1.68] 32.50
Hussein, R. M., et al. (2023). -0.67 [-2.10, 0.75] 11.63
Overall -0.94 [-1.46, -0.42]
Heterogeneity: I^2 = 67.40%, H^2 = 3.07
Test of $\theta_i = \theta_j$: Q(6) = 18.40, p = 0.01
Test of θ = 0: z = -3.55, p = 0.00
-10
-5
0
5

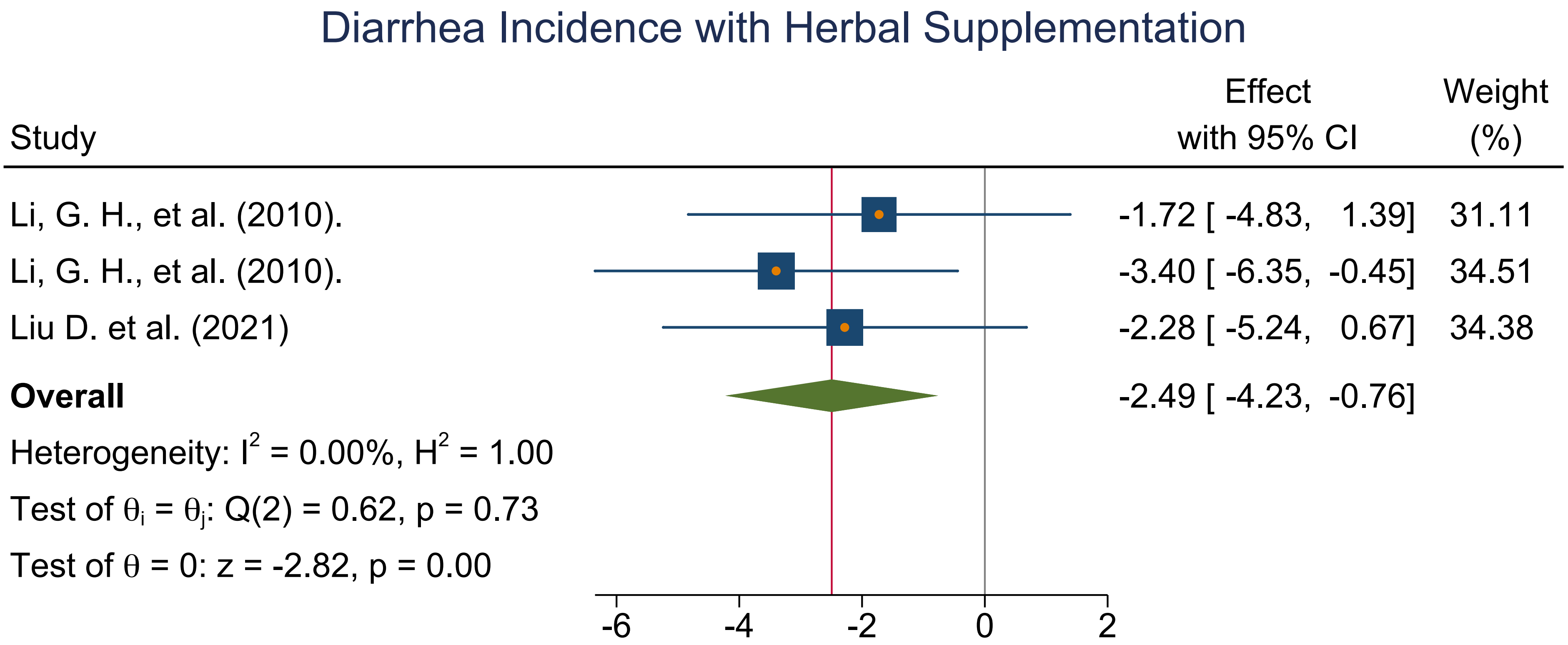

Diarrhea Incidence with Herbal Supplementation
Study
Effect with 95% CI
Weight (%)
Li, G. H., et al. (2010). -1.72 [-4.83, 1.39] 31.11
Li, G. H., et al. (2010). -3.40 [-6.35, -0.45] 34.51
Liu D. et al. (2021) -2.28 [-5.24, 0.67] 34.38
Overall -2.49 [-4.23, -0.76]
Heterogeneity: I^2 = 0.00%, H^2 = 1.00
Test of $\theta_i = \theta_j$: Q(2) = 0.62, p = 0.73
Test of θ = 0: z = -2.82, p = 0.00
-6
-4
-2
0
2

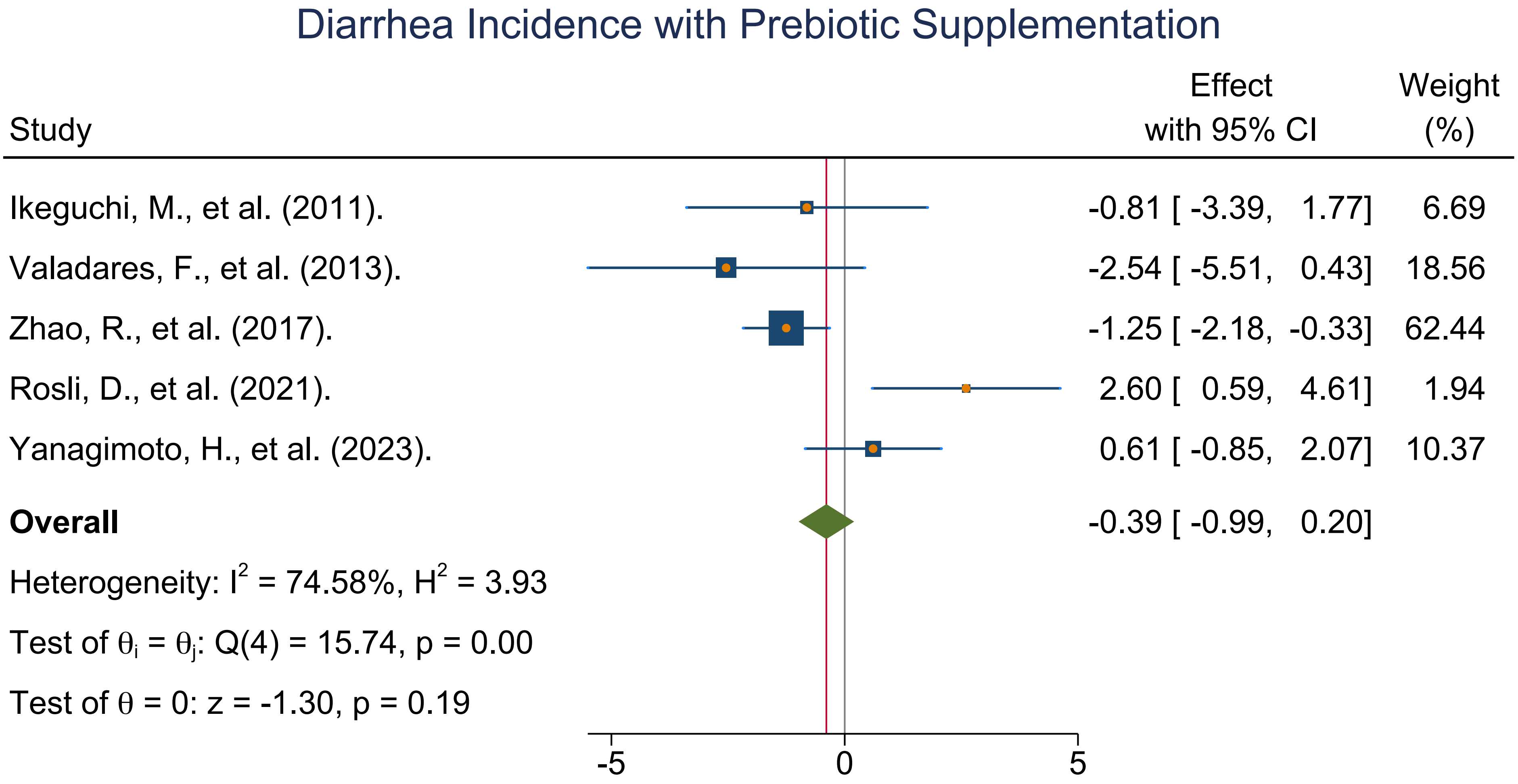
Diarrhea Incidence with Prebiotic Supplementation
Study
Effect with 95% CI
Weight (%)
Ikeguchi, M., et al. (2011). -0.81 [-3.39, 1.77] 6.69
Valadares, F., et al. (2013). -2.54 [-5.51, 0.43] 18.56
Zhao, R., et al. (2017). -1.25 [-2.18, -0.33] 62.44
Rosli, D., et al. (2021). 2.60 [0.59, 4.61] 1.94
Yanagimoto, H., et al. (2023). 0.61 [-0.85, 2.07] 10.37
Overall -0.39 [-0.99, 0.20]
Heterogeneity: I^2 = 74.58%, H^2 = 3.93
Test of $\theta_i = \theta_j$: Q(4) = 15.74, p = 0.00
Test of $\theta = 0$: z = -1.30, p = 0.19
-5
0
5

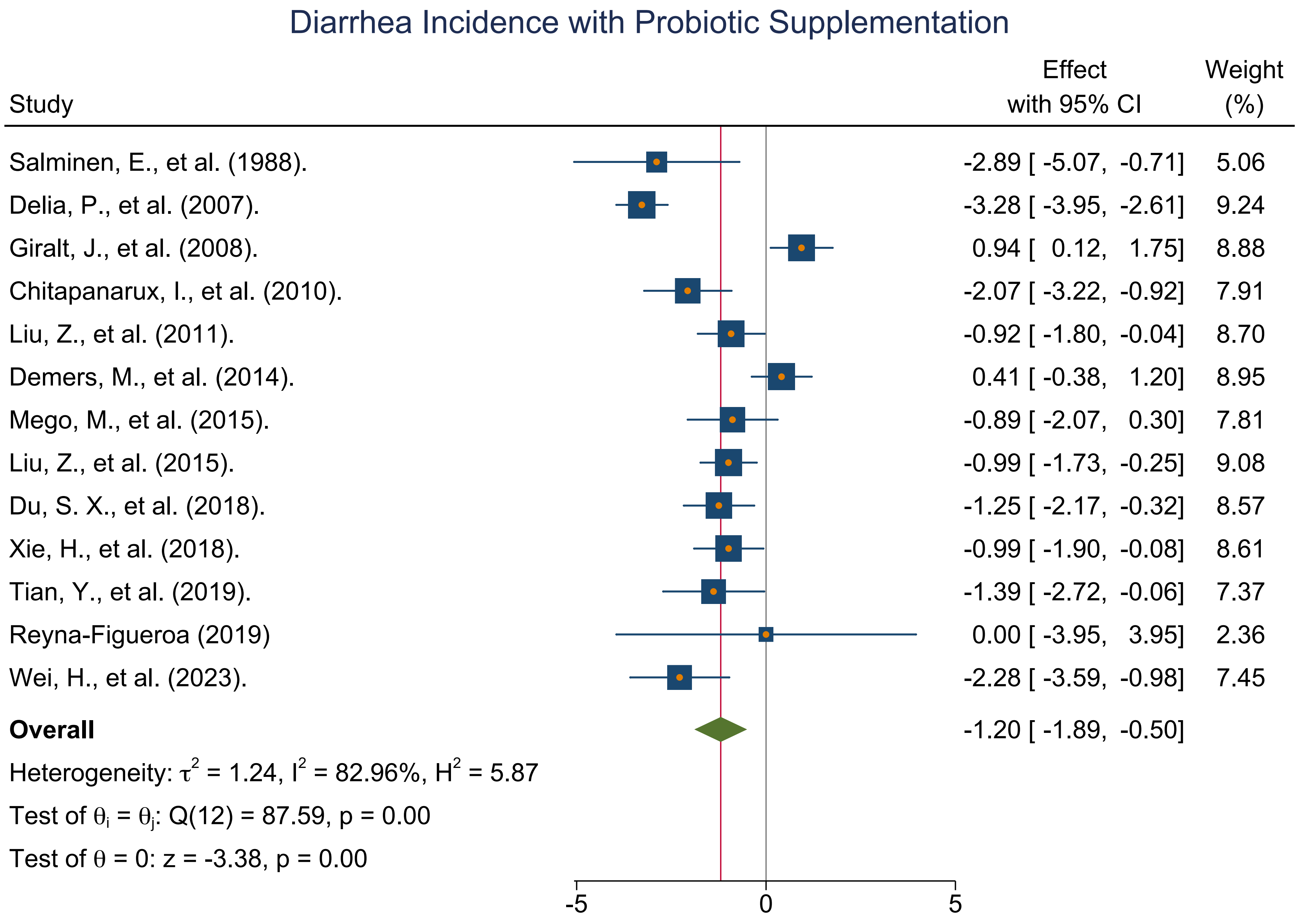
Diarrhea Incidence with Probiotic Supplementation
Study
Effect with 95% CI
Weight (%)
Salminen, E., et al. (1988). -2.89 [-5.07, -0.71] 5.06
Delia, P., et al. (2007). -3.28 [-3.95, -2.61] 9.24
Giralt, J., et al. (2008). 0.94 [0.12, 1.75] 8.88
Chitapanarux, I., et al. (2010). -2.07 [-3.22, -0.92] 7.91
Liu, Z., et al. (2011). -0.92 [-1.80, -0.04] 8.70
Demers, M., et al. (2014). 0.41 [-0.38, 1.20] 8.95
Mego, M., et al. (2015). -0.89 [-2.07, 0.30] 7.81
Liu, Z., et al. (2015). -0.99 [-1.73, -0.25] 9.08
Du, S. X., et al. (2018). -1.25 [-2.17, -0.32] 8.57
Xie, H., et al. (2018). -0.99 [-1.90, -0.08] 8.61
Tian, Y., et al. (2019). -1.39 [-2.72, -0.06] 7.37
Reyna-Figueroa (2019) 0.00 [-3.95, 3.95] 2.36
Wei, H., et al. (2023). -2.28 [-3.59, -0.98] 7.45
Overall -1.20 [-1.89, -0.50]
Heterogeneity: τ^2 = 1.24, I^2 = 82.96%, H^2 = 5.87
Test of $\theta_i = \theta_j$: Q(12) = 87.59, p = 0.00
Test of θ = 0: z = -3.38, p = 0.00
-5
0
5

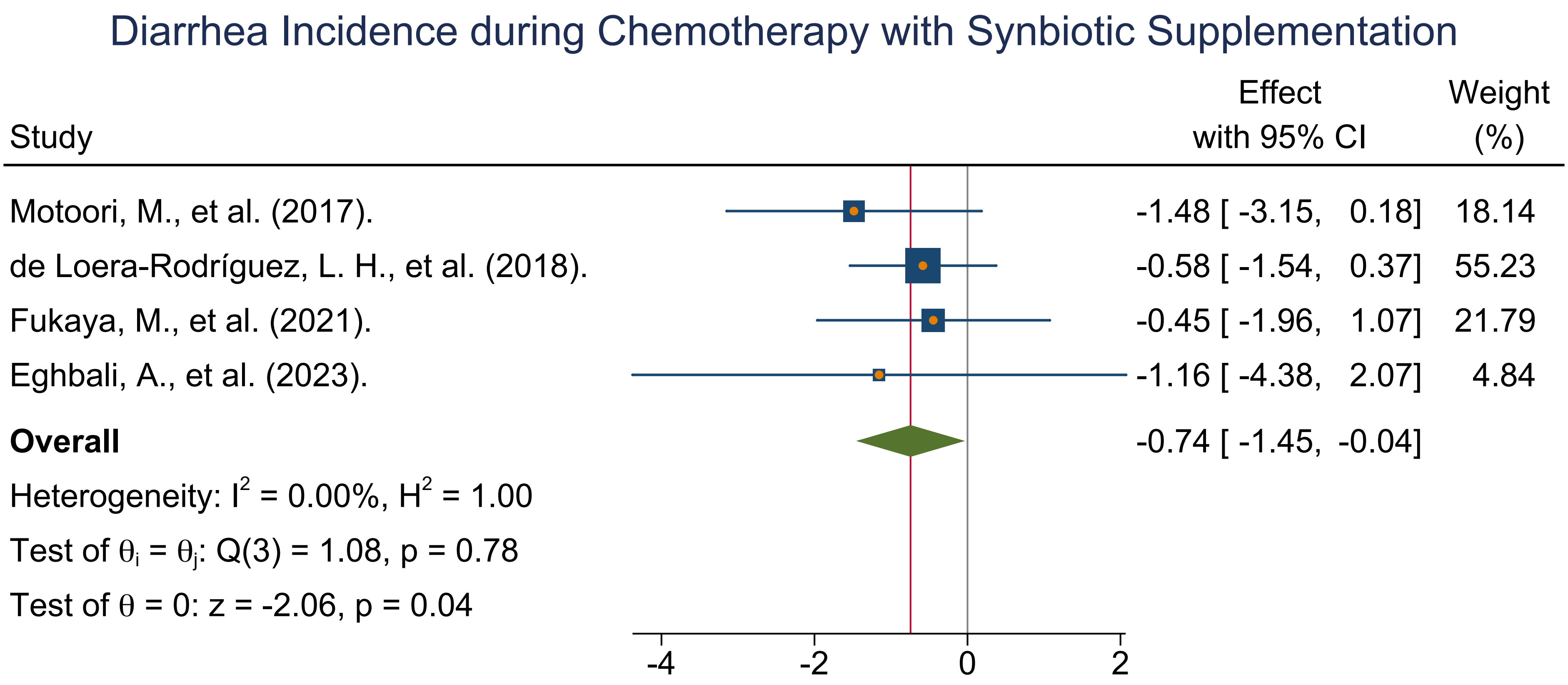
Diarrhea Incidence during Chemotherapy with Synbiotic Supplementation
Study
Effect with 95% CI
Weight (%)
Motoori, M., et al. (2017). -1.48 [-3.15, 0.18] 18.14
de Loera-Rodríguez, L. H., et al. (2018). -0.58 [-1.54, 0.37] 55.23
Fukaya, M., et al. (2021). -0.45 [-1.96, 1.07] 21.79
Eghbali, A., et al. (2023). -1.16 [-4.38, 2.07] 4.84
Overall -0.74 [-1.45, -0.04]
Heterogeneity: I^2 = 0.00%, H^2 = 1.00
Test of $\theta_i = \theta_j$: Q(3) = 1.08, p = 0.78
Test of θ = 0: z = -2.06, p = 0.04
-4
-2
0
2

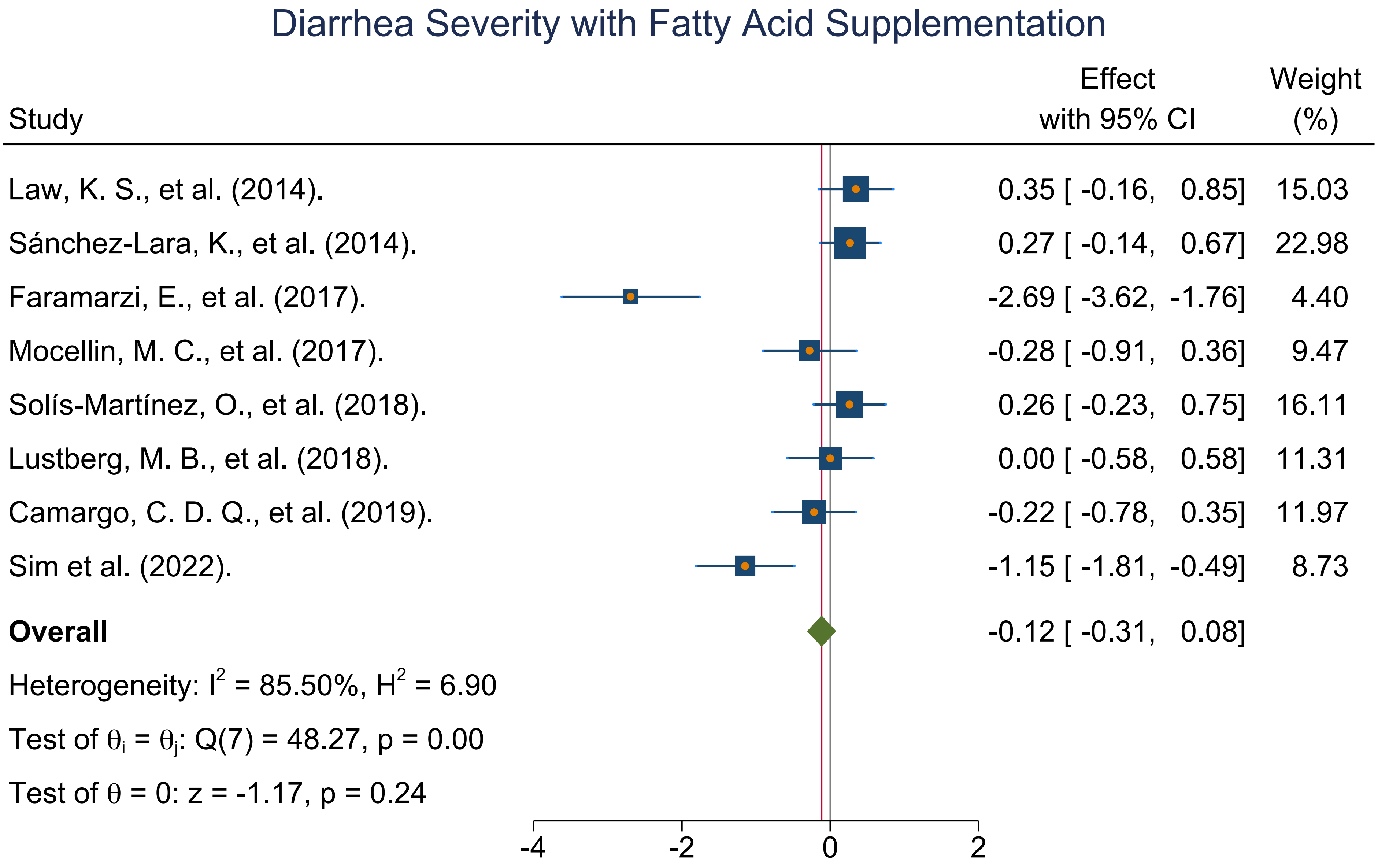
Diarrhea Severity with Fatty Acid Supplementation
Study
Effect with 95% CI
Weight (%)
Law, K. S., et al. (2014). 0.35 [-0.16, 0.85] 15.03
Sánchez-Lara, K., et al. (2014). 0.27 [-0.14, 0.67] 22.98
Faramarzi, E., et al. (2017). -2.69 [-3.62, -1.76] 4.40
Mocellin, M. C., et al. (2017). -0.28 [-0.91, 0.36] 9.47
Solís-Martínez, O., et al. (2018). 0.26 [-0.23, 0.75] 16.11
Lustberg, M. B., et al. (2018). 0.00 [-0.58, 0.58] 11.31
Camargo, C. D. Q., et al. (2019). -0.22 [-0.78, 0.35] 11.97
Sim et al. (2022). -1.15 [-1.81, -0.49] 8.73
Overall -0.12 [-0.31, 0.08]
Heterogeneity: I^2 = 85.50%, H^2 = 6.90
Test of $\theta_i = \theta_j$: Q(7) = 48.27, p = 0.00
Test of θ = 0: z = -1.17, p = 0.24
-4
-2
0
2

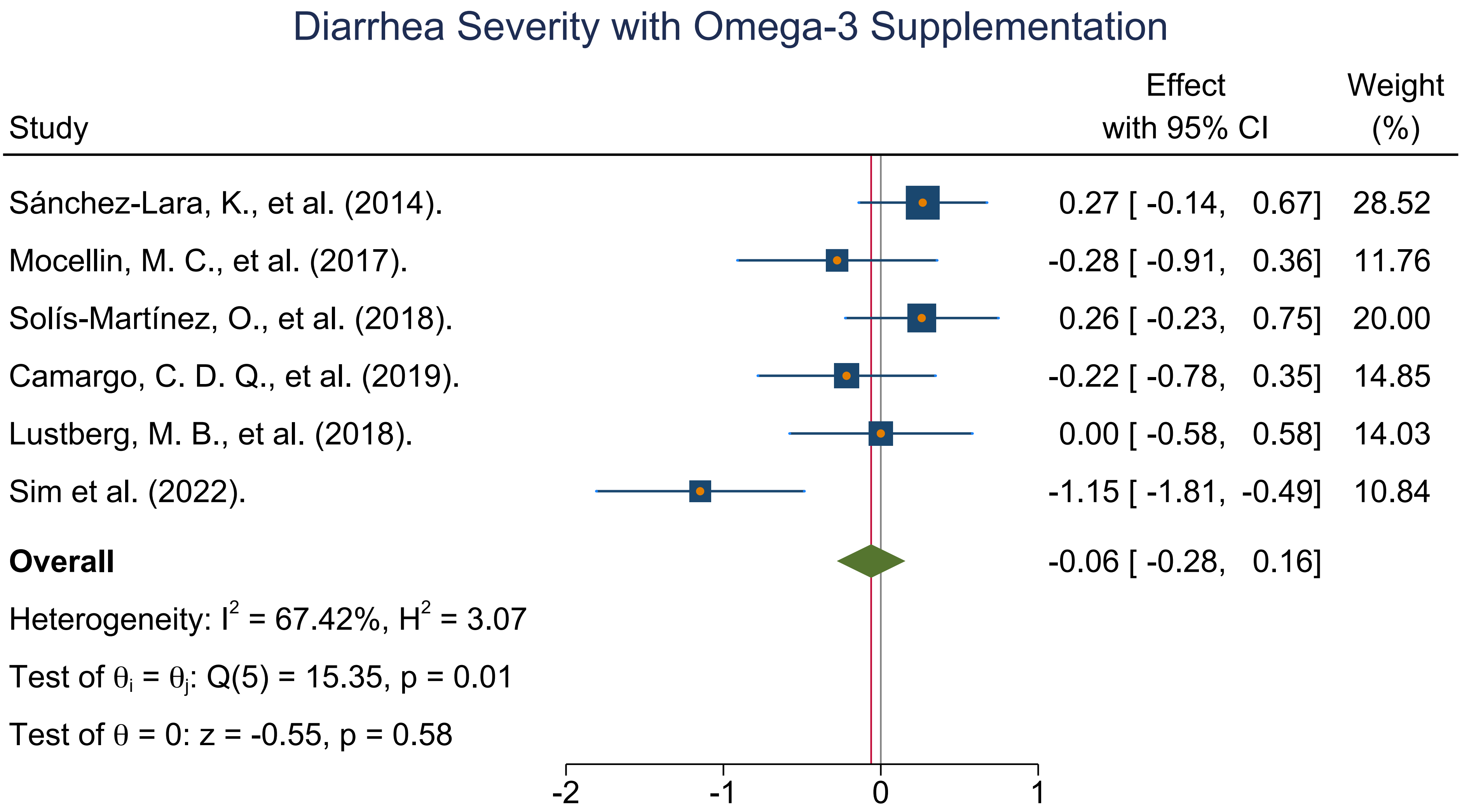
Diarrhea Severity with Omega-3 Supplementation
Study
Effect with 95% CI
Weight (%)
Sánchez-Lara, K., et al. (2014). 0.27 [-0.14, 0.67] 28.52
Mocellin, M. C., et al. (2017). -0.28 [-0.91, 0.36] 11.76
Solís-Martínez, O., et al. (2018). 0.26 [-0.23, 0.75] 20.00
Camargo, C. D. Q., et al. (2019). -0.22 [-0.78, 0.35] 14.85
Lustberg, M. B., et al. (2018). 0.00 [-0.58, 0.58] 14.03
Sim et al. (2022). -1.15 [-1.81, -0.49] 10.84
Overall -0.06 [-0.28, 0.16]
Heterogeneity: I^2 = 67.42%, H^2 = 3.07
Test of $\theta_i = \theta_j$: Q(5) = 15.35, p = 0.01
Test of θ = 0: z = -0.55, p = 0.58
-2
-1
0
1

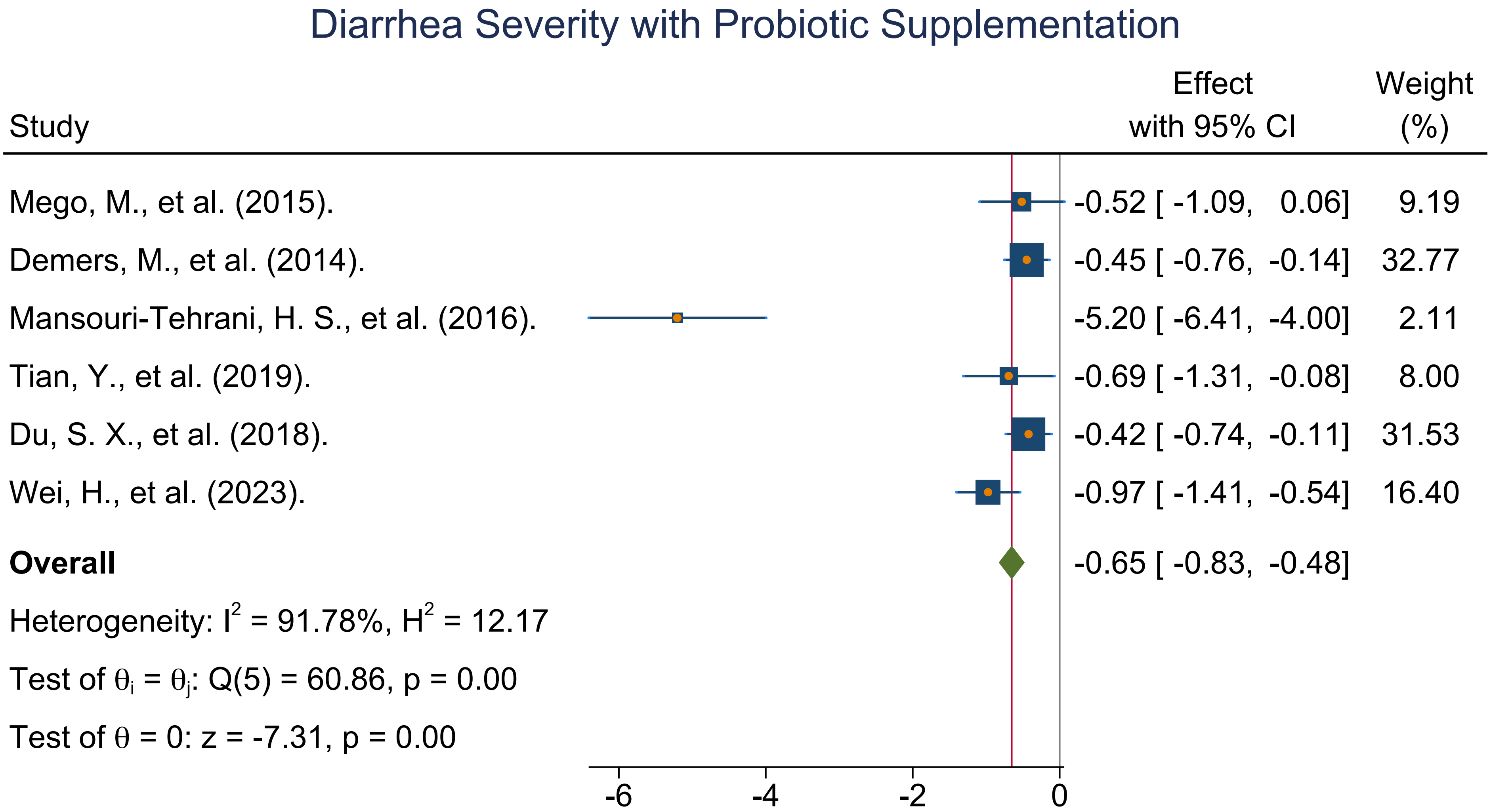

Diarrhea Severity with Probiotic Supplementation
Study
Effect
with 95% CI
Weight
(%)
Mego, M., et al. (2015).
-0.52 [-1.09, 0.06]
9.19
Demers, M., et al. (2014).
-0.45 [-0.76, -0.14]
32.77
Mansouri-Tehrani, H. S., et al. (2016).
-5.20 [-6.41, -4.00]
2.11
Tian, Y., et al. (2019).
-0.69 [-1.31, -0.08]
8.00
Du, S. X., et al. (2018).
-0.42 [-0.74, -0.11]
31.53
Wei, H., et al. (2023).
-0.97 [-1.41, -0.54]
16.40
Overall
-0.65 [-0.83, -0.48]
Heterogeneity: I^2 = 91.78%, H^2 = 12.17
Test of $\theta_i = \theta_j$: Q(5) = 60.86, p = 0.00
Test of θ = 0: z = -7.31, p = 0.00
-6
-4
-2
0

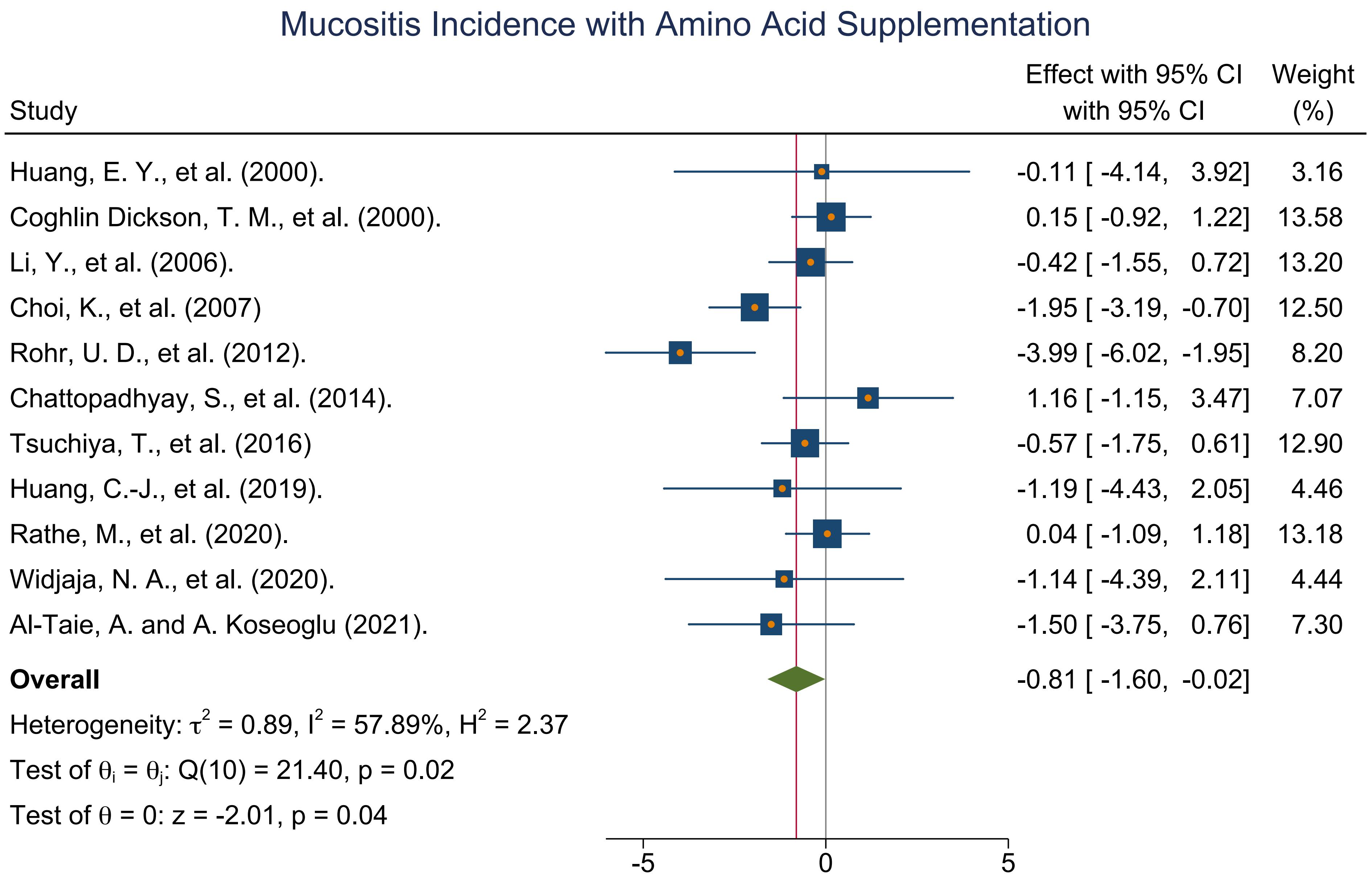

Mucositis Incidence with Amino Acid Supplementation
Study
Effect with 95% CI
with 95% CI
Weight (%)
Huang, E. Y., et al. (2000). -0.11 [-4.14, 3.92] 3.16
Coghlin Dickson, T. M., et al. (2000). 0.15 [-0.92, 1.22] 13.58
Li, Y., et al. (2006). -0.42 [-1.55, 0.72] 13.20
Choi, K., et al. (2007) -1.95 [-3.19, -0.70] 12.50
Rohr, U. D., et al. (2012). -3.99 [-6.02, -1.95] 8.20
Chattopadhyay, S., et al. (2014). 1.16 [-1.15, 3.47] 7.07
Tsuchiya, T., et al. (2016) -0.57 [-1.75, 0.61] 12.90
Huang, C.-J., et al. (2019). -1.19 [-4.43, 2.05] 4.46
Rathe, M., et al. (2020). 0.04 [-1.09, 1.18] 13.18
Widjaja, N. A., et al. (2020). -1.14 [-4.39, 2.11] 4.44
Al-Taie, A. and A. Koseoglu (2021). -1.50 [-3.75, 0.76] 7.30
Overall -0.81 [-1.60, -0.02]
Heterogeneity: τ² = 0.89, I² = 57.89%, H² = 2.37
Test of θi = θj: Q(10) = 21.40, p = 0.02
Test of θ = 0: z = -2.01, p = 0.04
-5
0
5

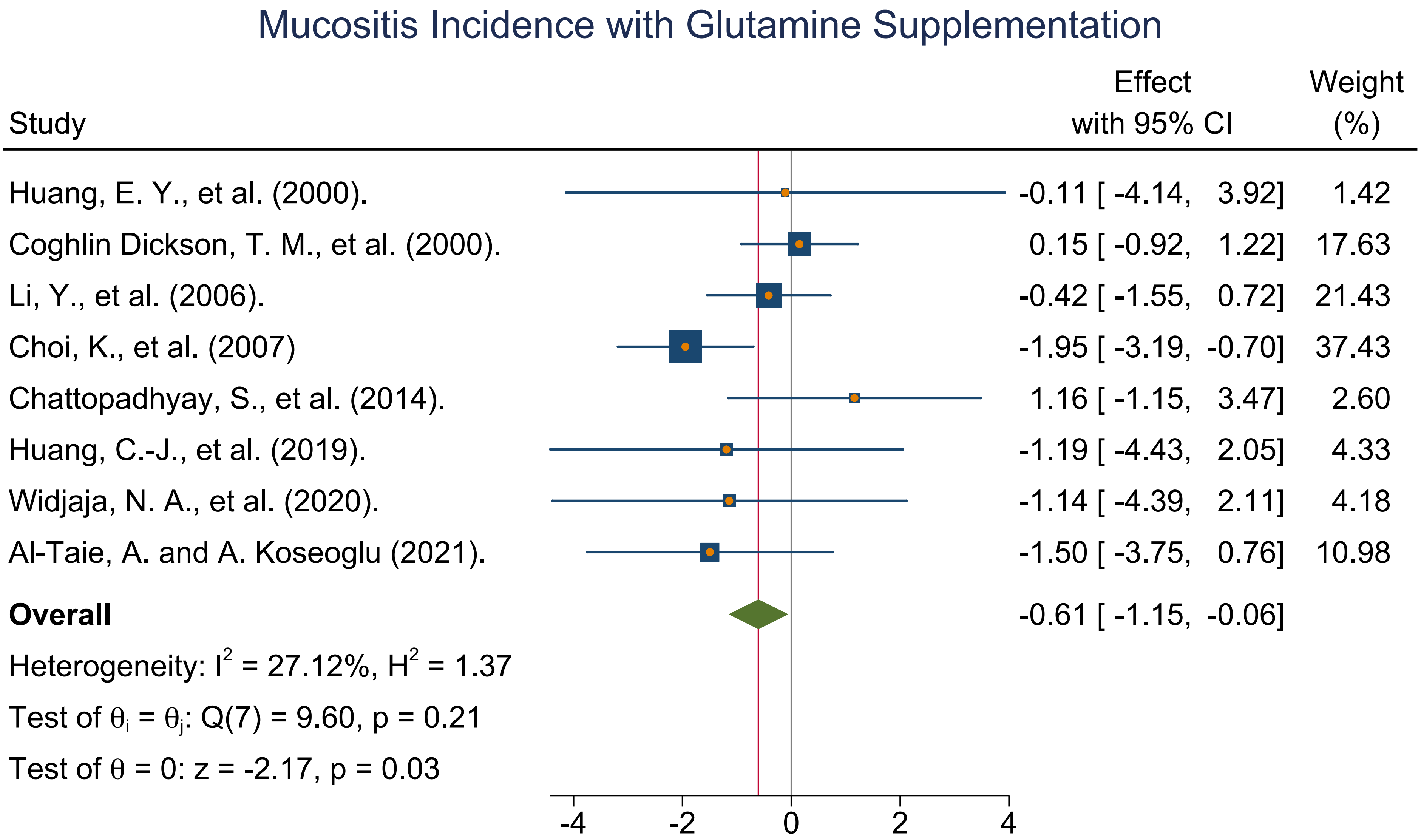
Mucositis Incidence with Glutamine Supplementation
Study
Effect with 95% CI
Weight (%)
Huang, E. Y., et al. (2000). -0.11 [-4.14, 3.92] 1.42
Coghlin Dickson, T. M., et al. (2000). 0.15 [-0.92, 1.22] 17.63
Li, Y., et al. (2006). -0.42 [-1.55, 0.72] 21.43
Choi, K., et al. (2007) -1.95 [-3.19, -0.70] 37.43
Chattopadhyay, S., et al. (2014). 1.16 [-1.15, 3.47] 2.60
Huang, C.-J., et al. (2019). -1.19 [-4.43, 2.05] 4.33
Widjaja, N. A., et al. (2020). -1.14 [-4.39, 2.11] 4.18
Al-Taie, A. and A. Koseoglu (2021). -1.50 [-3.75, 0.76] 10.98
Overall -0.61 [-1.15, -0.06]
Heterogeneity: I^2 = 27.12%, H^2 = 1.37
Test of $\theta_i = \theta_j$: Q(7) = 9.60, p = 0.21
Test of $\theta = 0$: z = -2.17, p = 0.03
-4
-2
0
2
4

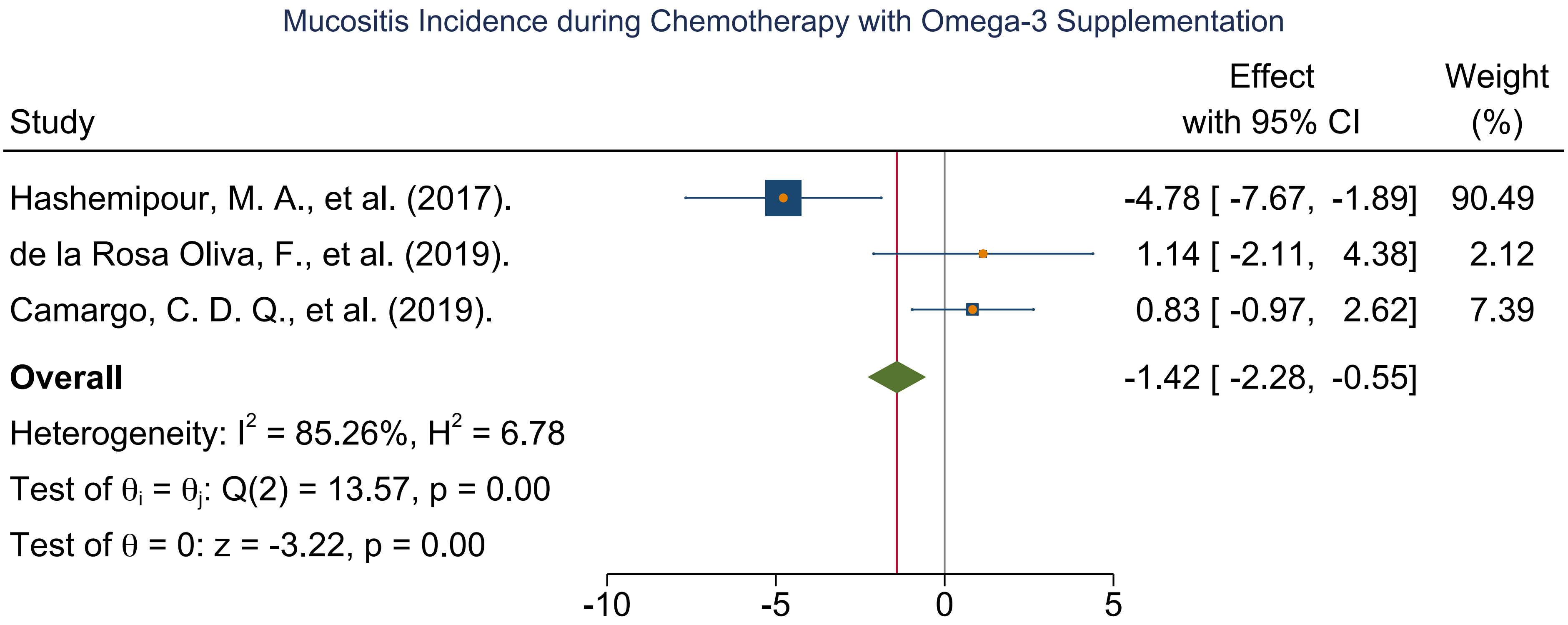

Mucositis Incidence during Chemotherapy with Omega-3 Supplementation
Study
Effect with 95% CI
Weight (%)
Hashemipour, M. A., et al. (2017). -4.78 [-7.67, -1.89] 90.49
de la Rosa Oliva, F., et al. (2019). 1.14 [-2.11, 4.38] 2.12
Camargo, C. D. Q., et al. (2019). 0.83 [-0.97, 2.62] 7.39
Overall -1.42 [-2.28, -0.55]
Heterogeneity: I^2 = 85.26%, H^2 = 6.78
Test of $\theta_i = \theta_j$: Q(2) = 13.57, p = 0.00
Test of θ = 0: z = -3.22, p = 0.00
-10
-5
0
5

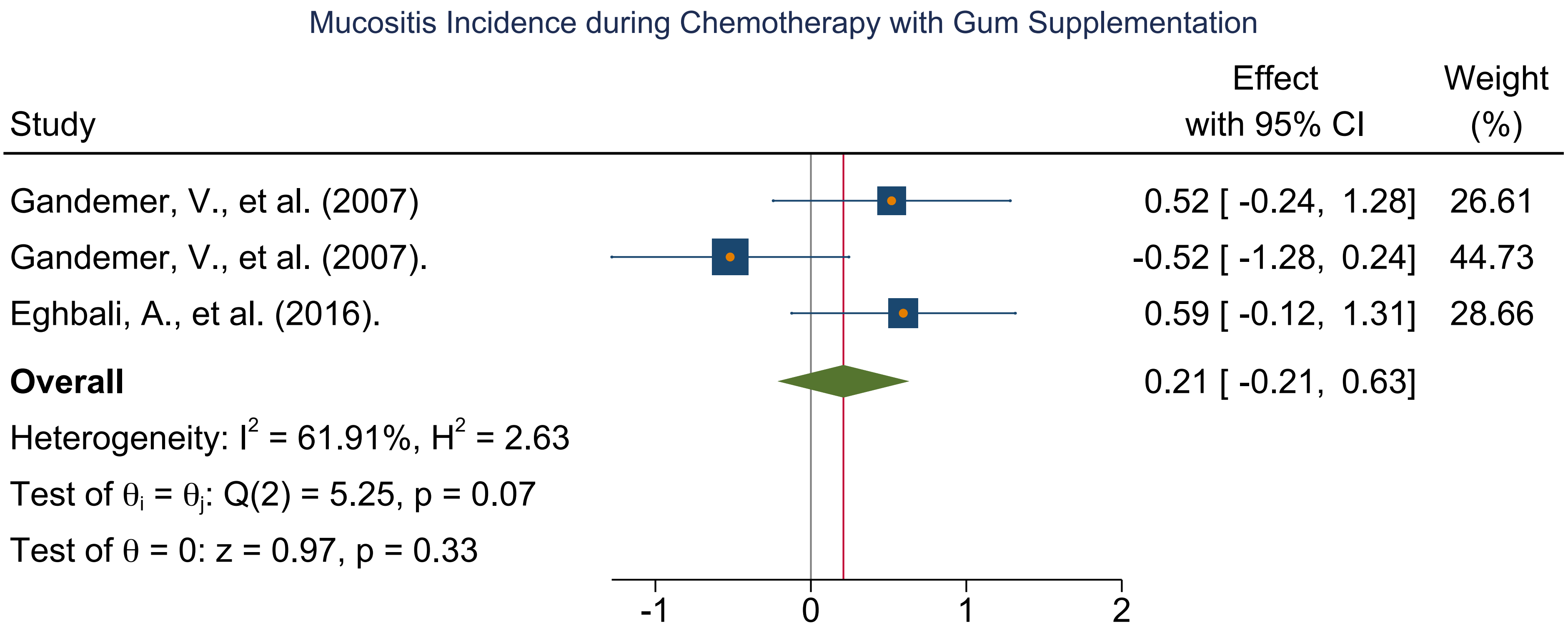

Mucositis Incidence during Chemotherapy with Gum Supplementation
Study
Effect with 95% CI
Weight (%)
Gandemer, V., et al. (2007)
0.52 [-0.24, 1.28]
26.61
Gandemer, V., et al. (2007).
-0.52 [-1.28, 0.24]
44.73
Eghbali, A., et al. (2016).
0.59 [-0.12, 1.31]
28.66
Overall
0.21 [-0.21, 0.63]
Heterogeneity: I^2 = 61.91%, H^2 = 2.63
Test of $\theta_i = \theta_j$: Q(2) = 5.25, p = 0.07
Test of θ = 0: z = 0.97, p = 0.33
-1
0
1
2

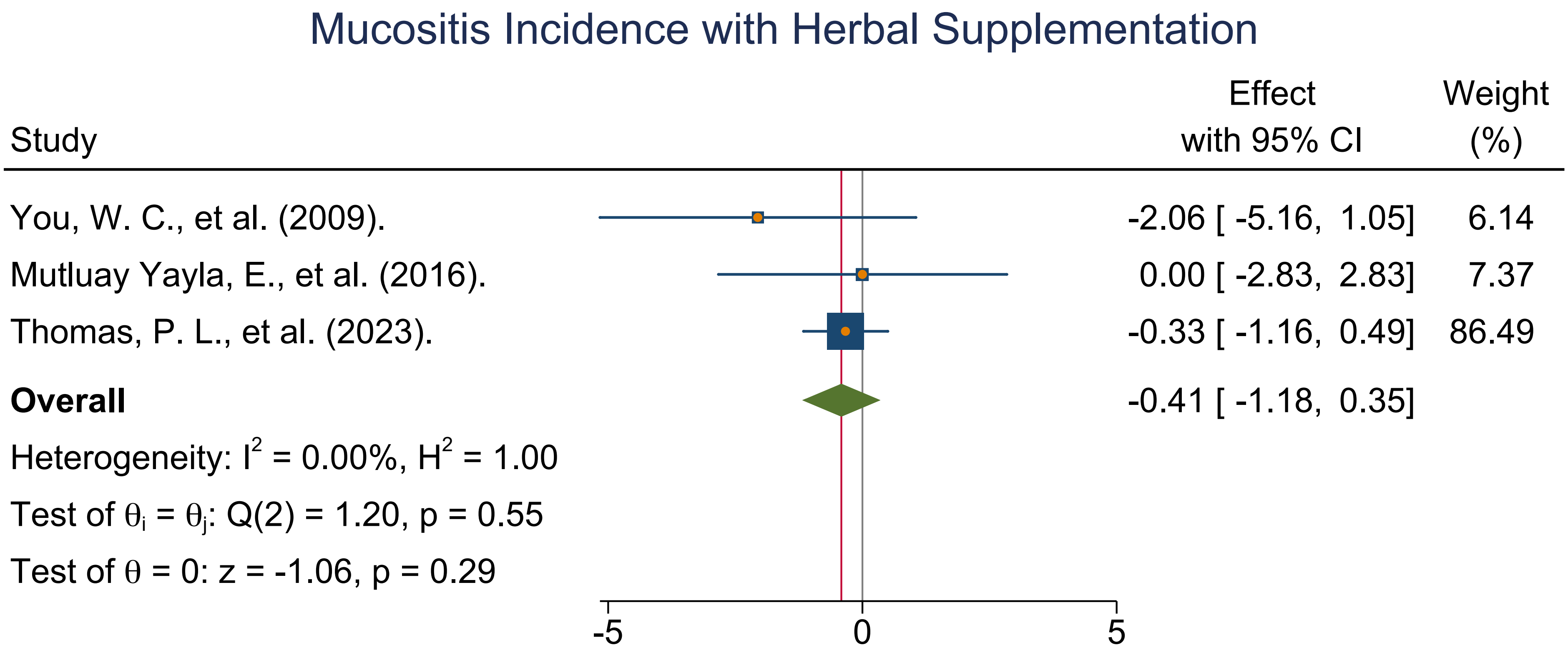

Mucositis Incidence with Herbal Supplementation
Study
Effect with 95% CI
Weight (%)
You, W. C., et al. (2009).
-2.06 [-5.16, 1.05]
6.14
Mutluay Yayla, E., et al. (2016).
0.00 [-2.83, 2.83]
7.37
Thomas, P. L., et al. (2023).
-0.33 [-1.16, 0.49]
86.49
Overall
-0.41 [-1.18, 0.35]
Heterogeneity: I2 = 0.00%, H2 = 1.00
Test of θi = θj: Q(2) = 1.20, p = 0.55
Test of θ = 0: z = -1.06, p = 0.29
-5
0
5

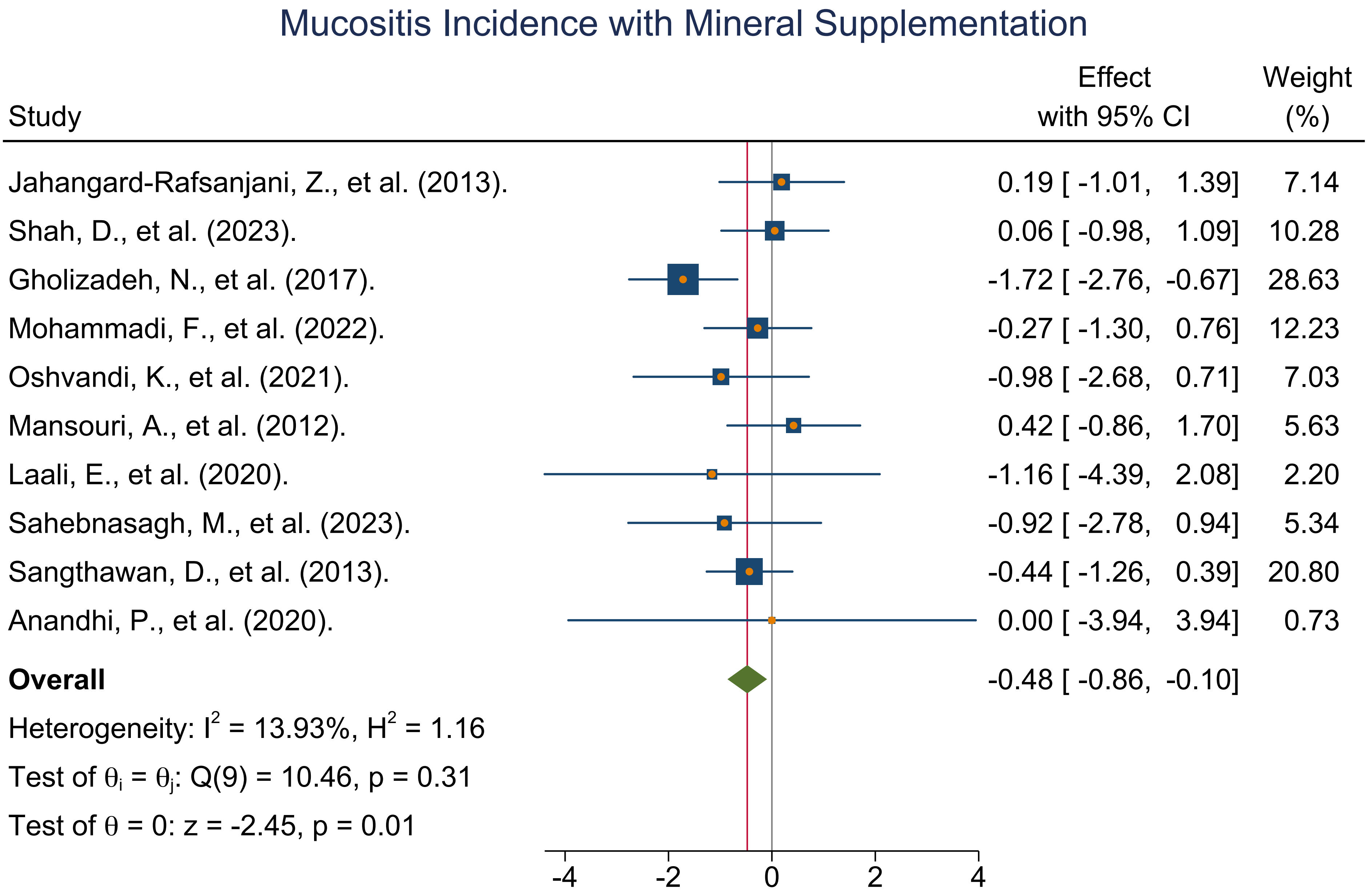
Mucositis Incidence with Mineral Supplementation
Study
Effect with 95% CI
Weight (%)
Jahangard-Rafsanjani, Z., et al. (2013). 0.19 [-1.01, 1.39] 7.14
Shah, D., et al. (2023). 0.06 [-0.98, 1.09] 10.28
Gholizadeh, N., et al. (2017). -1.72 [-2.76, -0.67] 28.63
Mohammadi, F., et al. (2022). -0.27 [-1.30, 0.76] 12.23
Oshvandi, K., et al. (2021). -0.98 [-2.68, 0.71] 7.03
Mansouri, A., et al. (2012). 0.42 [-0.86, 1.70] 5.63
Laali, E., et al. (2020). -1.16 [-4.39, 2.08] 2.20
Sahebnasagh, M., et al. (2023). -0.92 [-2.78, 0.94] 5.34
Sangthawan, D., et al. (2013). -0.44 [-1.26, 0.39] 20.80
Anandhi, P., et al. (2020). 0.00 [-3.94, 3.94] 0.73
Overall -0.48 [-0.86, -0.10]
Heterogeneity: I^2 = 13.93%, H^2 = 1.16
Test of $\theta_i = \theta_j$: Q(9) = 10.46, p = 0.31
Test of θ = 0: z = -2.45, p = 0.01
-4 -2 0 2 4

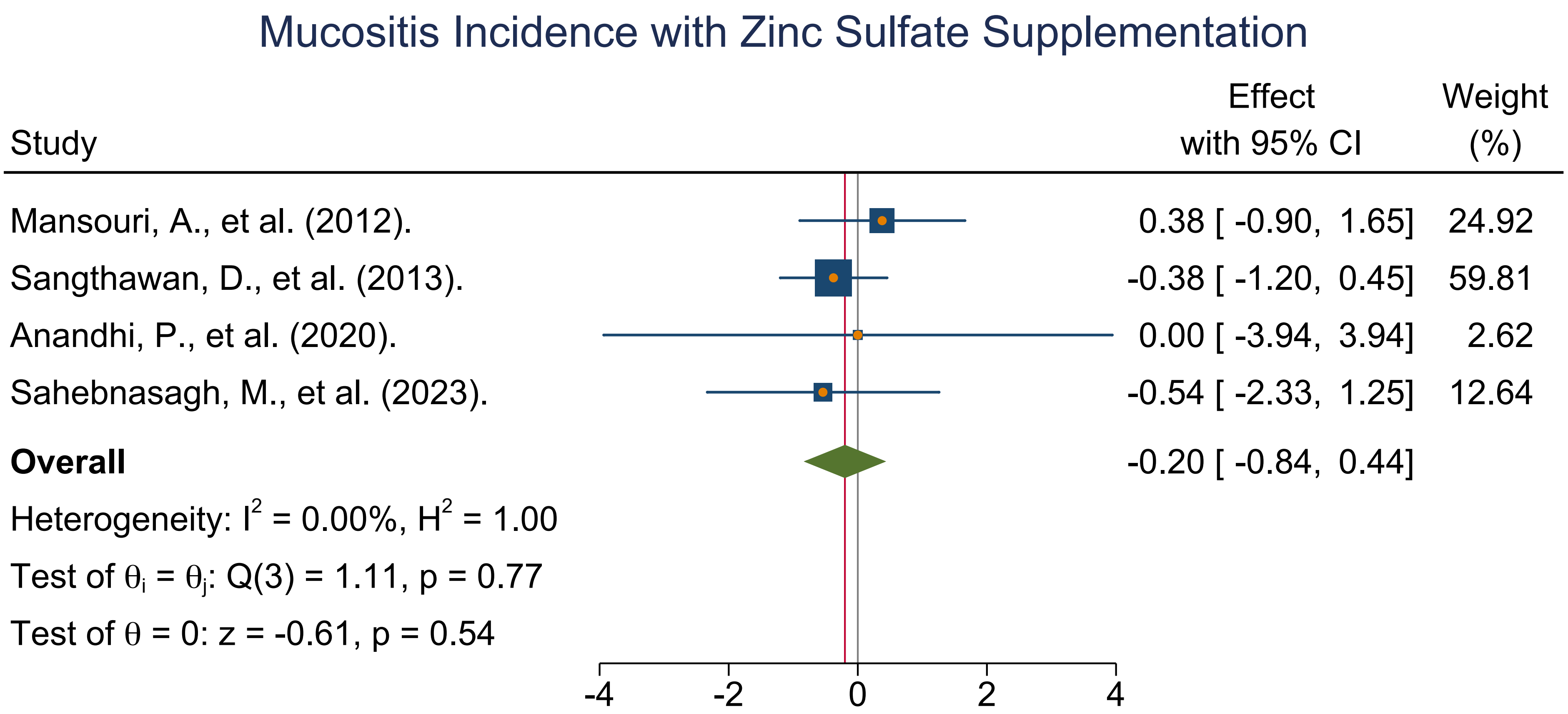
Mucositis Incidence with Zinc Sulfate Supplementation
Study
Effect with 95% CI
Weight (%)
Mansouri, A., et al. (2012). 0.38 [-0.90, 1.65] 24.92
Sangthawan, D., et al. (2013). -0.38 [-1.20, 0.45] 59.81
Anandhi, P., et al. (2020). 0.00 [-3.94, 3.94] 2.62
Sahebnasagh, M., et al. (2023). -0.54 [-2.33, 1.25] 12.64
Overall -0.20 [-0.84, 0.44]
Heterogeneity: I^2 = 0.00%, H^2 = 1.00
Test of $\theta_i = \theta_j$: Q(3) = 1.11, p = 0.77
Test of $\theta = 0$: z = -0.61, p = 0.54
-4
-2
0
2
4

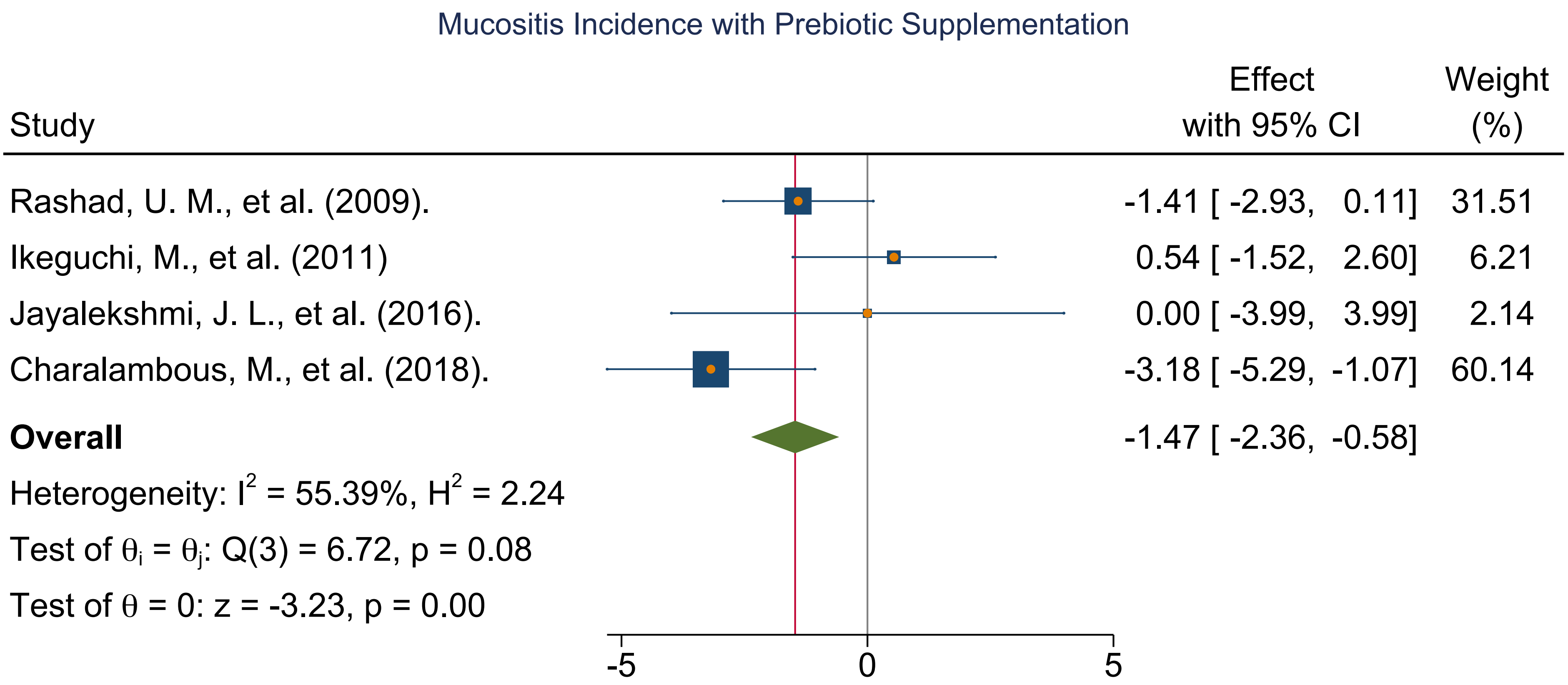
Mucositis Incidence with Prebiotic Supplementation
Study
Effect with 95% CI
Weight (%)
Rashad, U. M., et al. (2009). -1.41 [-2.93, 0.11] 31.51
Ikeguchi, M., et al. (2011) 0.54 [-1.52, 2.60] 6.21
Jayalekshmi, J. L., et al. (2016). 0.00 [-3.99, 3.99] 2.14
Charalambous, M., et al. (2018). -3.18 [-5.29, -1.07] 60.14
Overall -1.47 [-2.36, -0.58]
Heterogeneity: I² = 55.39%, H² = 2.24
Test of θi = θj: Q(3) = 6.72, p = 0.08
Test of θ = 0: z = -3.23, p = 0.00
-5
0
5

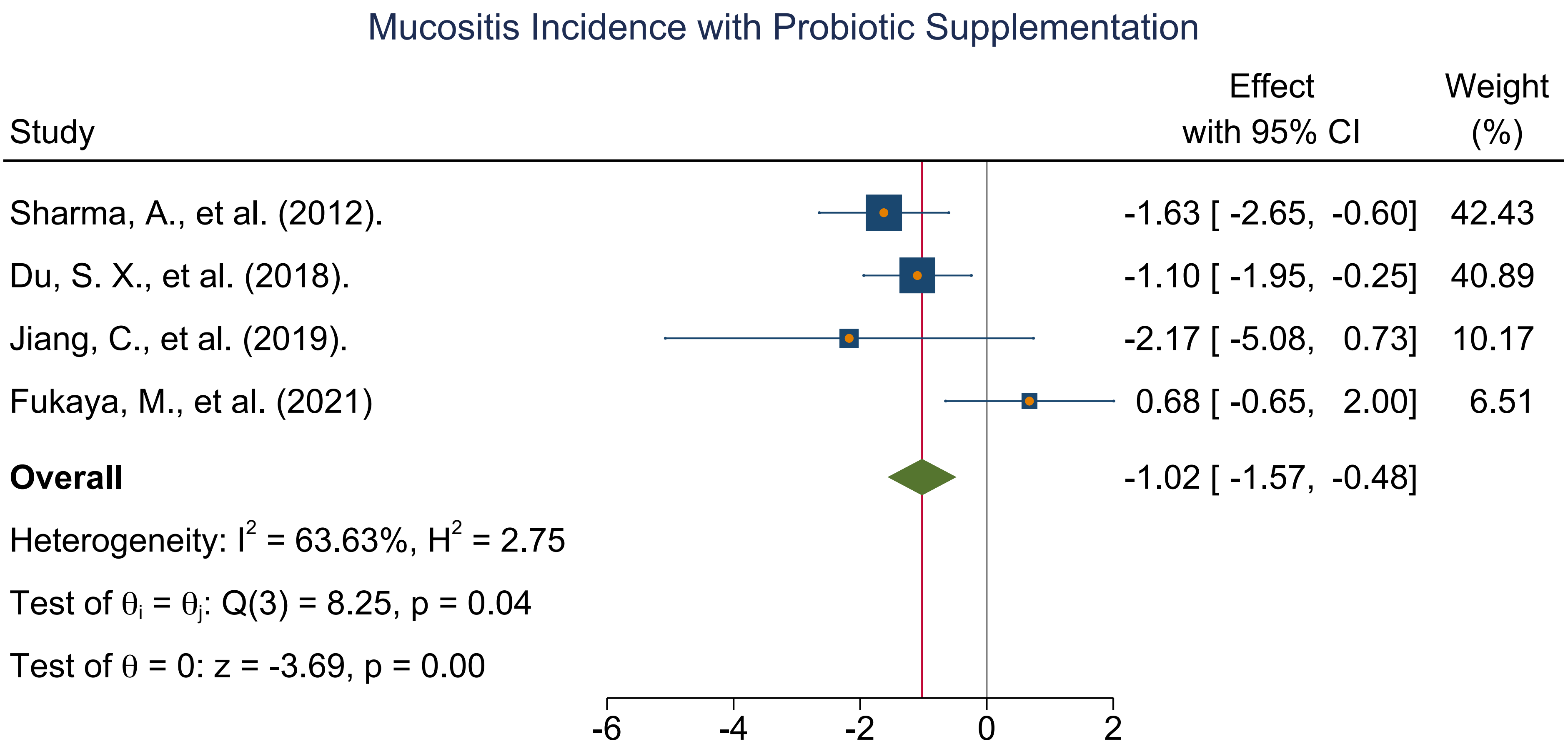
Mucositis Incidence with Probiotic Supplementation
Study
Effect with 95% CI
Weight (%)
Sharma, A., et al. (2012). -1.63 [-2.65, -0.60] 42.43
Du, S. X., et al. (2018). -1.10 [-1.95, -0.25] 40.89
Jiang, C., et al. (2019). -2.17 [-5.08, 0.73] 10.17
Fukaya, M., et al. (2021) 0.68 [-0.65, 2.00] 6.51
Overall -1.02 [-1.57, -0.48]
Heterogeneity: I^2 = 63.63%, H^2 = 2.75
Test of $\theta_i = \theta_j$: Q(3) = 8.25, p = 0.04
Test of θ = 0: z = -3.69, p = 0.00
-6
-4
-2
0
2

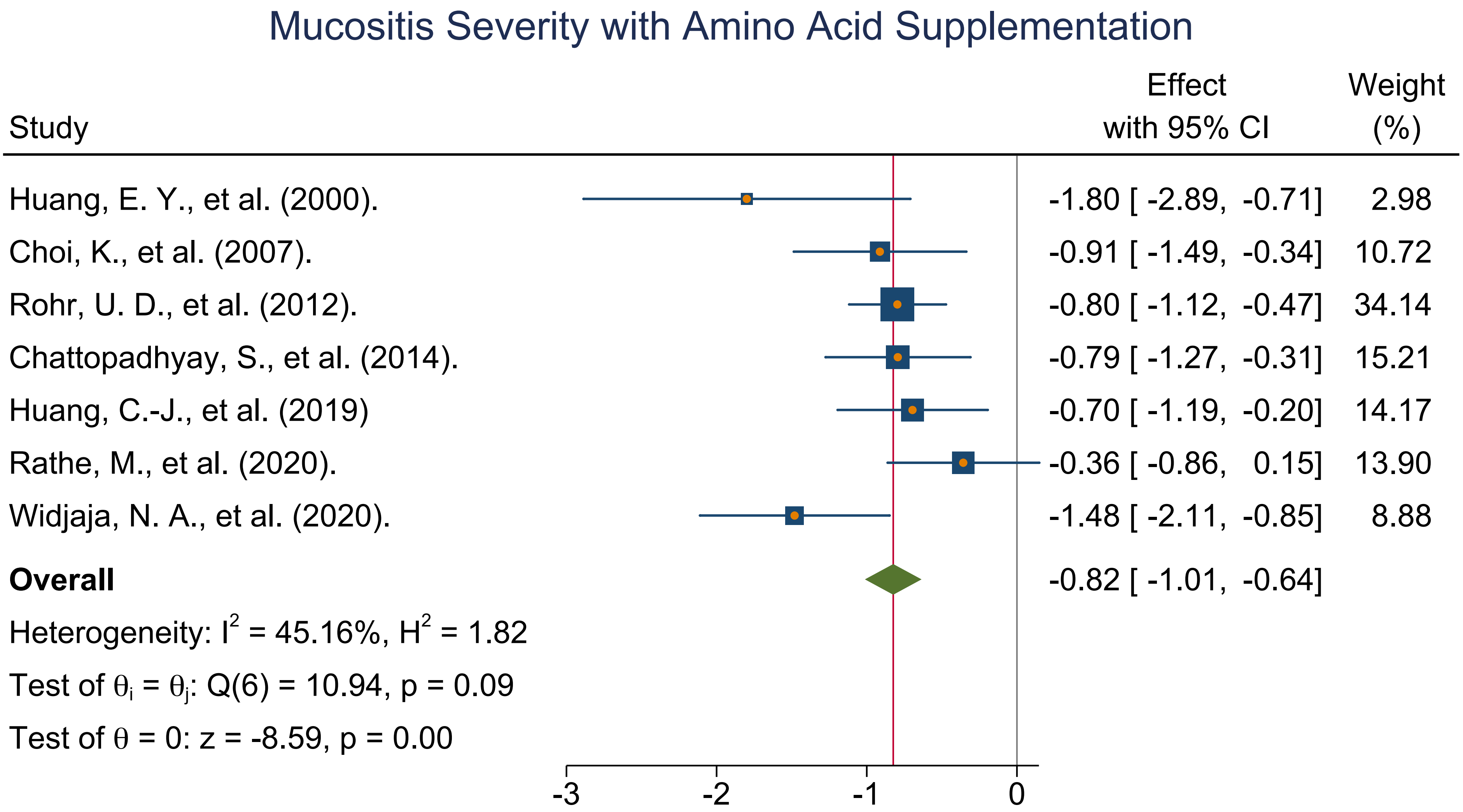

Mucositis Severity with Amino Acid Supplementation
Study
Effect with 95% CI
Weight (%)
Huang, E. Y., et al. (2000). -1.80 [-2.89, -0.71] 2.98
Choi, K., et al. (2007). -0.91 [-1.49, -0.34] 10.72
Rohr, U. D., et al. (2012). -0.80 [-1.12, -0.47] 34.14
Chattopadhyay, S., et al. (2014). -0.79 [-1.27, -0.31] 15.21
Huang, C.-J., et al. (2019) -0.70 [-1.19, -0.20] 14.17
Rathe, M., et al. (2020). -0.36 [-0.86, 0.15] 13.90
Widjaja, N. A., et al. (2020). -1.48 [-2.11, -0.85] 8.88
Overall -0.82 [-1.01, -0.64]
Heterogeneity: I² = 45.16%, H² = 1.82
Test of θi = θj: Q(6) = 10.94, p = 0.09
Test of θ = 0: z = -8.59, p = 0.00
-3
-2
-1
0

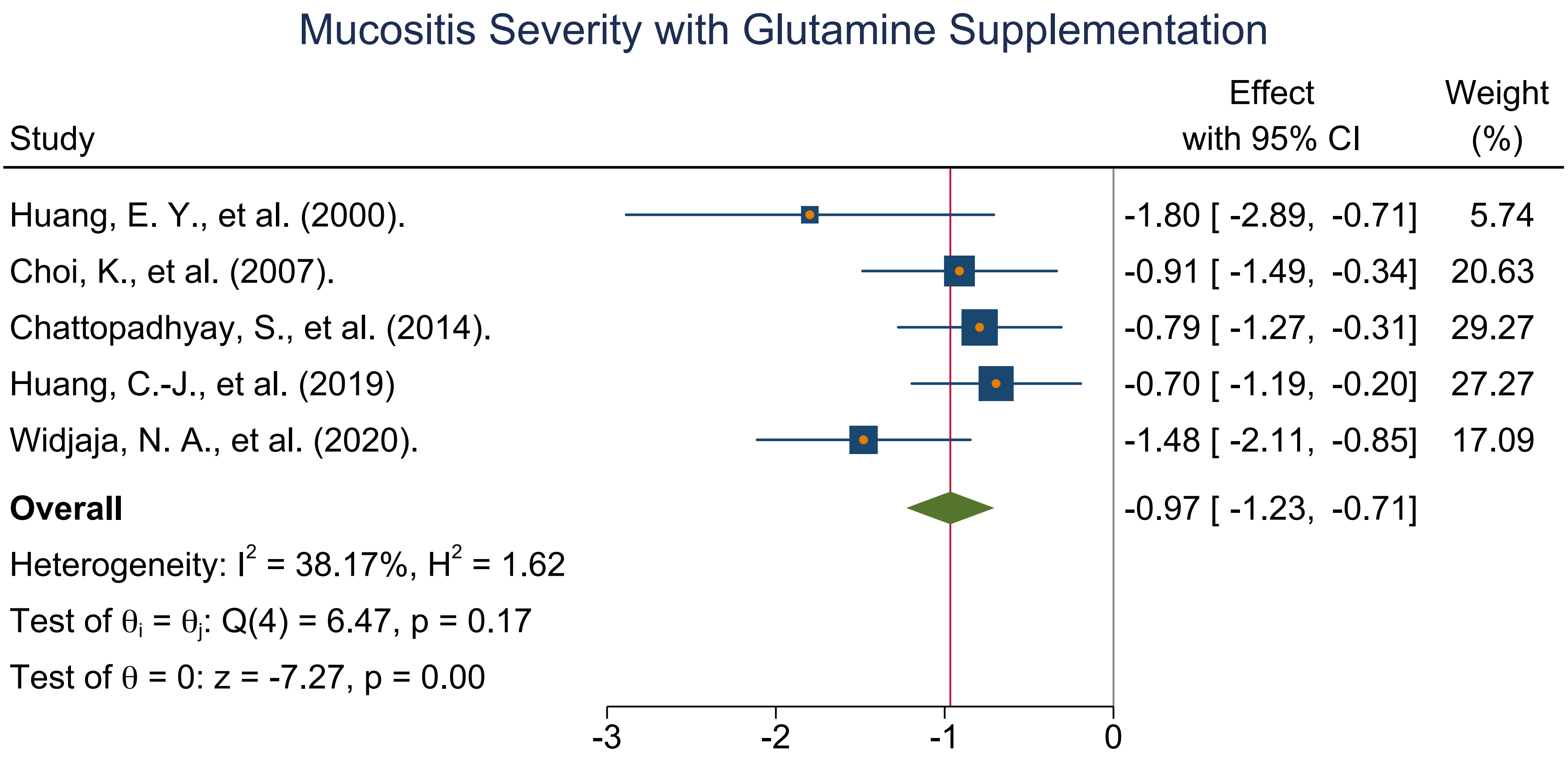
Mucositis Severity with Glutamine Supplementation
Study
Effect with 95% CI
Weight (%)
Huang, E. Y., et al. (2000).
-1.80 [-2.89, -0.71]
5.74
Choi, K., et al. (2007).
-0.91 [-1.49, -0.34]
20.63
Chattopadhyay, S., et al. (2014).
-0.79 [-1.27, -0.31]
29.27
Huang, C.-J., et al. (2019)
-0.70 [-1.19, -0.20]
27.27
Widjaja, N. A., et al. (2020).
-1.48 [-2.11, -0.85]
17.09
Overall
-0.97 [-1.23, -0.71]
Heterogeneity: I^2 = 38.17%, H^2 = 1.62
Test of $\theta_i = \theta_j$: Q(4) = 6.47, p = 0.17
Test of θ = 0: z = -7.27, p = 0.00
-3
-2
-1
0

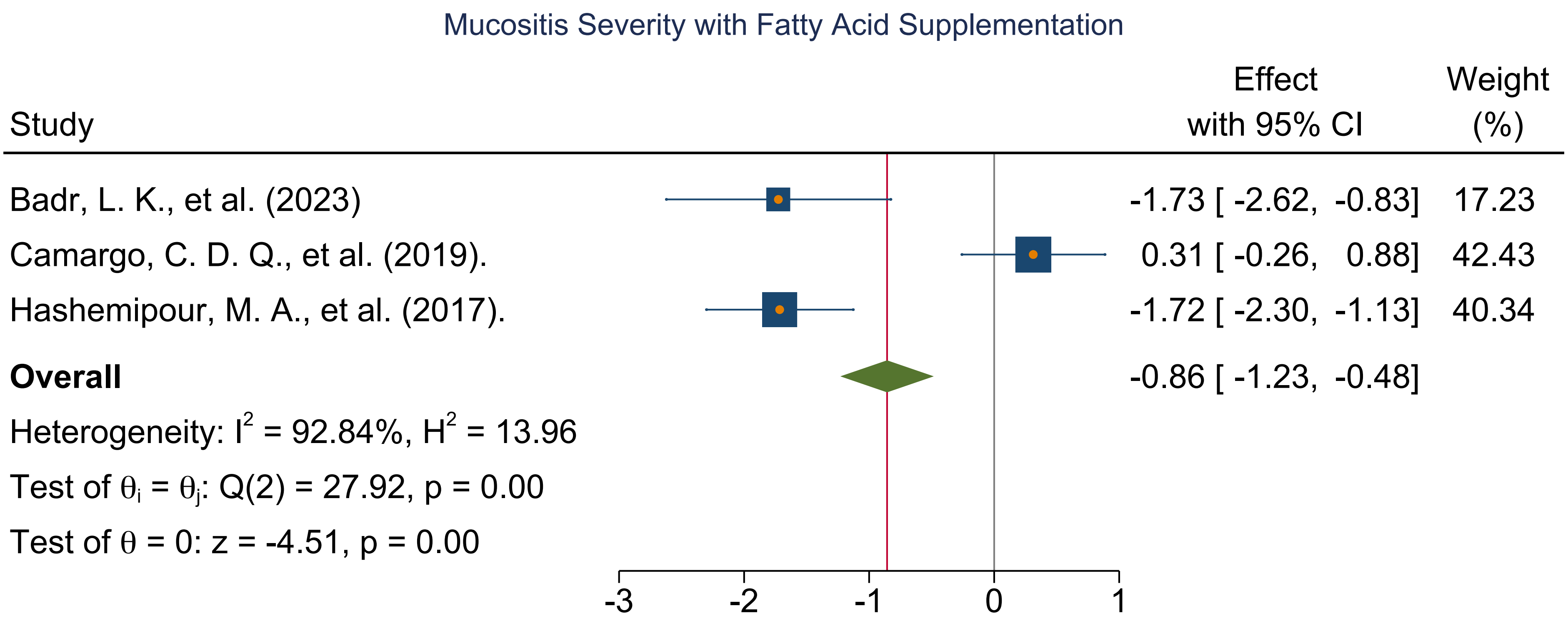
Mucositis Severity with Fatty Acid Supplementation
Study
Effect with 95% CI
Weight (%)
Badr, L. K., et al. (2023) -1.73 [-2.62, -0.83] 17.23
Camargo, C. D. Q., et al. (2019). 0.31 [-0.26, 0.88] 42.43
Hashemipour, M. A., et al. (2017). -1.72 [-2.30, -1.13] 40.34
Overall -0.86 [-1.23, -0.48]
Heterogeneity: I^2 = 92.84%, H^2 = 13.96
Test of $\theta_i = \theta_j$: Q(2) = 27.92, p = 0.00
Test of θ = 0: z = -4.51, p = 0.00
-3
-2
-1
0
1

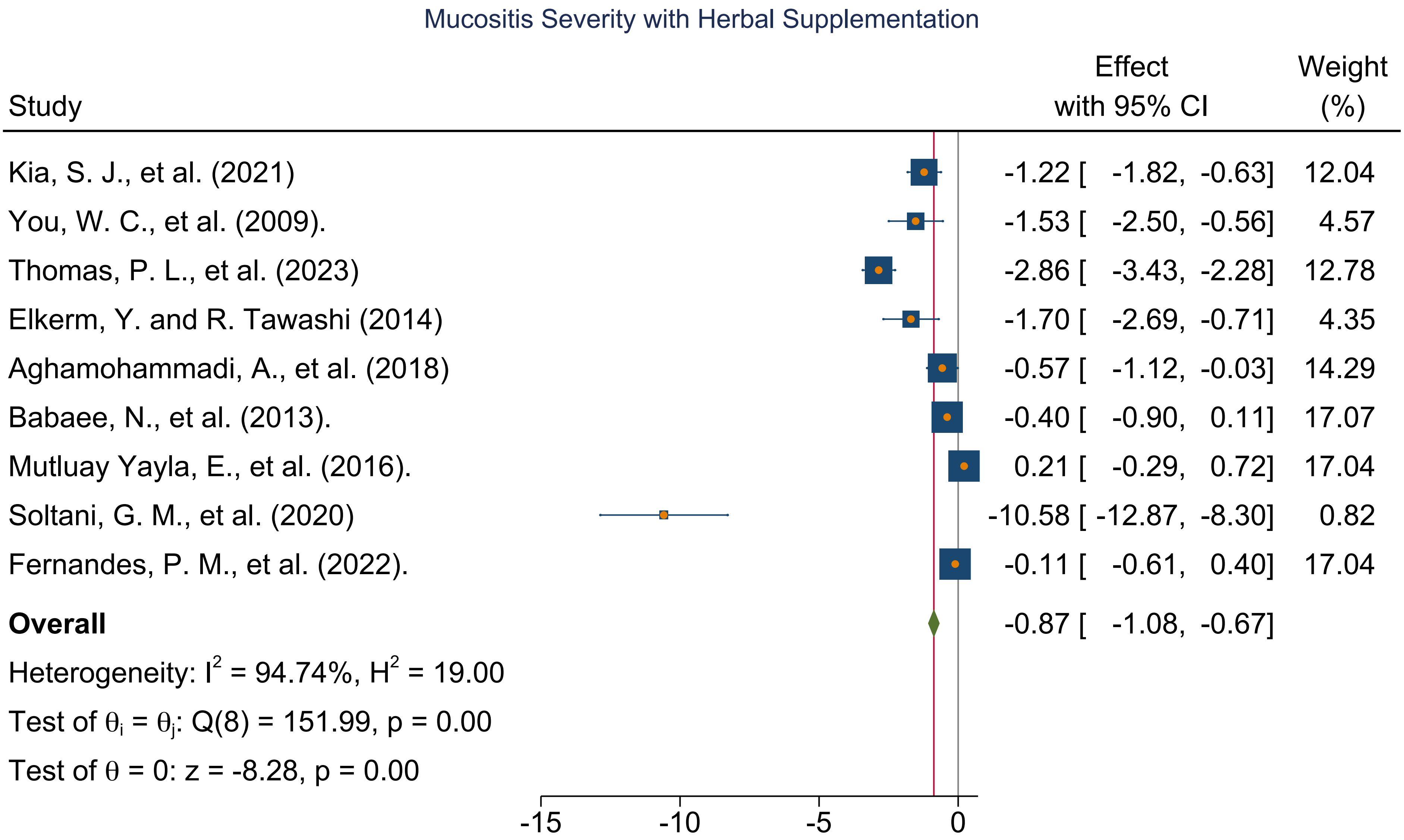
Mucositis Severity with Herbal Supplementation
Study
Effect
with 95% CI
Weight
(%)
Kia, S. J., et al. (2021)
-1.22 [-1.82, -0.63]
12.04
You, W. C., et al. (2009).
-1.53 [-2.50, -0.56]
4.57
Thomas, P. L., et al. (2023)
-2.86 [-3.43, -2.28]
12.78
Elkerm, Y. and R. Tawashi (2014)
-1.70 [-2.69, -0.71]
4.35
Aghamohammadi, A., et al. (2018)
-0.57 [-1.12, -0.03]
14.29
Babaee, N., et al. (2013).
-0.40 [-0.90, 0.11]
17.07
Mutluay Yayla, E., et al. (2016).
0.21 [-0.29, 0.72]
17.04
Soltani, G. M., et al. (2020)
-10.58 [-12.87, -8.30]
0.82
Fernandes, P. M., et al. (2022).
-0.11 [-0.61, 0.40]
17.04
Overall
-0.87 [-1.08, -0.67]
Heterogeneity: I^2 = 94.74%, H^2 = 19.00
Test of $\theta_i = \theta_j$: Q(8) = 151.99, p = 0.00
Test of θ = 0: z = -8.28, p = 0.00
-15
-10
-5
0

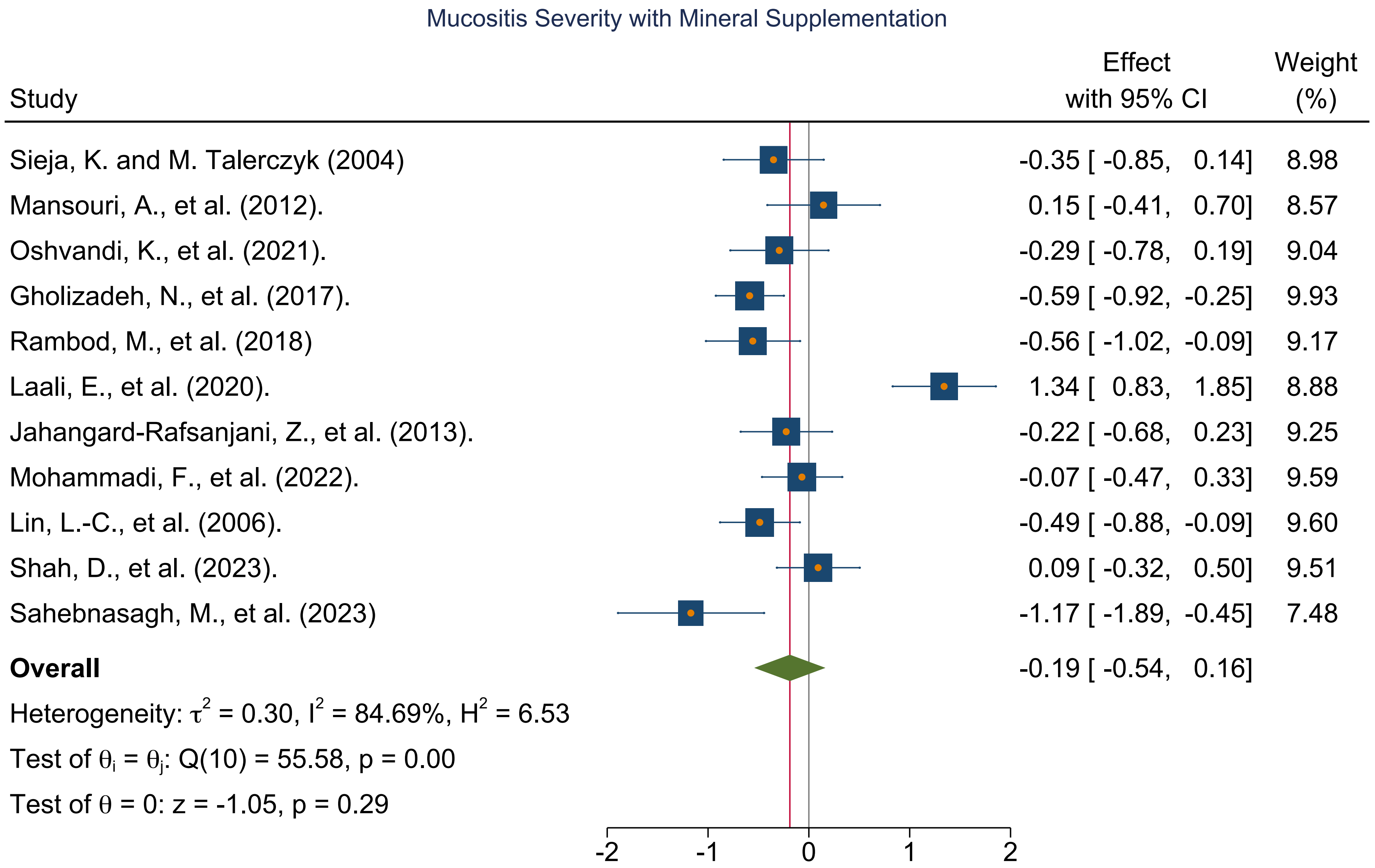
Mucositis Severity with Mineral Supplementation
Study
Effect with 95% CI
Weight (%)
Sieja, K. and M. Talerczyk (2004) -0.35 [-0.85, 0.14] 8.98
Mansouri, A., et al. (2012). 0.15 [-0.41, 0.70] 8.57
Oshvandi, K., et al. (2021). -0.29 [-0.78, 0.19] 9.04
Gholizadeh, N., et al. (2017). -0.59 [-0.92, -0.25] 9.93
Rambod, M., et al. (2018) -0.56 [-1.02, -0.09] 9.17
Laali, E., et al. (2020). 1.34 [0.83, 1.85] 8.88
Jahangard-Rafsanjani, Z., et al. (2013). -0.22 [-0.68, 0.23] 9.25
Mohammadi, F., et al. (2022). -0.07 [-0.47, 0.33] 9.59
Lin, L.-C., et al. (2006). -0.49 [-0.88, -0.09] 9.60
Shah, D., et al. (2023). 0.09 [-0.32, 0.50] 9.51
Sahebnasagh, M., et al. (2023) -1.17 [-1.89, -0.45] 7.48
Overall -0.19 [-0.54, 0.16]
Heterogeneity: τ^2 = 0.30, I^2 = 84.69%, H^2 = 6.53
Test of $\theta_i = \theta_j$: Q(10) = 55.58, p = 0.00
Test of θ = 0: z = -1.05, p = 0.29
-2
-1
0
1
2

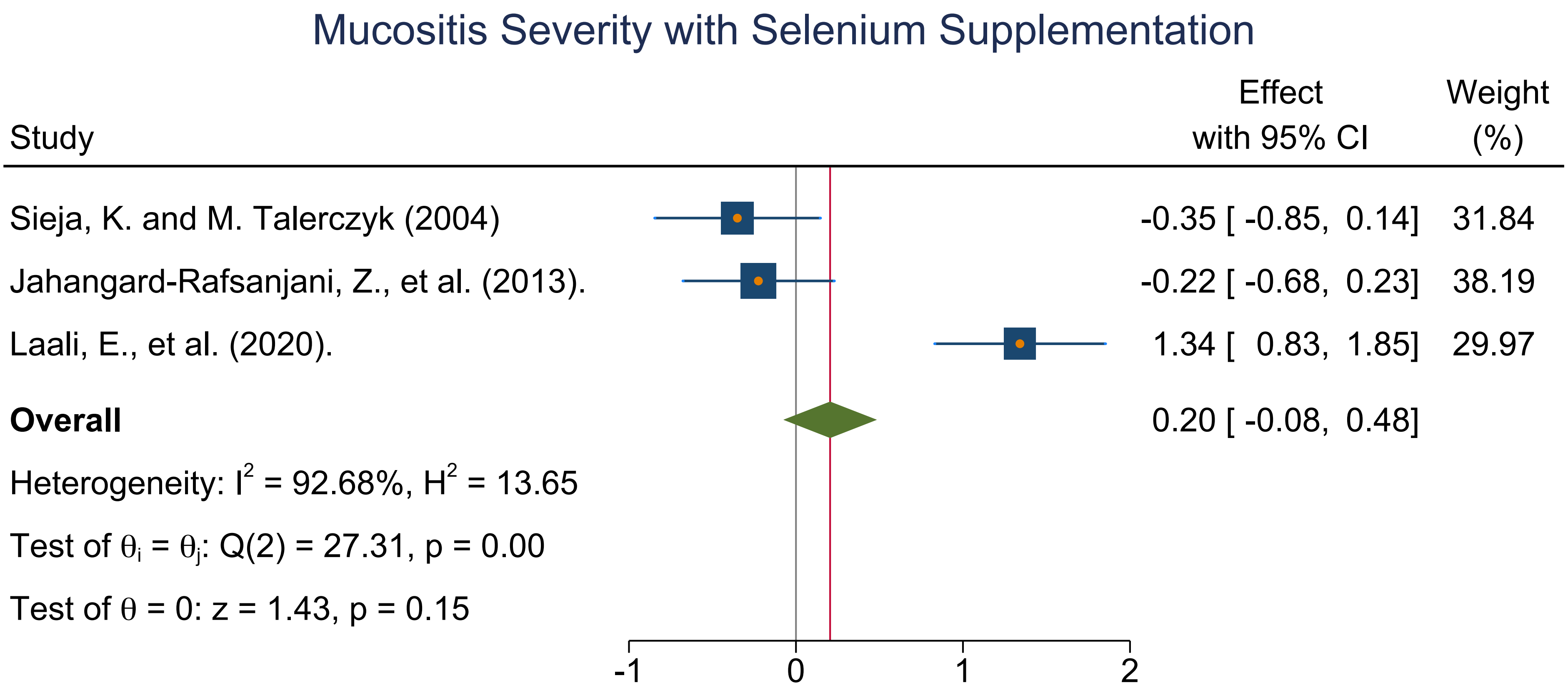
Mucositis Severity with Selenium Supplementation
Study
Effect with 95% CI
Weight (%)
Sieja, K. and M. Talerczyk (2004) -0.35 [-0.85, 0.14] 31.84
Jahangard-Rafsanjani, Z., et al. (2013). -0.22 [-0.68, 0.23] 38.19
Laali, E., et al. (2020). 1.34 [0.83, 1.85] 29.97
Overall 0.20 [-0.08, 0.48]
Heterogeneity: I^2 = 92.68%, H^2 = 13.65
Test of $\theta_i = \theta_j$: Q(2) = 27.31, p = 0.00
Test of θ = 0: z = 1.43, p = 0.15
-1
0
1
2

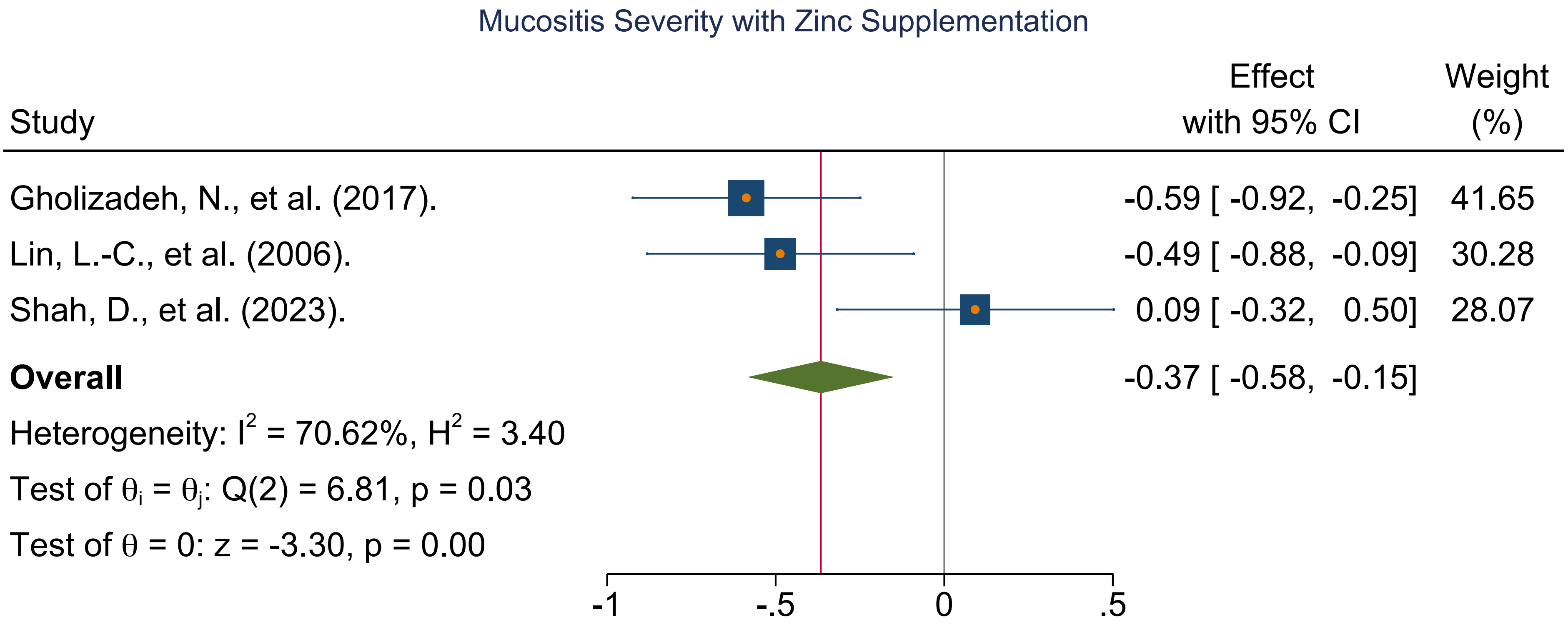
Mucositis Severity with Zinc Supplementation
Study
Effect with 95% CI
Weight (%)
Gholizadeh, N., et al. (2017). -0.59 [-0.92, -0.25] 41.65
Lin, L.-C., et al. (2006). -0.49 [-0.88, -0.09] 30.28
Shah, D., et al. (2023). 0.09 [-0.32, 0.50] 28.07
Overall -0.37 [-0.58, -0.15]
Heterogeneity: I^2 = 70.62%, H^2 = 3.40
Test of $\theta_i = \theta_j$: Q(2) = 6.81, p = 0.03
Test of θ = 0: z = -3.30, p = 0.00
-1
-.5
0
.5

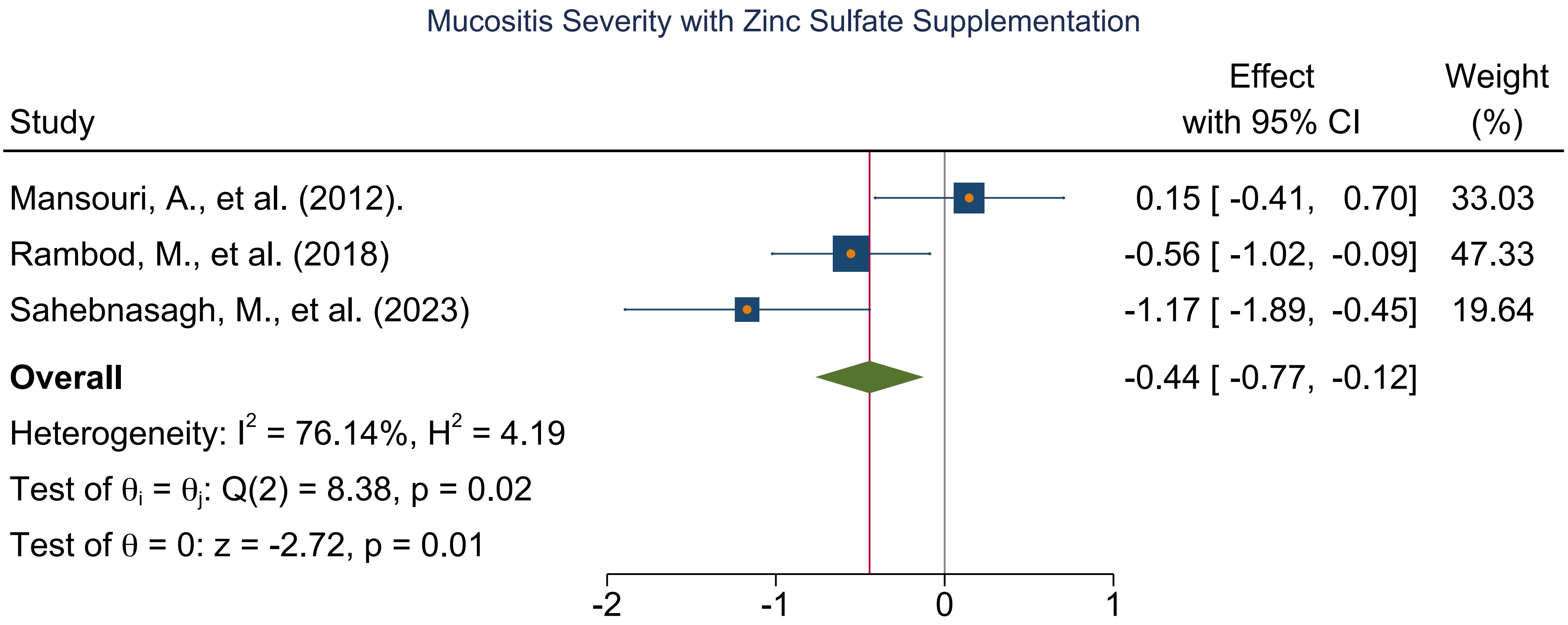

Mucositis Severity with Zinc Sulfate Supplementation
Study
Effect with 95% CI
Weight (%)
Mansouri, A., et al. (2012).
0.15 [-0.41, 0.70]
33.03
Rambod, M., et al. (2018)
-0.56 [-1.02, -0.09]
47.33
Sahebnasagh, M., et al. (2023)
-1.17 [-1.89, -0.45]
19.64
Overall
-0.44 [-0.77, -0.12]
Heterogeneity: I² = 76.14%, H² = 4.19
Test of θi = θj: Q(2) = 8.38, p = 0.02
Test of θ = 0: z = -2.72, p = 0.01
-2
-1
0
1

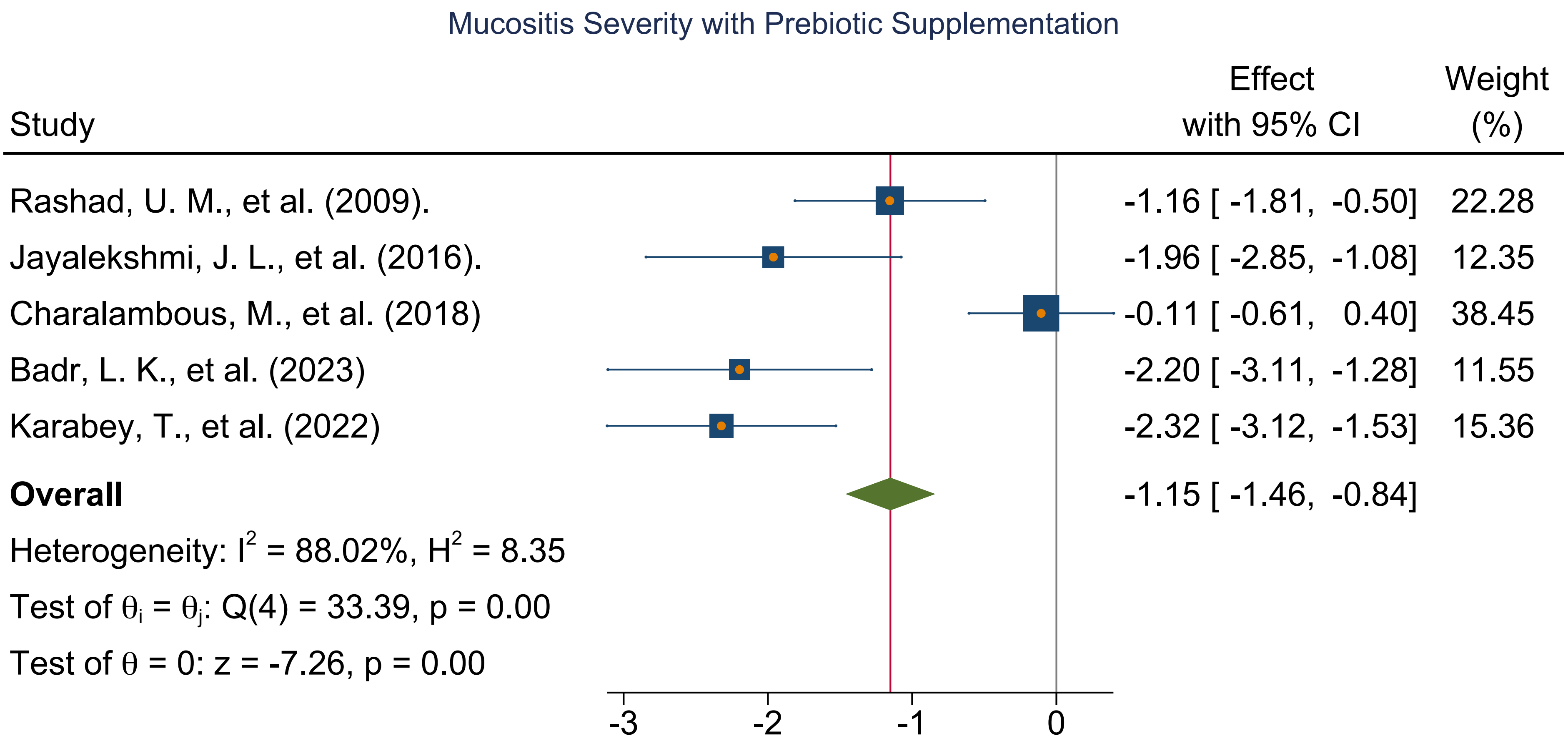

Mucositis Severity with Prebiotic Supplementation
Study
Effect with 95% CI
Weight (%)
Rashad, U. M., et al. (2009). -1.16 [-1.81, -0.50] 22.28
Jayalekshmi, J. L., et al. (2016). -1.96 [-2.85, -1.08] 12.35
Charalambous, M., et al. (2018) -0.11 [-0.61, 0.40] 38.45
Badr, L. K., et al. (2023) -2.20 [-3.11, -1.28] 11.55
Karabey, T., et al. (2022) -2.32 [-3.12, -1.53] 15.36
Overall -1.15 [-1.46, -0.84]
Heterogeneity: I^2 = 88.02%, H^2 = 8.35
Test of $\theta_i = \theta_j$: Q(4) = 33.39, p = 0.00
Test of θ = 0: z = -7.26, p = 0.00
-3
-2
-1
0

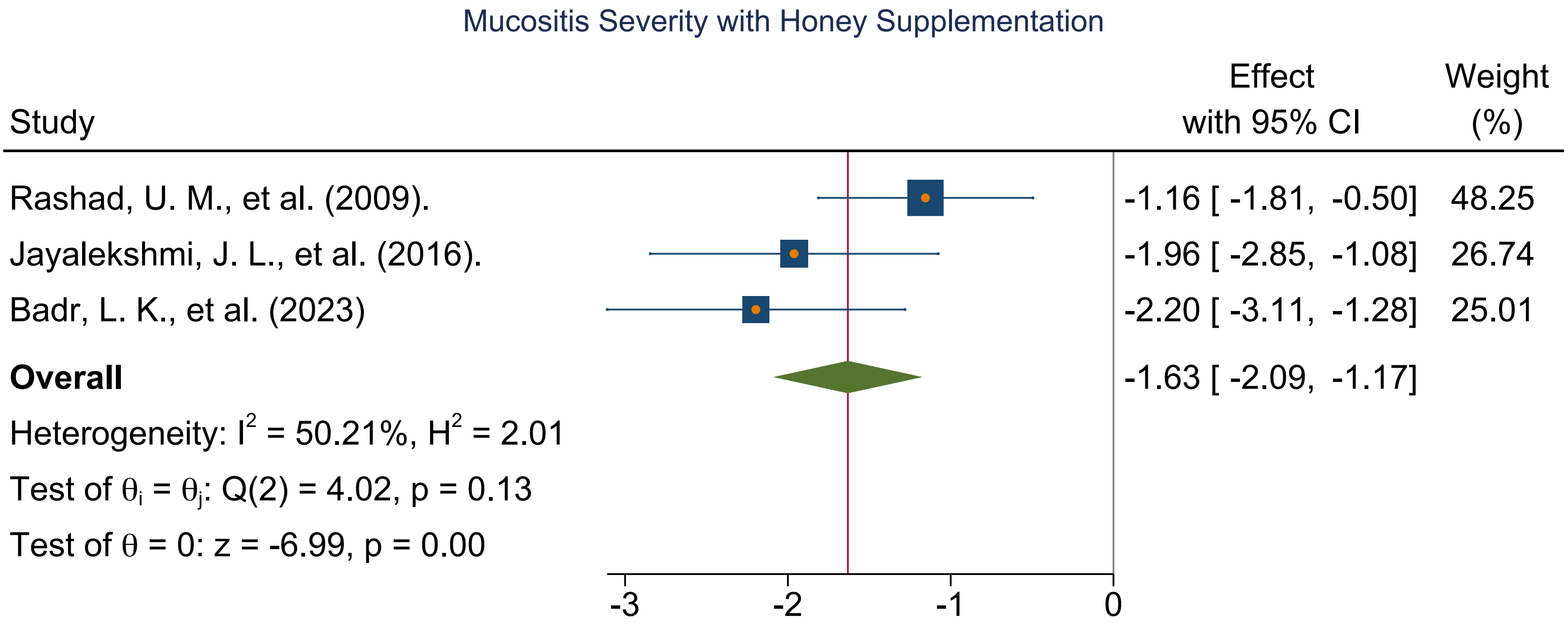
Mucositis Severity with Honey Supplementation
Study
Effect with 95% CI
Weight (%)
Rashad, U. M., et al. (2009).
-1.16 [-1.81, -0.50]
48.25
Jayalekshmi, J. L., et al. (2016).
-1.96 [-2.85, -1.08]
26.74
Badr, L. K., et al. (2023)
-2.20 [-3.11, -1.28]
25.01
Overall
-1.63 [-2.09, -1.17]
Heterogeneity: I2 = 50.21%, H2 = 2.01
Test of θi = θj: Q(2) = 4.02, p = 0.13
Test of θ = 0: z = -6.99, p = 0.00
-3
-2
-1
0

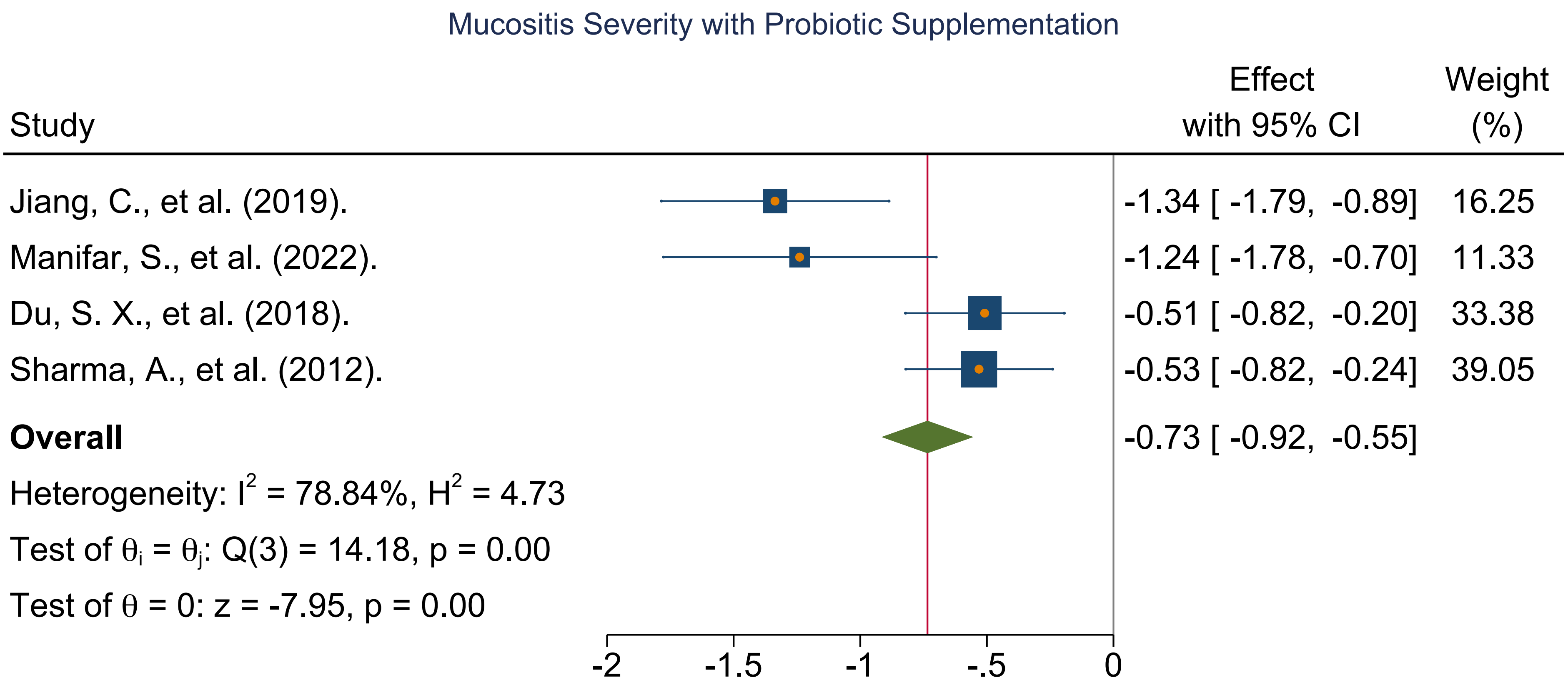
Mucositis Severity with Probiotic Supplementation
Study
Effect
with 95% CI
Weight
(%)
Jiang, C., et al. (2019).
-1.34 [-1.79, -0.89]
16.25
Manifar, S., et al. (2022).
-1.24 [-1.78, -0.70]
11.33
Du, S. X., et al. (2018).
-0.51 [-0.82, -0.20]
33.38
Sharma, A., et al. (2012).
-0.53 [-0.82, -0.24]
39.05
Overall
-0.73 [-0.92, -0.55]
Heterogeneity: I^2 = 78.84%, H^2 = 4.73
Test of $\theta_i = \theta_j$: Q(3) = 14.18, p = 0.00
Test of θ = 0: z = -7.95, p = 0.00
-2
-1.5
-1
-.5
0

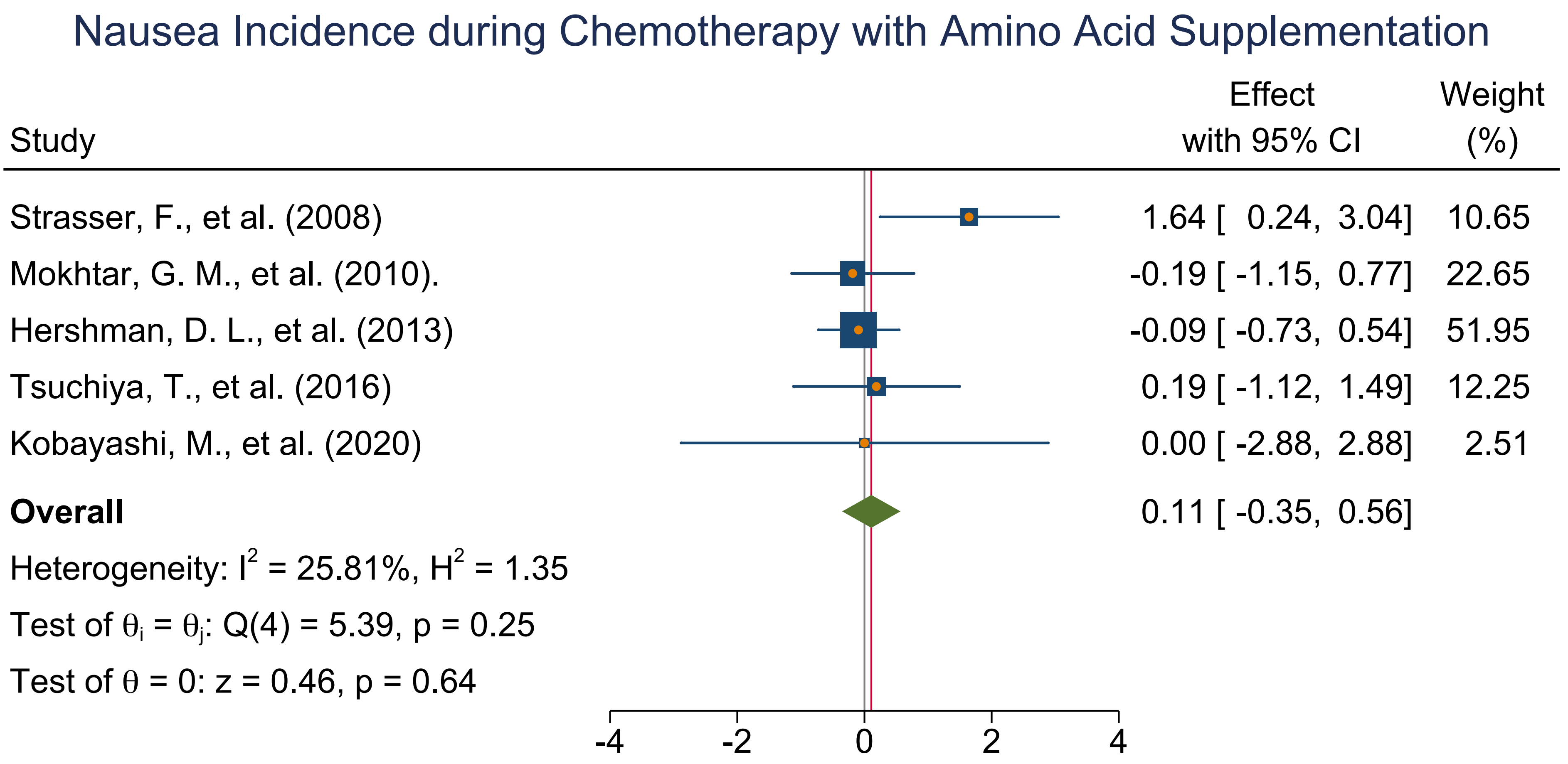

Nausea Incidence during Chemotherapy with Amino Acid Supplementation
Study
Effect with 95% CI
Weight (%)
Strasser, F., et al. (2008)
1.64 [0.24, 3.04]
10.65
Mokhtar, G. M., et al. (2010).
-0.19 [-1.15, 0.77]
22.65
Hershman, D. L., et al. (2013)
-0.09 [-0.73, 0.54]
51.95
Tsuchiya, T., et al. (2016)
0.19 [-1.12, 1.49]
12.25
Kobayashi, M., et al. (2020)
0.00 [-2.88, 2.88]
2.51
Overall
0.11 [-0.35, 0.56]
Heterogeneity: I^2 = 25.81%, H^2 = 1.35
Test of $\theta_i = \theta_j$: Q(4) = 5.39, p = 0.25
Test of θ = 0: z = 0.46, p = 0.64
-4
-2
0
2
4

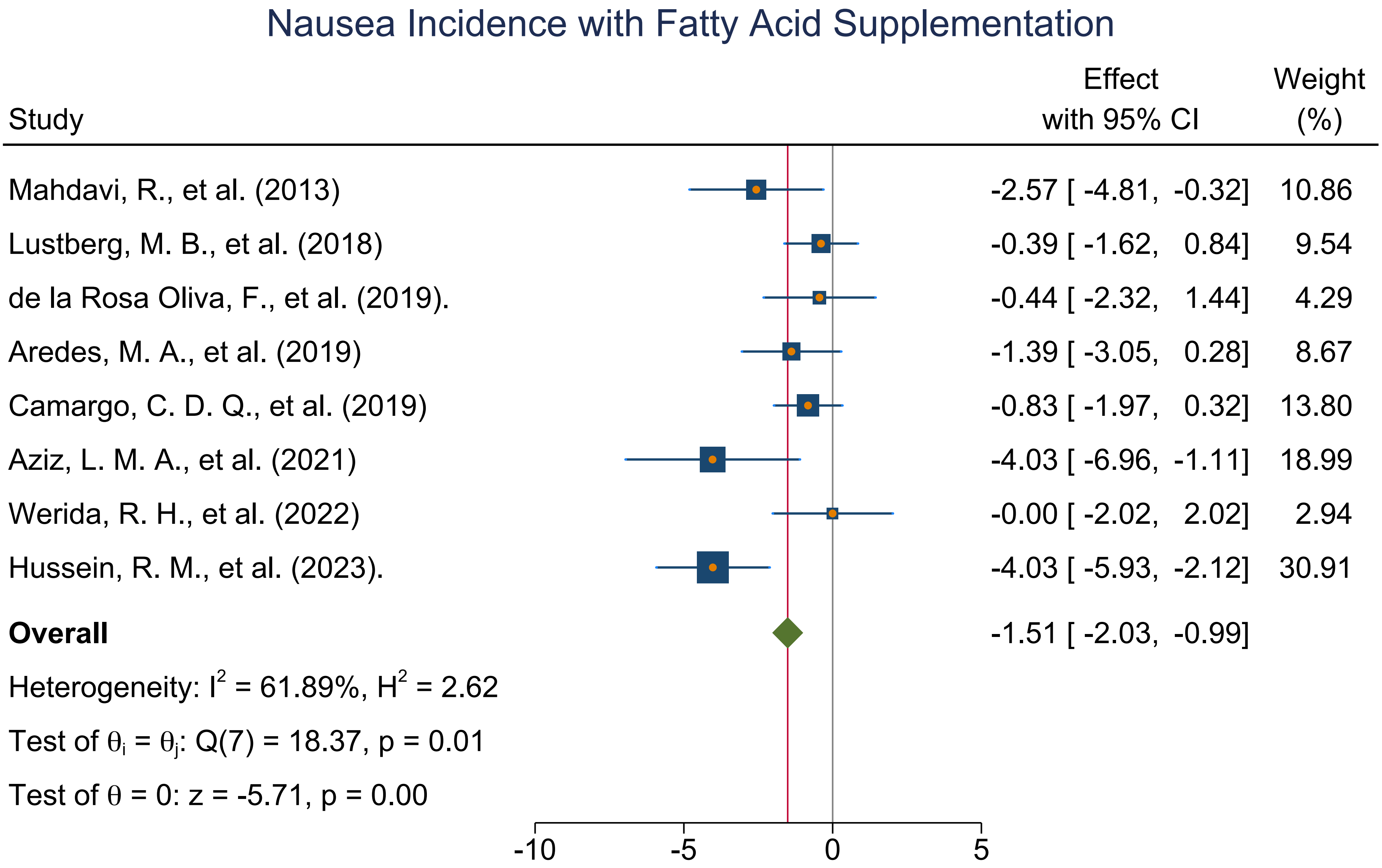
Nausea Incidence with Fatty Acid Supplementation
Study
Effect with 95% CI
Weight (%)
Mahdavi, R., et al. (2013) -2.57 [-4.81, -0.32] 10.86
Lustberg, M. B., et al. (2018) -0.39 [-1.62, 0.84] 9.54
de la Rosa Oliva, F., et al. (2019). -0.44 [-2.32, 1.44] 4.29
Aredes, M. A., et al. (2019) -1.39 [-3.05, 0.28] 8.67
Camargo, C. D. Q., et al. (2019) -0.83 [-1.97, 0.32] 13.80
Aziz, L. M. A., et al. (2021) -4.03 [-6.96, -1.11] 18.99
Werida, R. H., et al. (2022) -0.00 [-2.02, 2.02] 2.94
Hussein, R. M., et al. (2023). -4.03 [-5.93, -2.12] 30.91
Overall -1.51 [-2.03, -0.99]
Heterogeneity: I^2 = 61.89%, H^2 = 2.62
Test of $\theta_i = \theta_j$: Q(7) = 18.37, p = 0.01
Test of θ = 0: z = -5.71, p = 0.00
-10
-5
0
5

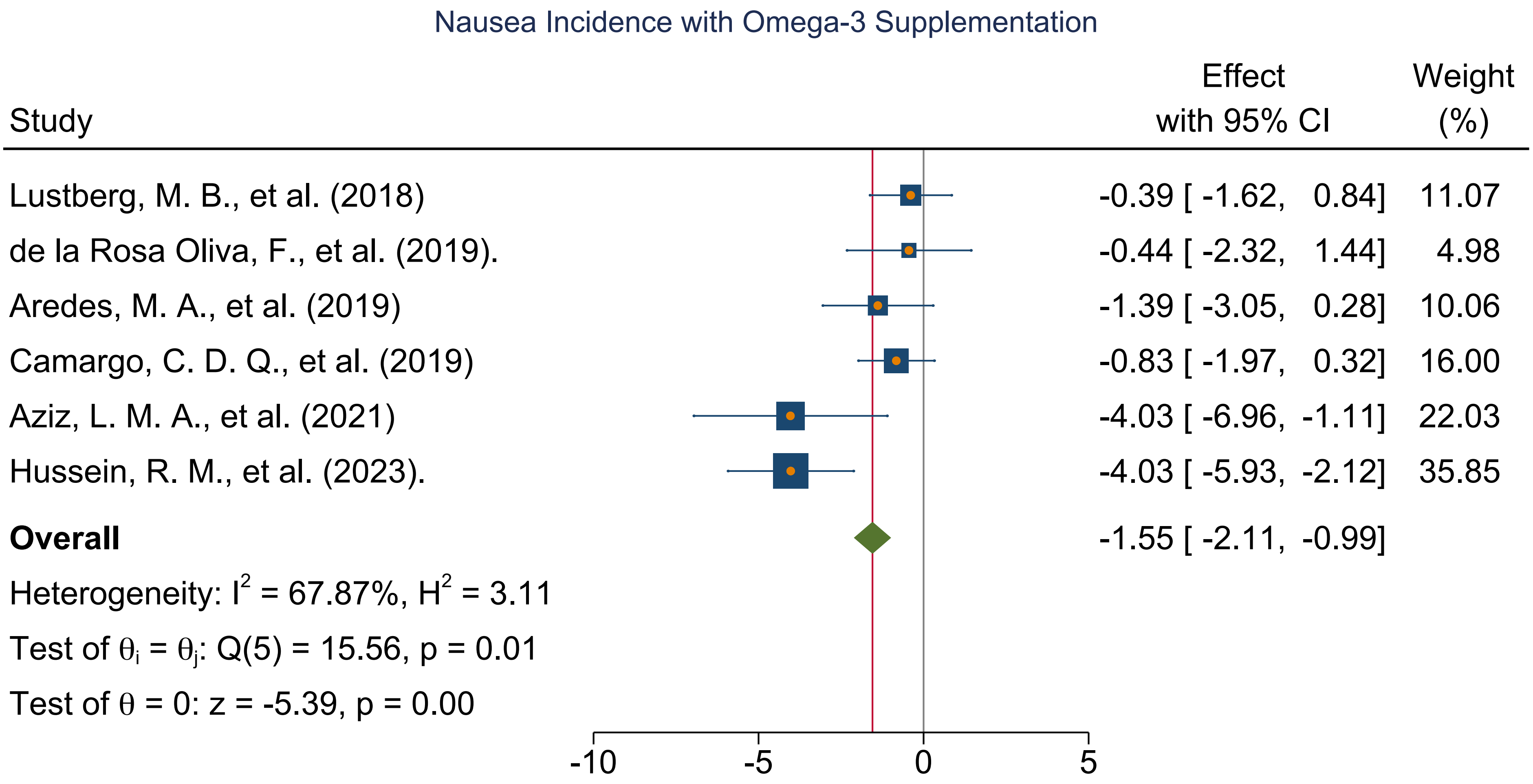

Nausea Incidence with Omega-3 Supplementation
Study
Effect with 95% CI
Weight (%)
Lustberg, M. B., et al. (2018) -0.39 [-1.62, 0.84] 11.07
de la Rosa Oliva, F., et al. (2019). -0.44 [-2.32, 1.44] 4.98
Aredes, M. A., et al. (2019) -1.39 [-3.05, 0.28] 10.06
Camargo, C. D. Q., et al. (2019) -0.83 [-1.97, 0.32] 16.00
Aziz, L. M. A., et al. (2021) -4.03 [-6.96, -1.11] 22.03
Hussein, R. M., et al. (2023). -4.03 [-5.93, -2.12] 35.85
Overall -1.55 [-2.11, -0.99]
Heterogeneity: I^2 = 67.87%, H^2 = 3.11
Test of $\theta_i = \theta_j$: Q(5) = 15.56, p = 0.01
Test of θ = 0: z = -5.39, p = 0.00
-10
-5
0
5

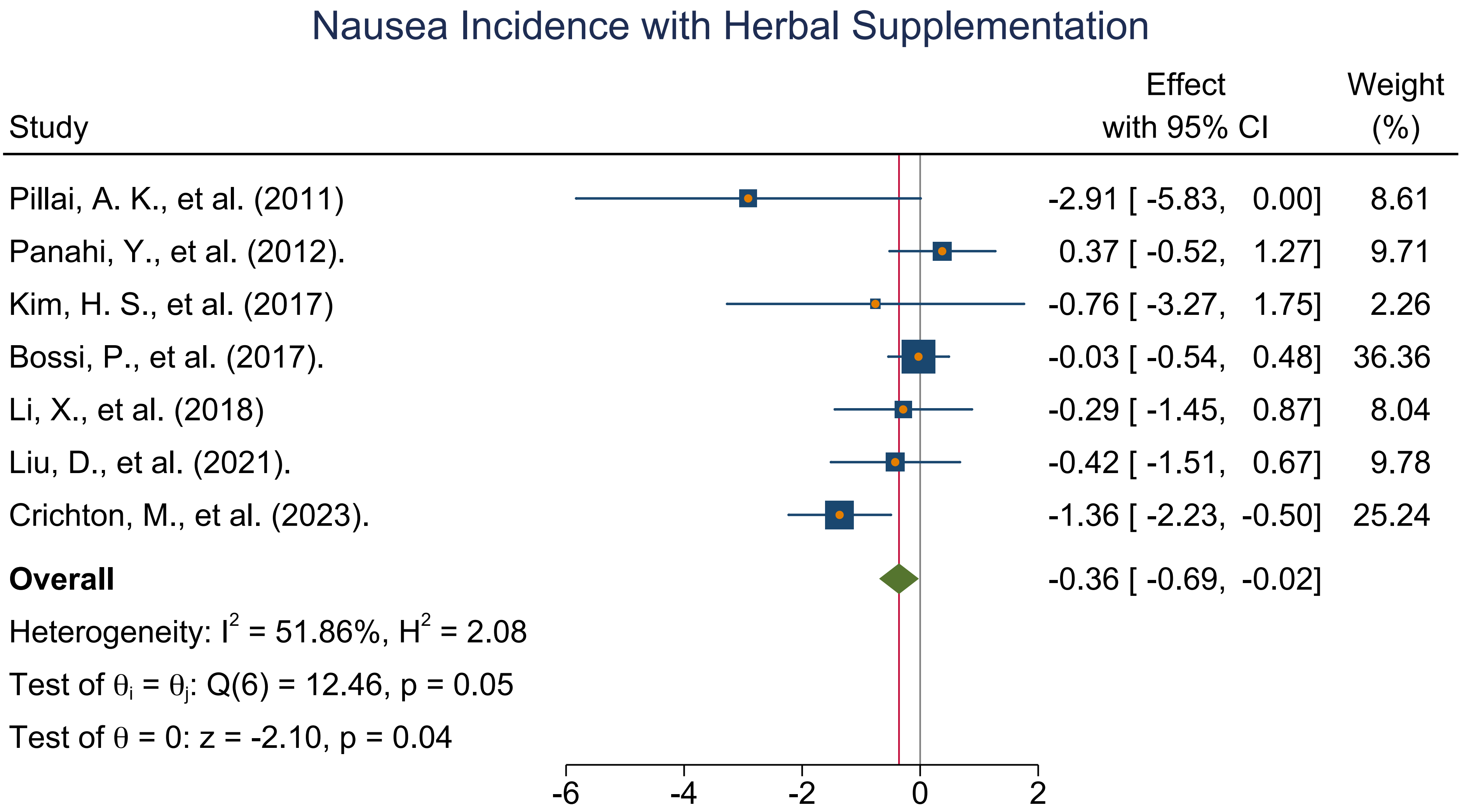
Nausea Incidence with Herbal Supplementation
Study
Effect with 95% CI
Weight (%)
Pillai, A. K., et al. (2011) -2.91 [-5.83, 0.00] 8.61
Panahi, Y., et al. (2012). 0.37 [-0.52, 1.27] 9.71
Kim, H. S., et al. (2017) -0.76 [-3.27, 1.75] 2.26
Bossi, P., et al. (2017). -0.03 [-0.54, 0.48] 36.36
Li, X., et al. (2018) -0.29 [-1.45, 0.87] 8.04
Liu, D., et al. (2021). -0.42 [-1.51, 0.67] 9.78
Crichton, M., et al. (2023). -1.36 [-2.23, -0.50] 25.24
Overall -0.36 [-0.69, -0.02]
Heterogeneity: I² = 51.86%, H² = 2.08
Test of θi = θj: Q(6) = 12.46, p = 0.05
Test of θ = 0: z = -2.10, p = 0.04
-6
-4
-2
0
2

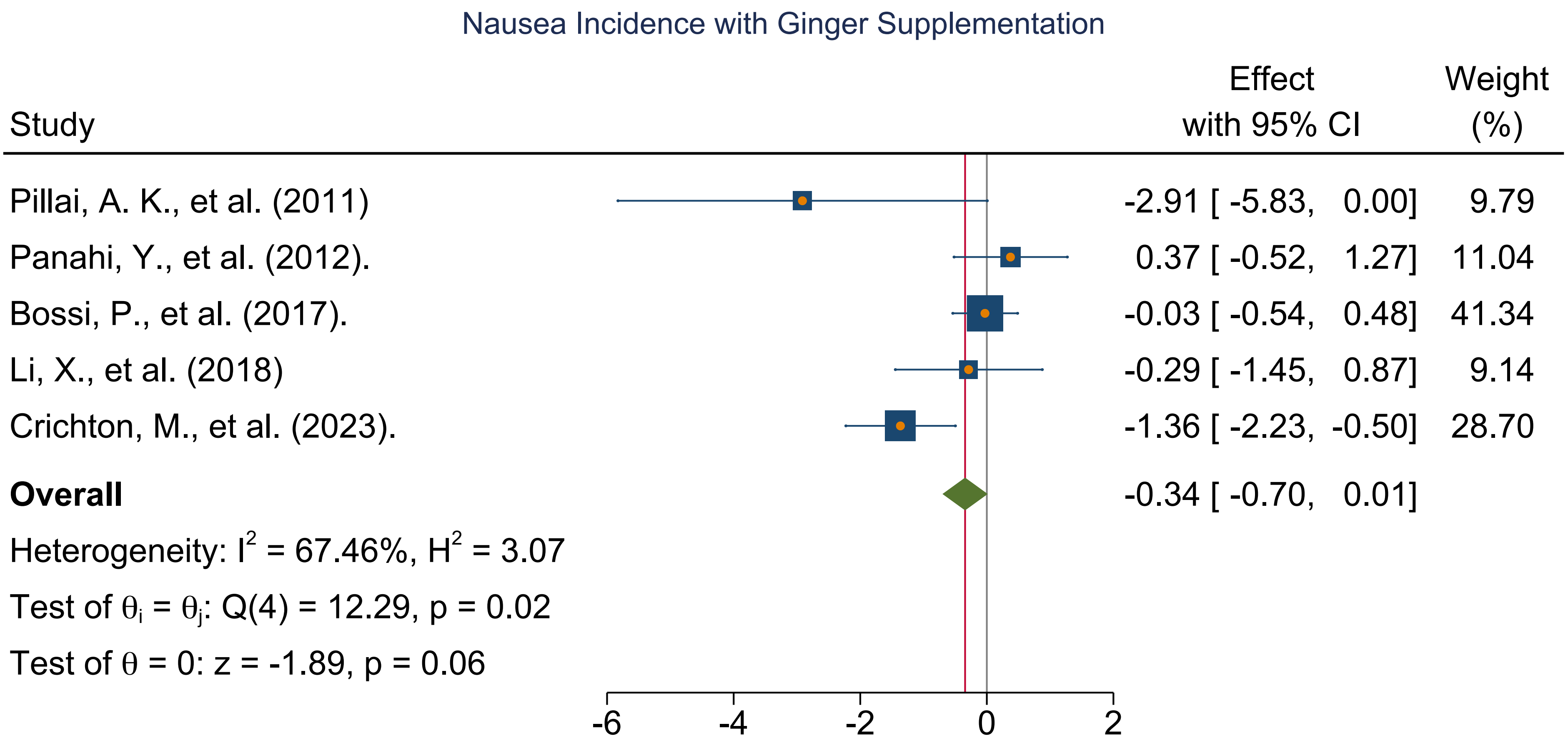
Nausea Incidence with Ginger Supplementation
Study
Effect with 95% CI
Weight (%)
Pillai, A. K., et al. (2011) -2.91 [-5.83, 0.00] 9.79
Panahi, Y., et al. (2012). 0.37 [-0.52, 1.27] 11.04
Bossi, P., et al. (2017). -0.03 [-0.54, 0.48] 41.34
Li, X., et al. (2018) -0.29 [-1.45, 0.87] 9.14
Crichton, M., et al. (2023). -1.36 [-2.23, -0.50] 28.70
Overall -0.34 [-0.70, 0.01]
Heterogeneity: I^2 = 67.46%, H^2 = 3.07
Test of $\theta_i = \theta_j$: Q(4) = 12.29, p = 0.02
Test of θ = 0: z = -1.89, p = 0.06
-6
-4
-2
0
2

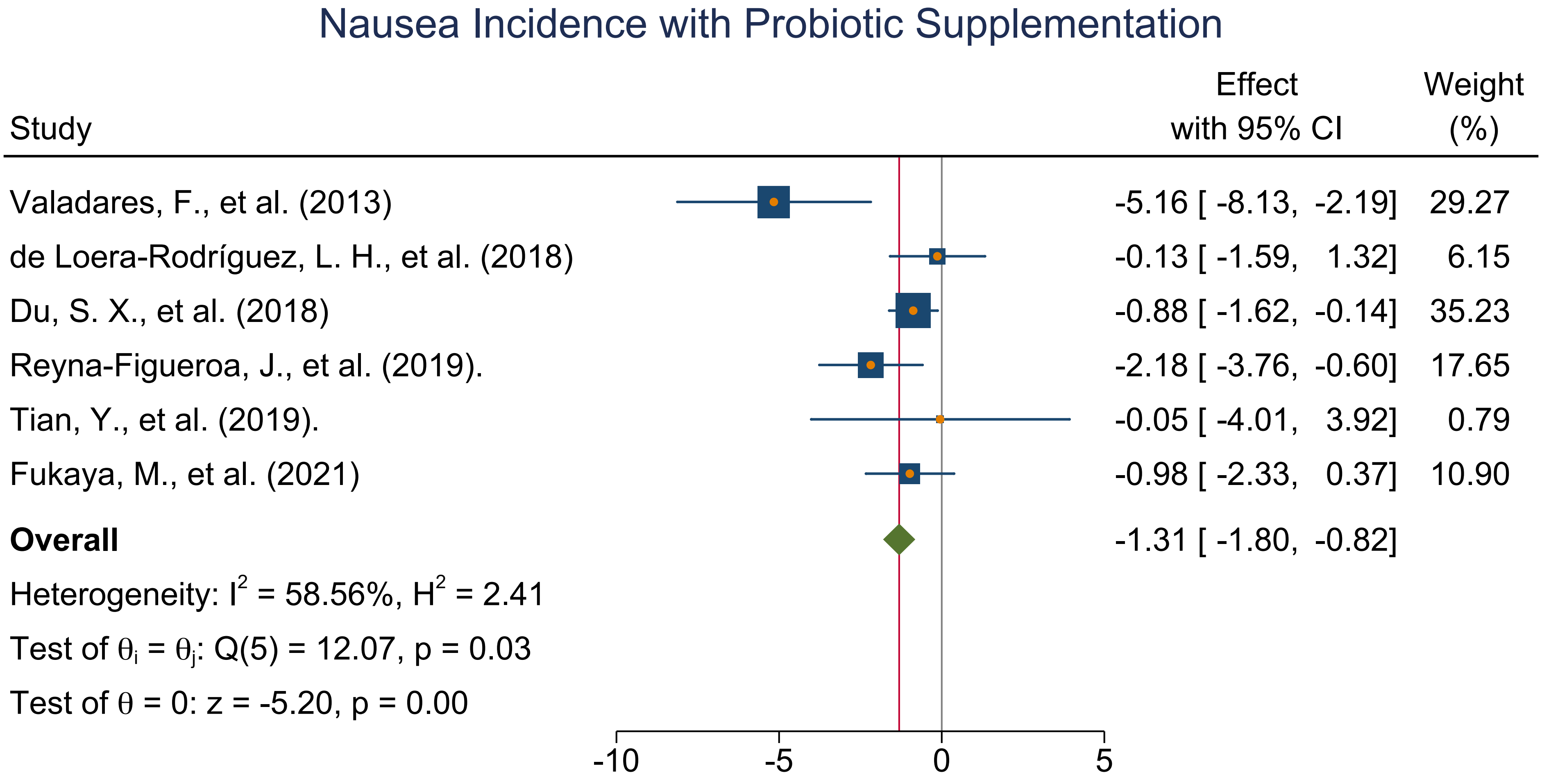

Nausea Incidence with Probiotic Supplementation
Study
Effect with 95% CI
Weight (%)
Valadares, F., et al. (2013) -5.16 [-8.13, -2.19] 29.27
de Loera-Rodríguez, L. H., et al. (2018) -0.13 [-1.59, 1.32] 6.15
Du, S. X., et al. (2018) -0.88 [-1.62, -0.14] 35.23
Reyna-Figueroa, J., et al. (2019). -2.18 [-3.76, -0.60] 17.65
Tian, Y., et al. (2019). -0.05 [-4.01, 3.92] 0.79
Fukaya, M., et al. (2021) -0.98 [-2.33, 0.37] 10.90
Overall -1.31 [-1.80, -0.82]
Heterogeneity: I^2 = 58.56%, H^2 = 2.41
Test of $\theta_i = \theta_j$: Q(5) = 12.07, p = 0.03
Test of θ = 0: z = -5.20, p = 0.00
-10
-5
0
5

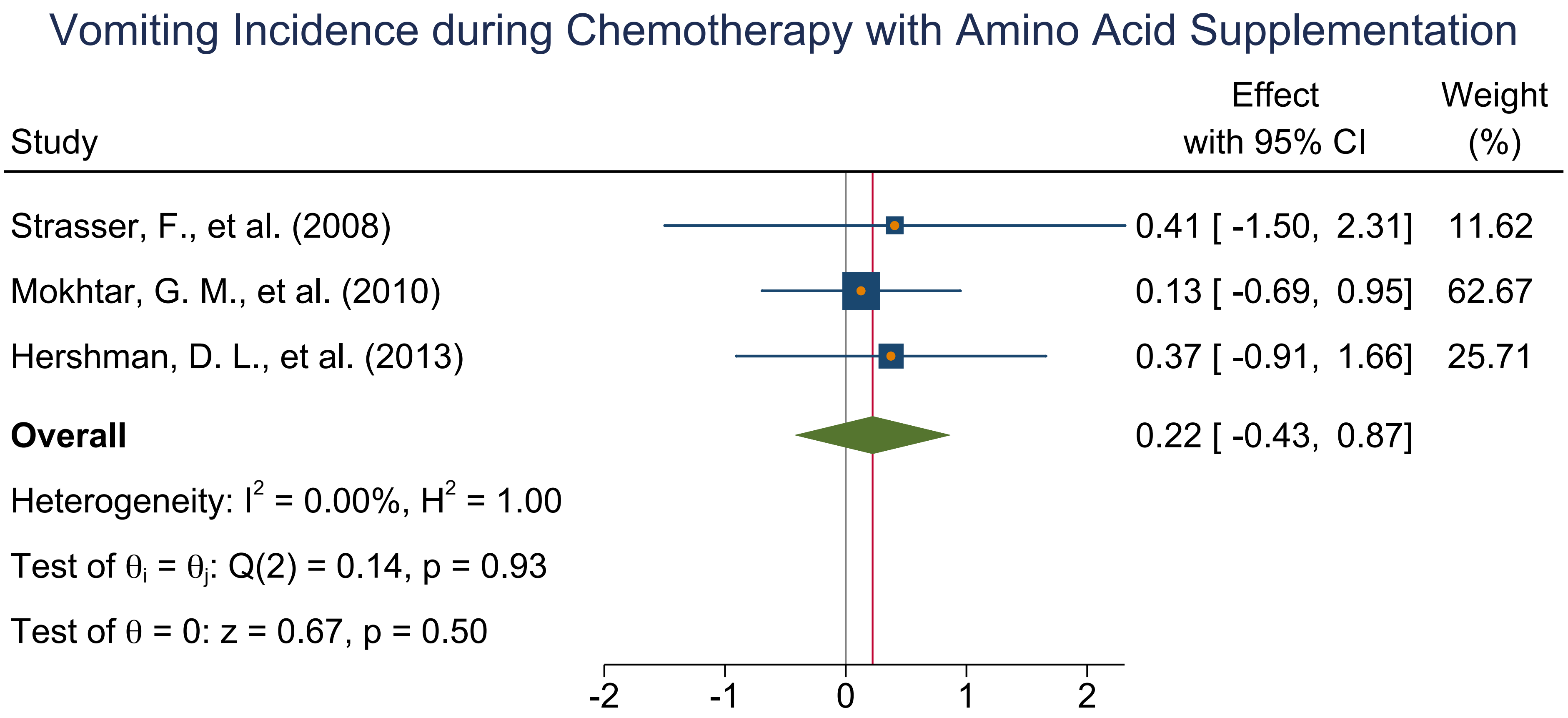

Vomiting Incidence during Chemotherapy with Amino Acid Supplementation
Study
Effect with 95% CI
Weight (%)
Strasser, F., et al. (2008) 0.41 [-1.50, 2.31] 11.62
Mokhtar, G. M., et al. (2010) 0.13 [-0.69, 0.95] 62.67
Hershman, D. L., et al. (2013) 0.37 [-0.91, 1.66] 25.71
Overall 0.22 [-0.43, 0.87]
Heterogeneity: I^2 = 0.00%, H^2 = 1.00
Test of $\theta_i = \theta_j$: Q(2) = 0.14, p = 0.93
Test of θ = 0: z = 0.67, p = 0.50
-2
-1
0
1
2

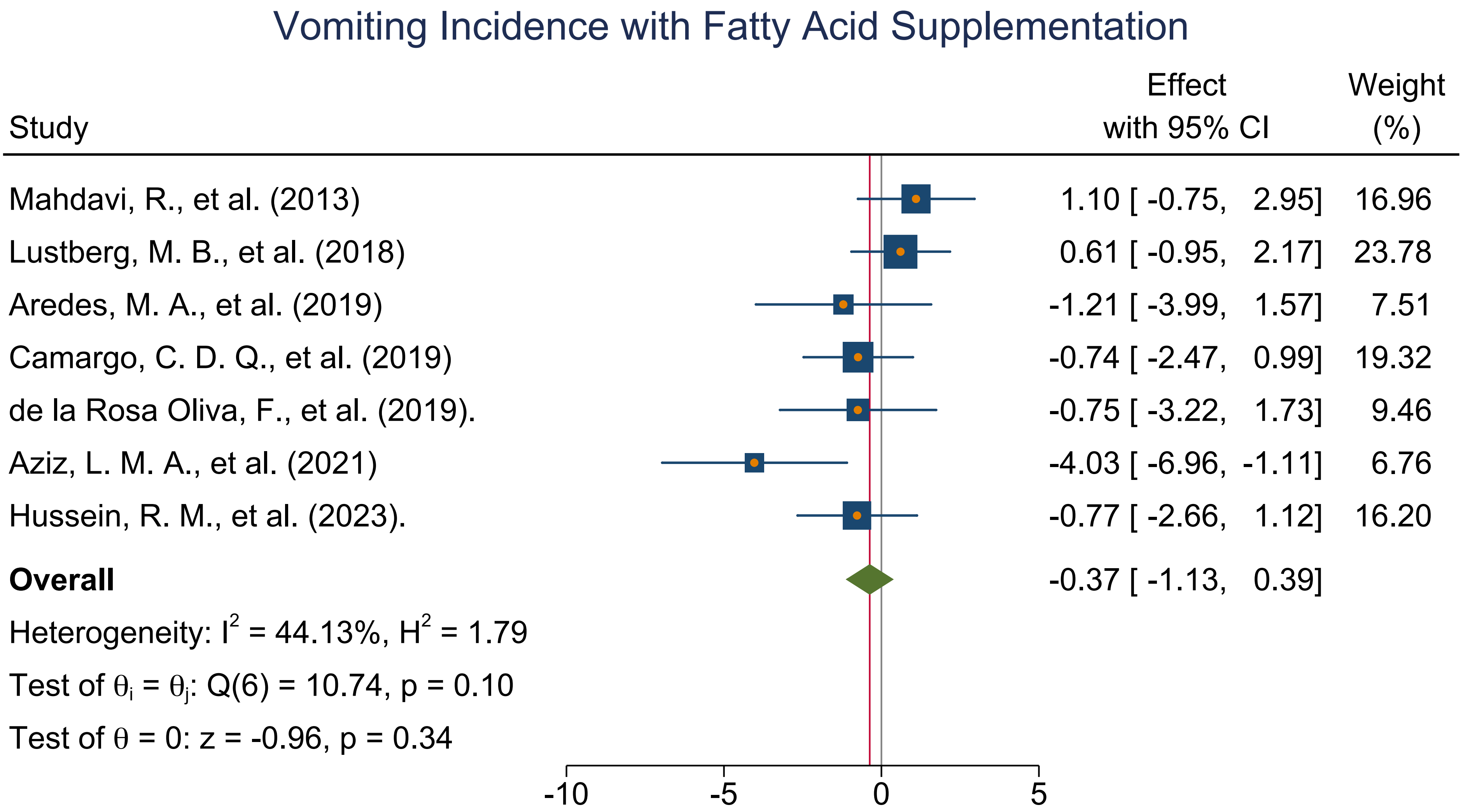
Vomiting Incidence with Fatty Acid Supplementation
Study
Effect with 95% CI
Weight (%)
Mahdavi, R., et al. (2013) 1.10 [-0.75, 2.95] 16.96
Lustberg, M. B., et al. (2018) 0.61 [-0.95, 2.17] 23.78
Aredes, M. A., et al. (2019) -1.21 [-3.99, 1.57] 7.51
Camargo, C. D. Q., et al. (2019) -0.74 [-2.47, 0.99] 19.32
de la Rosa Oliva, F., et al. (2019). -0.75 [-3.22, 1.73] 9.46
Aziz, L. M. A., et al. (2021) -4.03 [-6.96, -1.11] 6.76
Hussein, R. M., et al. (2023). -0.77 [-2.66, 1.12] 16.20
Overall -0.37 [-1.13, 0.39]
Heterogeneity: I^2 = 44.13%, H^2 = 1.79
Test of $\theta_i = \theta_j$: Q(6) = 10.74, p = 0.10
Test of θ = 0: z = -0.96, p = 0.34
-10
-5
0
5

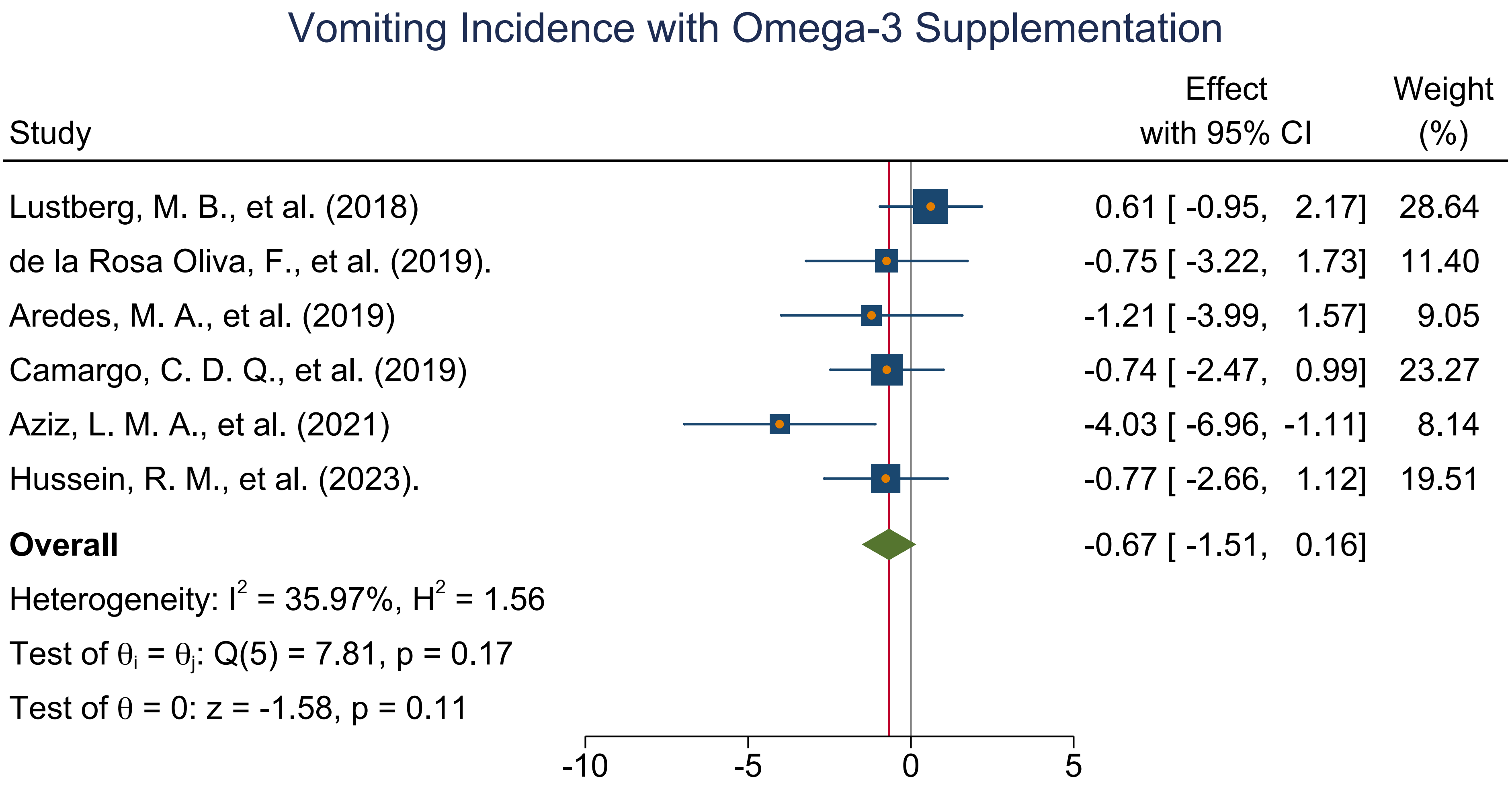
Vomiting Incidence with Omega-3 Supplementation
Study
Effect with 95% CI
Weight (%)
Lustberg, M. B., et al. (2018) 0.61 [-0.95, 2.17] 28.64
de la Rosa Oliva, F., et al. (2019). -0.75 [-3.22, 1.73] 11.40
Aredes, M. A., et al. (2019) -1.21 [-3.99, 1.57] 9.05
Camargo, C. D. Q., et al. (2019) -0.74 [-2.47, 0.99] 23.27
Aziz, L. M. A., et al. (2021) -4.03 [-6.96, -1.11] 8.14
Hussein, R. M., et al. (2023). -0.77 [-2.66, 1.12] 19.51
Overall -0.67 [-1.51, 0.16]
Heterogeneity: I^2 = 35.97%, H^2 = 1.56
Test of $\theta_i = \theta_j$: Q(5) = 7.81, p = 0.17
Test of θ = 0: z = -1.58, p = 0.11
-10
-5
0
5

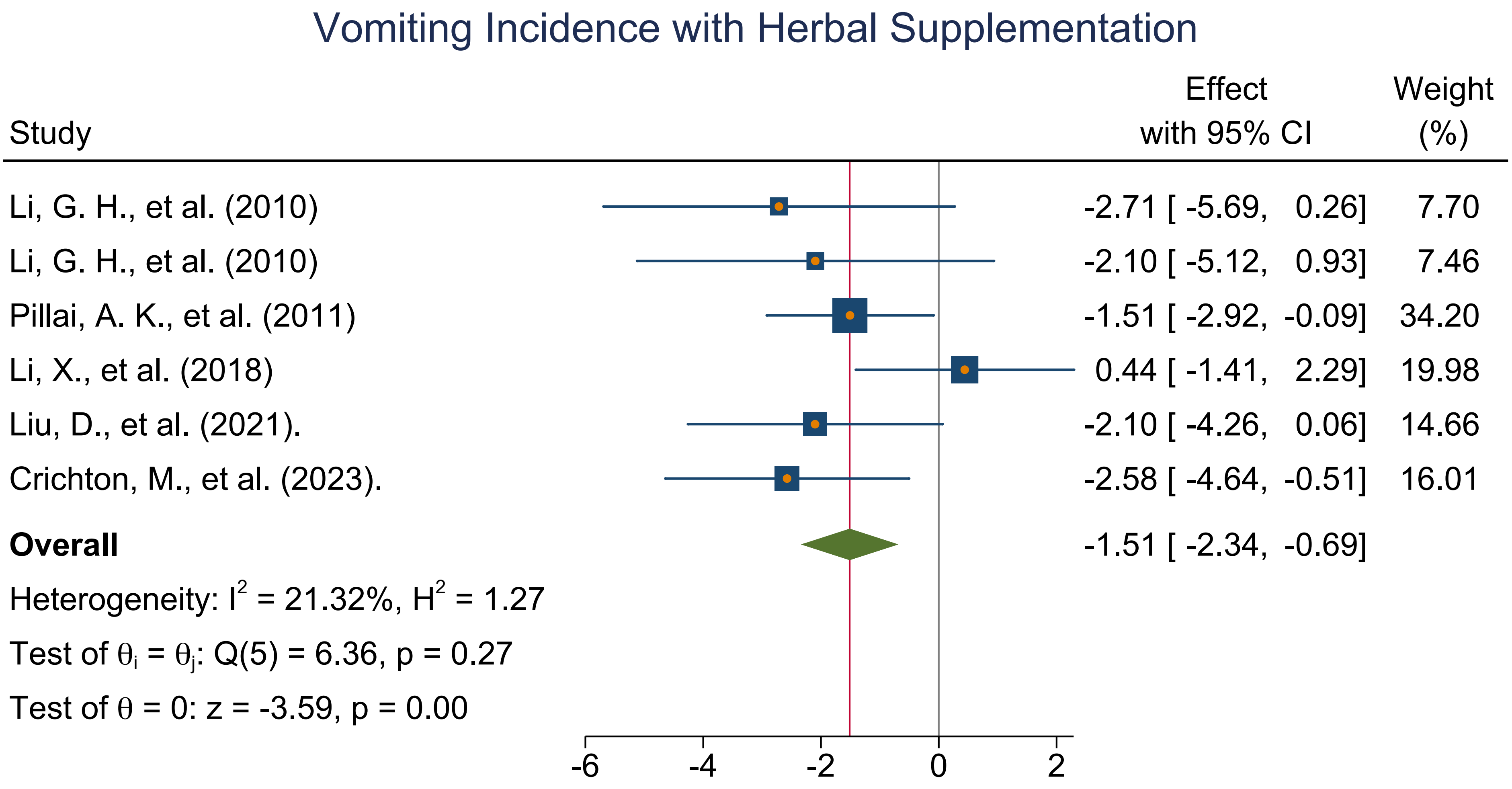

Vomiting Incidence with Herbal Supplementation
Study
Effect with 95% CI
Weight (%)
Li, G. H., et al. (2010) -2.71 [-5.69, 0.26] 7.70
Li, G. H., et al. (2010) -2.10 [-5.12, 0.93] 7.46
Pillai, A. K., et al. (2011) -1.51 [-2.92, -0.09] 34.20
Li, X., et al. (2018) 0.44 [-1.41, 2.29] 19.98
Liu, D., et al. (2021). -2.10 [-4.26, 0.06] 14.66
Crichton, M., et al. (2023). -2.58 [-4.64, -0.51] 16.01
Overall -1.51 [-2.34, -0.69]
Heterogeneity: I^2 = 21.32%, H^2 = 1.27
Test of $\theta_i = \theta_j$: Q(5) = 6.36, p = 0.27
Test of θ = 0: z = -3.59, p = 0.00
-6
-4
-2
0
2

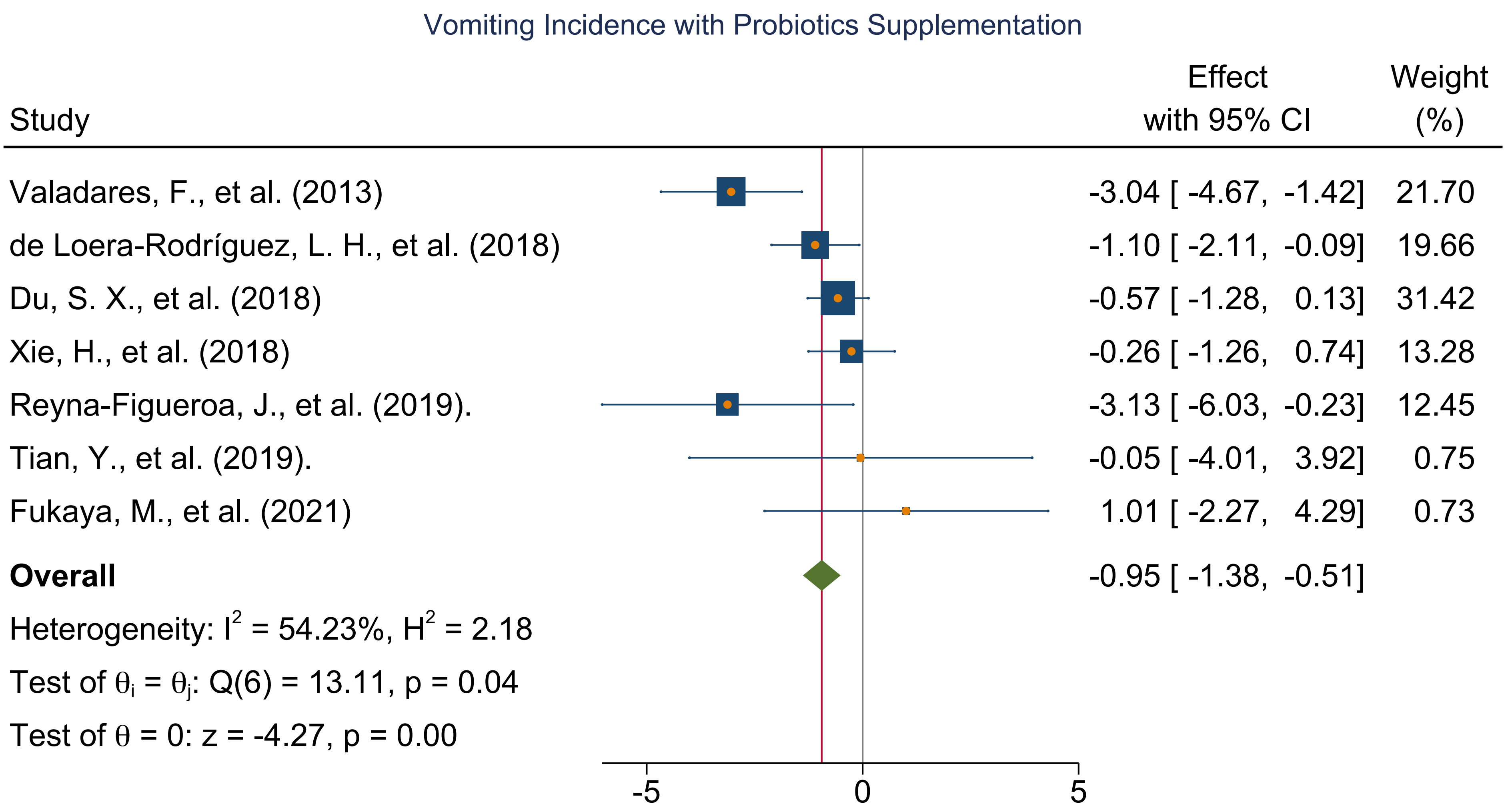
Vomiting Incidence with Probiotics Supplementation
Study
Effect with 95% CI
Weight (%)
Valadares, F., et al. (2013) -3.04 [-4.67, -1.42] 21.70
de Loera-Rodríguez, L. H., et al. (2018) -1.10 [-2.11, -0.09] 19.66
Du, S. X., et al. (2018) -0.57 [-1.28, 0.13] 31.42
Xie, H., et al. (2018) -0.26 [-1.26, 0.74] 13.28
Reyna-Figueroa, J., et al. (2019). -3.13 [-6.03, -0.23] 12.45
Tian, Y., et al. (2019). -0.05 [-4.01, 3.92] 0.75
Fukaya, M., et al. (2021) 1.01 [-2.27, 4.29] 0.73
Overall -0.95 [-1.38, -0.51]
Heterogeneity: I^2 = 54.23%, H^2 = 2.18
Test of $\theta_i = \theta_j$: Q(6) = 13.11, p = 0.04
Test of θ = 0: z = -4.27, p = 0.00
-5
0
5

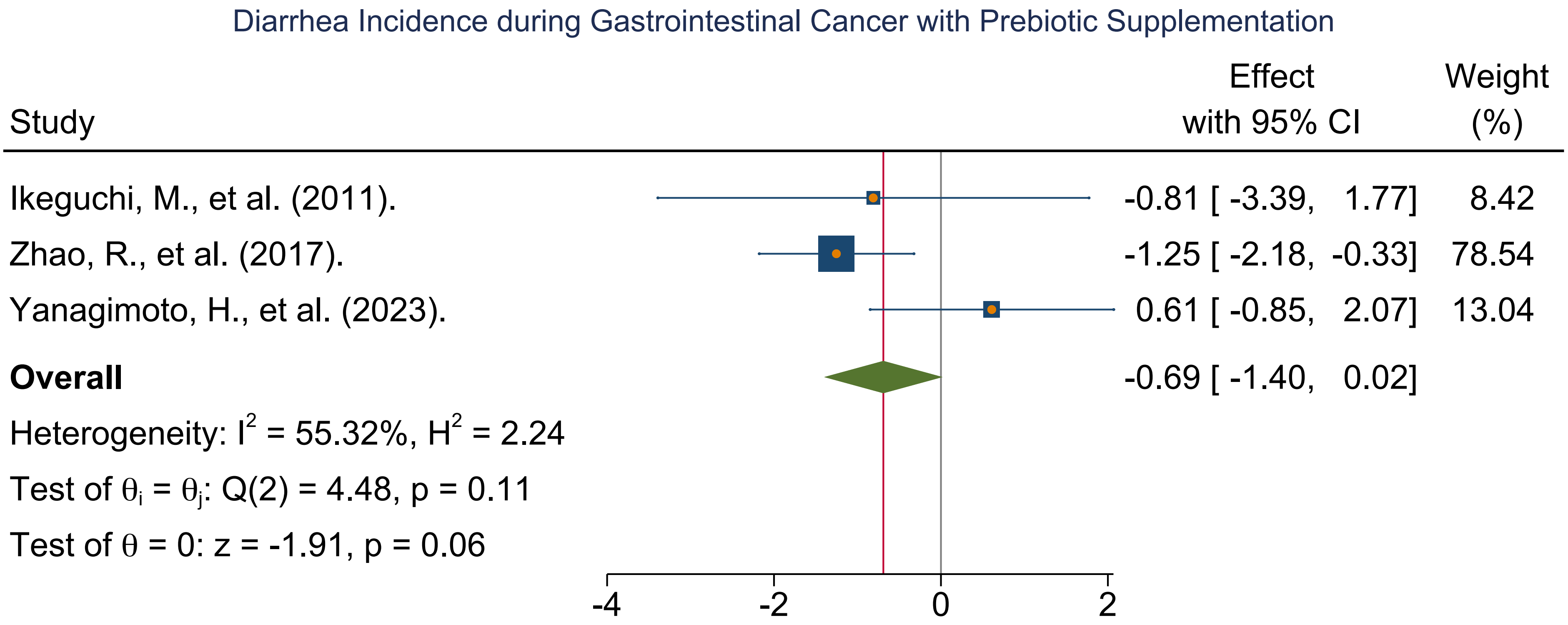
Diarrhea Incidence during Gastrointestinal Cancer with Prebiotic Supplementation
Study
Effect with 95% CI
Weight (%)
Ikeguchi, M., et al. (2011). -0.81 [-3.39, 1.77] 8.42
Zhao, R., et al. (2017). -1.25 [-2.18, -0.33] 78.54
Yanagimoto, H., et al. (2023). 0.61 [-0.85, 2.07] 13.04
Overall -0.69 [-1.40, 0.02]
Heterogeneity: I² = 55.32%, H² = 2.24
Test of θi = θj: Q(2) = 4.48, p = 0.11
Test of θ = 0: z = -1.91, p = 0.06
-4
-2
0
2

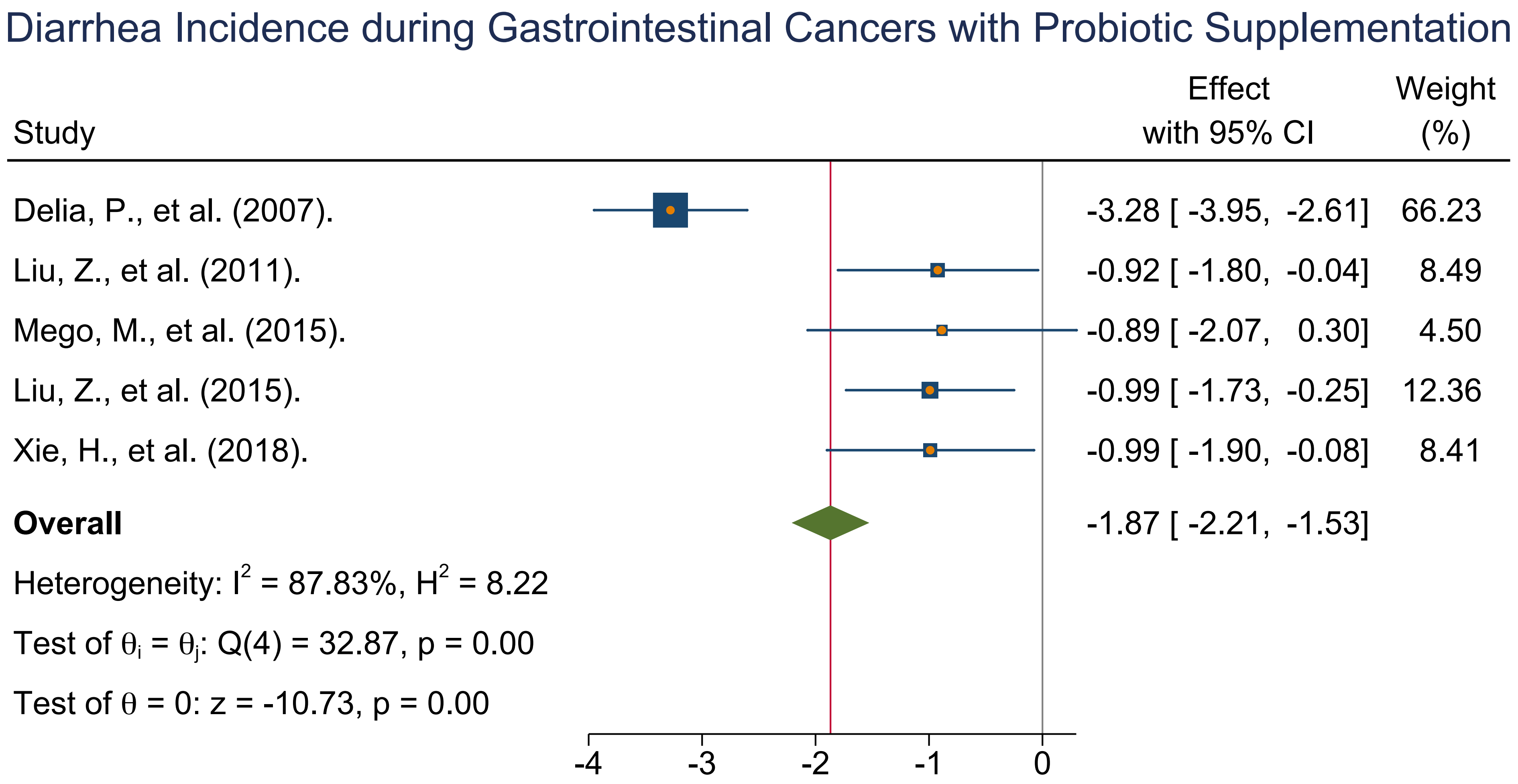
Diarrhea Incidence during Gastrointestinal Cancers with Probiotic Supplementation
Study
Effect with 95% CI
Weight (%)
Delia, P., et al. (2007). -3.28 [-3.95, -2.61] 66.23
Liu, Z., et al. (2011). -0.92 [-1.80, -0.04] 8.49
Mego, M., et al. (2015). -0.89 [-2.07, 0.30] 4.50
Liu, Z., et al. (2015). -0.99 [-1.73, -0.25] 12.36
Xie, H., et al. (2018). -0.99 [-1.90, -0.08] 8.41
Overall -1.87 [-2.21, -1.53]
Heterogeneity: I^2 = 87.83%, H^2 = 8.22
Test of $\theta_i = \theta_j$: Q(4) = 32.87, p = 0.00
Test of θ = 0: z = -10.73, p = 0.00
-4
-3
-2
-1
0

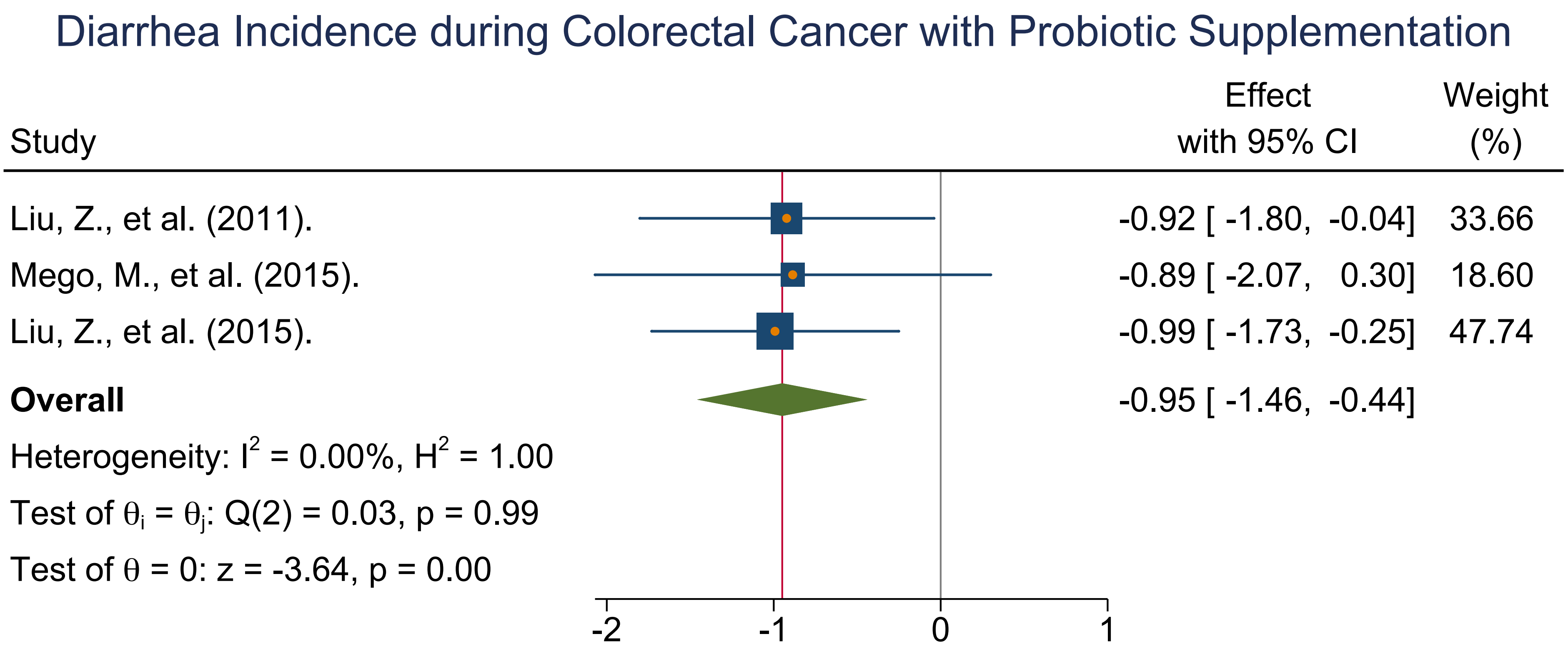
Diarrhea Incidence during Colorectal Cancer with Probiotic Supplementation
Study
Effect with 95% CI
Weight (%)
Liu, Z., et al. (2011). -0.92 [-1.80, -0.04] 33.66
Mego, M., et al. (2015). -0.89 [-2.07, 0.30] 18.60
Liu, Z., et al. (2015). -0.99 [-1.73, -0.25] 47.74
Overall -0.95 [-1.46, -0.44]
Heterogeneity: I^2 = 0.00%, H^2 = 1.00
Test of $\theta_i = \theta_j$: Q(2) = 0.03, p = 0.99
Test of θ = 0: z = -3.64, p = 0.00
-2
-1
0
1

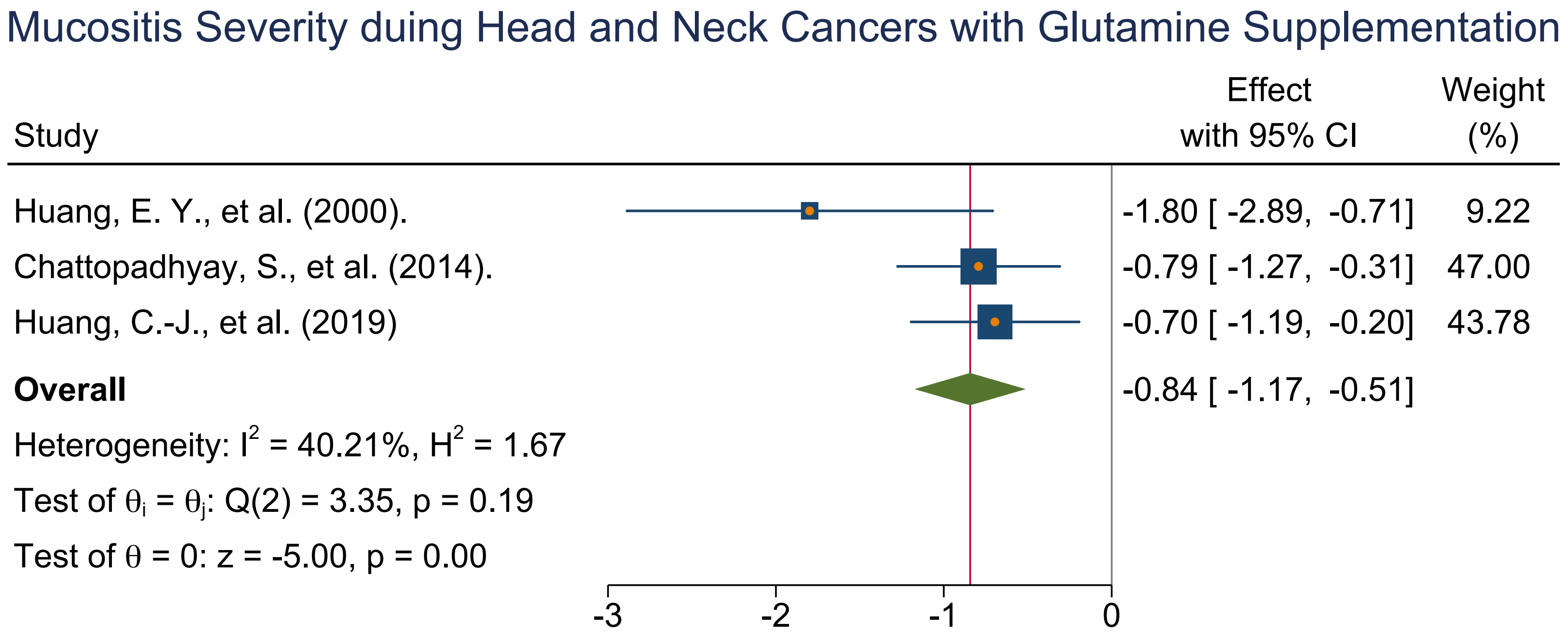
Mucositis Severity duing Head and Neck Cancers with Glutamine Supplementation
Study
Effect with 95% CI
Weight (%)
Huang, E. Y., et al. (2000).
-1.80 [-2.89, -0.71]
9.22
Chattopadhyay, S., et al. (2014).
-0.79 [-1.27, -0.31]
47.00
Huang, C.-J., et al. (2019)
-0.70 [-1.19, -0.20]
43.78
Overall
-0.84 [-1.17, -0.51]
Heterogeneity: I^2 = 40.21%, H^2 = 1.67
Test of $\theta_i = \theta_j$: Q(2) = 3.35, p = 0.19
Test of θ = 0: z = -5.00, p = 0.00
-3
-2
-1
0

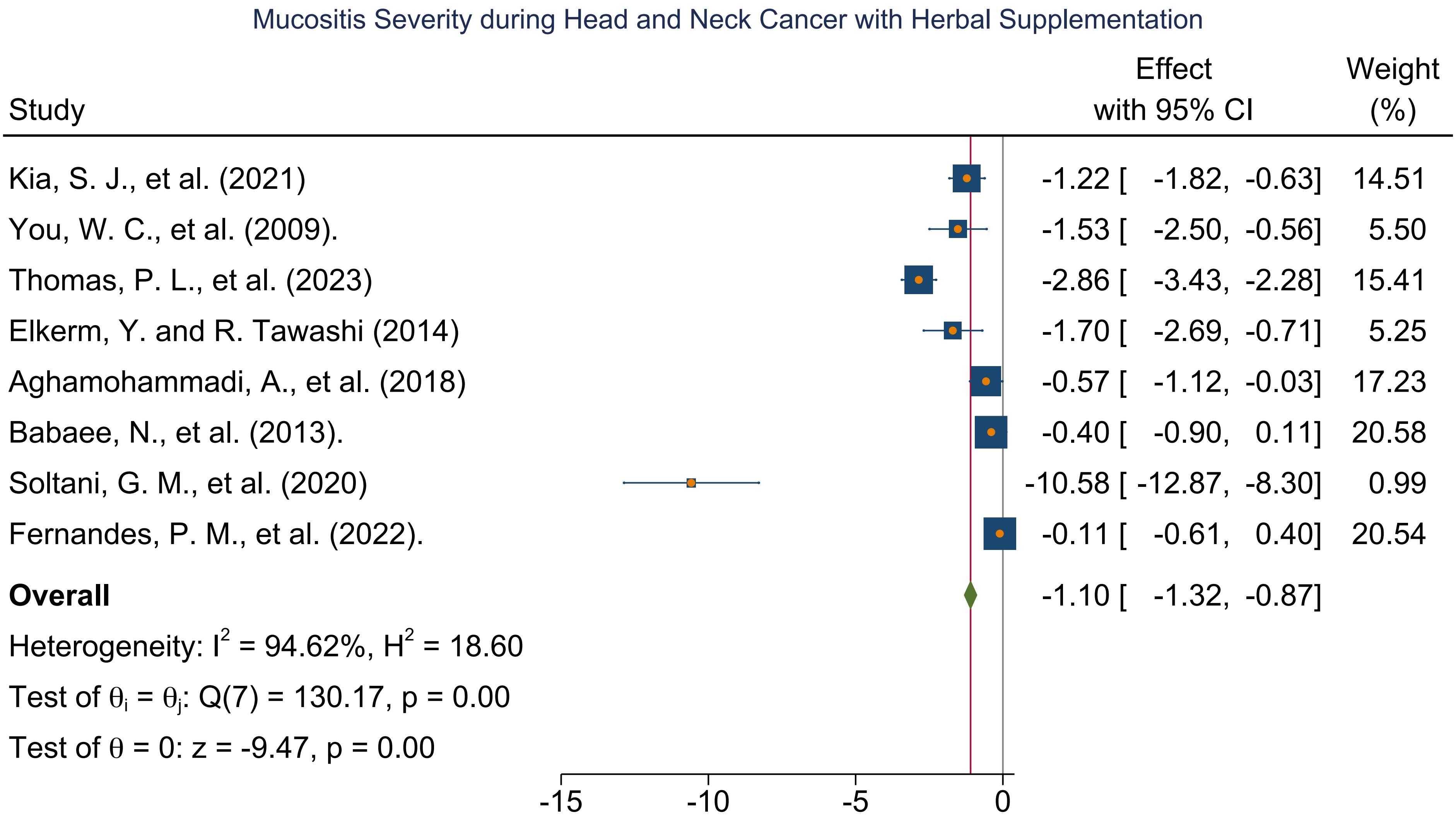
Mucositis Severity during Head and Neck Cancer with Herbal Supplementation
Study
Effect with 95% CI
Weight (%)
Kia, S. J., et al. (2021) -1.22 [-1.82, -0.63] 14.51
You, W. C., et al. (2009). -1.53 [-2.50, -0.56] 5.50
Thomas, P. L., et al. (2023) -2.86 [-3.43, -2.28] 15.41
Elkerm, Y. and R. Tawashi (2014) -1.70 [-2.69, -0.71] 5.25
Aghamohammadi, A., et al. (2018) -0.57 [-1.12, -0.03] 17.23
Babaee, N., et al. (2013). -0.40 [-0.90, 0.11] 20.58
Soltani, G. M., et al. (2020) -10.58 [-12.87, -8.30] 0.99
Fernandes, P. M., et al. (2022). -0.11 [-0.61, 0.40] 20.54
Overall -1.10 [-1.32, -0.87]
Heterogeneity: I^2 = 94.62%, H^2 = 18.60
Test of $\theta_i = \theta_j$: Q(7) = 130.17, p = 0.00
Test of θ = 0: z = -9.47, p = 0.00
-15
-10
-5
0

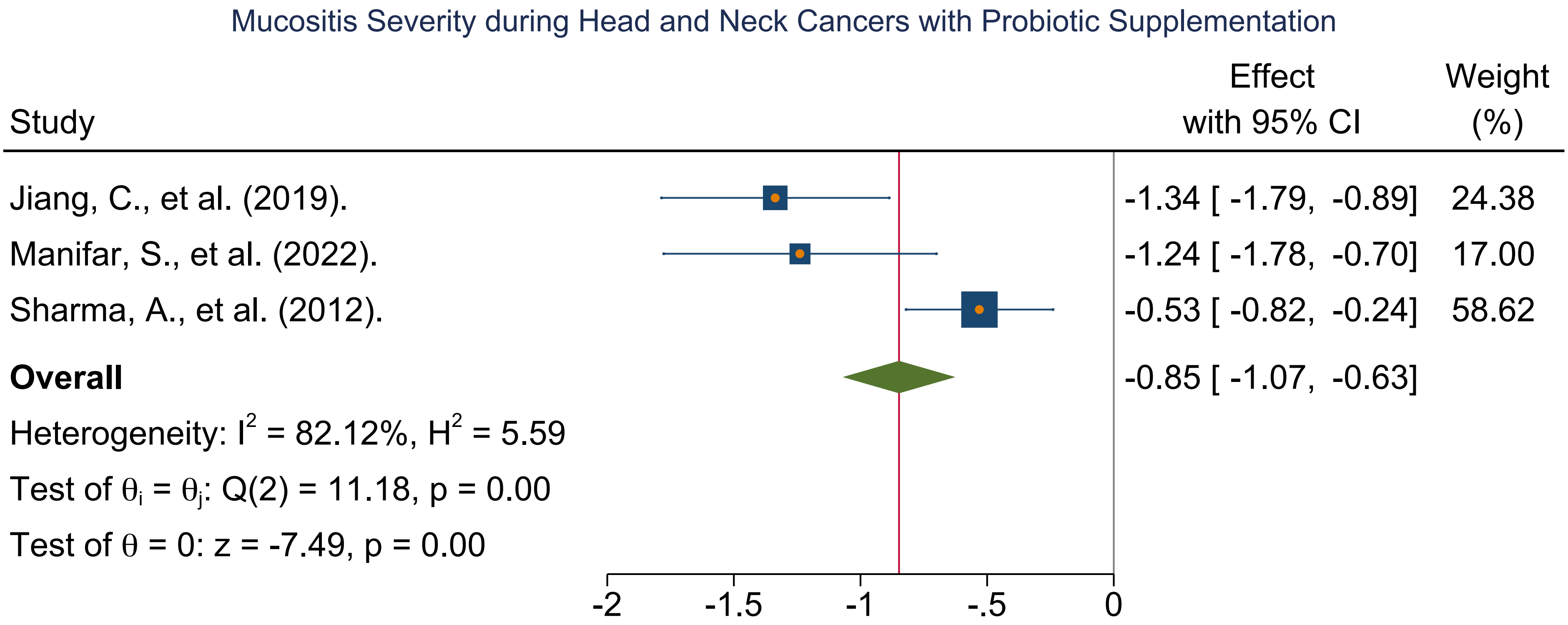
Mucositis Severity during Head and Neck Cancers with Probiotic Supplementation
Study
Effect with 95% CI
Weight (%)
Jiang, C., et al. (2019).
-1.34 [-1.79, -0.89]
24.38
Manifar, S., et al. (2022).
-1.24 [-1.78, -0.70]
17.00
Sharma, A., et al. (2012).
-0.53 [-0.82, -0.24]
58.62
Overall
-0.85 [-1.07, -0.63]
Heterogeneity: I2 = 82.12%, H2 = 5.59
Test of θi = θj: Q(2) = 11.18, p = 0.00
Test of θ = 0: z = -7.49, p = 0.00
-2
-1.5
-1
-.5
0

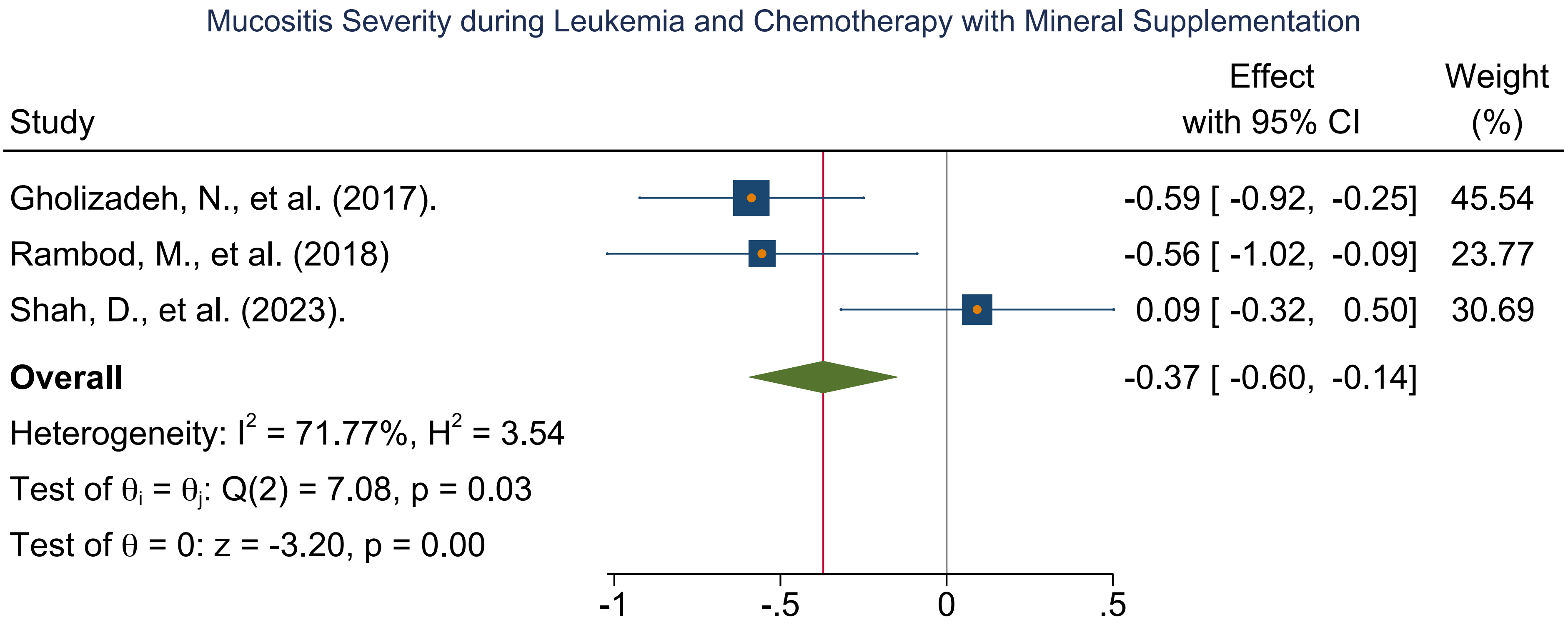
Mucositis Severity during Leukemia and Chemotherapy with Mineral Supplementation
Study
Effect with 95% CI
Weight (%)
Gholizadeh, N., et al. (2017). -0.59 [-0.92, -0.25] 45.54
Rambod, M., et al. (2018) -0.56 [-1.02, -0.09] 23.77
Shah, D., et al. (2023). 0.09 [-0.32, 0.50] 30.69
Overall -0.37 [-0.60, -0.14]
Heterogeneity: I2 = 71.77%, H2 = 3.54
Test of θi = θj: Q(2) = 7.08, p = 0.03
Test of θ = 0: z = -3.20, p = 0.00
-1
-.5
0
.5

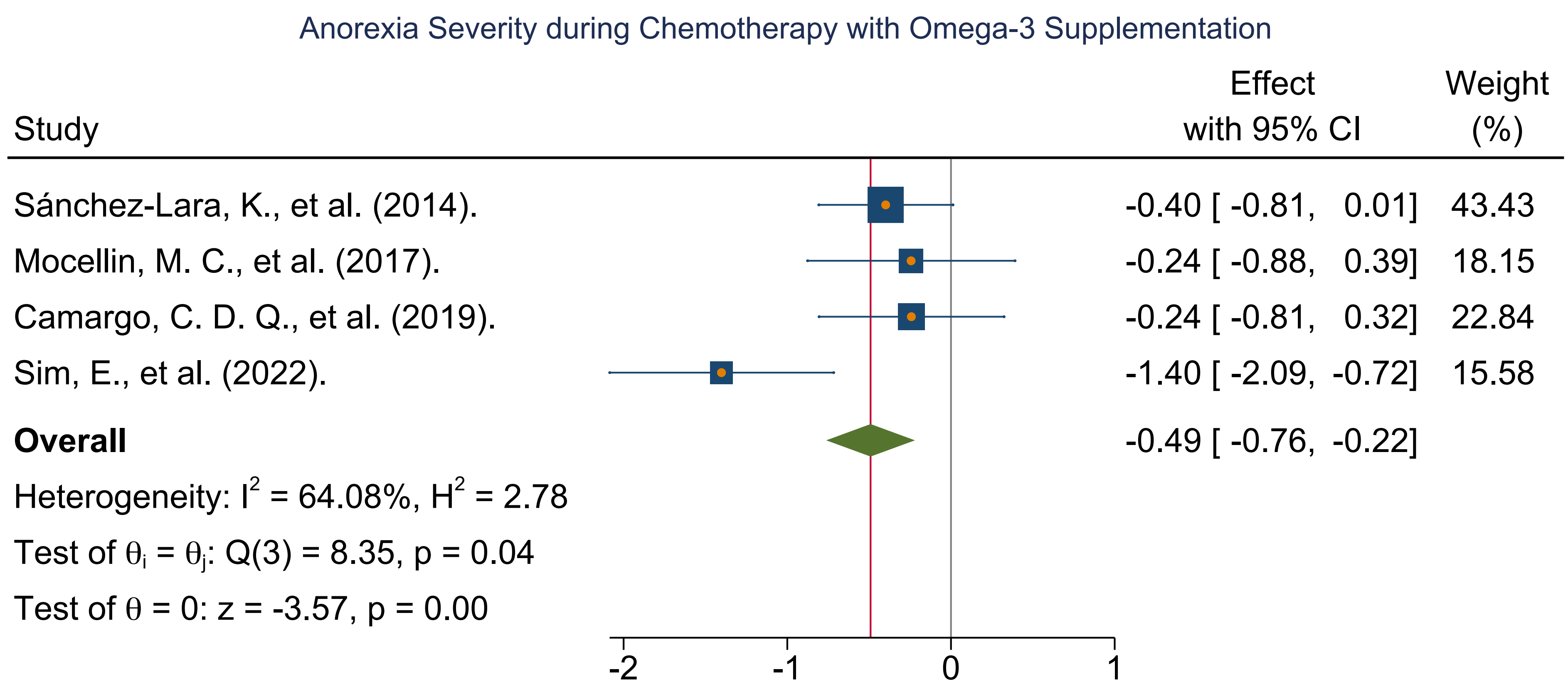

Anorexia Severity during Chemotherapy with Omega-3 Supplementation
Study
Effect with 95% CI
Weight (%)
Sánchez-Lara, K., et al. (2014). -0.40 [-0.81, 0.01] 43.43
Mocellin, M. C., et al. (2017). -0.24 [-0.88, 0.39] 18.15
Camargo, C. D. Q., et al. (2019). -0.24 [-0.81, 0.32] 22.84
Sim, E., et al. (2022). -1.40 [-2.09, -0.72] 15.58
Overall -0.49 [-0.76, -0.22]
Heterogeneity: I^2 = 64.08%, H^2 = 2.78
Test of $\theta_i = \theta_j$: Q(3) = 8.35, p = 0.04
Test of θ = 0: z = -3.57, p = 0.00
-2
-1
0
1

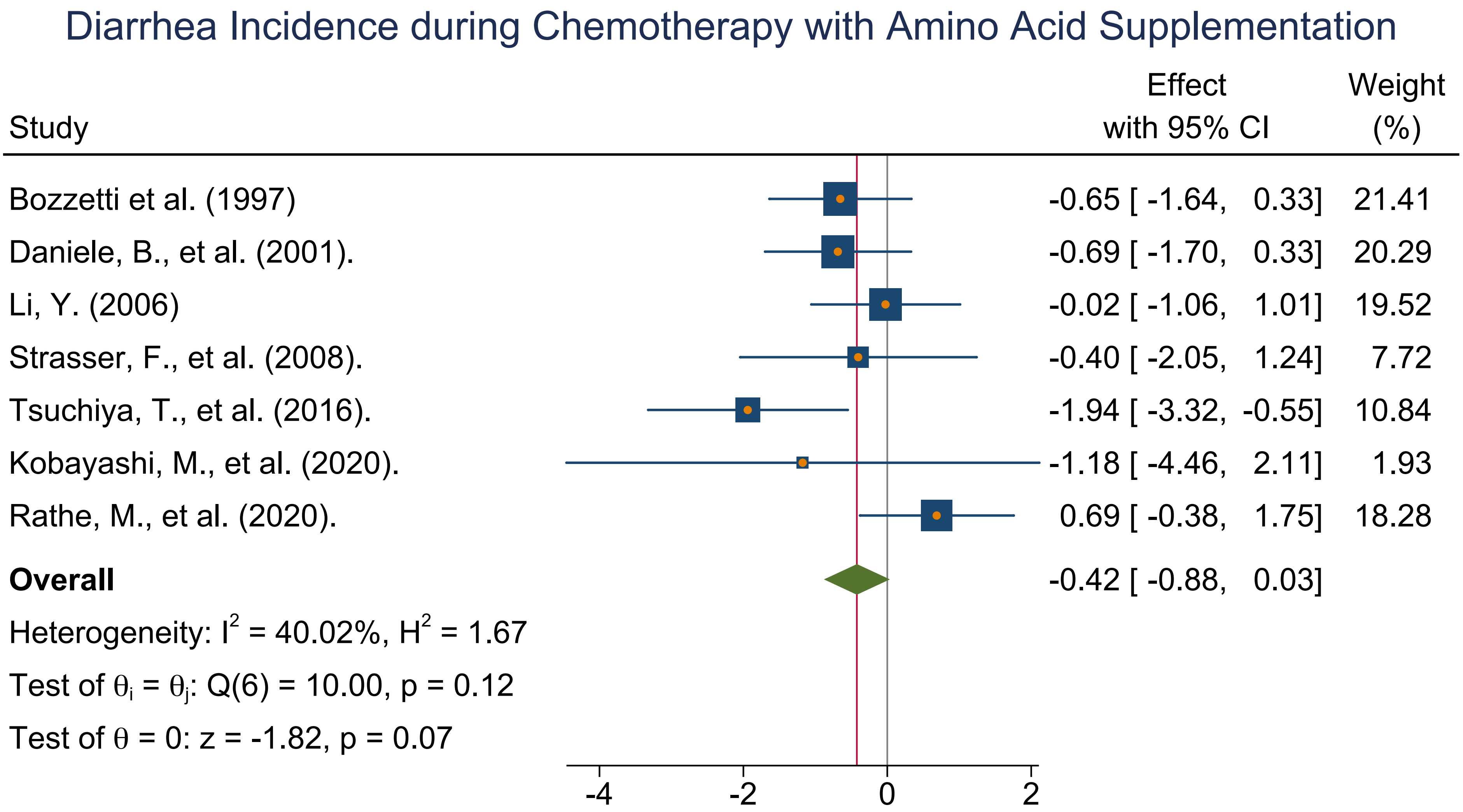
Diarrhea Incidence during Chemotherapy with Amino Acid Supplementation
Study
Effect with 95% CI
Weight (%)
Bozzetti et al. (1997) -0.65 [-1.64, 0.33] 21.41
Daniele, B., et al. (2001). -0.69 [-1.70, 0.33] 20.29
Li, Y. (2006) -0.02 [-1.06, 1.01] 19.52
Strasser, F., et al. (2008). -0.40 [-2.05, 1.24] 7.72
Tsuchiya, T., et al. (2016). -1.94 [-3.32, -0.55] 10.84
Kobayashi, M., et al. (2020). -1.18 [-4.46, 2.11] 1.93
Rathe, M., et al. (2020). 0.69 [-0.38, 1.75] 18.28
Overall -0.42 [-0.88, 0.03]
Heterogeneity: I^2 = 40.02%, H^2 = 1.67
Test of $\theta_i = \theta_j$: Q(6) = 10.00, p = 0.12
Test of θ = 0: z = -1.82, p = 0.07
-4
-2
0
2

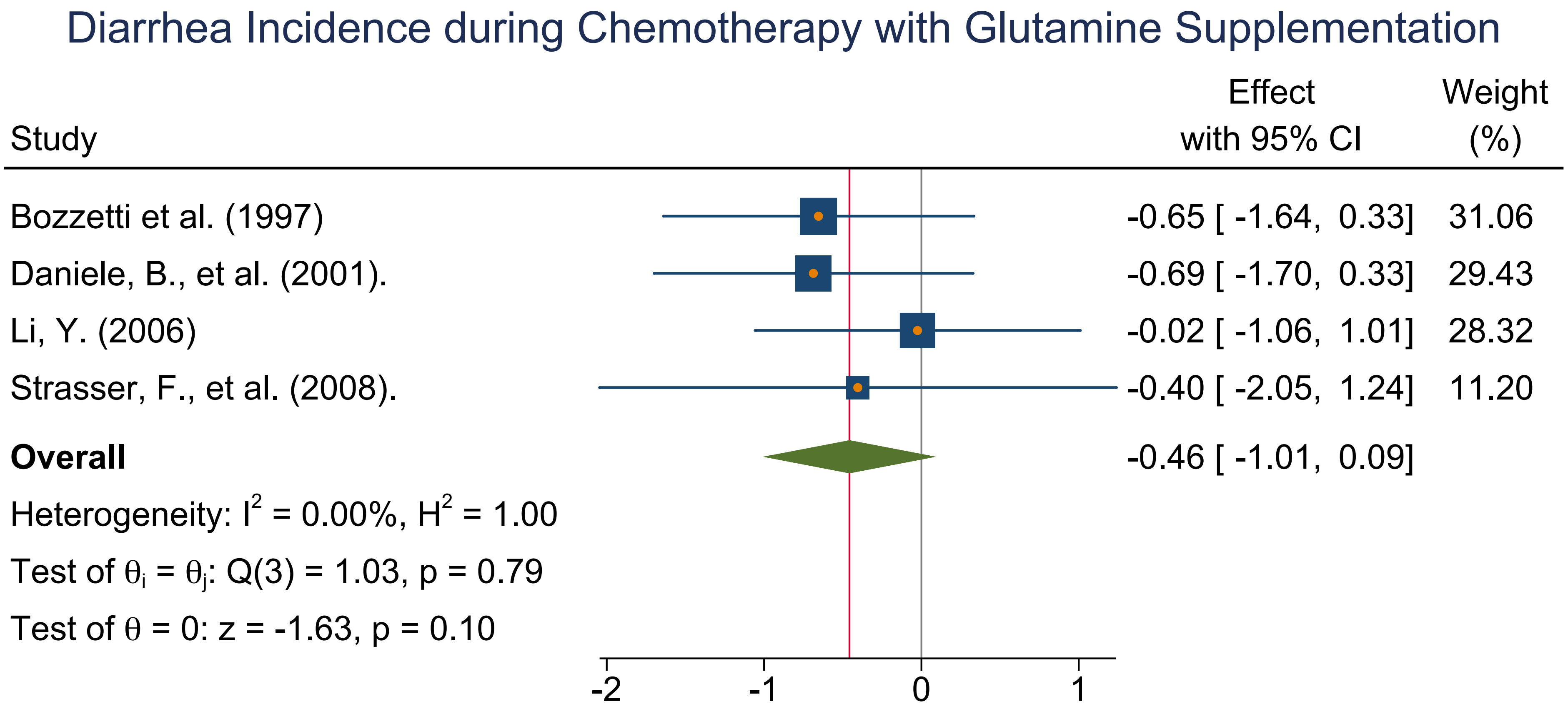
Diarrhea Incidence during Chemotherapy with Glutamine Supplementation
Study
Effect with 95% CI
Weight (%)
Bozzetti et al. (1997) -0.65 [-1.64, 0.33] 31.06
Daniele, B., et al. (2001). -0.69 [-1.70, 0.33] 29.43
Li, Y. (2006) -0.02 [-1.06, 1.01] 28.32
Strasser, F., et al. (2008). -0.40 [-2.05, 1.24] 11.20
Overall -0.46 [-1.01, 0.09]
Heterogeneity: I^2 = 0.00%, H^2 = 1.00
Test of $\theta_i = \theta_j$: Q(3) = 1.03, p = 0.79
Test of θ = 0: z = -1.63, p = 0.10
-2
-1
0
1

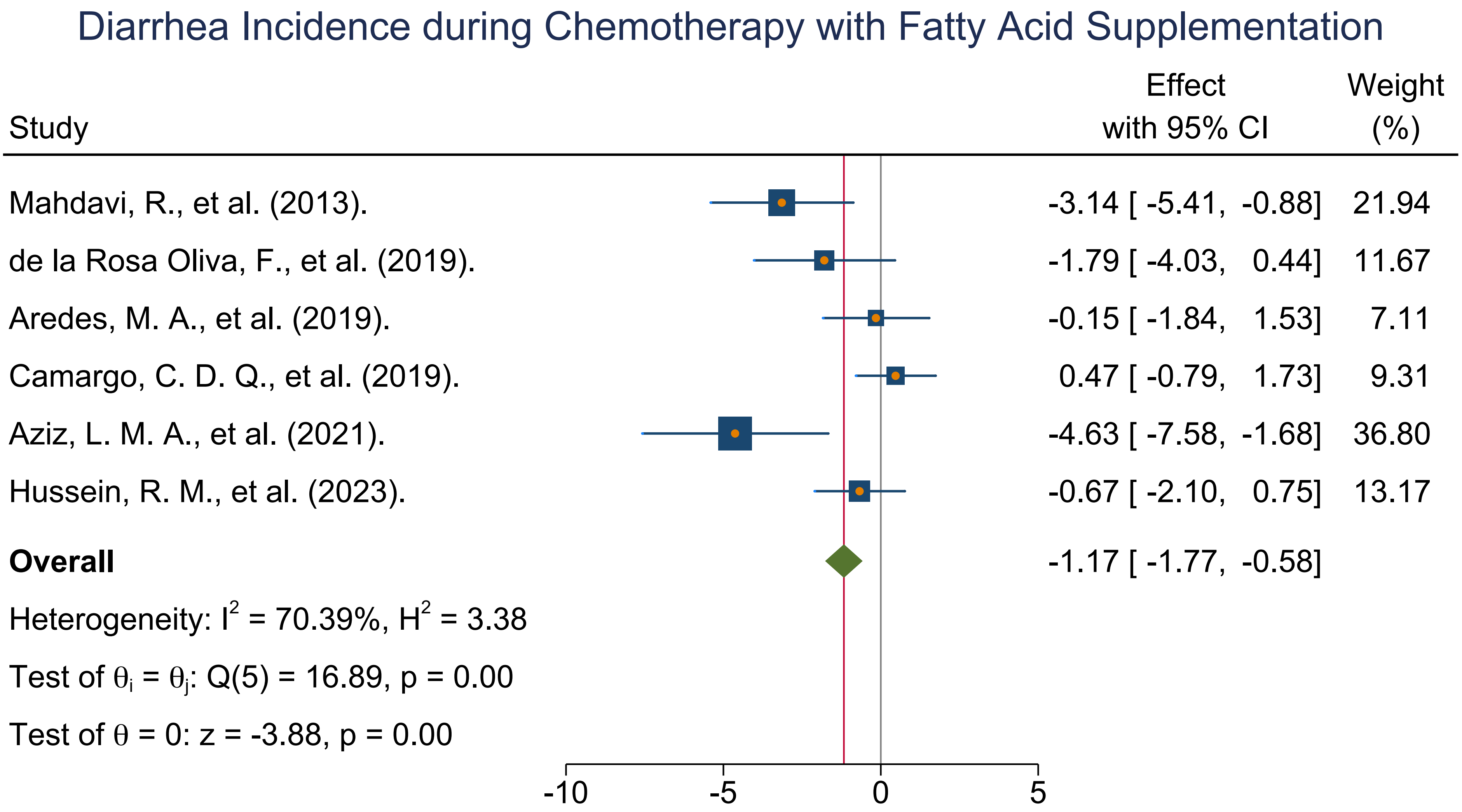
Diarrhea Incidence during Chemotherapy with Fatty Acid Supplementation
Study
Effect with 95% CI
Weight (%)
Mahdavi, R., et al. (2013). -3.14 [-5.41, -0.88] 21.94
de la Rosa Oliva, F., et al. (2019). -1.79 [-4.03, 0.44] 11.67
Aredes, M. A., et al. (2019). -0.15 [-1.84, 1.53] 7.11
Camargo, C. D. Q., et al. (2019). 0.47 [-0.79, 1.73] 9.31
Aziz, L. M. A., et al. (2021). -4.63 [-7.58, -1.68] 36.80
Hussein, R. M., et al. (2023). -0.67 [-2.10, 0.75] 13.17
Overall -1.17 [-1.77, -0.58]
Heterogeneity: I² = 70.39%, H² = 3.38
Test of θi = θj: Q(5) = 16.89, p = 0.00
Test of θ = 0: z = -3.88, p = 0.00
-10
-5
0
5

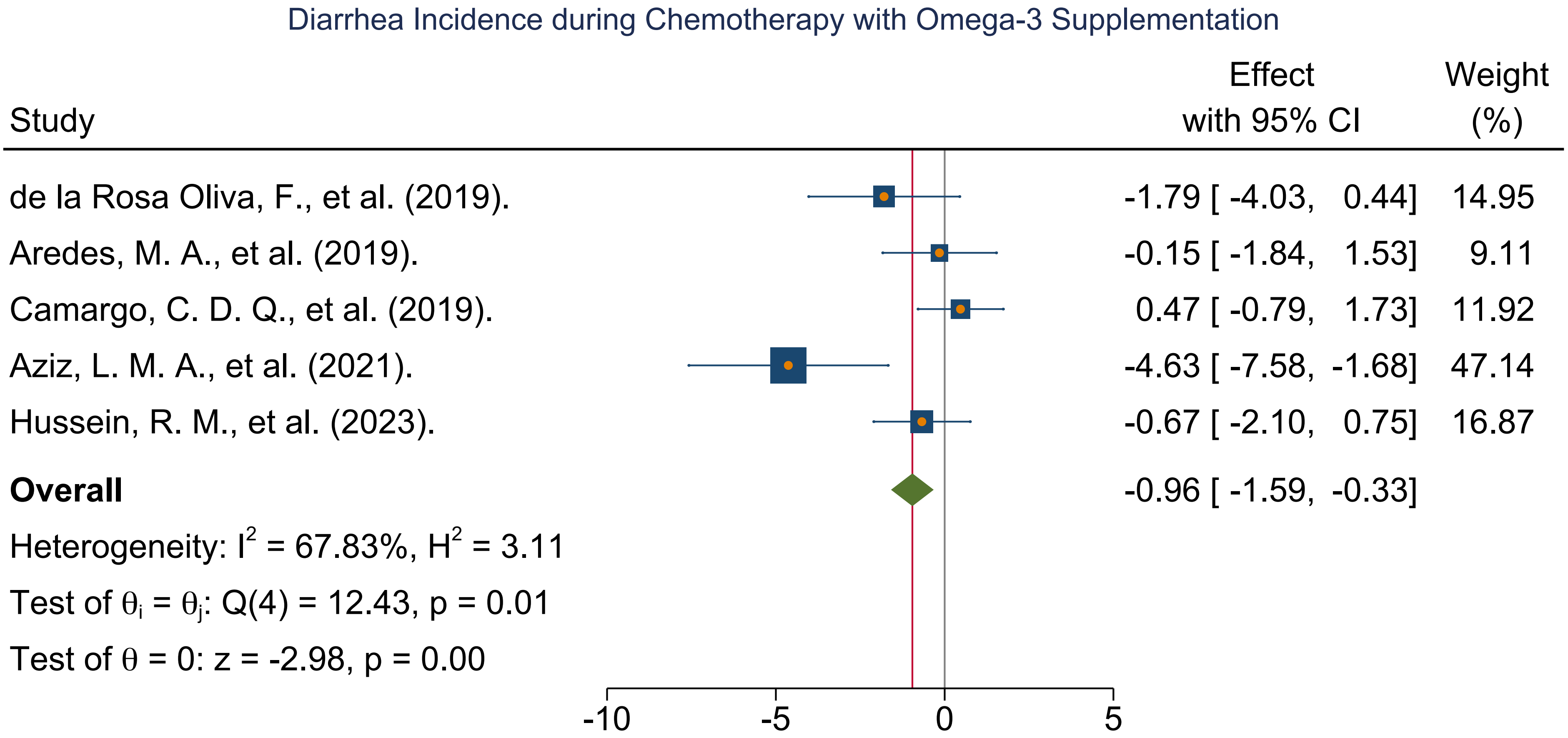
Diarrhea Incidence during Chemotherapy with Omega-3 Supplementation
Study
Effect with 95% CI
Weight (%)
de la Rosa Oliva, F., et al. (2019).
-1.79 [-4.03, 0.44]
14.95
Aredes, M. A., et al. (2019).
-0.15 [-1.84, 1.53]
9.11
Camargo, C. D. Q., et al. (2019).
0.47 [-0.79, 1.73]
11.92
Aziz, L. M. A., et al. (2021).
-4.63 [-7.58, -1.68]
47.14
Hussein, R. M., et al. (2023).
-0.67 [-2.10, 0.75]
16.87
Overall
-0.96 [-1.59, -0.33]
Heterogeneity: I^2 = 67.83%, H^2 = 3.11
Test of $\theta_i = \theta_j$: Q(4) = 12.43, p = 0.01
Test of $\theta = 0$: z = -2.98, p = 0.00
-10
-5
0
5

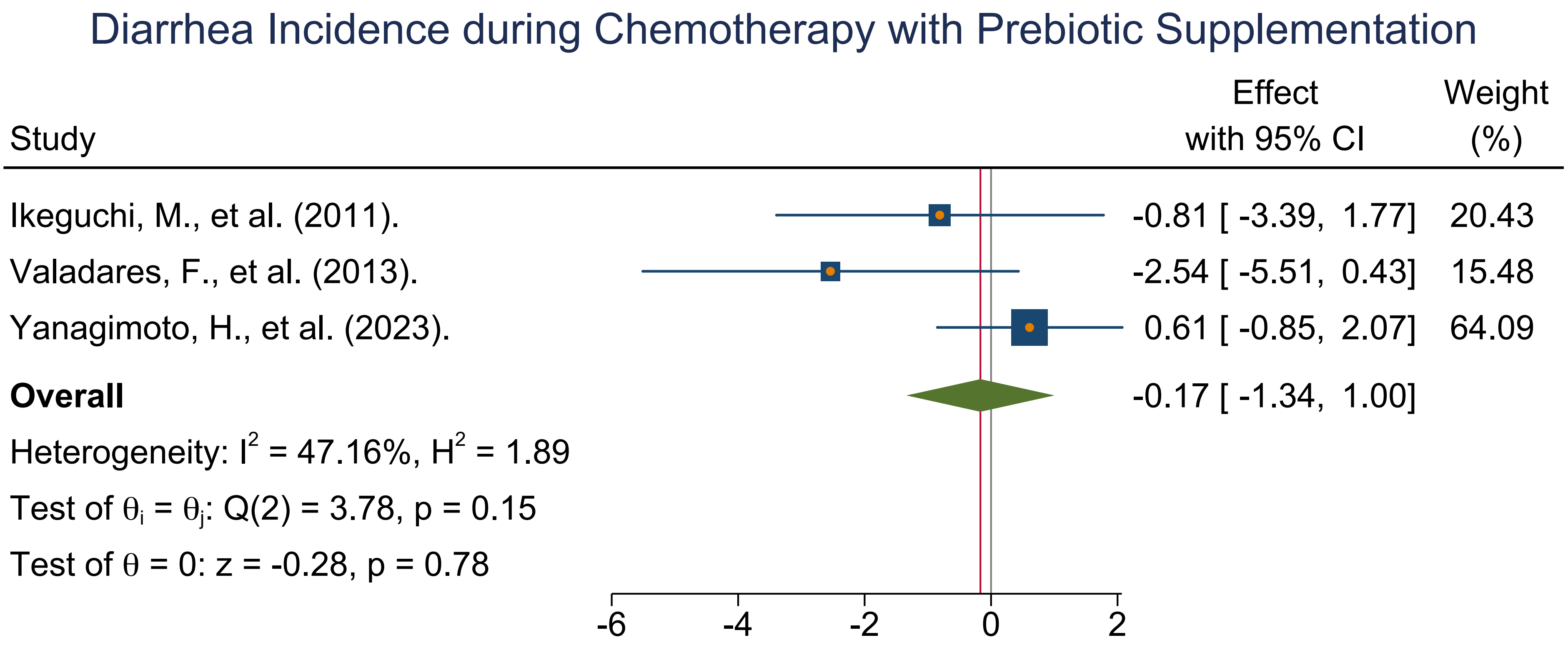
Diarrhea Incidence during Chemotherapy with Prebiotic Supplementation
Study
Effect with 95% CI
Weight (%)
Ikeguchi, M., et al. (2011). -0.81 [-3.39, 1.77] 20.43
Valadares, F., et al. (2013). -2.54 [-5.51, 0.43] 15.48
Yanagimoto, H., et al. (2023). 0.61 [-0.85, 2.07] 64.09
Overall -0.17 [-1.34, 1.00]
Heterogeneity: I^2 = 47.16%, H^2 = 1.89
Test of $\theta_i = \theta_j$: Q(2) = 3.78, p = 0.15
Test of θ = 0: z = -0.28, p = 0.78
-6
-4
-2
0
2

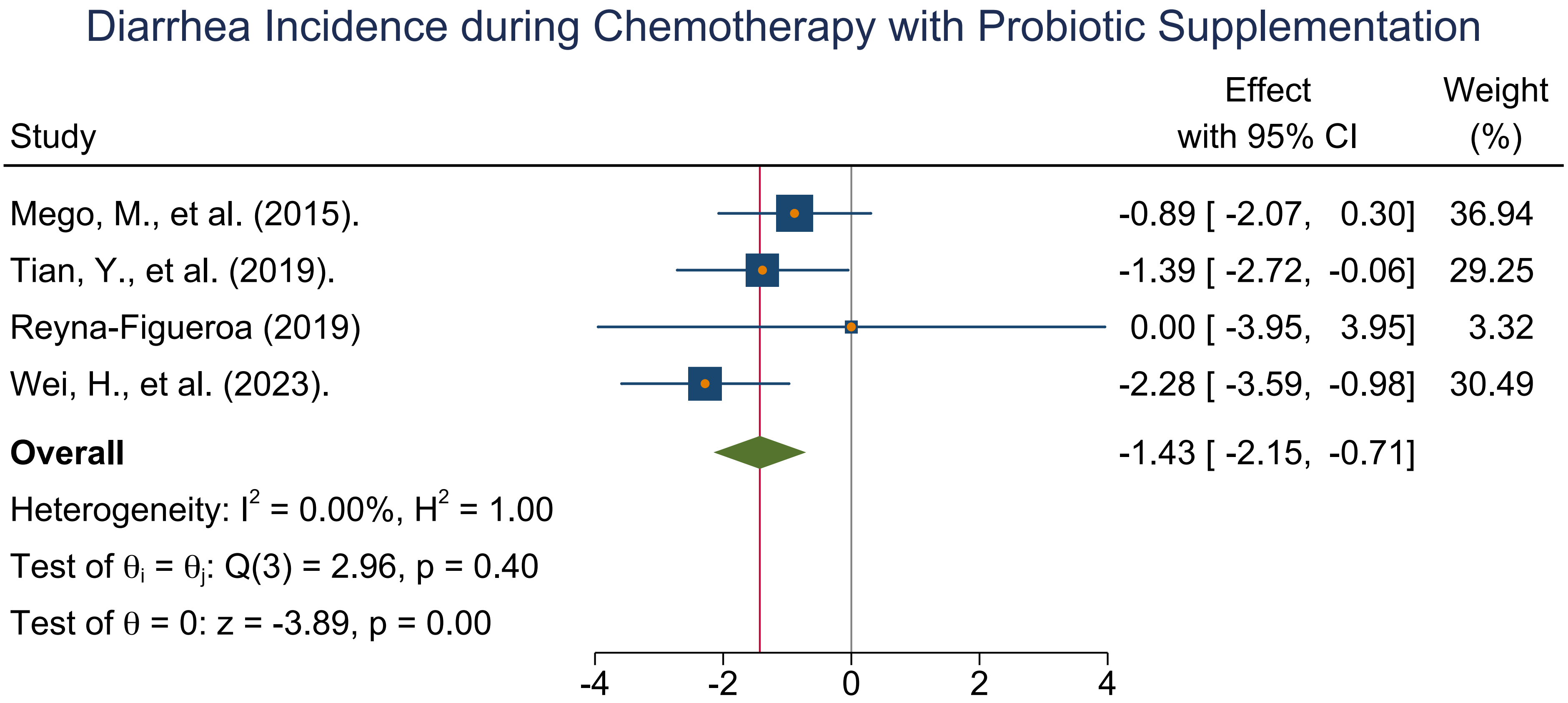
Diarrhea Incidence during Chemotherapy with Probiotic Supplementation
Study
Effect with 95% CI
Weight (%)
Mego, M., et al. (2015). -0.89 [-2.07, 0.30] 36.94
Tian, Y., et al. (2019). -1.39 [-2.72, -0.06] 29.25
Reyna-Figueroa (2019) 0.00 [-3.95, 3.95] 3.32
Wei, H., et al. (2023). -2.28 [-3.59, -0.98] 30.49
Overall -1.43 [-2.15, -0.71]
Heterogeneity: I^2 = 0.00%, H^2 = 1.00
Test of $\theta_i = \theta_j$: Q(3) = 2.96, p = 0.40
Test of $\theta = 0$: z = -3.89, p = 0.00
-4
-2
0
2
4

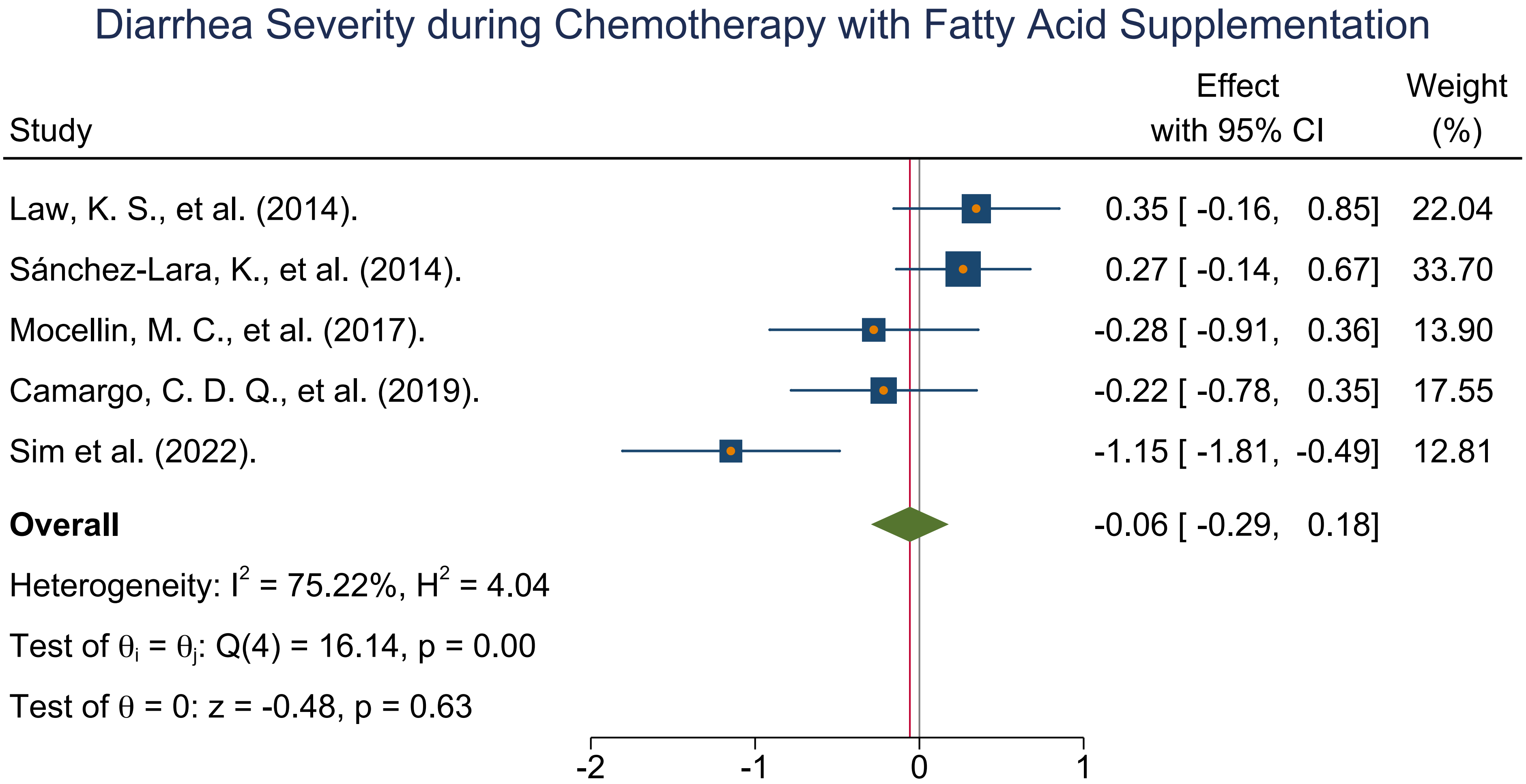
Diarrhea Severity during Chemotherapy with Fatty Acid Supplementation
Study
Effect with 95% CI
Weight (%)
Law, K. S., et al. (2014). 0.35 [-0.16, 0.85] 22.04
Sánchez-Lara, K., et al. (2014). 0.27 [-0.14, 0.67] 33.70
Mocellin, M. C., et al. (2017). -0.28 [-0.91, 0.36] 13.90
Camargo, C. D. Q., et al. (2019). -0.22 [-0.78, 0.35] 17.55
Sim et al. (2022). -1.15 [-1.81, -0.49] 12.81
Overall -0.06 [-0.29, 0.18]
Heterogeneity: I^2 = 75.22%, H^2 = 4.04
Test of $\theta_i = \theta_j$: Q(4) = 16.14, p = 0.00
Test of θ = 0: z = -0.48, p = 0.63
-2
-1
0
1

Diarrhea Severity during Chemotherapy with Probiotic Supplementation

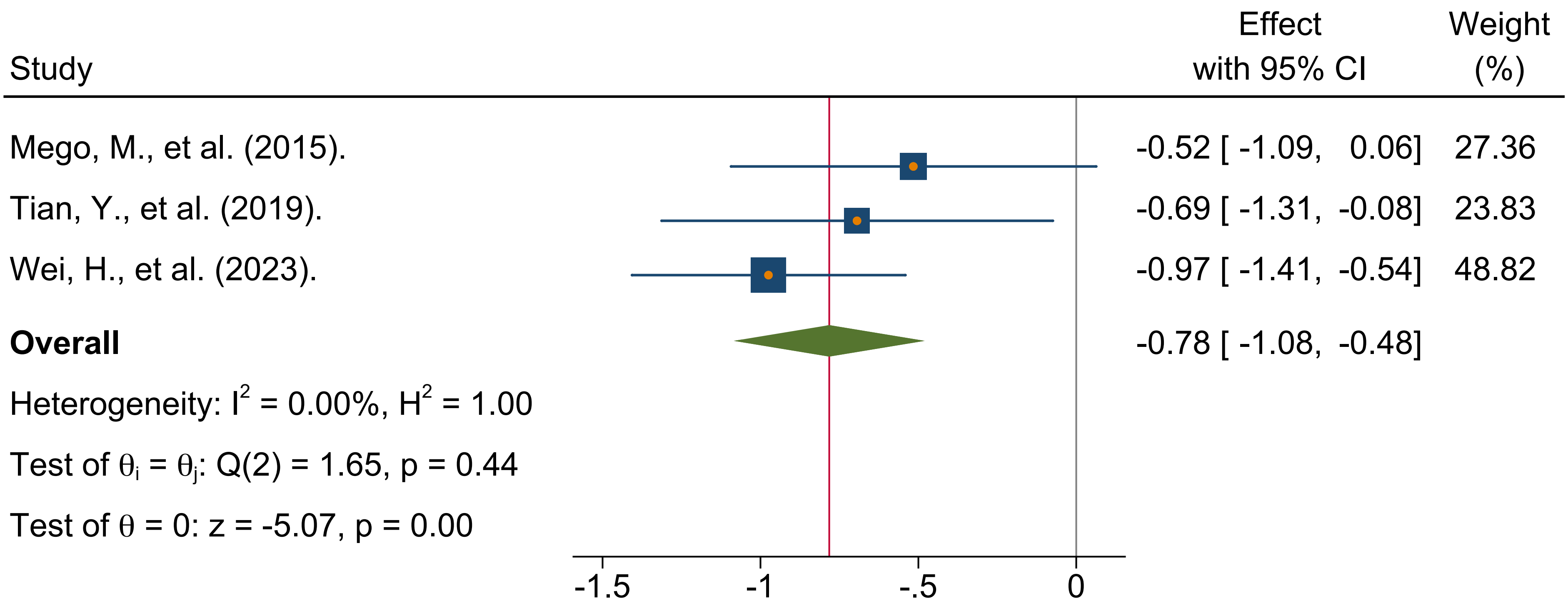

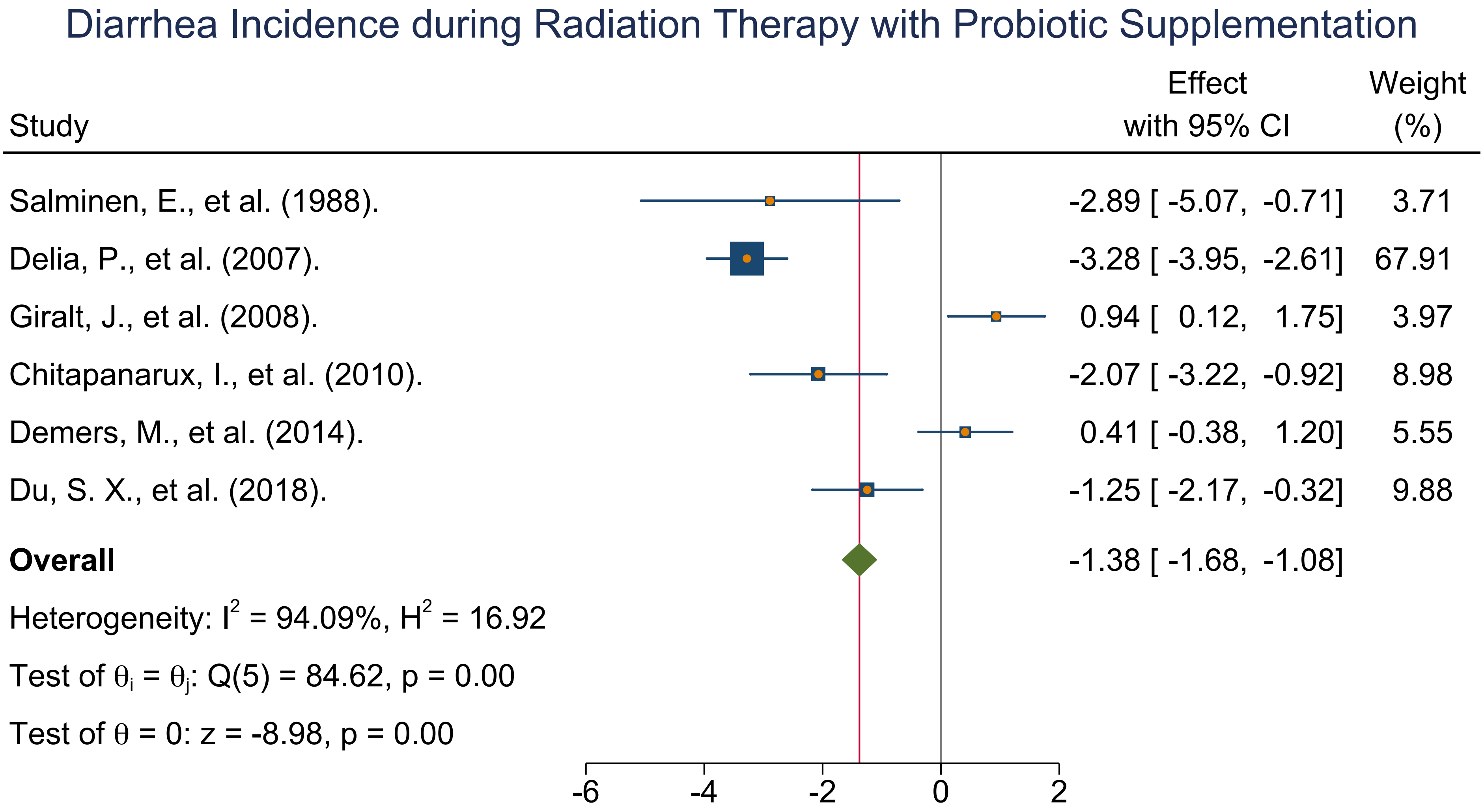
Diarrhea Incidence during Radiation Therapy with Probiotic Supplementation
Study
Effect with 95% CI
Weight (%)
Salminen, E., et al. (1988). -2.89 [-5.07, -0.71] 3.71
Delia, P., et al. (2007). -3.28 [-3.95, -2.61] 67.91
Giralt, J., et al. (2008). 0.94 [0.12, 1.75] 3.97
Chitapanarux, I., et al. (2010). -2.07 [-3.22, -0.92] 8.98
Demers, M., et al. (2014). 0.41 [-0.38, 1.20] 5.55
Du, S. X., et al. (2018). -1.25 [-2.17, -0.32] 9.88
Overall -1.38 [-1.68, -1.08]
Heterogeneity: I^2 = 94.09%, H^2 = 16.92
Test of $\theta_i = \theta_j$: Q(5) = 84.62, p = 0.00
Test of θ = 0: z = -8.98, p = 0.00
-6
-4
-2
0
2

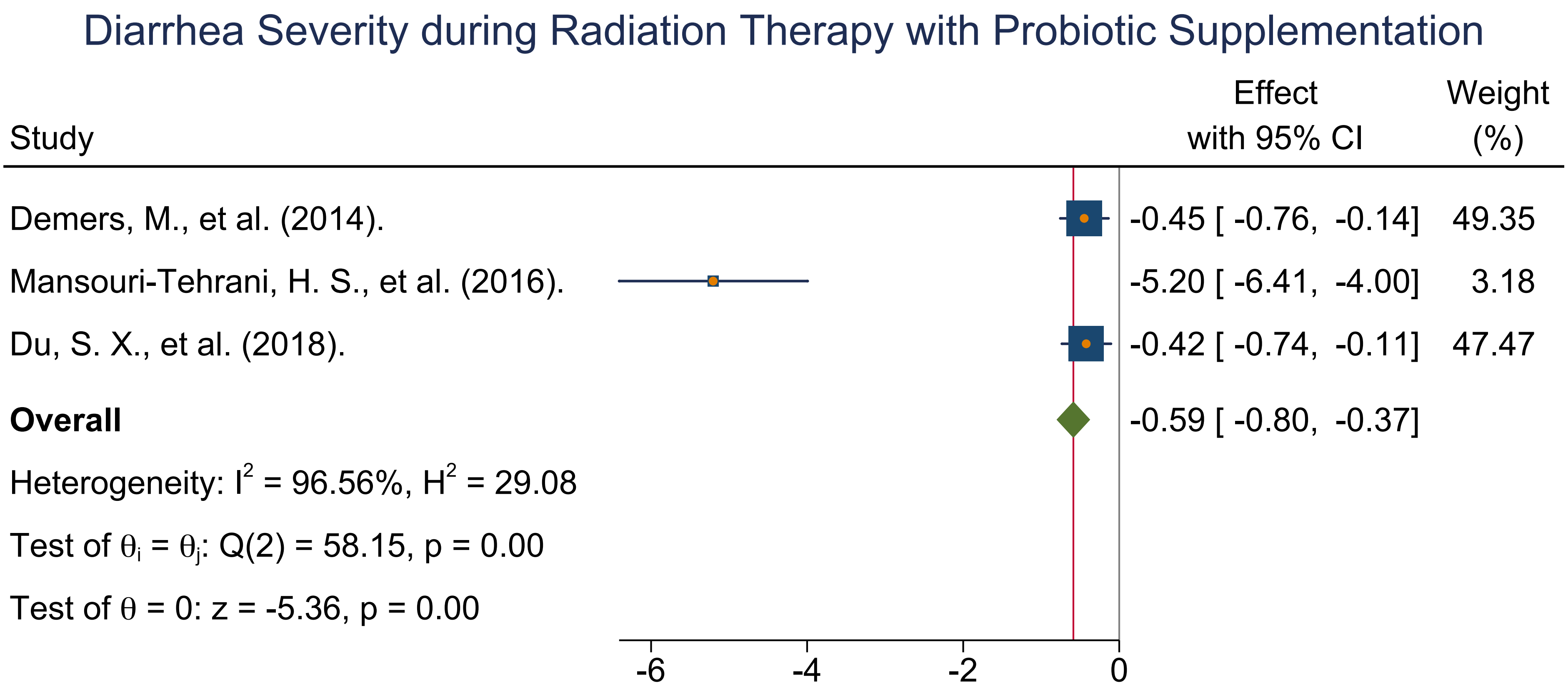
Diarrhea Severity during Radiation Therapy with Probiotic Supplementation
Study
Effect with 95% CI
Weight (%)
Demers, M., et al. (2014). -0.45 [-0.76, -0.14] 49.35
Mansouri-Tehrani, H. S., et al. (2016). -5.20 [-6.41, -4.00] 3.18
Du, S. X., et al. (2018). -0.42 [-0.74, -0.11] 47.47
Overall -0.59 [-0.80, -0.37]
Heterogeneity: I2 = 96.56%, H2 = 29.08
Test of θi = θj: Q(2) = 58.15, p = 0.00
Test of θ = 0: z = -5.36, p = 0.00
-6
-4
-2
0

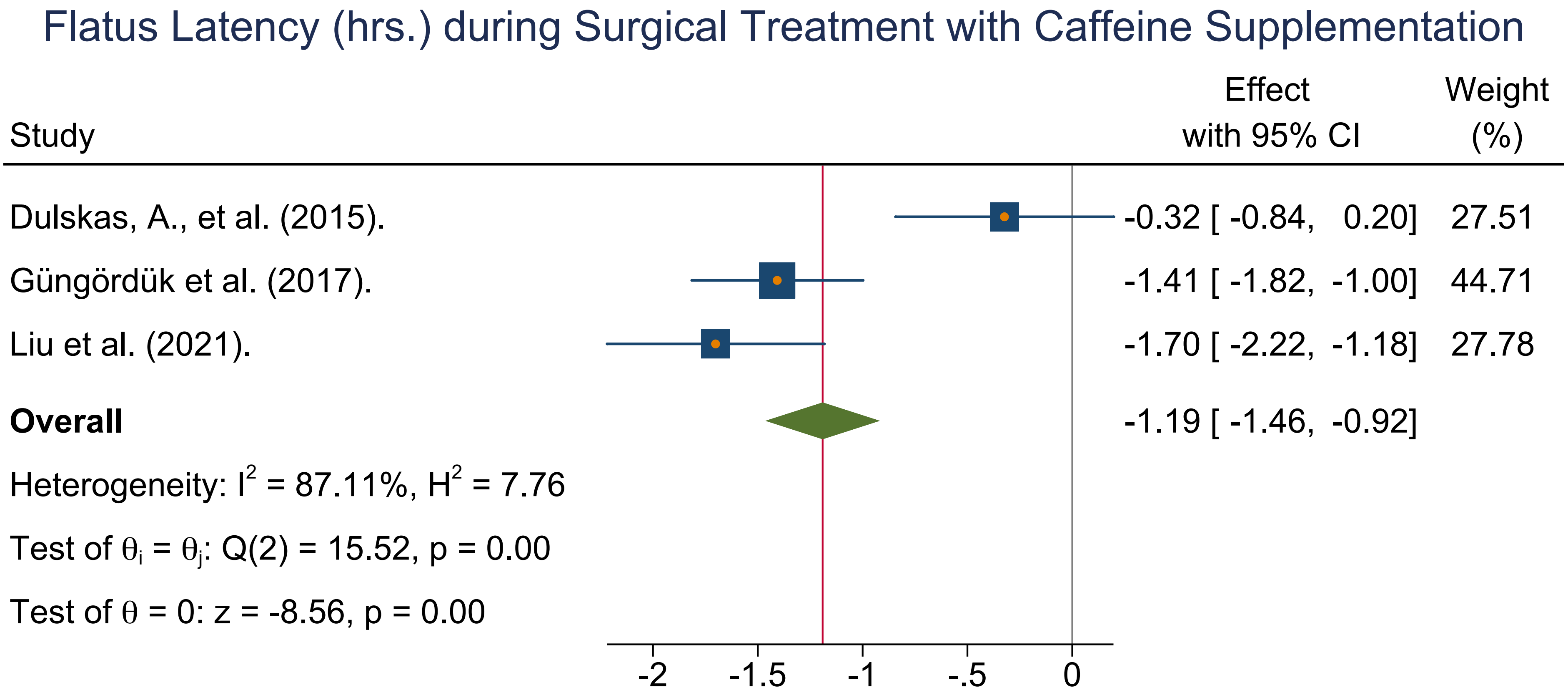

Flatus Latency (hrs.) during Surgical Treatment with Caffeine Supplementation
Study
Effect with 95% CI
Weight (%)
Dulskas, A., et al. (2015). -0.32 [-0.84, 0.20] 27.51
Güngördük et al. (2017). -1.41 [-1.82, -1.00] 44.71
Liu et al. (2021). -1.70 [-2.22, -1.18] 27.78
Overall -1.19 [-1.46, -0.92]
Heterogeneity: I^2 = 87.11%, H^2 = 7.76
Test of $\theta_i = \theta_j$: Q(2) = 15.52, p = 0.00
Test of θ = 0: z = -8.56, p = 0.00
-2
-1.5
-1
-.5
0

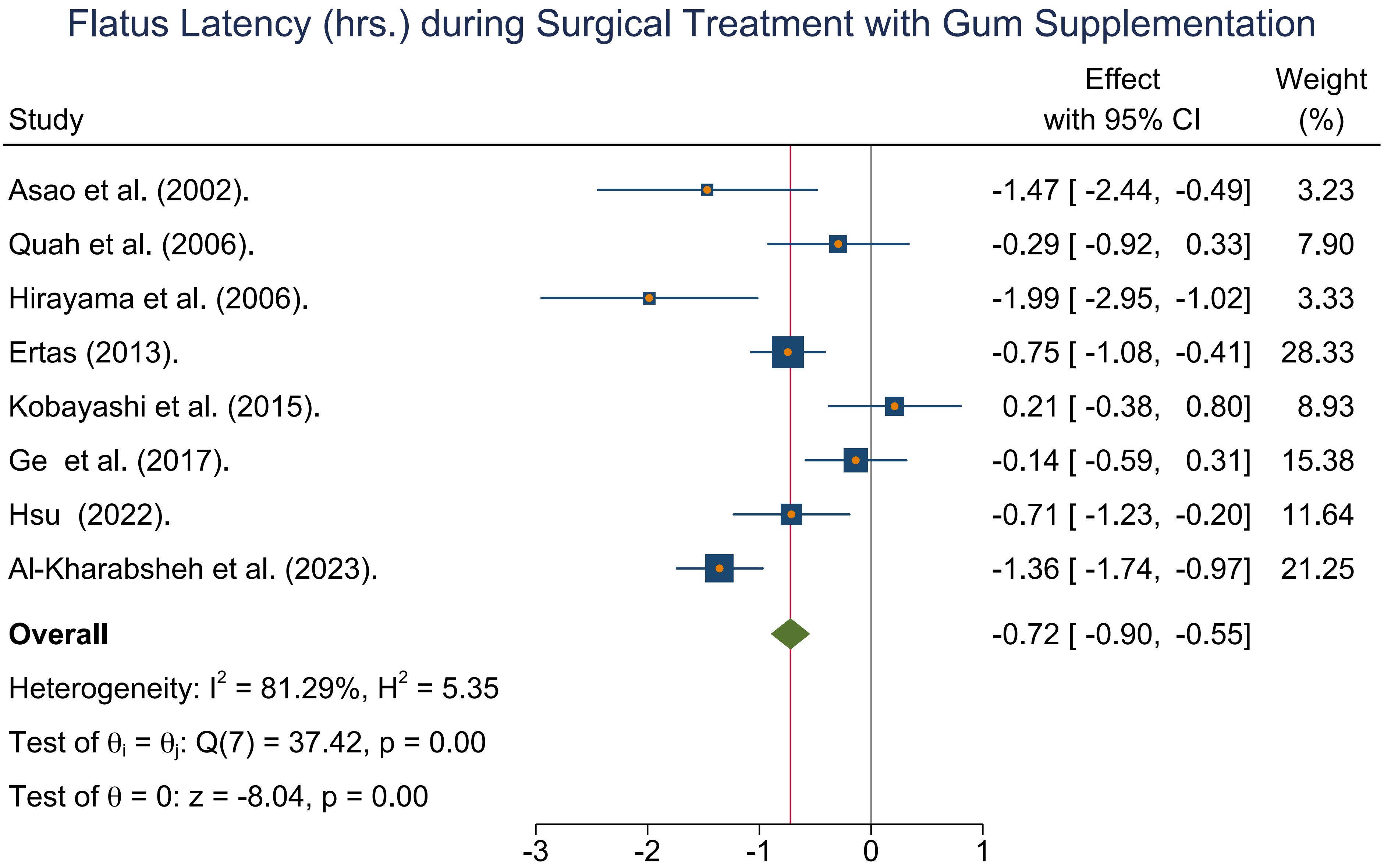
Flatus Latency (hrs.) during Surgical Treatment with Gum Supplementation
Study
Effect with 95% CI
Weight (%)
Asao et al. (2002). -1.47 [-2.44, -0.49] 3.23
Quah et al. (2006). -0.29 [-0.92, 0.33] 7.90
Hirayama et al. (2006). -1.99 [-2.95, -1.02] 3.33
Ertas (2013). -0.75 [-1.08, -0.41] 28.33
Kobayashi et al. (2015). 0.21 [-0.38, 0.80] 8.93
Ge et al. (2017). -0.14 [-0.59, 0.31] 15.38
Hsu (2022). -0.71 [-1.23, -0.20] 11.64
Al-Kharabsheh et al. (2023). -1.36 [-1.74, -0.97] 21.25
Overall -0.72 [-0.90, -0.55]
Heterogeneity: I2 = 81.29%, H2 = 5.35
Test of θi = θj: Q(7) = 37.42, p = 0.00
Test of θ = 0: z = -8.04, p = 0.00
-3
-2
-1
0
1

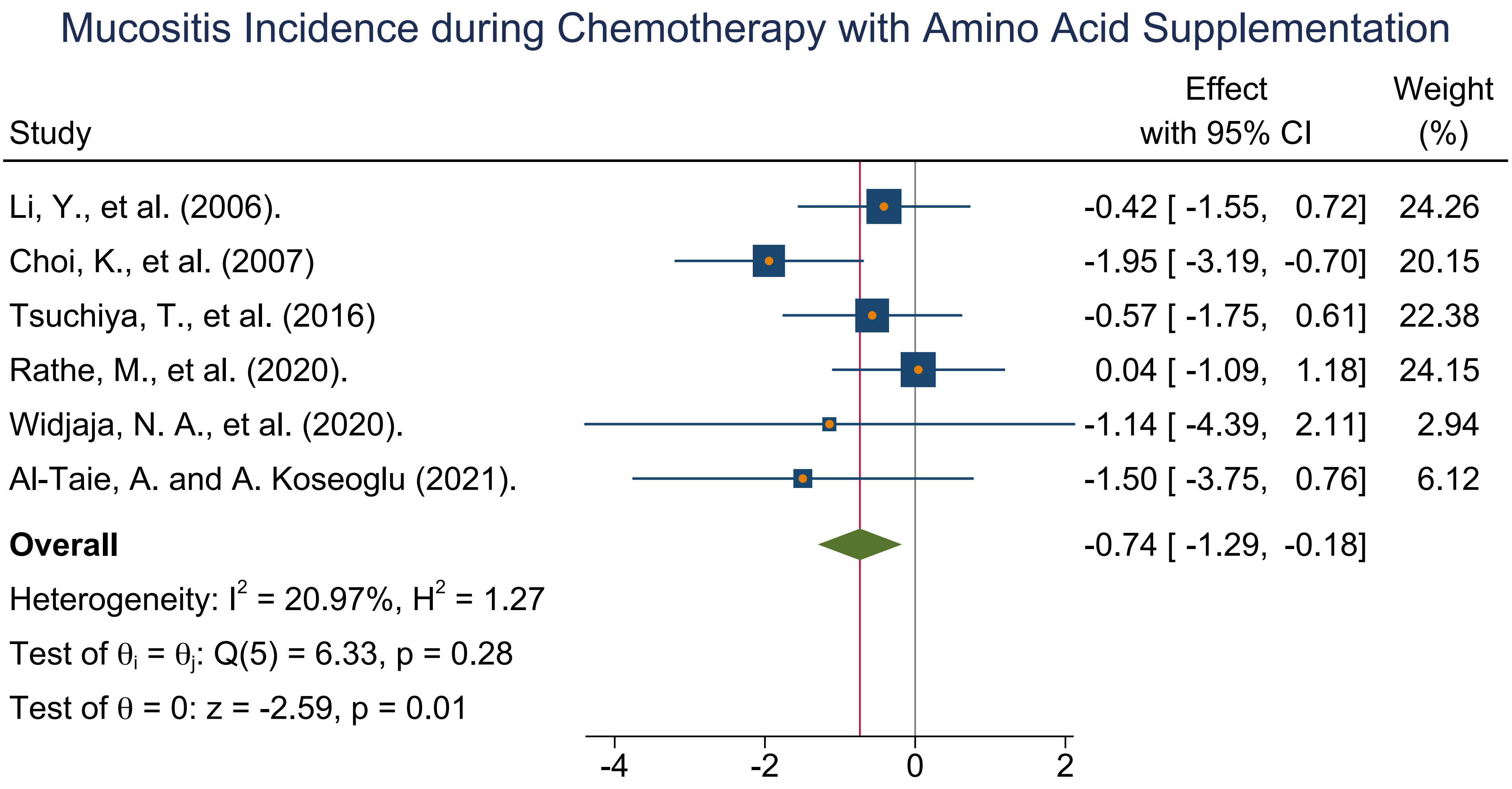
Mucositis Incidence during Chemotherapy with Amino Acid Supplementation
Study
Effect with 95% CI
Weight (%)
Li, Y., et al. (2006). -0.42 [-1.55, 0.72] 24.26
Choi, K., et al. (2007) -1.95 [-3.19, -0.70] 20.15
Tsuchiya, T., et al. (2016) -0.57 [-1.75, 0.61] 22.38
Rathe, M., et al. (2020). 0.04 [-1.09, 1.18] 24.15
Widjaja, N. A., et al. (2020). -1.14 [-4.39, 2.11] 2.94
Al-Taie, A. and A. Koseoglu (2021). -1.50 [-3.75, 0.76] 6.12
Overall -0.74 [-1.29, -0.18]
Heterogeneity: I^2 = 20.97%, H^2 = 1.27
Test of $\theta_i = \theta_j$: Q(5) = 6.33, p = 0.28
Test of θ = 0: z = -2.59, p = 0.01
-4
-2
0
2

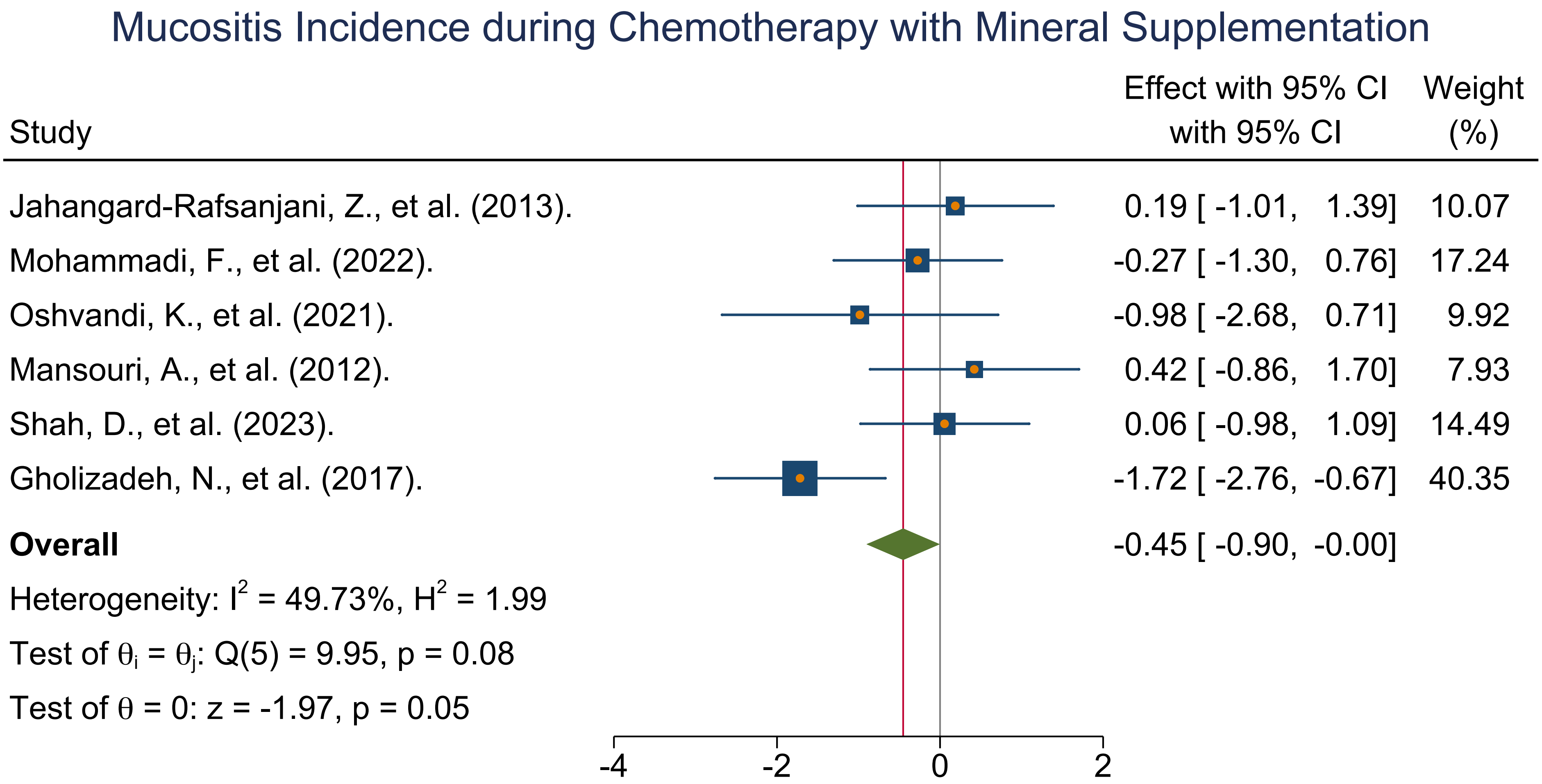
Mucositis Incidence during Chemotherapy with Mineral Supplementation
Study
Effect with 95% CI
with 95% CI
Weight
(%)
Jahangard-Rafsanjani, Z., et al. (2013).
0.19 [-1.01, 1.39] 10.07
Mohammadi, F., et al. (2022).
-0.27 [-1.30, 0.76] 17.24
Oshvandi, K., et al. (2021).
-0.98 [-2.68, 0.71] 9.92
Mansouri, A., et al. (2012).
0.42 [-0.86, 1.70] 7.93
Shah, D., et al. (2023).
0.06 [-0.98, 1.09] 14.49
Gholizadeh, N., et al. (2017).
-1.72 [-2.76, -0.67] 40.35
Overall
-0.45 [-0.90, -0.00]
Heterogeneity: I^2 = 49.73%, H^2 = 1.99
Test of $\theta_i = \theta_j$: Q(5) = 9.95, p = 0.08
Test of θ = 0: z = -1.97, p = 0.05
-4
-2
0
2

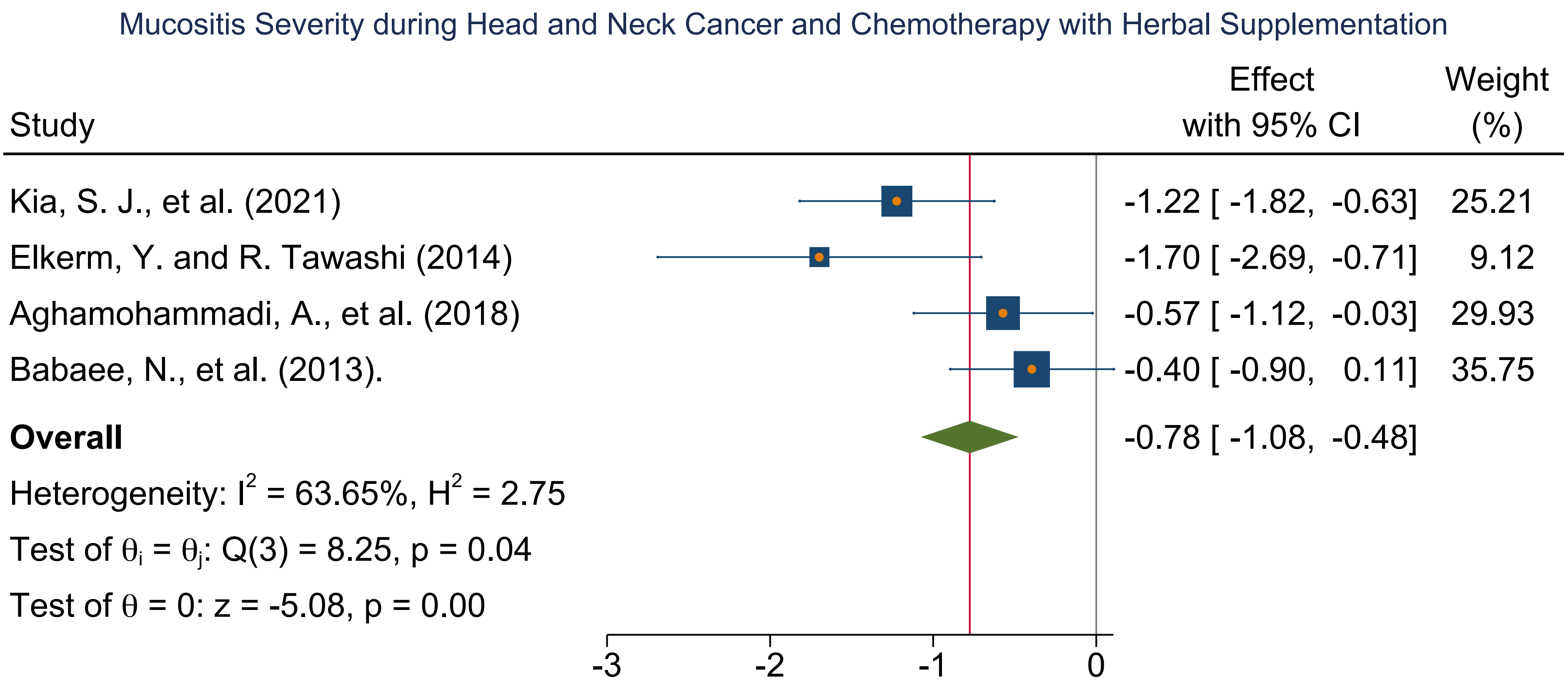
Mucositis Severity during Head and Neck Cancer and Chemotherapy with Herbal Supplementation
Study
Effect with 95% CI
Weight (%)
Kia, S. J., et al. (2021) -1.22 [-1.82, -0.63] 25.21
Elkerm, Y. and R. Tawashi (2014) -1.70 [-2.69, -0.71] 9.12
Aghamohammadi, A., et al. (2018) -0.57 [-1.12, -0.03] 29.93
Babaee, N., et al. (2013). -0.40 [-0.90, 0.11] 35.75
Overall -0.78 [-1.08, -0.48]
Heterogeneity: I^2 = 63.65%, H^2 = 2.75
Test of $\theta_i = \theta_j$: Q(3) = 8.25, p = 0.04
Test of θ = 0: z = -5.08, p = 0.00
-3
-2
-1
0

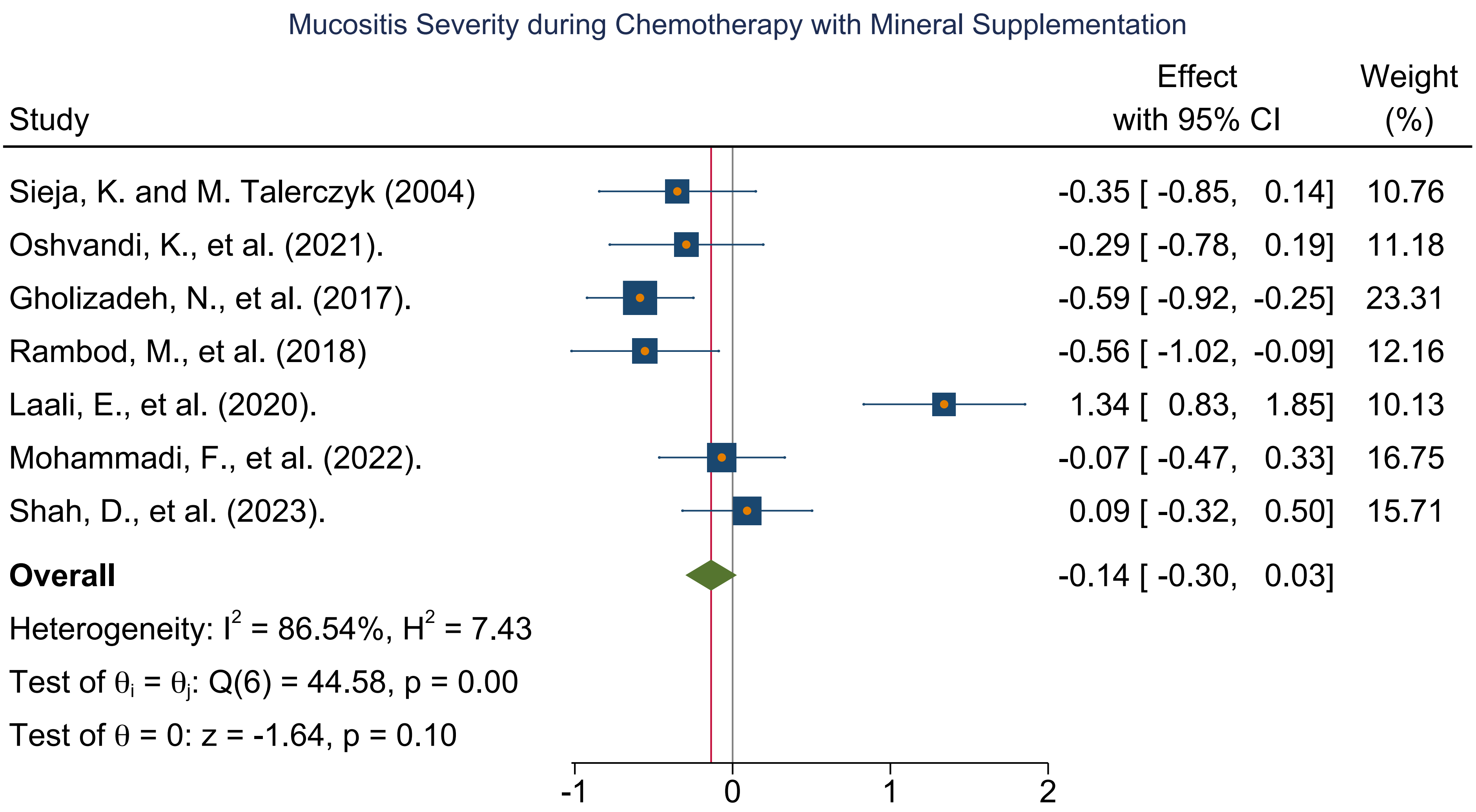

Mucositis Severity during Chemotherapy with Mineral Supplementation
Study
Effect with 95% CI
Weight (%)
Sieja, K. and M. Talerczyk (2004) -0.35 [-0.85, 0.14] 10.76
Oshvandi, K., et al. (2021). -0.29 [-0.78, 0.19] 11.18
Gholizadeh, N., et al. (2017). -0.59 [-0.92, -0.25] 23.31
Rambod, M., et al. (2018) -0.56 [-1.02, -0.09] 12.16
Laali, E., et al. (2020). 1.34 [0.83, 1.85] 10.13
Mohammadi, F., et al. (2022). -0.07 [-0.47, 0.33] 16.75
Shah, D., et al. (2023). 0.09 [-0.32, 0.50] 15.71
Overall -0.14 [-0.30, 0.03]
Heterogeneity: I^2 = 86.54%, H^2 = 7.43
Test of $\theta_i = \theta_j$: Q(6) = 44.58, p = 0.00
Test of θ = 0: z = -1.64, p = 0.10
-1
0
1
2

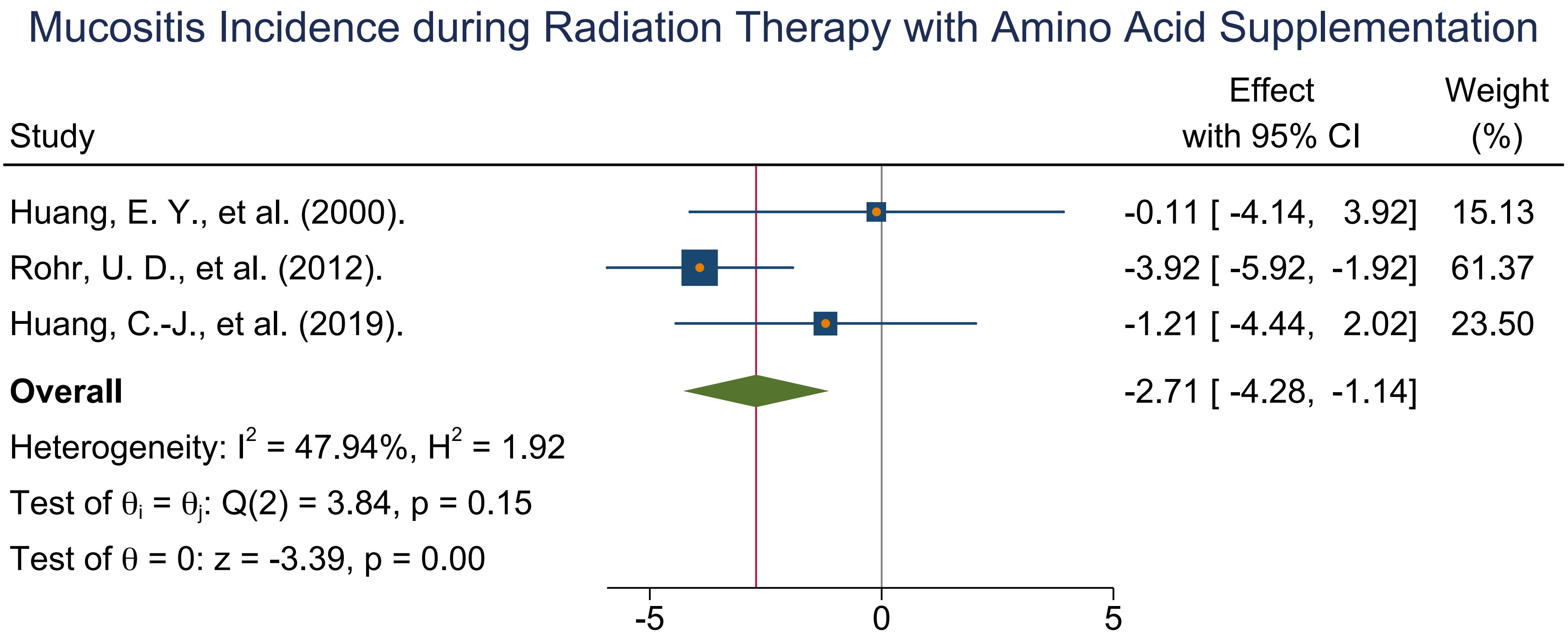
Mucositis Incidence during Radiation Therapy with Amino Acid Supplementation
Study
Effect with 95% CI
Weight (%)
Huang, E. Y., et al. (2000).
-0.11 [-4.14, 3.92]
15.13
Rohr, U. D., et al. (2012).
-3.92 [-5.92, -1.92]
61.37
Huang, C.-J., et al. (2019).
-1.21 [-4.44, 2.02]
23.50
Overall
-2.71 [-4.28, -1.14]
Heterogeneity: I2 = 47.94%, H2 = 1.92
Test of θi = θj: Q(2) = 3.84, p = 0.15
Test of θ = 0: z = -3.39, p = 0.00
-5
0
5

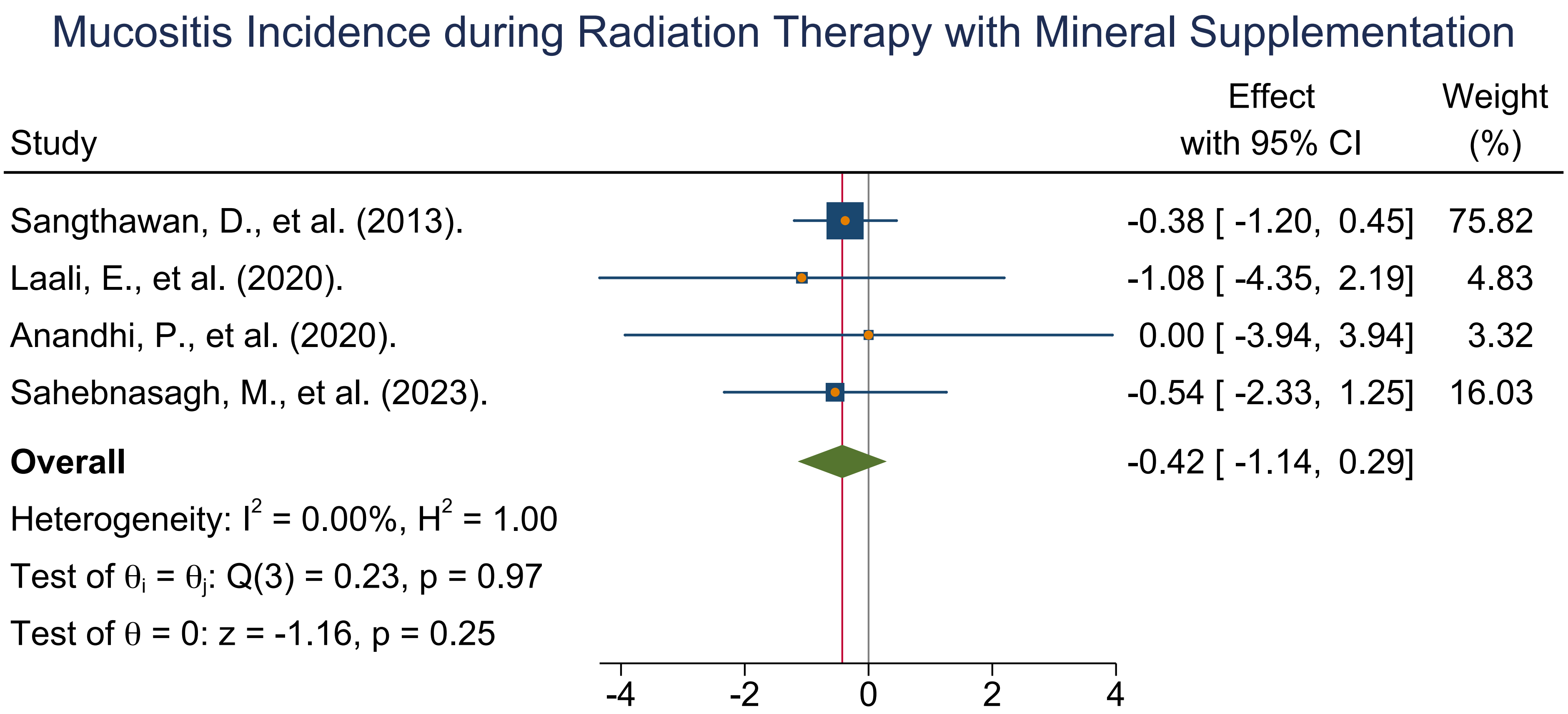
Mucositis Incidence during Radiation Therapy with Mineral Supplementation
Study
Effect with 95% CI
Weight (%)
Sangthawan, D., et al. (2013). -0.38 [-1.20, 0.45] 75.82
Laali, E., et al. (2020). -1.08 [-4.35, 2.19] 4.83
Anandhi, P., et al. (2020). 0.00 [-3.94, 3.94] 3.32
Sahebnasagh, M., et al. (2023). -0.54 [-2.33, 1.25] 16.03
Overall -0.42 [-1.14, 0.29]
Heterogeneity: I² = 0.00%, H² = 1.00
Test of θi = θj: Q(3) = 0.23, p = 0.97
Test of θ = 0: z = -1.16, p = 0.25
-4
-2
0
2
4

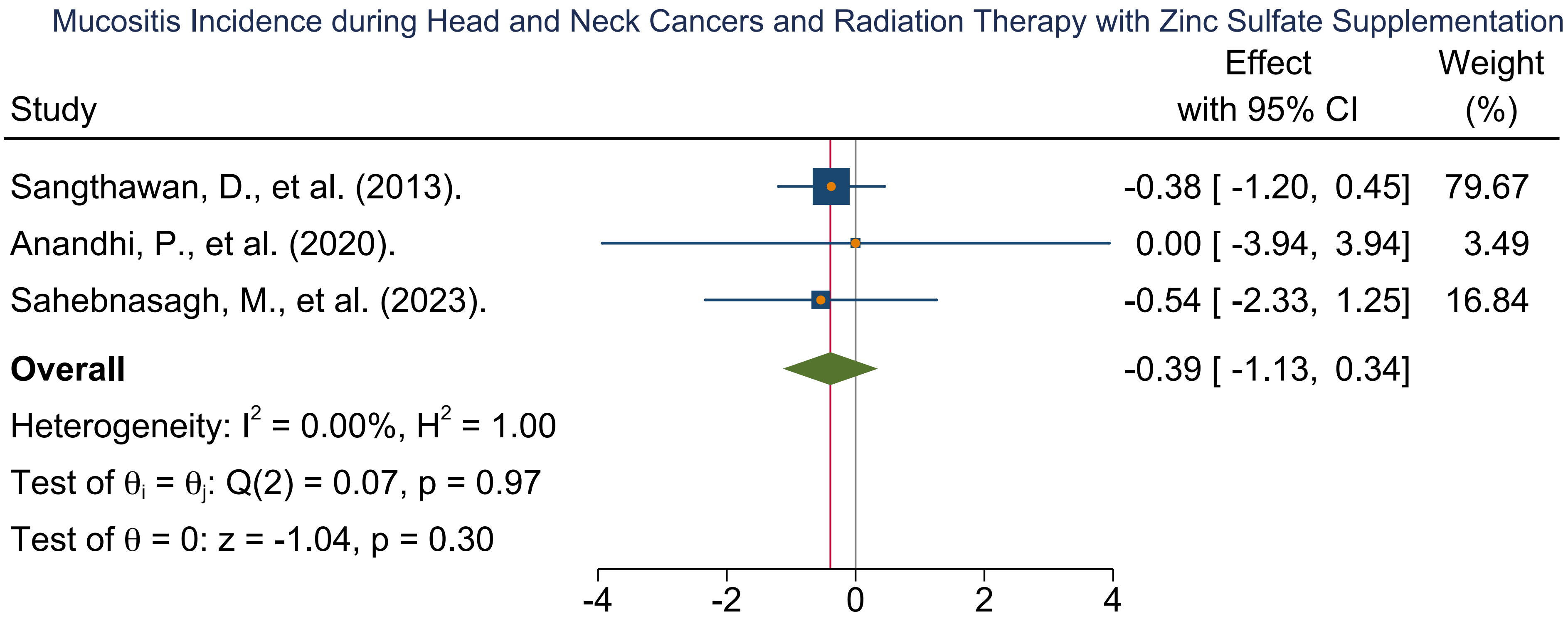
Mucositis Incidence during Head and Neck Cancers and Radiation Therapy with Zinc Sulfate Supplementation
Study
Effect with 95% CI
Weight (%)
Sangthawan, D., et al. (2013). -0.38 [-1.20, 0.45] 79.67
Anandhi, P., et al. (2020). 0.00 [-3.94, 3.94] 3.49
Sahebnasagh, M., et al. (2023). -0.54 [-2.33, 1.25] 16.84
Overall -0.39 [-1.13, 0.34]
Heterogeneity: I^2 = 0.00%, H^2 = 1.00
Test of $\theta_i = \theta_j$: Q(2) = 0.07, p = 0.97
Test of θ = 0: z = -1.04, p = 0.30
-4
-2
0
2
4

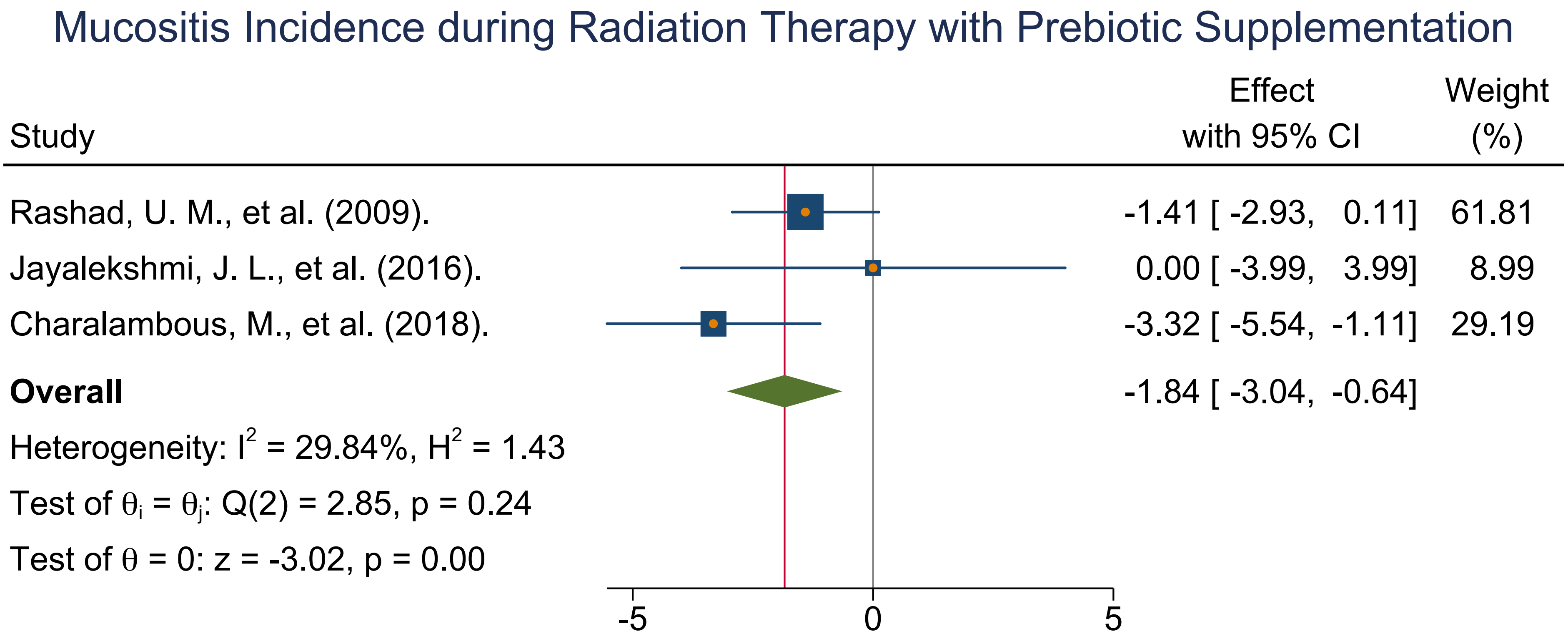
Mucositis Incidence during Radiation Therapy with Prebiotic Supplementation
Study
Effect with 95% CI
Weight (%)
Rashad, U. M., et al. (2009).
-1.41 [-2.93, 0.11]
61.81
Jayalekshmi, J. L., et al. (2016).
0.00 [-3.99, 3.99]
8.99
Charalambous, M., et al. (2018).
-3.32 [-5.54, -1.11]
29.19
Overall
-1.84 [-3.04, -0.64]
Heterogeneity: I^2 = 29.84%, H^2 = 1.43
Test of $\theta_i = \theta_j$: Q(2) = 2.85, p = 0.24
Test of θ = 0: z = -3.02, p = 0.00
-5
0
5

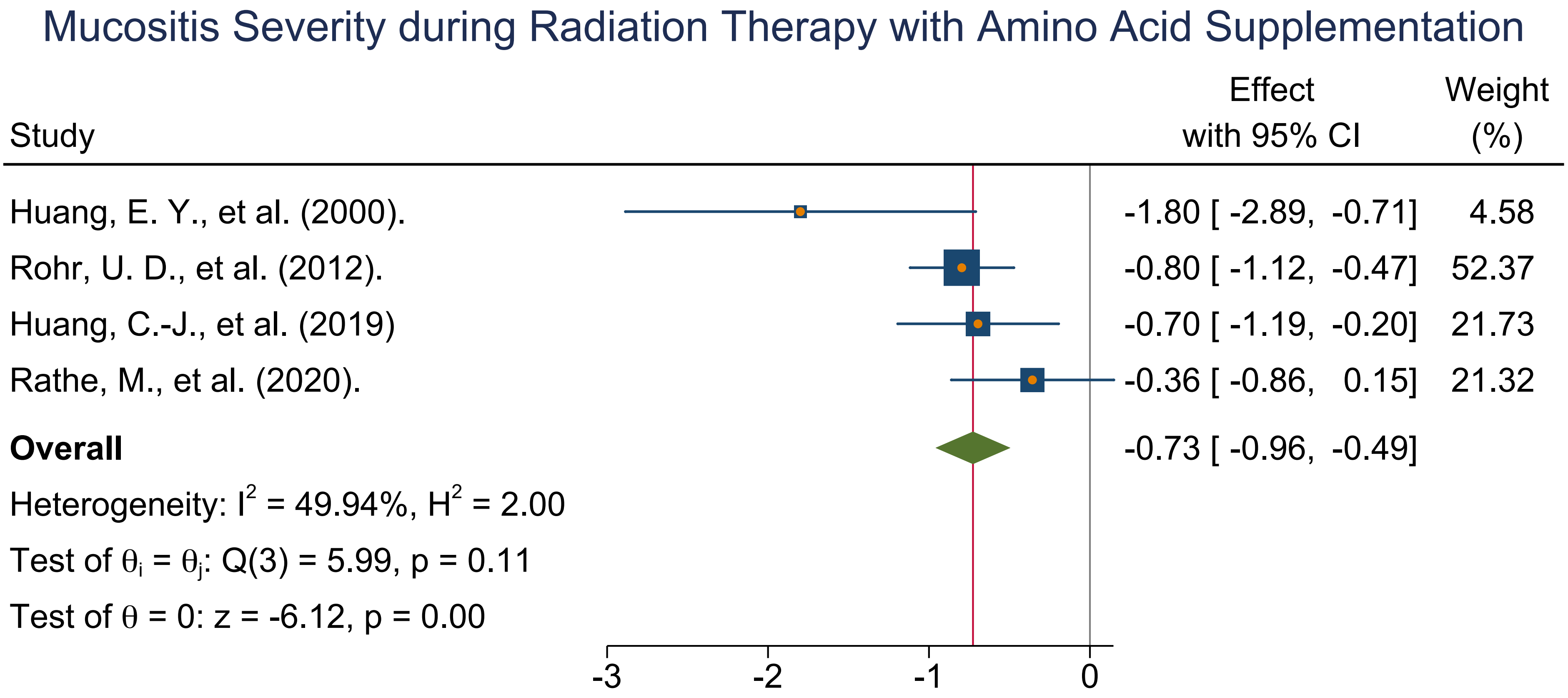

Mucositis Severity during Radiation Therapy with Amino Acid Supplementation
Study
Effect with 95% CI
Weight (%)
Huang, E. Y., et al. (2000). -1.80 [-2.89, -0.71] 4.58
Rohr, U. D., et al. (2012). -0.80 [-1.12, -0.47] 52.37
Huang, C.-J., et al. (2019) -0.70 [-1.19, -0.20] 21.73
Rathe, M., et al. (2020). -0.36 [-0.86, 0.15] 21.32
Overall -0.73 [-0.96, -0.49]
Heterogeneity: I2 = 49.94%, H2 = 2.00
Test of θi = θj: Q(3) = 5.99, p = 0.11
Test of θ = 0: z = -6.12, p = 0.00
-3
-2
-1
0

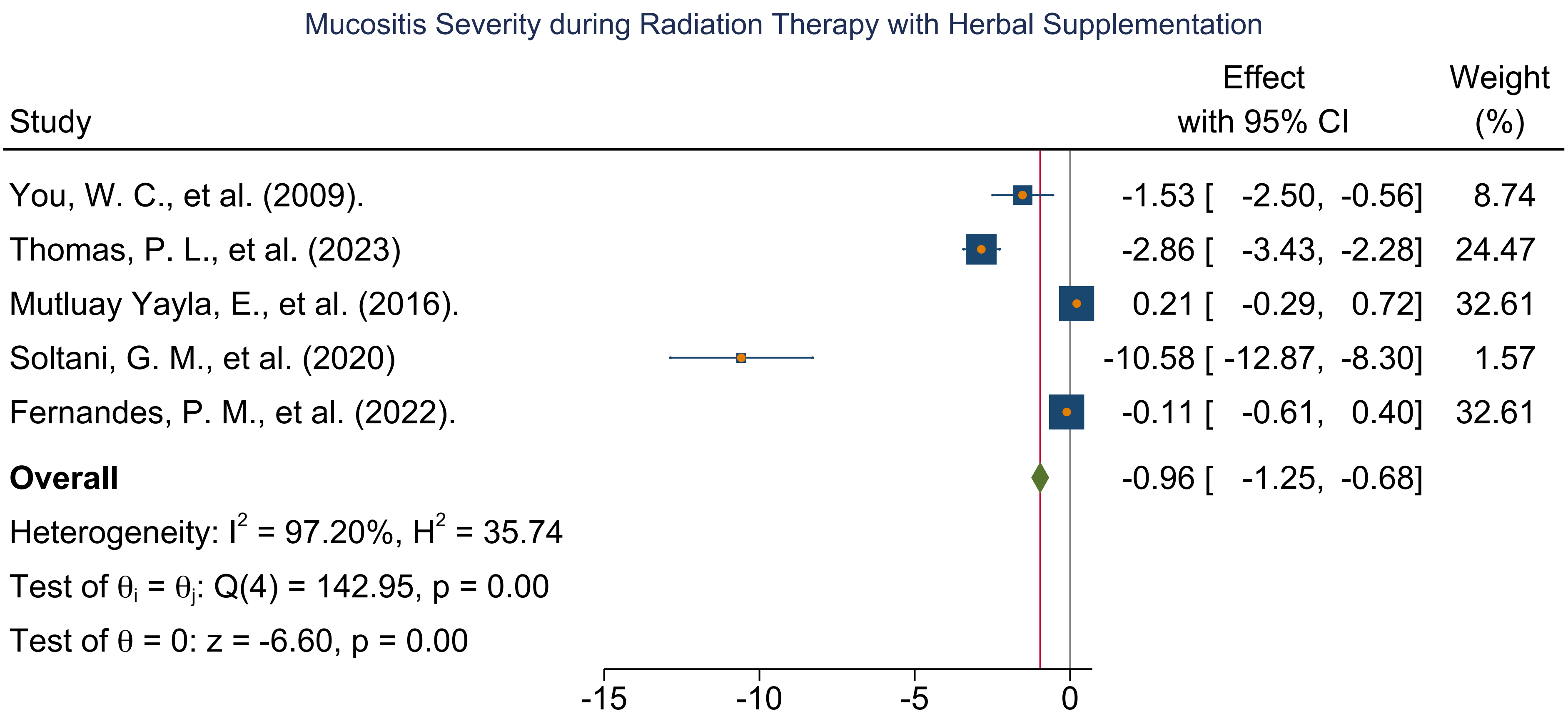
Mucositis Severity during Radiation Therapy with Herbal Supplementation
Study
Effect with 95% CI
Weight (%)
You, W. C., et al. (2009). -1.53 [-2.50, -0.56] 8.74
Thomas, P. L., et al. (2023) -2.86 [-3.43, -2.28] 24.47
Mutluay Yayla, E., et al. (2016). 0.21 [-0.29, 0.72] 32.61
Soltani, G. M., et al. (2020) -10.58 [-12.87, -8.30] 1.57
Fernandes, P. M., et al. (2022). -0.11 [-0.61, 0.40] 32.61
Overall -0.96 [-1.25, -0.68]
Heterogeneity: I^2 = 97.20%, H^2 = 35.74
Test of $\theta_i = \theta_j$: Q(4) = 142.95, p = 0.00
Test of θ = 0: z = -6.60, p = 0.00
-15
-10
-5
0

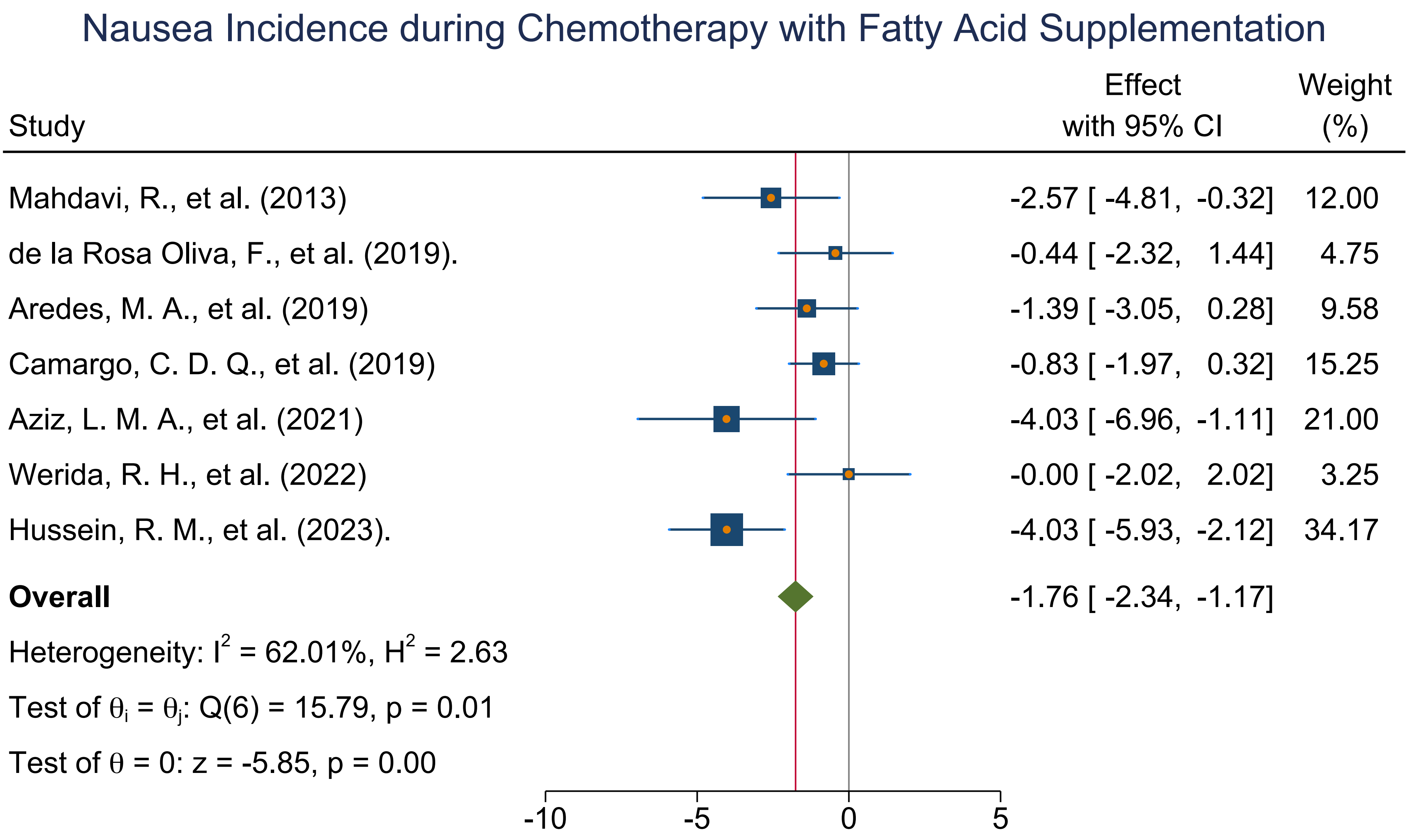
Nausea Incidence during Chemotherapy with Fatty Acid Supplementation
Study
Effect with 95% CI
Weight (%)
Mahdavi, R., et al. (2013) -2.57 [-4.81, -0.32] 12.00
de la Rosa Oliva, F., et al. (2019). -0.44 [-2.32, 1.44] 4.75
Aredes, M. A., et al. (2019) -1.39 [-3.05, 0.28] 9.58
Camargo, C. D. Q., et al. (2019) -0.83 [-1.97, 0.32] 15.25
Aziz, L. M. A., et al. (2021) -4.03 [-6.96, -1.11] 21.00
Werida, R. H., et al. (2022) -0.00 [-2.02, 2.02] 3.25
Hussein, R. M., et al. (2023). -4.03 [-5.93, -2.12] 34.17
Overall -1.76 [-2.34, -1.17]
Heterogeneity: I^2 = 62.01%, H^2 = 2.63
Test of $\theta_i = \theta_j$: Q(6) = 15.79, p = 0.01
Test of θ = 0: z = -5.85, p = 0.00
-10
-5
0
5

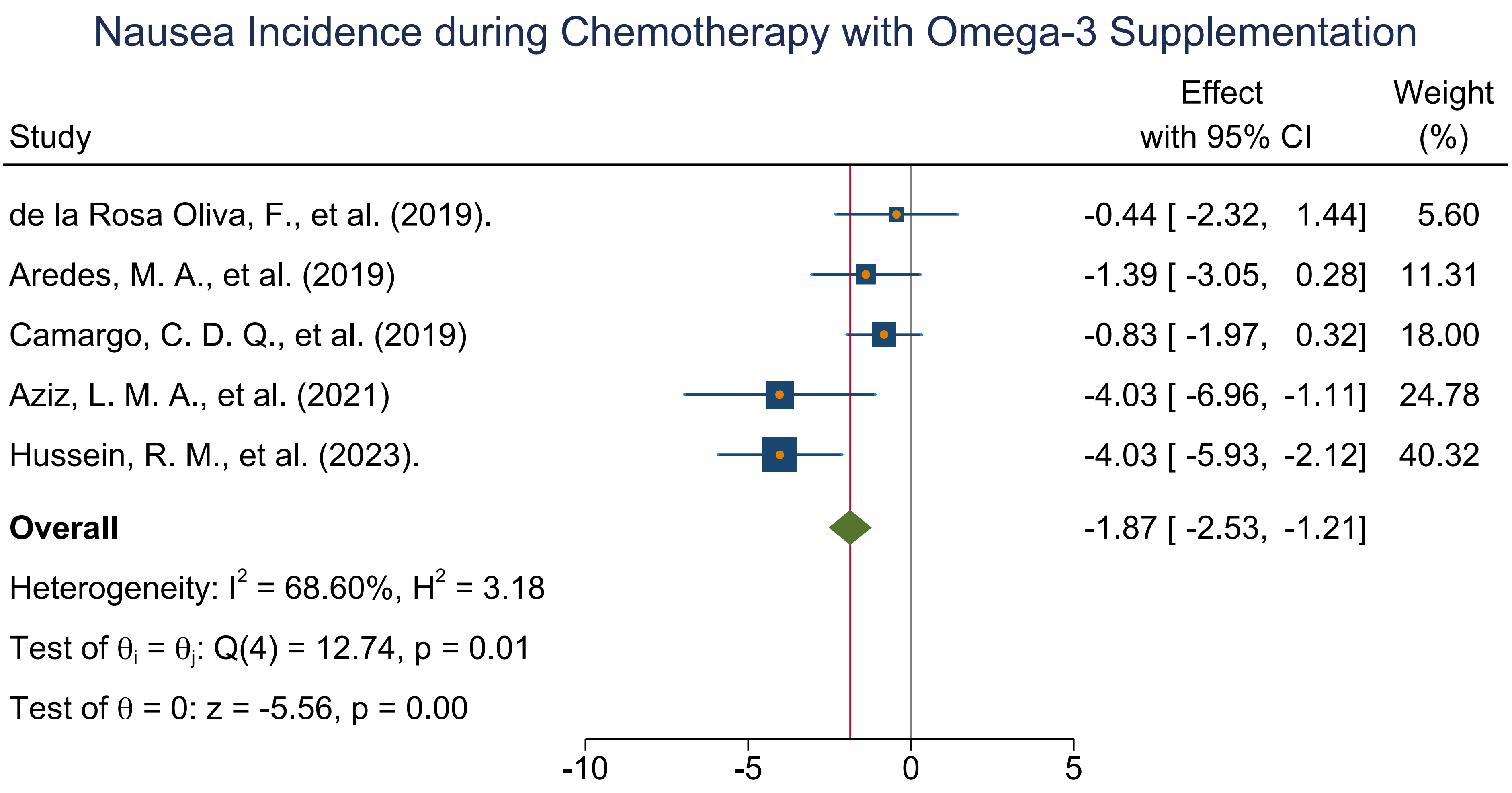
Nausea Incidence during Chemotherapy with Omega-3 Supplementation
Study
Effect with 95% CI
Weight (%)
de la Rosa Oliva, F., et al. (2019). -0.44 [-2.32, 1.44] 5.60
Aredes, M. A., et al. (2019) -1.39 [-3.05, 0.28] 11.31
Camargo, C. D. Q., et al. (2019) -0.83 [-1.97, 0.32] 18.00
Aziz, L. M. A., et al. (2021) -4.03 [-6.96, -1.11] 24.78
Hussein, R. M., et al. (2023). -4.03 [-5.93, -2.12] 40.32
Overall -1.87 [-2.53, -1.21]
Heterogeneity: I^2 = 68.60%, H^2 = 3.18
Test of $\theta_i = \theta_j$: Q(4) = 12.74, p = 0.01
Test of θ = 0: z = -5.56, p = 0.00
-10
-5
0
5

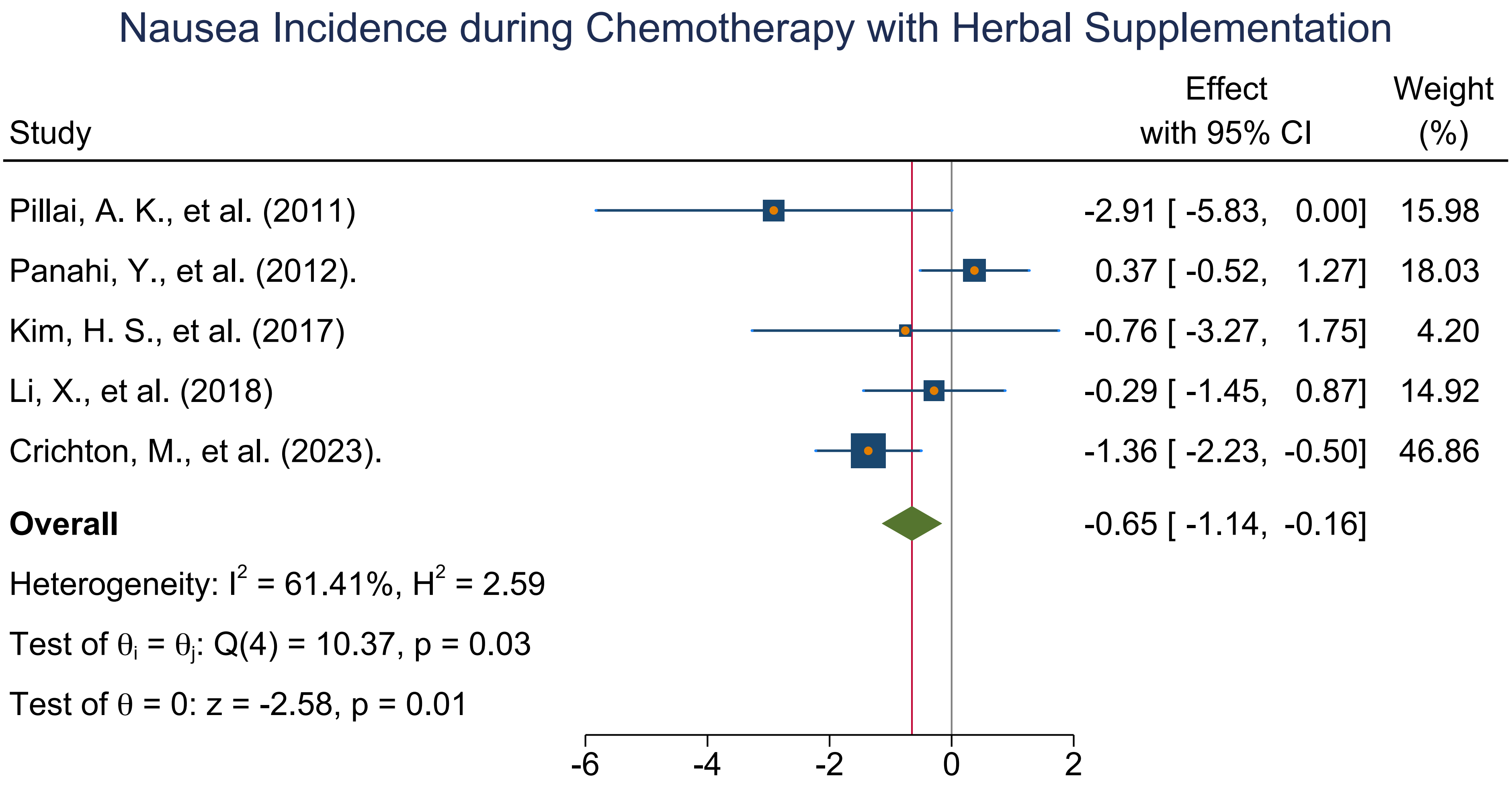
Nausea Incidence during Chemotherapy with Herbal Supplementation
Study
Effect with 95% CI
Weight (%)
Pillai, A. K., et al. (2011) -2.91 [-5.83, 0.00] 15.98
Panahi, Y., et al. (2012). 0.37 [-0.52, 1.27] 18.03
Kim, H. S., et al. (2017) -0.76 [-3.27, 1.75] 4.20
Li, X., et al. (2018) -0.29 [-1.45, 0.87] 14.92
Crichton, M., et al. (2023). -1.36 [-2.23, -0.50] 46.86
Overall -0.65 [-1.14, -0.16]
Heterogeneity: I^2 = 61.41%, H^2 = 2.59
Test of $\theta_i = \theta_j$: Q(4) = 10.37, p = 0.03
Test of $\theta = 0$: z = -2.58, p = 0.01
-6
-4
-2
0
2

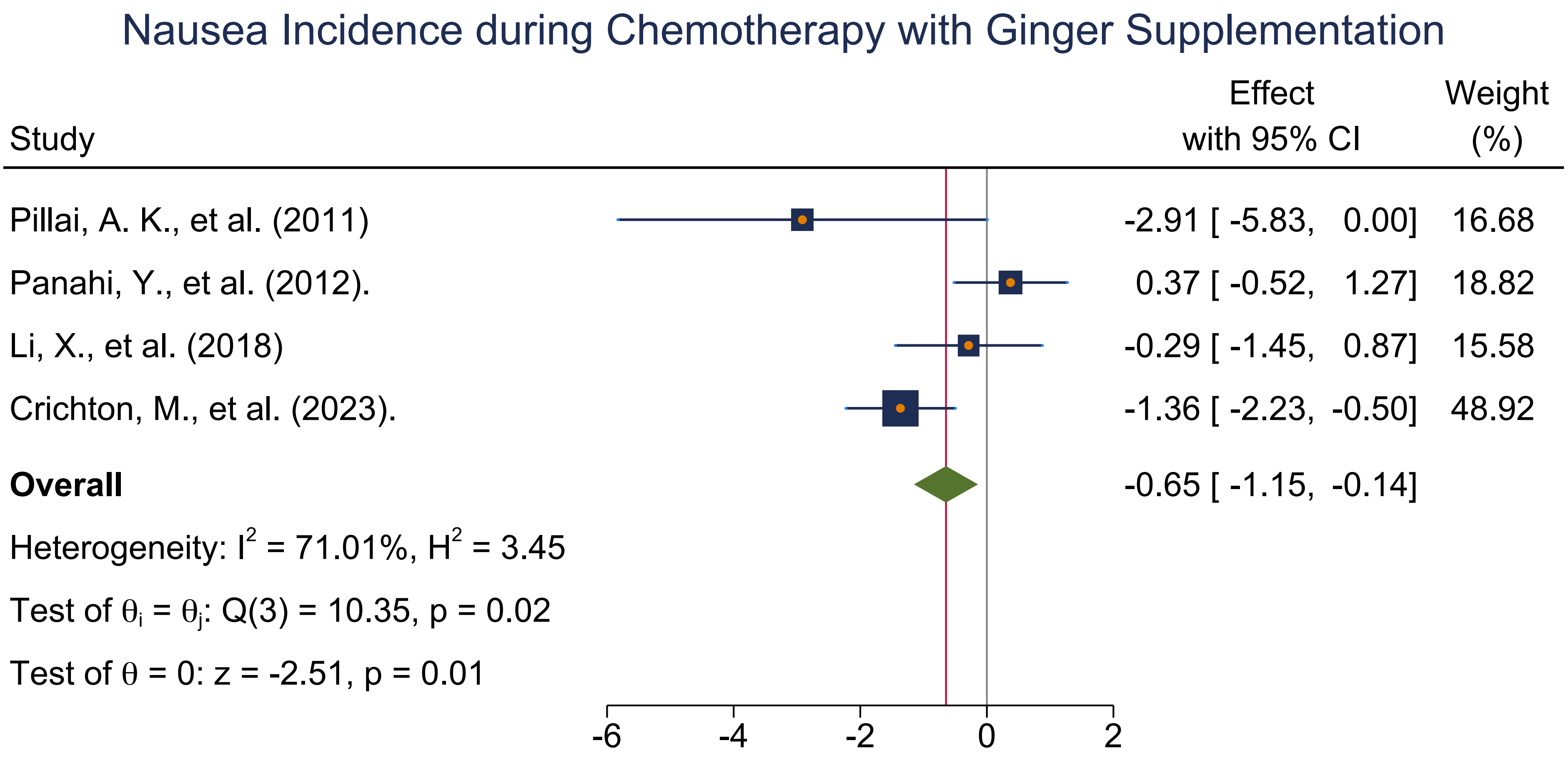
Nausea Incidence during Chemotherapy with Ginger Supplementation
Study
Effect with 95% CI
Weight (%)
Pillai, A. K., et al. (2011) -2.91 [-5.83, 0.00] 16.68
Panahi, Y., et al. (2012). 0.37 [-0.52, 1.27] 18.82
Li, X., et al. (2018) -0.29 [-1.45, 0.87] 15.58
Crichton, M., et al. (2023). -1.36 [-2.23, -0.50] 48.92
Overall -0.65 [-1.15, -0.14]
Heterogeneity: I^2 = 71.01%, H^2 = 3.45
Test of $\theta_i = \theta_j$: Q(3) = 10.35, p = 0.02
Test of θ = 0: z = -2.51, p = 0.01
-6
-4
-2
0
2

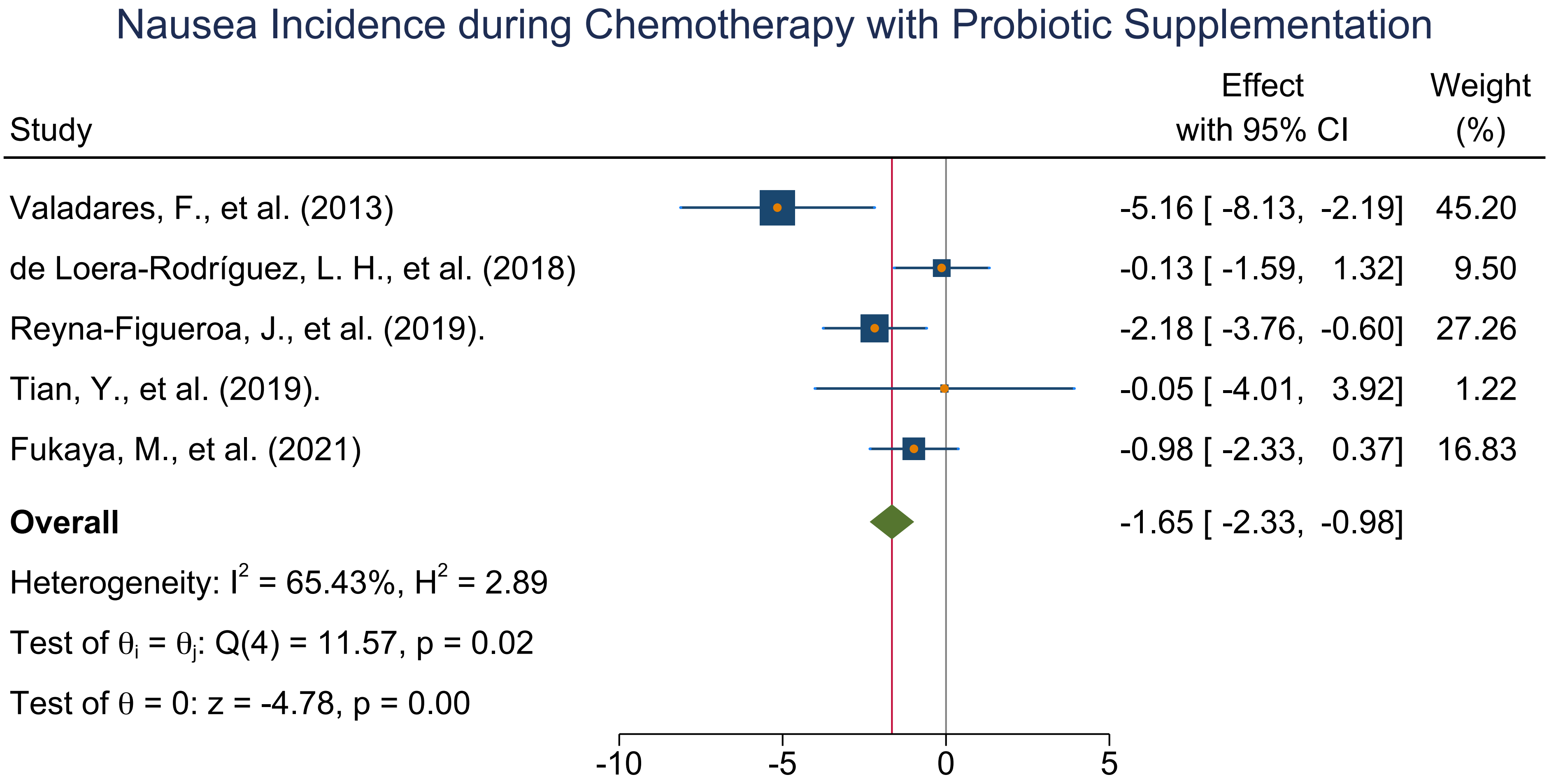
Nausea Incidence during Chemotherapy with Probiotic Supplementation
Study
Effect with 95% CI
Weight (%)
Valadares, F., et al. (2013) -5.16 [-8.13, -2.19] 45.20
de Loera-Rodríguez, L. H., et al. (2018) -0.13 [-1.59, 1.32] 9.50
Reyna-Figueroa, J., et al. (2019). -2.18 [-3.76, -0.60] 27.26
Tian, Y., et al. (2019). -0.05 [-4.01, 3.92] 1.22
Fukaya, M., et al. (2021) -0.98 [-2.33, 0.37] 16.83
Overall -1.65 [-2.33, -0.98]
Heterogeneity: I^2 = 65.43%, H^2 = 2.89
Test of $\theta_i = \theta_j$: Q(4) = 11.57, p = 0.02
Test of θ = 0: z = -4.78, p = 0.00
-10
-5
0
5

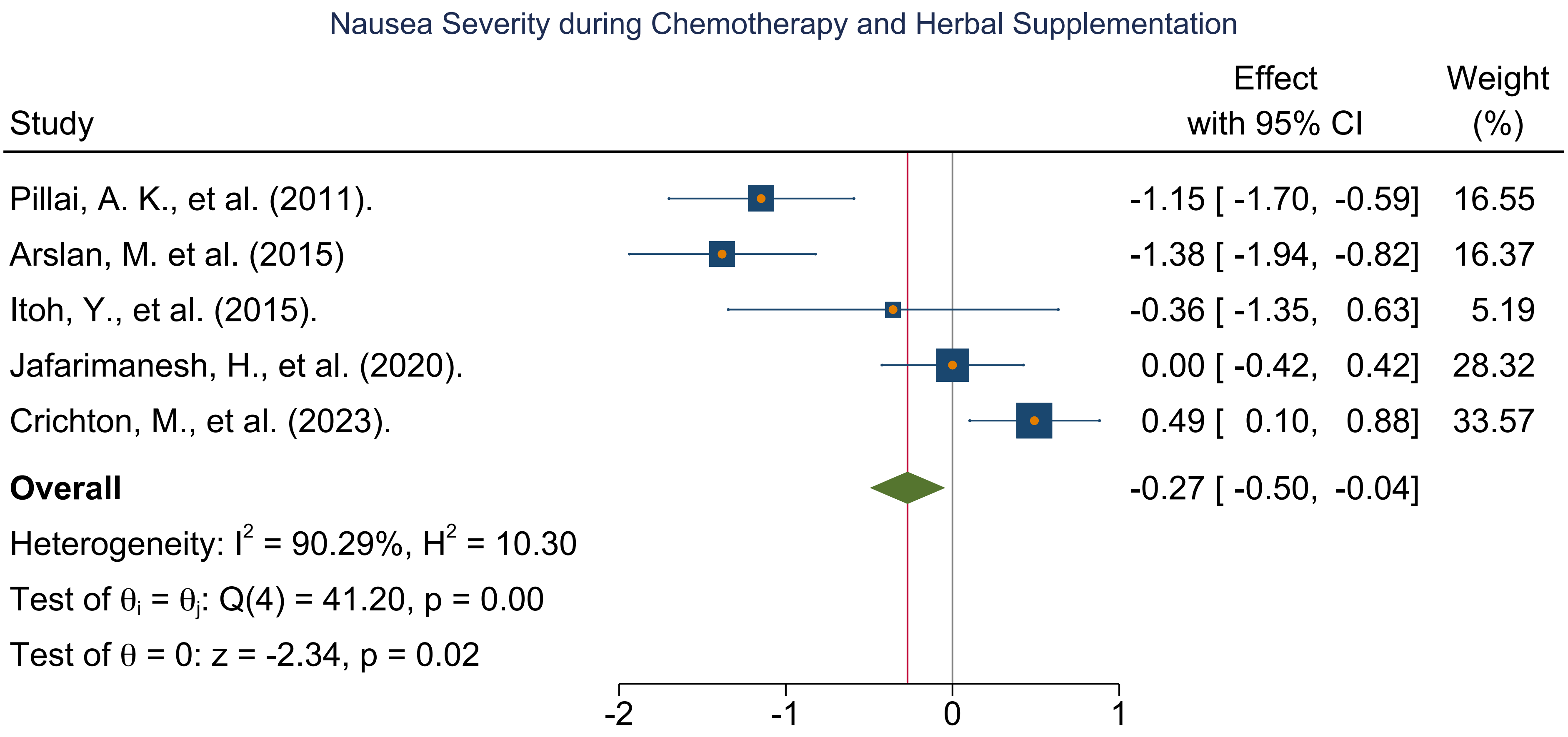
Nausea Severity during Chemotherapy and Herbal Supplementation
Study
Effect with 95% CI
Weight (%)
Pillai, A. K., et al. (2011). -1.15 [-1.70, -0.59] 16.55
Arslan, M. et al. (2015) -1.38 [-1.94, -0.82] 16.37
Itoh, Y., et al. (2015). -0.36 [-1.35, 0.63] 5.19
Jafarimanesh, H., et al. (2020). 0.00 [-0.42, 0.42] 28.32
Crichton, M., et al. (2023). 0.49 [0.10, 0.88] 33.57
Overall -0.27 [-0.50, -0.04]
Heterogeneity: I2 = 90.29%, H2 = 10.30
Test of θi = θj: Q(4) = 41.20, p = 0.00
Test of θ = 0: z = -2.34, p = 0.02
-2
-1
0
1

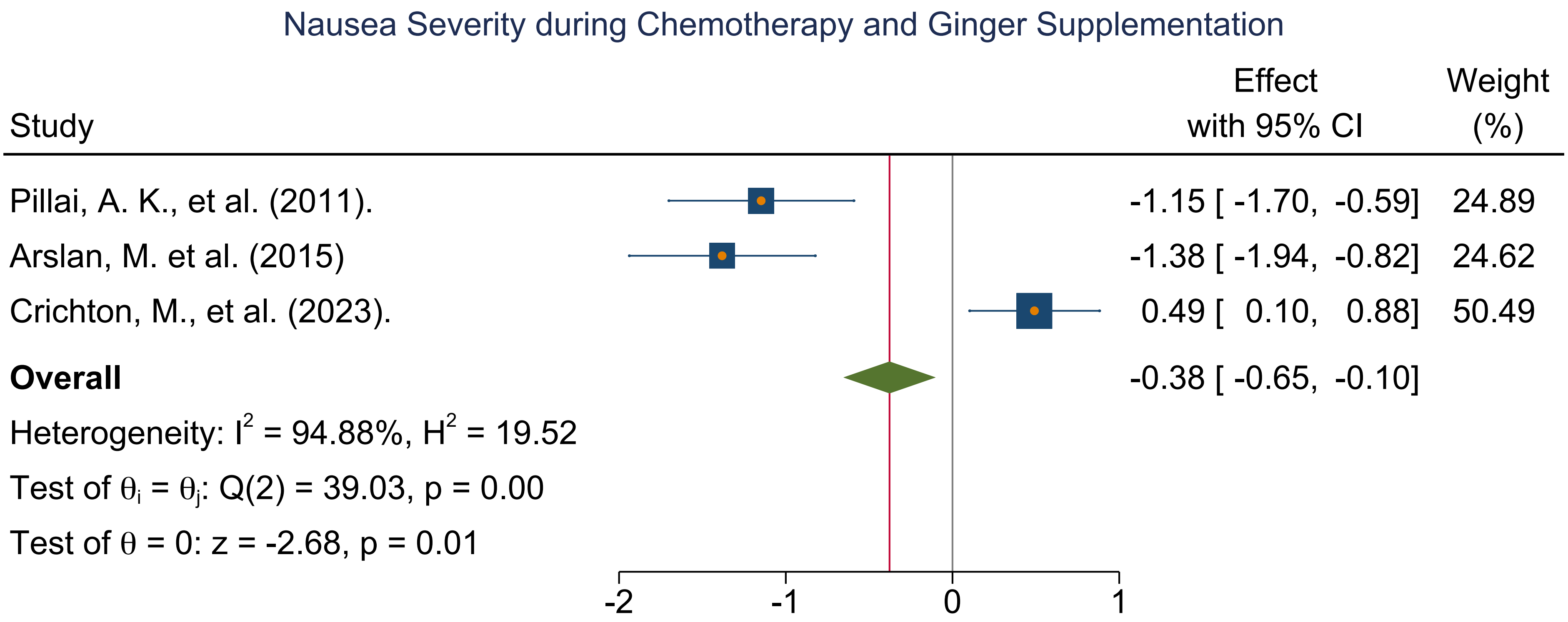
Nausea Severity during Chemotherapy and Ginger Supplementation
Study
Effect with 95% CI
Weight (%)
Pillai, A. K., et al. (2011). -1.15 [-1.70, -0.59] 24.89
Arslan, M. et al. (2015) -1.38 [-1.94, -0.82] 24.62
Crichton, M., et al. (2023). 0.49 [0.10, 0.88] 50.49
Overall -0.38 [-0.65, -0.10]
Heterogeneity: I^2 = 94.88%, H^2 = 19.52
Test of $\theta_i = \theta_j$: Q(2) = 39.03, p = 0.00
Test of θ = 0: z = -2.68, p = 0.01
-2
-1
0
1

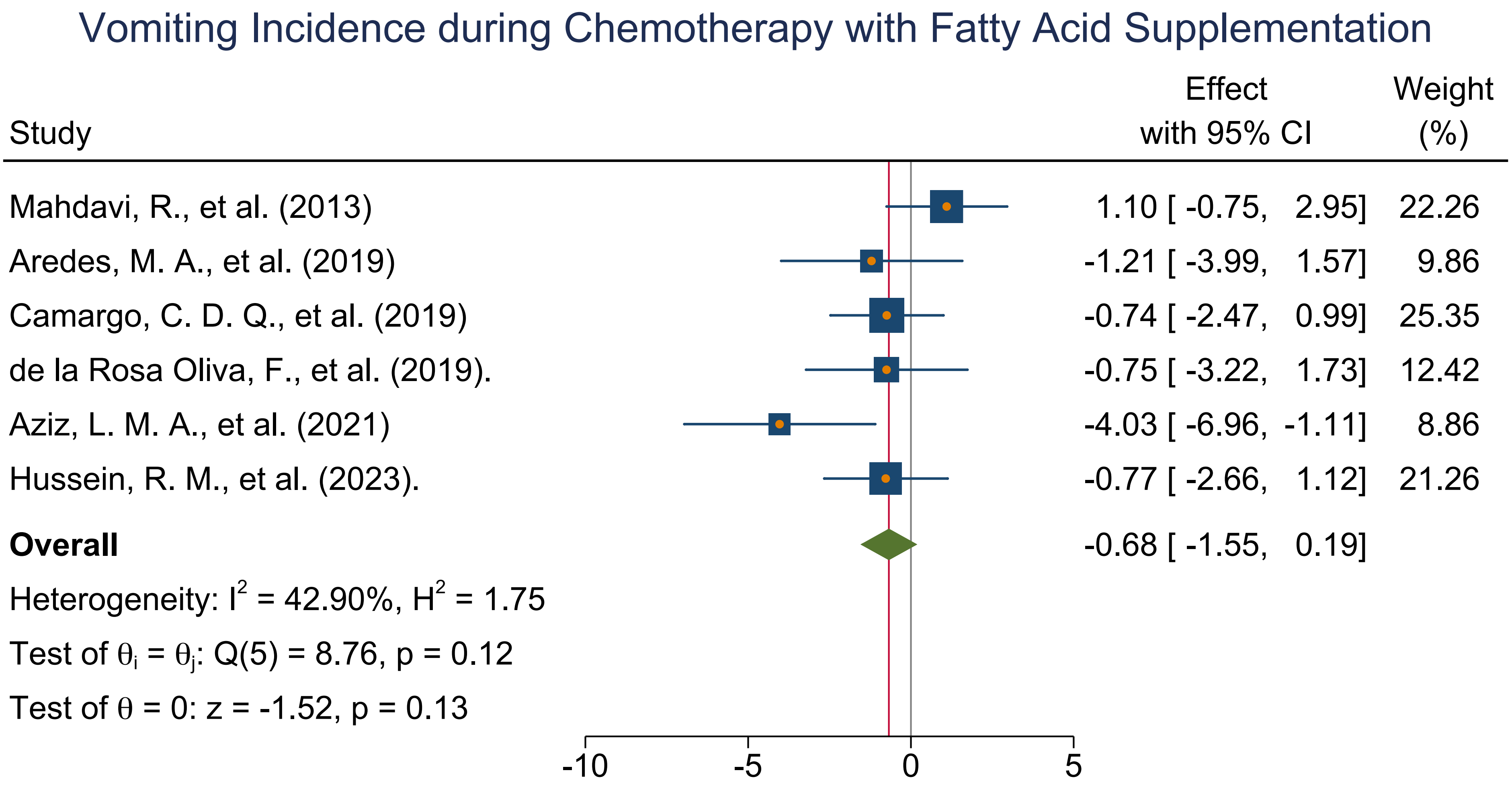
Vomiting Incidence during Chemotherapy with Fatty Acid Supplementation
Study
Effect with 95% CI
Weight (%)
Mahdavi, R., et al. (2013) 1.10 [-0.75, 2.95] 22.26
Aredes, M. A., et al. (2019) -1.21 [-3.99, 1.57] 9.86
Camargo, C. D. Q., et al. (2019) -0.74 [-2.47, 0.99] 25.35
de la Rosa Oliva, F., et al. (2019). -0.75 [-3.22, 1.73] 12.42
Aziz, L. M. A., et al. (2021) -4.03 [-6.96, -1.11] 8.86
Hussein, R. M., et al. (2023). -0.77 [-2.66, 1.12] 21.26
Overall -0.68 [-1.55, 0.19]
Heterogeneity: I^2 = 42.90%, H^2 = 1.75
Test of $\theta_i = \theta_j$: Q(5) = 8.76, p = 0.12
Test of θ = 0: z = -1.52, p = 0.13
-10
-5
0
5

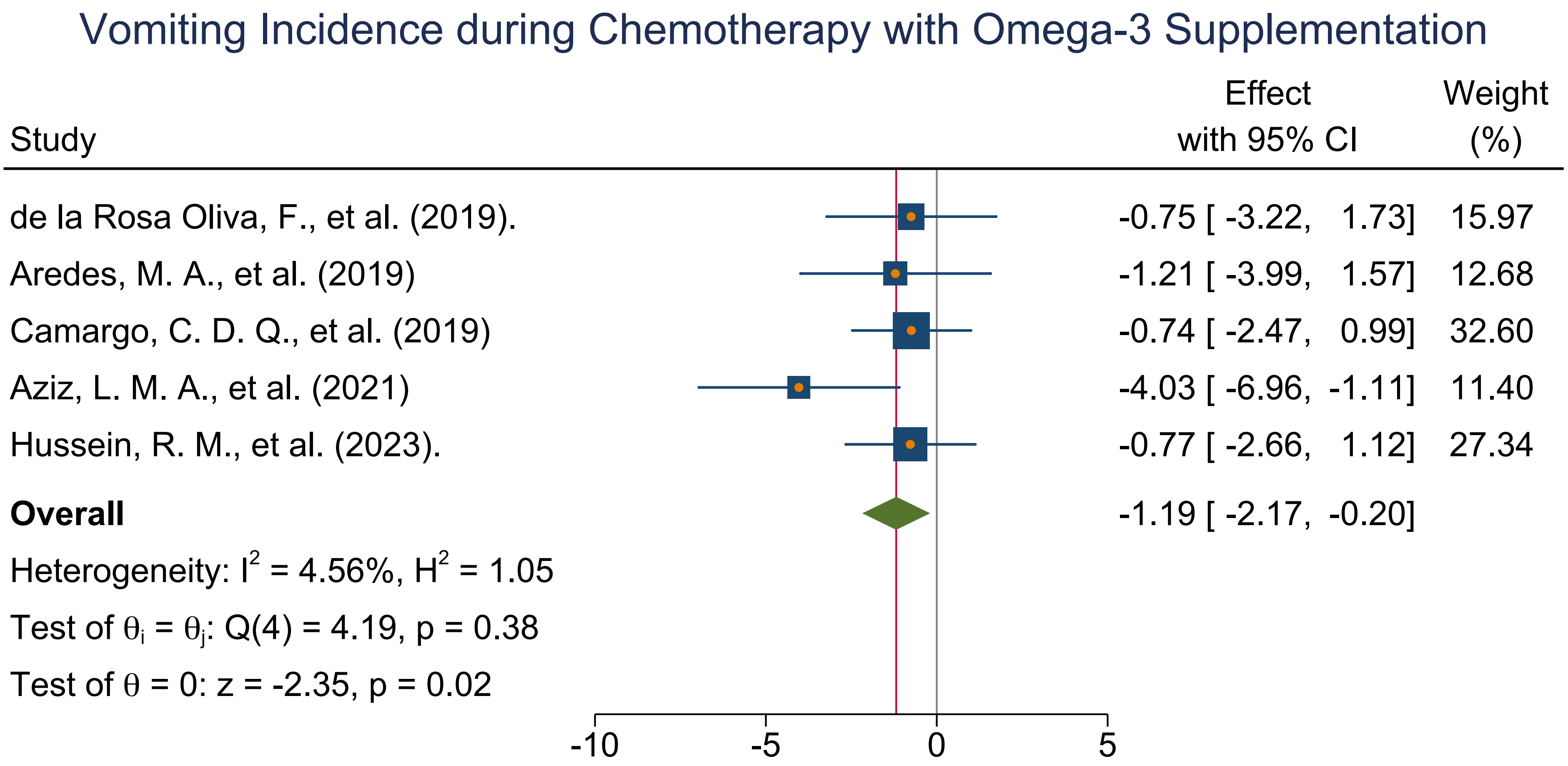
Vomiting Incidence during Chemotherapy with Omega-3 Supplementation
Study
Effect with 95% CI
Weight (%)
de la Rosa Oliva, F., et al. (2019).
-0.75 [-3.22, 1.73]
15.97
Aredes, M. A., et al. (2019)
-1.21 [-3.99, 1.57]
12.68
Camargo, C. D. Q., et al. (2019)
-0.74 [-2.47, 0.99]
32.60
Aziz, L. M. A., et al. (2021)
-4.03 [-6.96, -1.11]
11.40
Hussein, R. M., et al. (2023).
-0.77 [-2.66, 1.12]
27.34
Overall
-1.19 [-2.17, -0.20]
Heterogeneity: I^2 = 4.56%, H^2 = 1.05
Test of $\theta_i = \theta_j$: Q(4) = 4.19, p = 0.38
Test of θ = 0: z = -2.35, p = 0.02
-10
-5
0
5

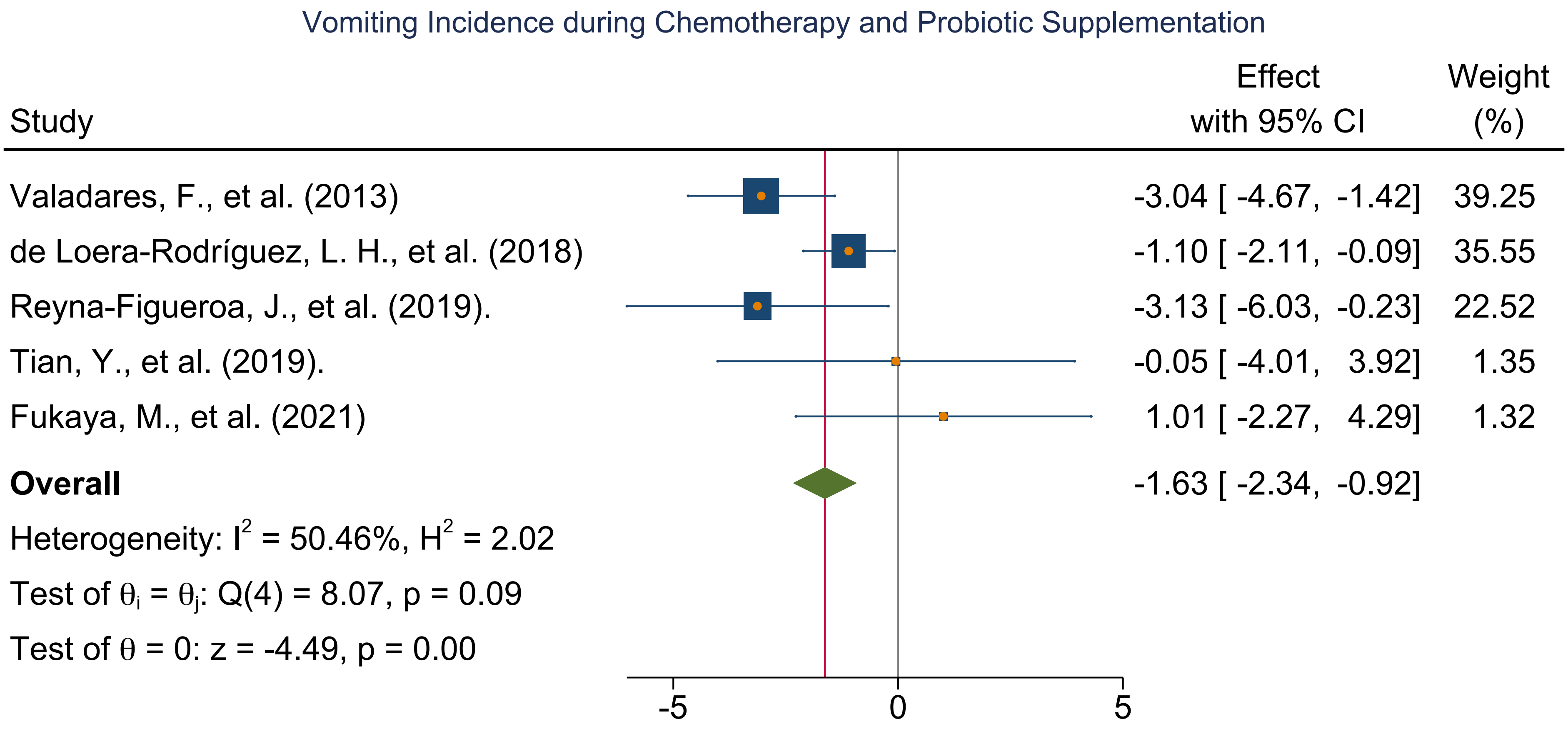
Vomiting Incidence during Chemotherapy and Probiotic Supplementation
Study
Effect with 95% CI
Weight (%)
Valadares, F., et al. (2013) -3.04 [-4.67, -1.42] 39.25
de Loera-Rodríguez, L. H., et al. (2018) -1.10 [-2.11, -0.09] 35.55
Reyna-Figueroa, J., et al. (2019). -3.13 [-6.03, -0.23] 22.52
Tian, Y., et al. (2019). -0.05 [-4.01, 3.92] 1.35
Fukaya, M., et al. (2021) 1.01 [-2.27, 4.29] 1.32
Overall -1.63 [-2.34, -0.92]
Heterogeneity: I^2 = 50.46%, H^2 = 2.02
Test of $\theta_i = \theta_j$: Q(4) = 8.07, p = 0.09
Test of θ = 0: z = -4.49, p = 0.00
-5
0
5

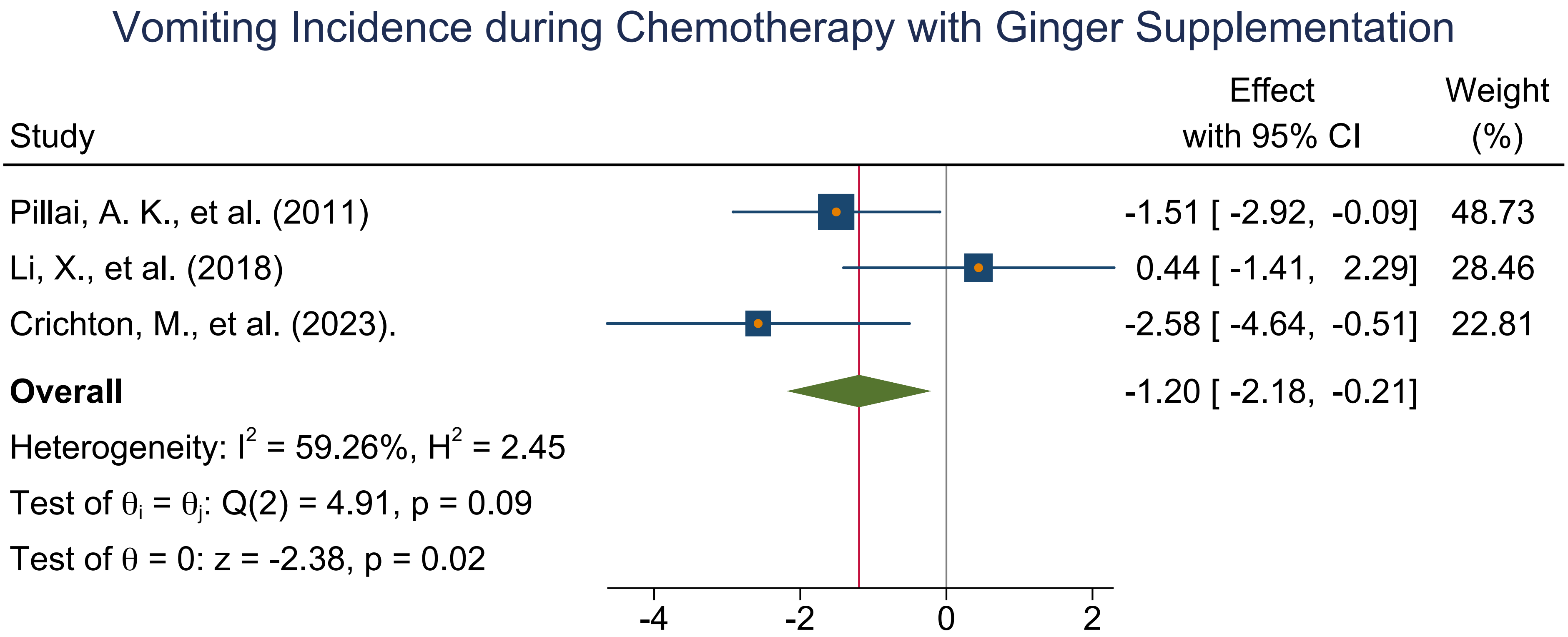

Vomiting Incidence during Chemotherapy with Ginger Supplementation
Study
Effect with 95% CI
Weight (%)
Pillai, A. K., et al. (2011)
-1.51 [-2.92, -0.09]
48.73
Li, X., et al. (2018)
0.44 [-1.41, 2.29]
28.46
Crichton, M., et al. (2023).
-2.58 [-4.64, -0.51]
22.81
Overall
-1.20 [-2.18, -0.21]
Heterogeneity: I^2 = 59.26%, H^2 = 2.45
Test of $\theta_i = \theta_j$: Q(2) = 4.91, p = 0.09
Test of θ = 0: z = -2.38, p = 0.02
-4
-2
0
2

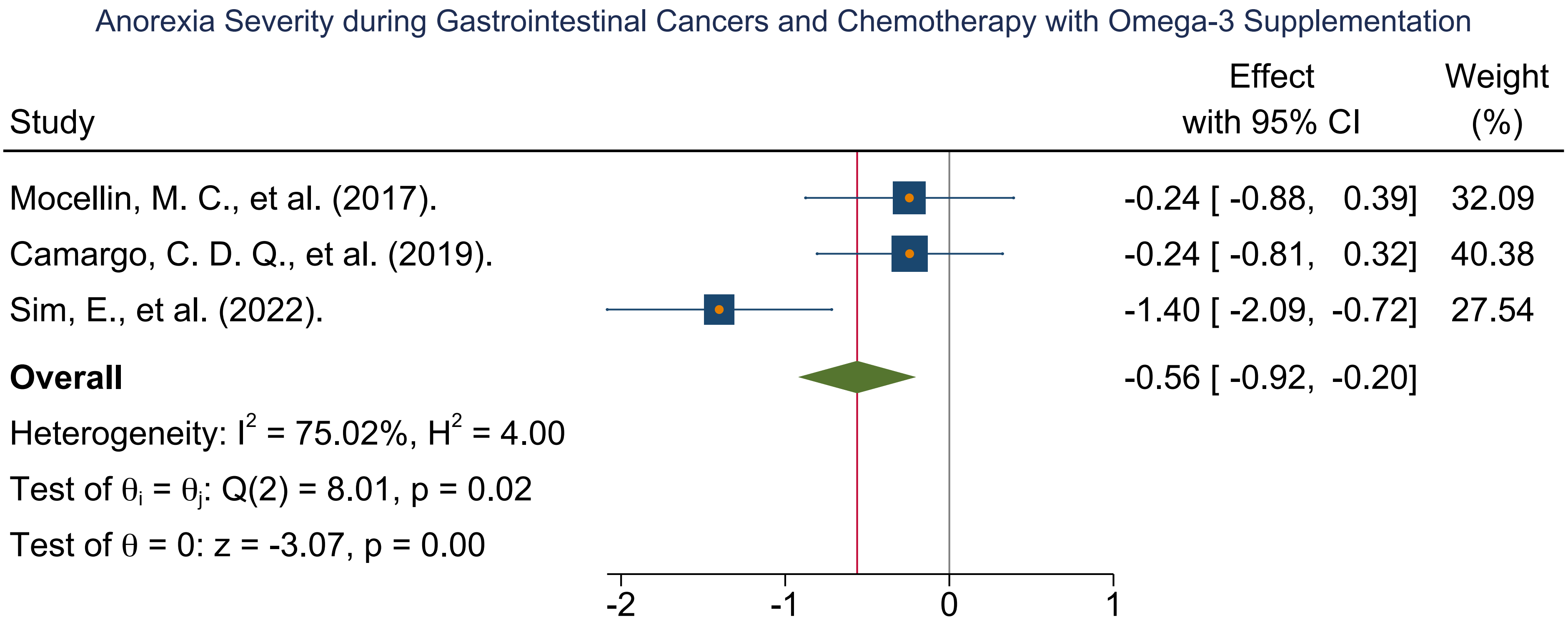

Anorexia Severity during Gastrointestinal Cancers and Chemotherapy with Omega-3 Supplementation
Study
Effect with 95% CI
Weight (%)
Mocellin, M. C., et al. (2017). -0.24 [-0.88, 0.39] 32.09
Camargo, C. D. Q., et al. (2019). -0.24 [-0.81, 0.32] 40.38
Sim, E., et al. (2022). -1.40 [-2.09, -0.72] 27.54
Overall -0.56 [-0.92, -0.20]
Heterogeneity: I^2 = 75.02%, H^2 = 4.00
Test of $\theta_i = \theta_j$: Q(2) = 8.01, p = 0.02
Test of θ = 0: z = -3.07, p = 0.00
-2
-1
0
1

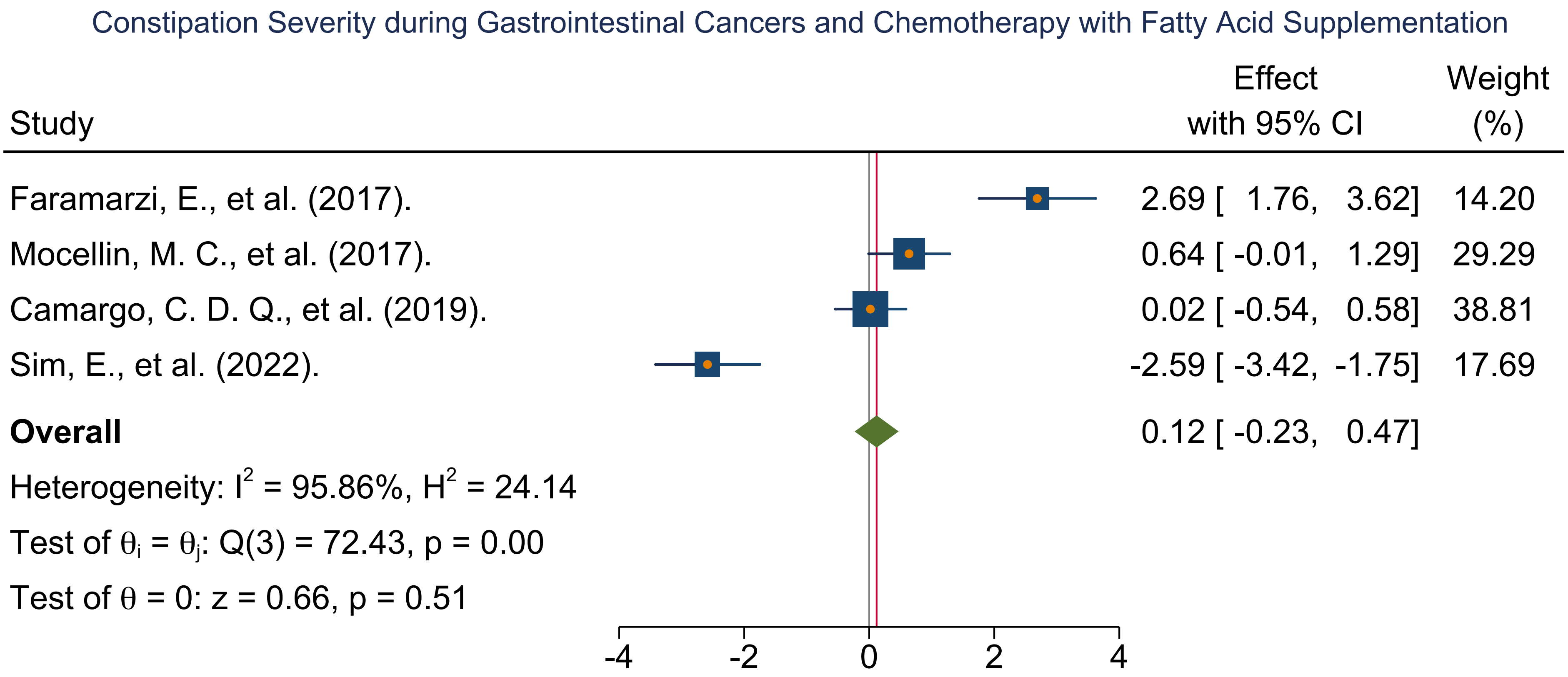

Constipation Severity during Gastrointestinal Cancers and Chemotherapy with Fatty Acid Supplementation
Study
Effect with 95% CI
Weight (%)
Faramarzi, E., et al. (2017). 2.69 [1.76, 3.62] 14.20
Mocellin, M. C., et al. (2017). 0.64 [-0.01, 1.29] 29.29
Camargo, C. D. Q., et al. (2019). 0.02 [-0.54, 0.58] 38.81
Sim, E., et al. (2022). -2.59 [-3.42, -1.75] 17.69
Overall 0.12 [-0.23, 0.47]
Heterogeneity: I^2 = 95.86%, H^2 = 24.14
Test of $\theta_i = \theta_j$: Q(3) = 72.43, p = 0.00
Test of θ = 0: z = 0.66, p = 0.51
-4
-2
0
2
4

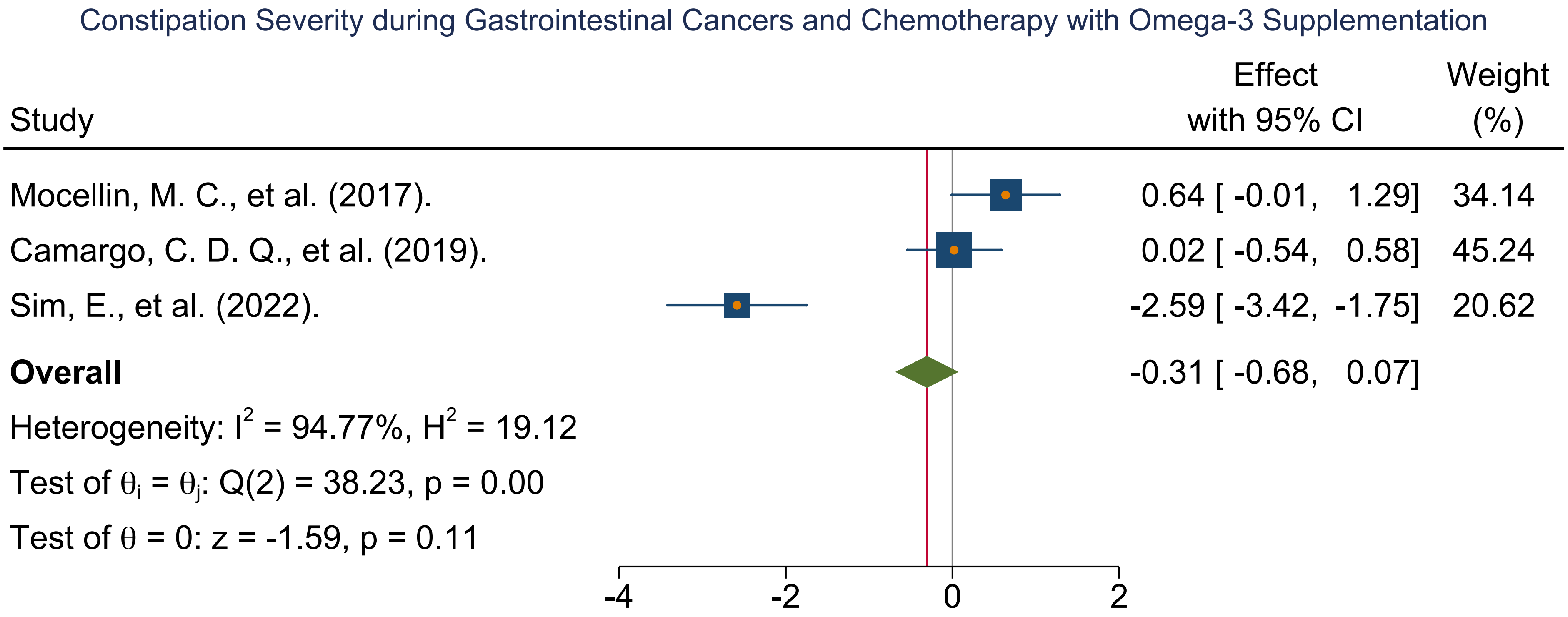
Constipation Severity during Gastrointestinal Cancers and Chemotherapy with Omega-3 Supplementation
Study
Effect with 95% CI
Weight (%)
Mocellin, M. C., et al. (2017). 0.64 [-0.01, 1.29] 34.14
Camargo, C. D. Q., et al. (2019). 0.02 [-0.54, 0.58] 45.24
Sim, E., et al. (2022). -2.59 [-3.42, -1.75] 20.62
Overall -0.31 [-0.68, 0.07]
Heterogeneity: I² = 94.77%, H² = 19.12
Test of θi = θj: Q(2) = 38.23, p = 0.00
Test of θ = 0: z = -1.59, p = 0.11
-4
-2
0
2

Defecation Latency (hrs.) during Colorectal Cancers and Surgical Treatment with Gum Supplementation

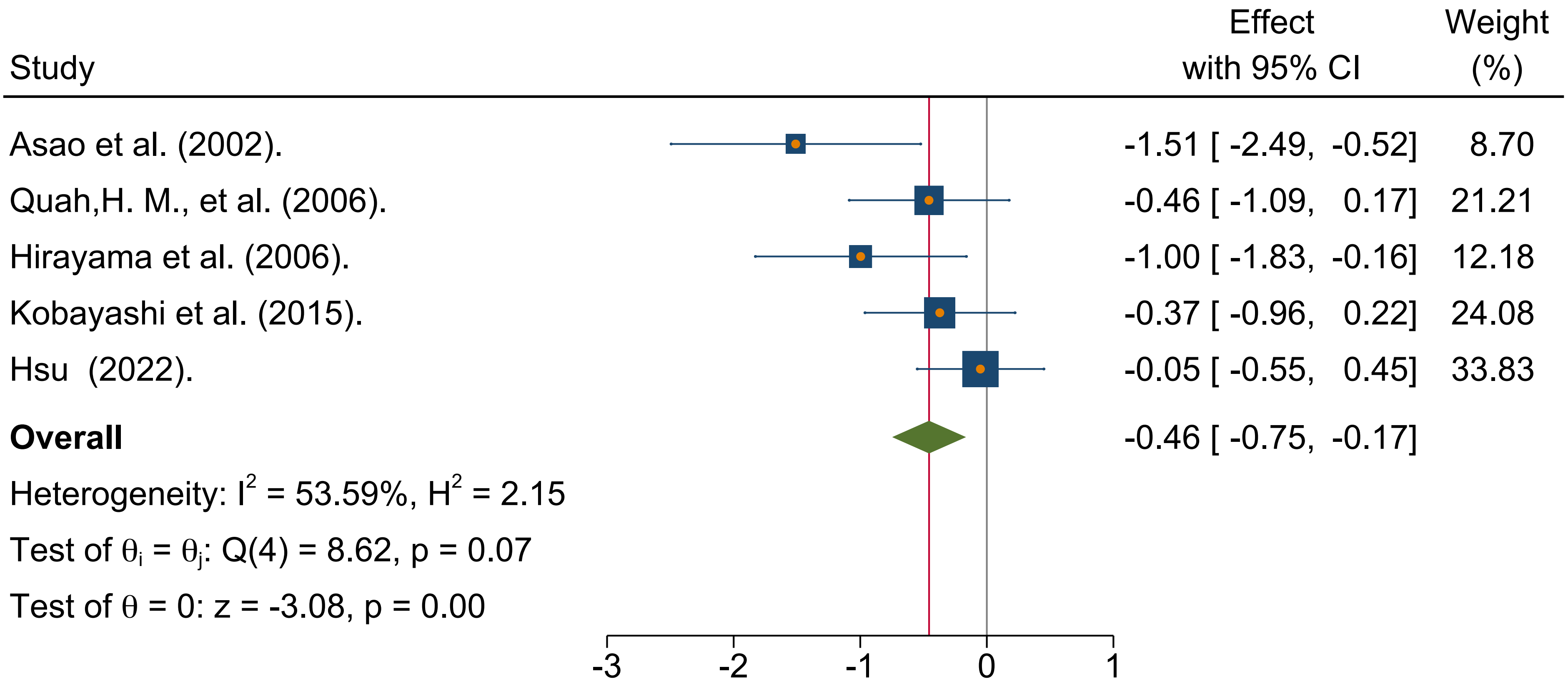

Diarrhea Incidence during Gastrointestinal Cancer and Chemotherapy with Amino Acid Supplementation

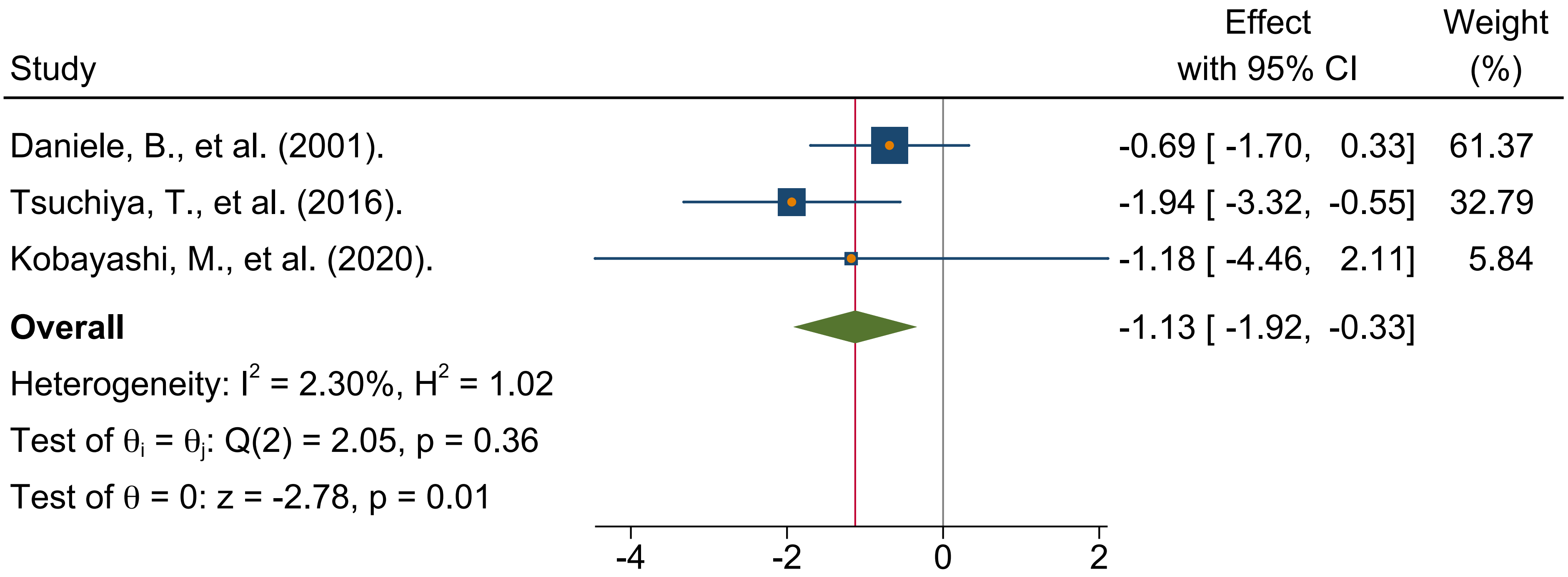

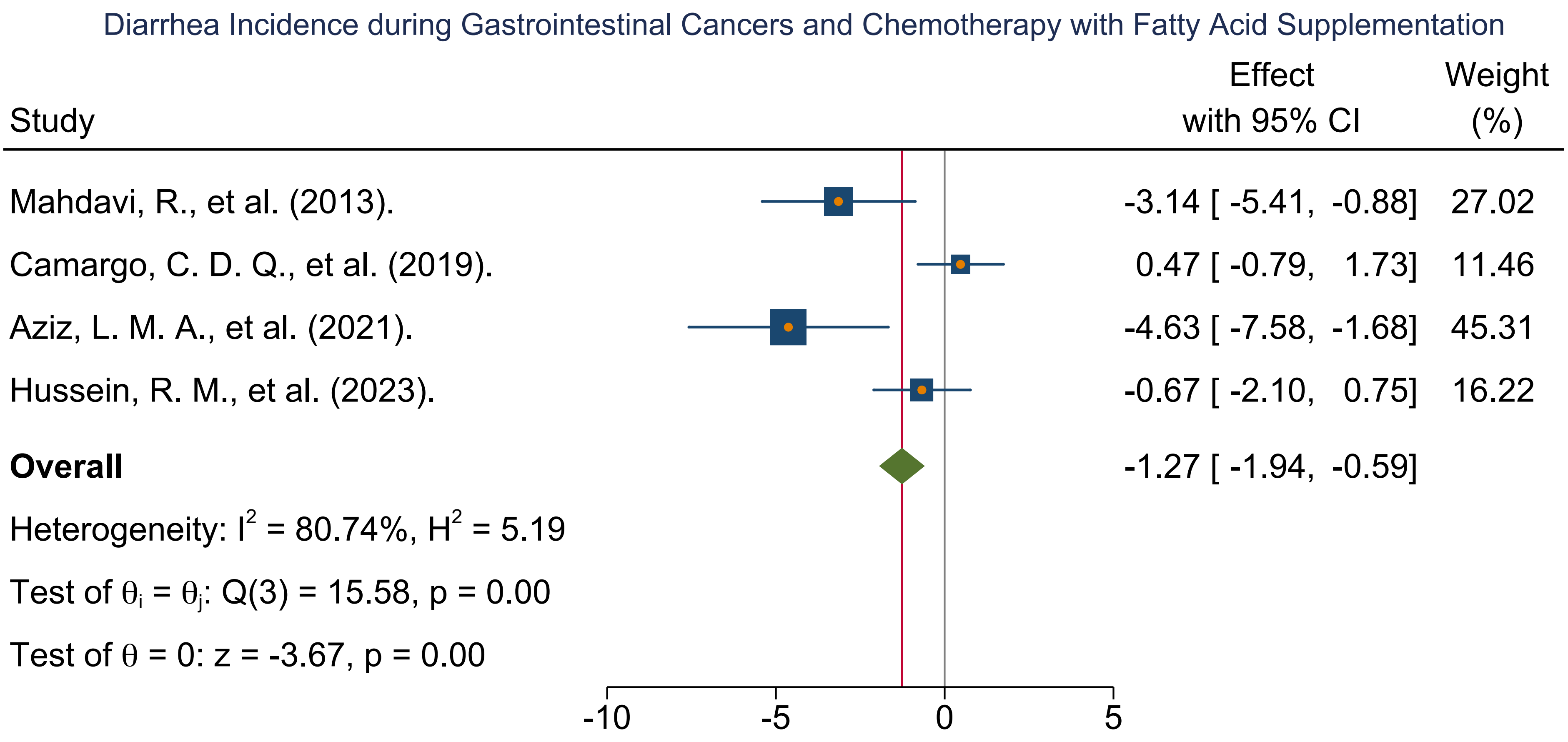
Diarrhea Incidence during Gastrointestinal Cancers and Chemotherapy with Fatty Acid Supplementation
Study
Effect with 95% CI
Weight (%)
Mahdavi, R., et al. (2013). -3.14 [-5.41, -0.88] 27.02
Camargo, C. D. Q., et al. (2019). 0.47 [-0.79, 1.73] 11.46
Aziz, L. M. A., et al. (2021). -4.63 [-7.58, -1.68] 45.31
Hussein, R. M., et al. (2023). -0.67 [-2.10, 0.75] 16.22
Overall -1.27 [-1.94, -0.59]
Heterogeneity: I^2 = 80.74%, H^2 = 5.19
Test of $\theta_i = \theta_j$: Q(3) = 15.58, p = 0.00
Test of θ = 0: z = -3.67, p = 0.00
-10
-5
0
5

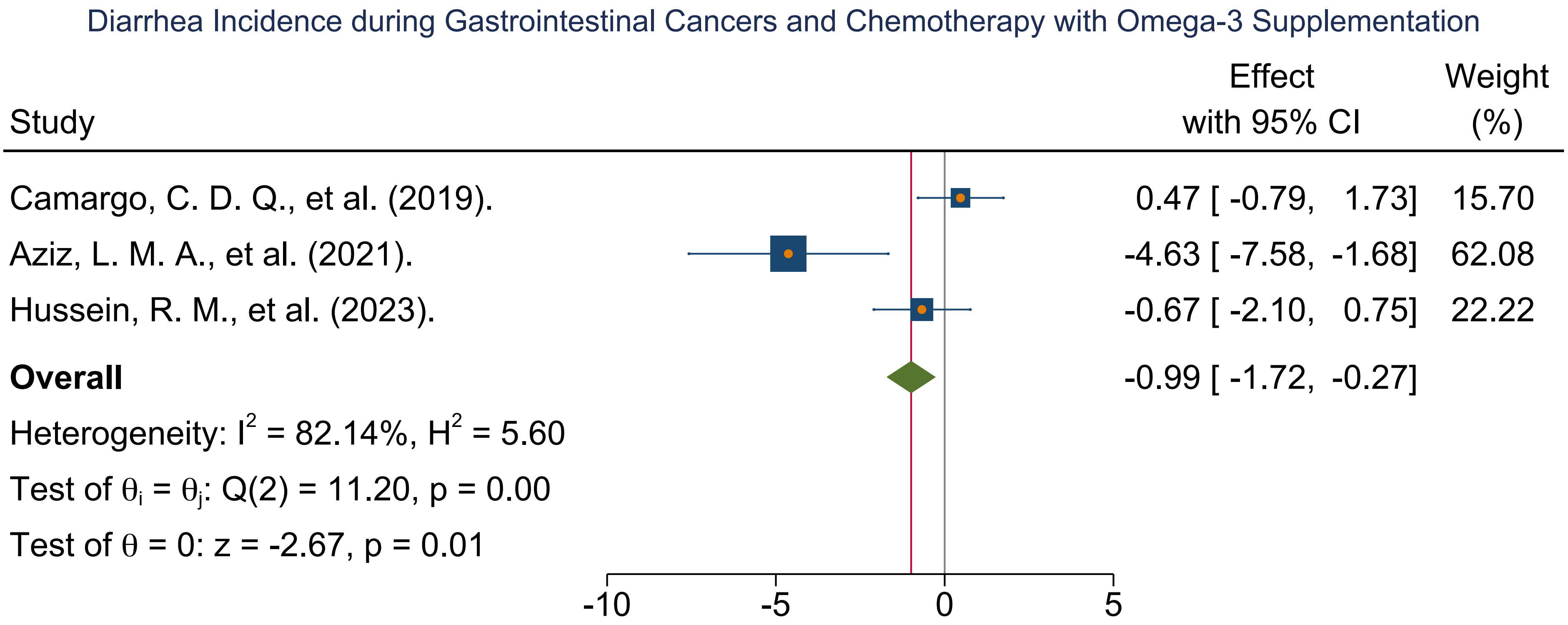
Diarrhea Incidence during Gastrointestinal Cancers and Chemotherapy with Omega-3 Supplementation
Study
Effect with 95% CI
Weight (%)
Camargo, C. D. Q., et al. (2019). 0.47 [-0.79, 1.73] 15.70
Aziz, L. M. A., et al. (2021). -4.63 [-7.58, -1.68] 62.08
Hussein, R. M., et al. (2023). -0.67 [-2.10, 0.75] 22.22
Overall -0.99 [-1.72, -0.27]
Heterogeneity: I^2 = 82.14%, H^2 = 5.60
Test of $\theta_i = \theta_j$: Q(2) = 11.20, p = 0.00
Test of θ = 0: z = -2.67, p = 0.01
-10
-5
0
5

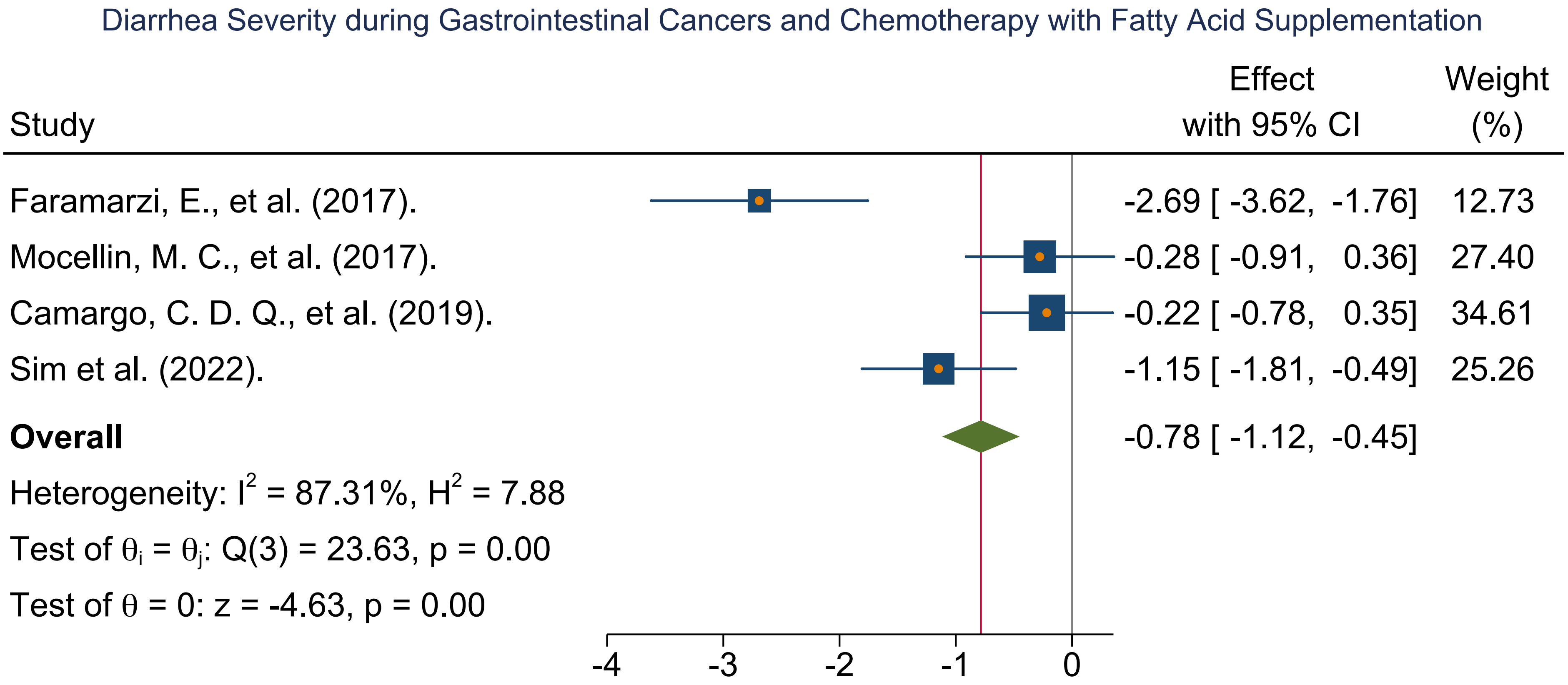
Diarrhea Severity during Gastrointestinal Cancers and Chemotherapy with Fatty Acid Supplementation
Study
Effect with 95% CI
Weight (%)
Faramarzi, E., et al. (2017). -2.69 [-3.62, -1.76] 12.73
Mocellin, M. C., et al. (2017). -0.28 [-0.91, 0.36] 27.40
Camargo, C. D. Q., et al. (2019). -0.22 [-0.78, 0.35] 34.61
Sim et al. (2022). -1.15 [-1.81, -0.49] 25.26
Overall -0.78 [-1.12, -0.45]
Heterogeneity: I² = 87.31%, H² = 7.88
Test of θi = θj: Q(3) = 23.63, p = 0.00
Test of θ = 0: z = -4.63, p = 0.00
-4
-3
-2
-1
0

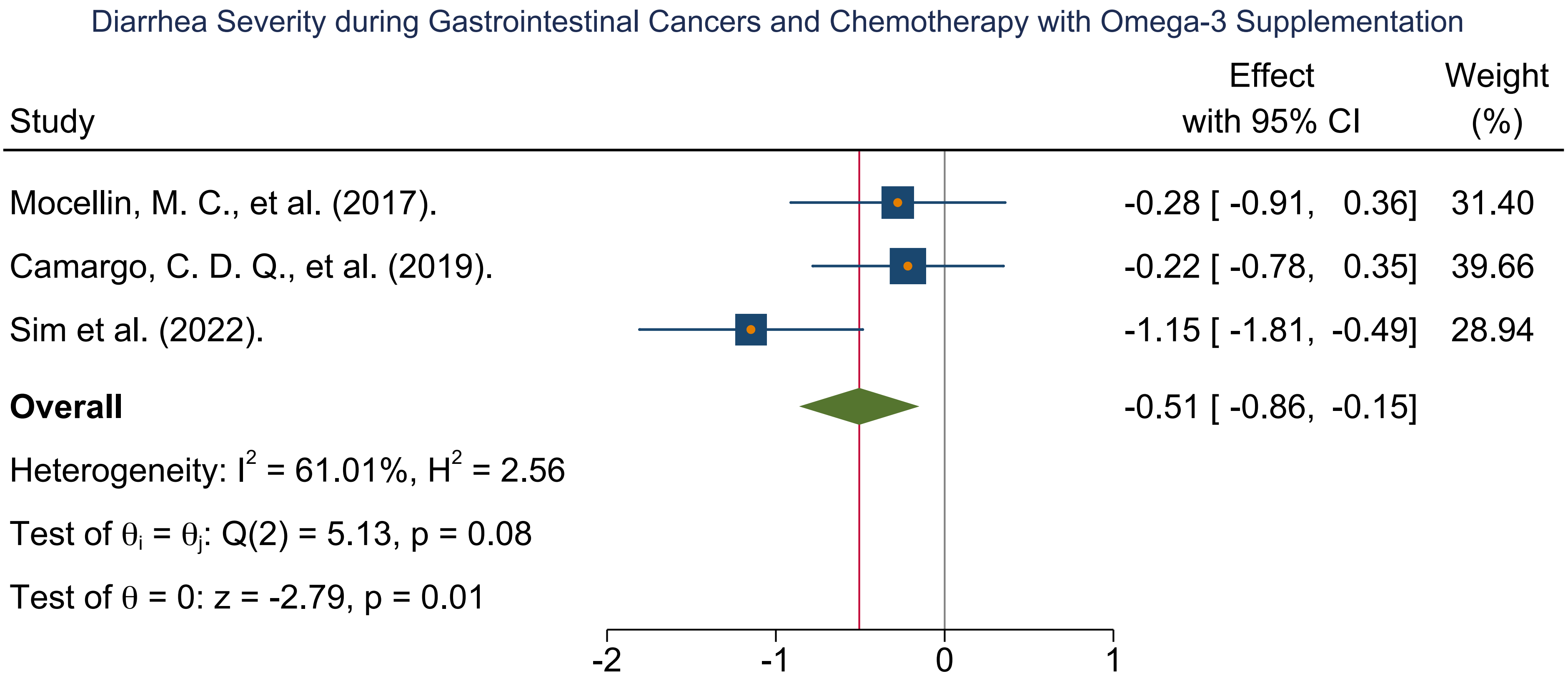

Diarrhea Severity during Gastrointestinal Cancers and Chemotherapy with Omega-3 Supplementation
Study
Effect with 95% CI
Weight (%)
Mocellin, M. C., et al. (2017). -0.28 [-0.91, 0.36] 31.40
Camargo, C. D. Q., et al. (2019). -0.22 [-0.78, 0.35] 39.66
Sim et al. (2022). -1.15 [-1.81, -0.49] 28.94
Overall -0.51 [-0.86, -0.15]
Heterogeneity: I2 = 61.01%, H2 = 2.56
Test of θi = θj: Q(2) = 5.13, p = 0.08
Test of θ = 0: z = -2.79, p = 0.01
-2
-1
0
1

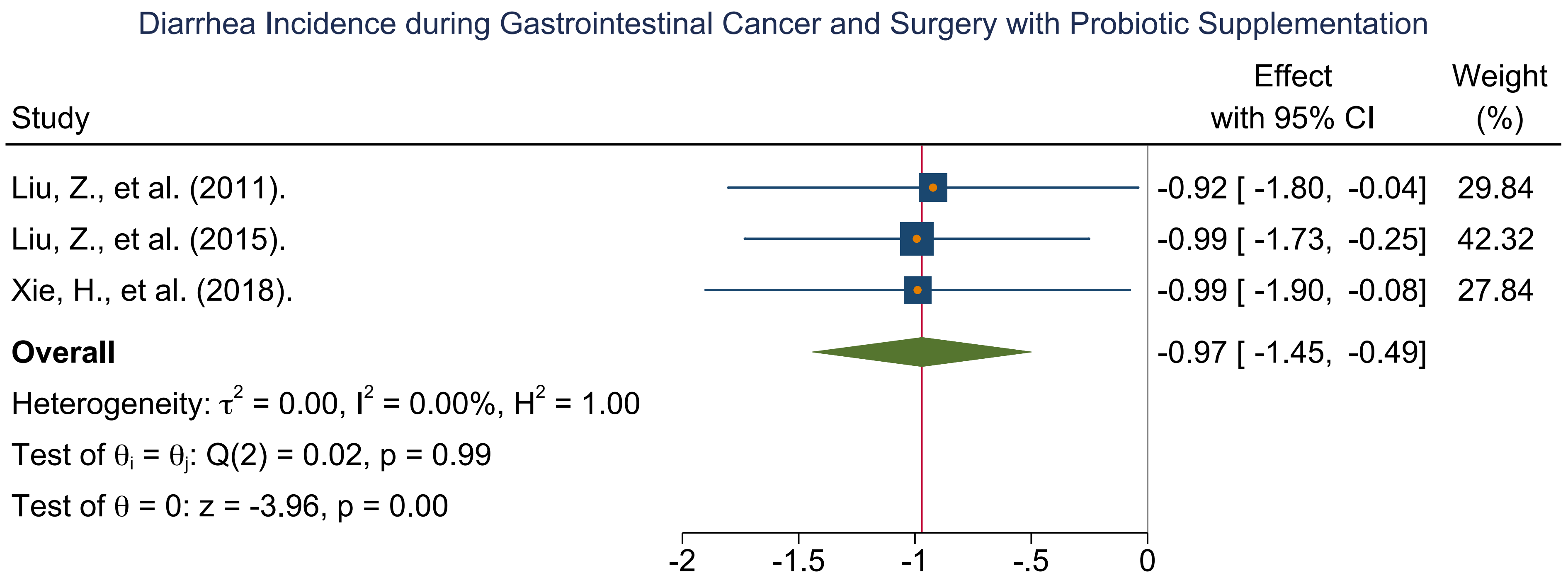
Diarrhea Incidence during Gastrointestinal Cancer and Surgery with Probiotic Supplementation
Study
Effect with 95% CI
Weight (%)
Liu, Z., et al. (2011). -0.92 [-1.80, -0.04] 29.84
Liu, Z., et al. (2015). -0.99 [-1.73, -0.25] 42.32
Xie, H., et al. (2018). -0.99 [-1.90, -0.08] 27.84
Overall -0.97 [-1.45, -0.49]
Heterogeneity: τ^2 = 0.00, I^2 = 0.00%, H^2 = 1.00
Test of $\theta_i = \theta_j$: Q(2) = 0.02, p = 0.99
Test of θ = 0: z = -3.96, p = 0.00
-2
-1.5
-1
-.5
0

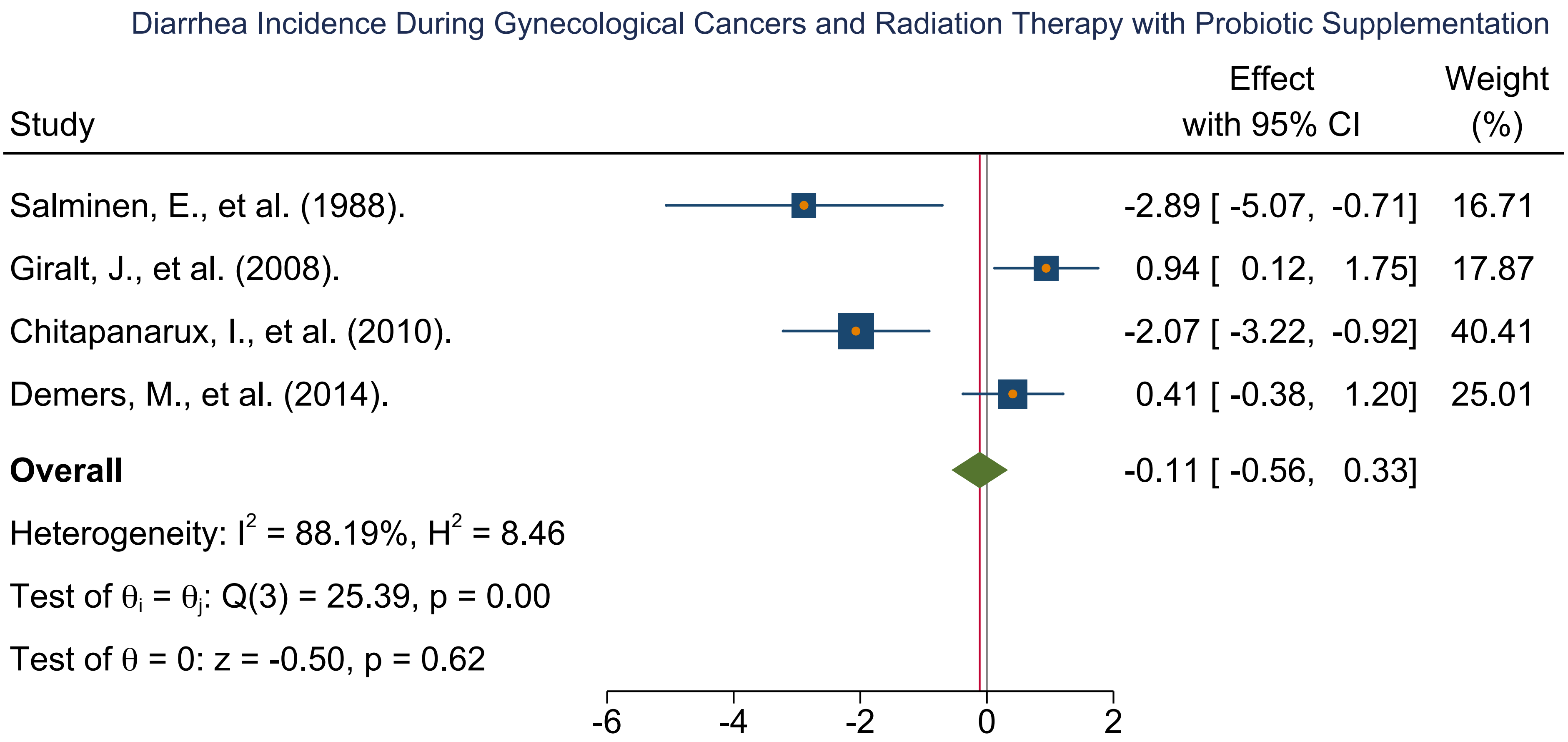
Diarrhea Incidence During Gynecological Cancers and Radiation Therapy with Probiotic Supplementation
Study
Effect with 95% CI
Weight (%)
Salminen, E., et al. (1988). -2.89 [-5.07, -0.71] 16.71
Giralt, J., et al. (2008). 0.94 [0.12, 1.75] 17.87
Chitapanarux, I., et al. (2010). -2.07 [-3.22, -0.92] 40.41
Demers, M., et al. (2014). 0.41 [-0.38, 1.20] 25.01
Overall -0.11 [-0.56, 0.33]
Heterogeneity: I^2 = 88.19%, H^2 = 8.46
Test of $\theta_i = \theta_j$: Q(3) = 25.39, p = 0.00
Test of θ = 0: z = -0.50, p = 0.62
-6
-4
-2
0
2

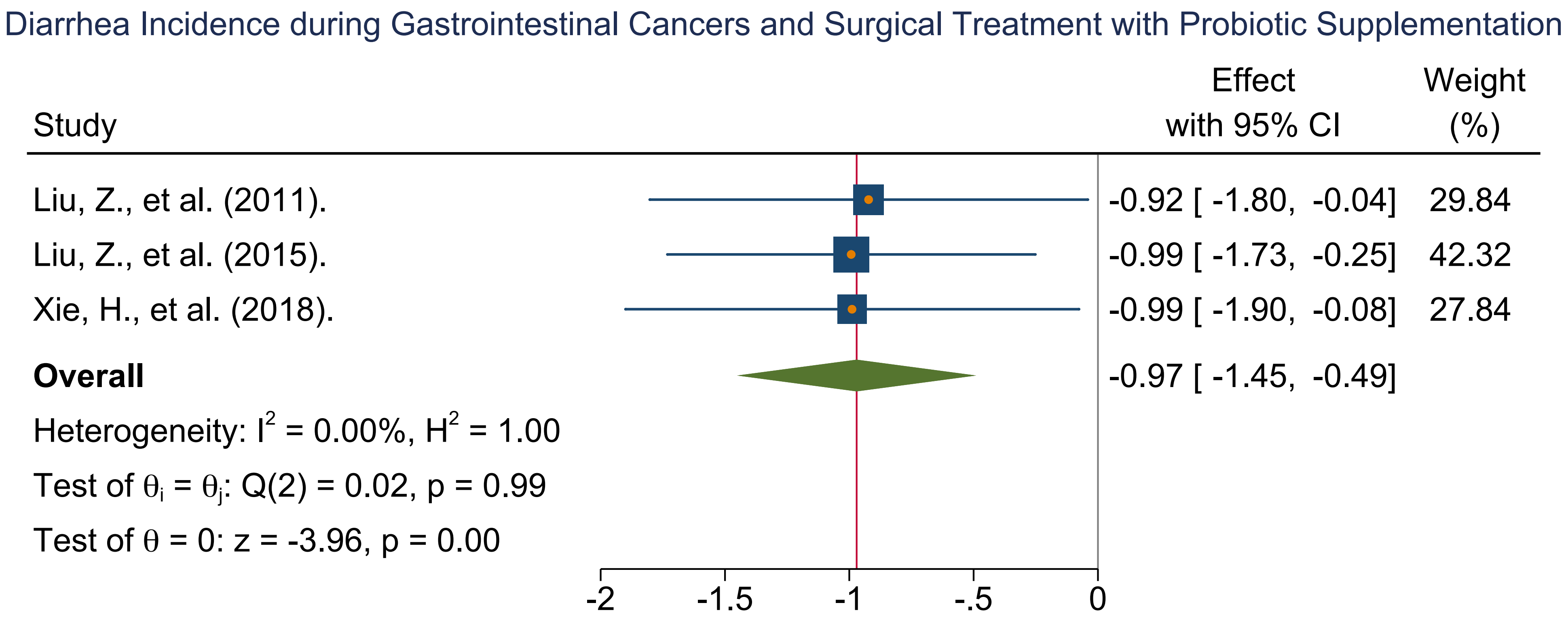
Diarrhea Incidence during Gastrointestinal Cancers and Surgical Treatment with Probiotic Supplementation
Study
Effect with 95% CI
Weight (%)
Liu, Z., et al. (2011).
-0.92 [-1.80, -0.04] 29.84
Liu, Z., et al. (2015).
-0.99 [-1.73, -0.25] 42.32
Xie, H., et al. (2018).
-0.99 [-1.90, -0.08] 27.84
Overall
-0.97 [-1.45, -0.49]
Heterogeneity: I^2 = 0.00%, H^2 = 1.00
Test of $\theta_i = \theta_j$: Q(2) = 0.02, p = 0.99
Test of θ = 0: z = -3.96, p = 0.00
-2
-1.5
-1
-.5
0

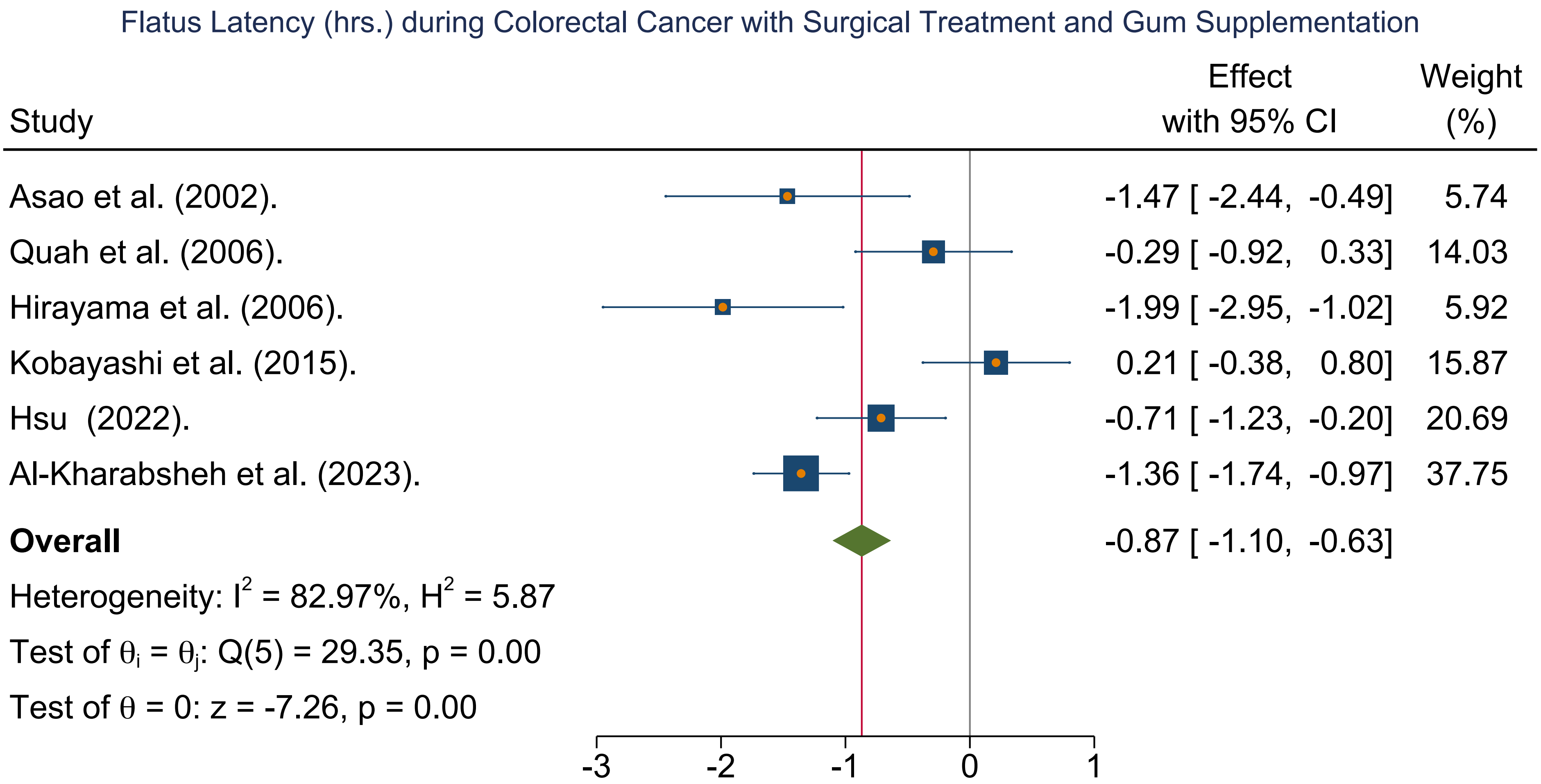
Flatus Latency (hrs.) during Colorectal Cancer with Surgical Treatment and Gum Supplementation
Study
Effect with 95% CI
Weight (%)
Asao et al. (2002). -1.47 [-2.44, -0.49] 5.74
Quah et al. (2006). -0.29 [-0.92, 0.33] 14.03
Hirayama et al. (2006). -1.99 [-2.95, -1.02] 5.92
Kobayashi et al. (2015). 0.21 [-0.38, 0.80] 15.87
Hsu (2022). -0.71 [-1.23, -0.20] 20.69
Al-Kharabsheh et al. (2023). -1.36 [-1.74, -0.97] 37.75
Overall -0.87 [-1.10, -0.63]
Heterogeneity: I^2 = 82.97%, H^2 = 5.87
Test of $\theta_i = \theta_j$: Q(5) = 29.35, p = 0.00
Test of θ = 0: z = -7.26, p = 0.00
-3
-2
-1
0
1

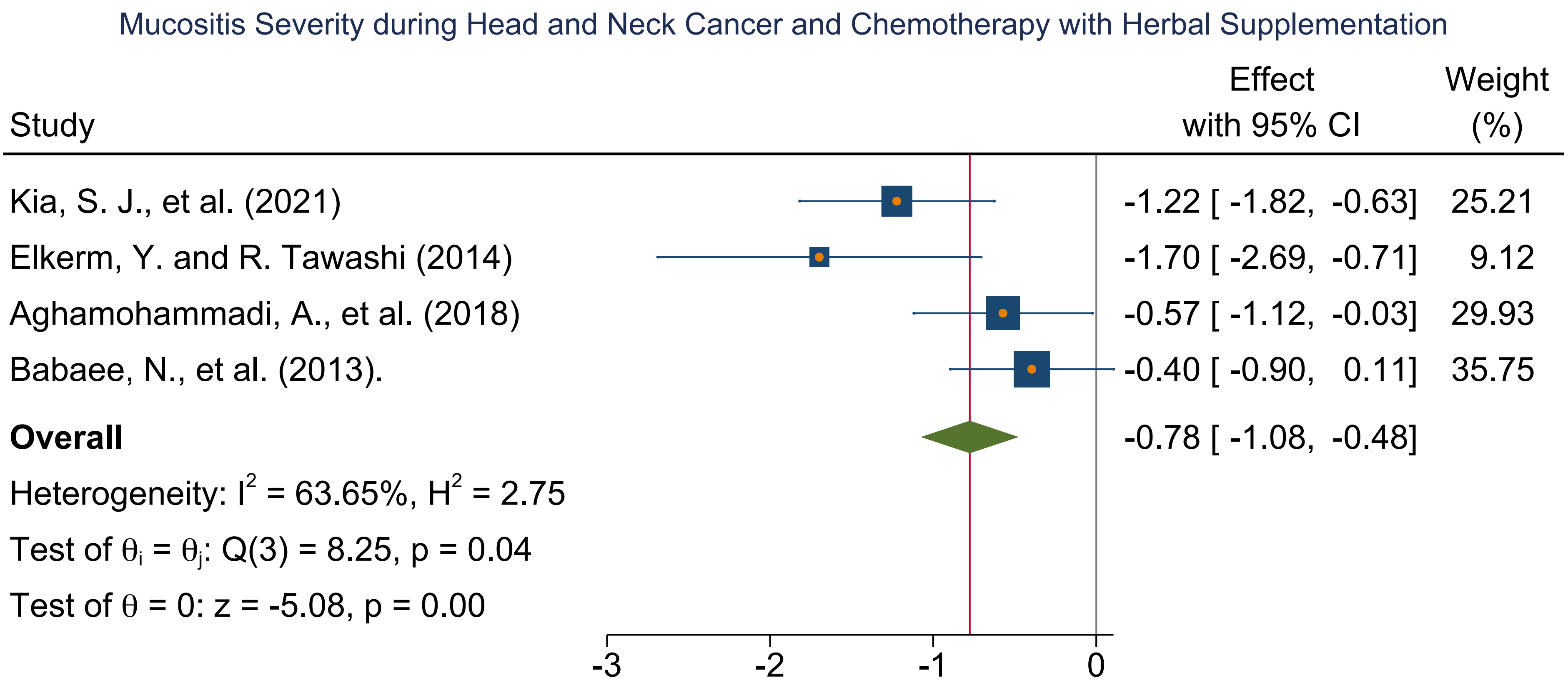

Mucositis Severity during Head and Neck Cancer and Chemotherapy with Herbal Supplementation
Study
Effect with 95% CI
Weight (%)
Kia, S. J., et al. (2021)
-1.22 [-1.82, -0.63]
25.21
Elkerm, Y. and R. Tawashi (2014)
-1.70 [-2.69, -0.71]
9.12
Aghamohammadi, A., et al. (2018)
-0.57 [-1.12, -0.03]
29.93
Babaee, N., et al. (2013).
-0.40 [-0.90, 0.11]
35.75
Overall
-0.78 [-1.08, -0.48]
Heterogeneity: I^2 = 63.65%, H^2 = 2.75
Test of $\theta_i = \theta_j$: Q(3) = 8.25, p = 0.04
Test of θ = 0: z = -5.08, p = 0.00
-3
-2
-1
0

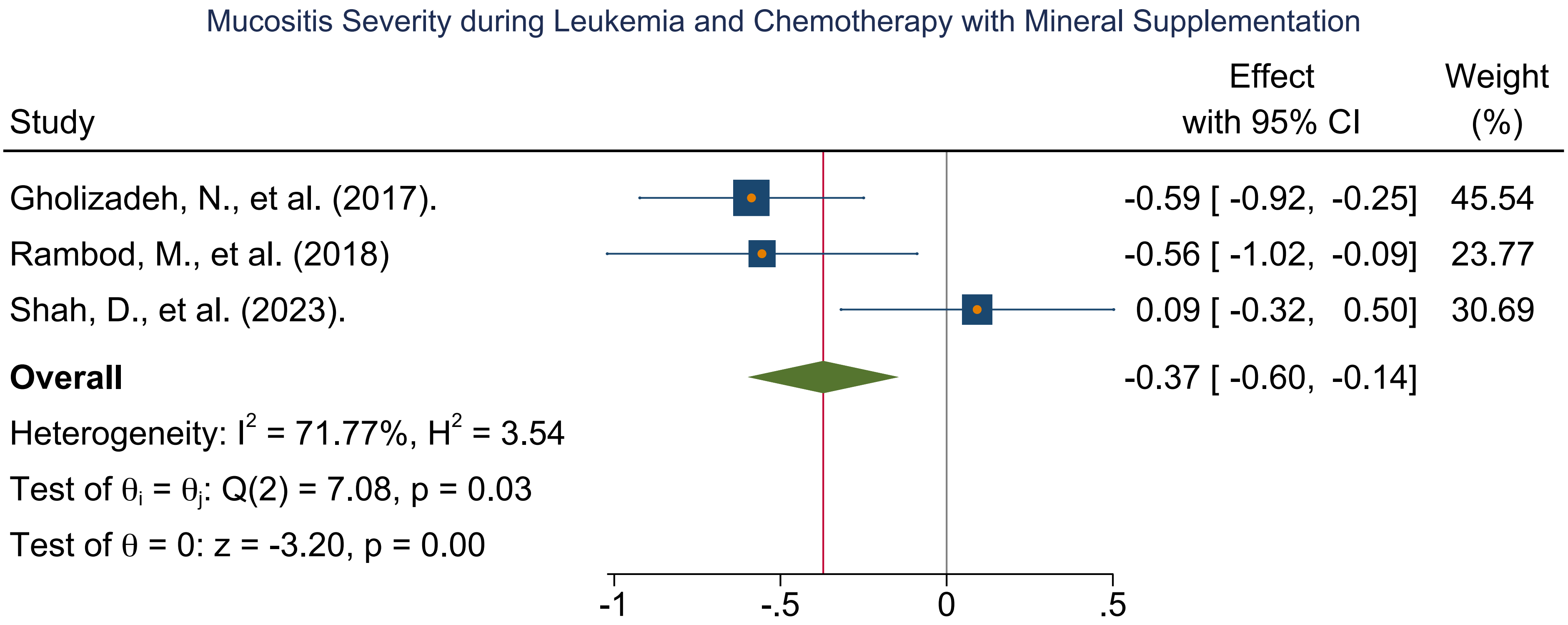

Mucositis Severity during Leukemia and Chemotherapy with Mineral Supplementation
Study
Effect with 95% CI
Weight (%)
Gholizadeh, N., et al. (2017).
-0.59 [-0.92, -0.25]
45.54
Rambod, M., et al. (2018)
-0.56 [-1.02, -0.09]
23.77
Shah, D., et al. (2023).
0.09 [-0.32, 0.50]
30.69
Overall
-0.37 [-0.60, -0.14]
Heterogeneity: I^2 = 71.77%, H^2 = 3.54
Test of $\theta_i = \theta_j$: Q(2) = 7.08, p = 0.03
Test of θ = 0: z = -3.20, p = 0.00
-1
-.5
0
.5

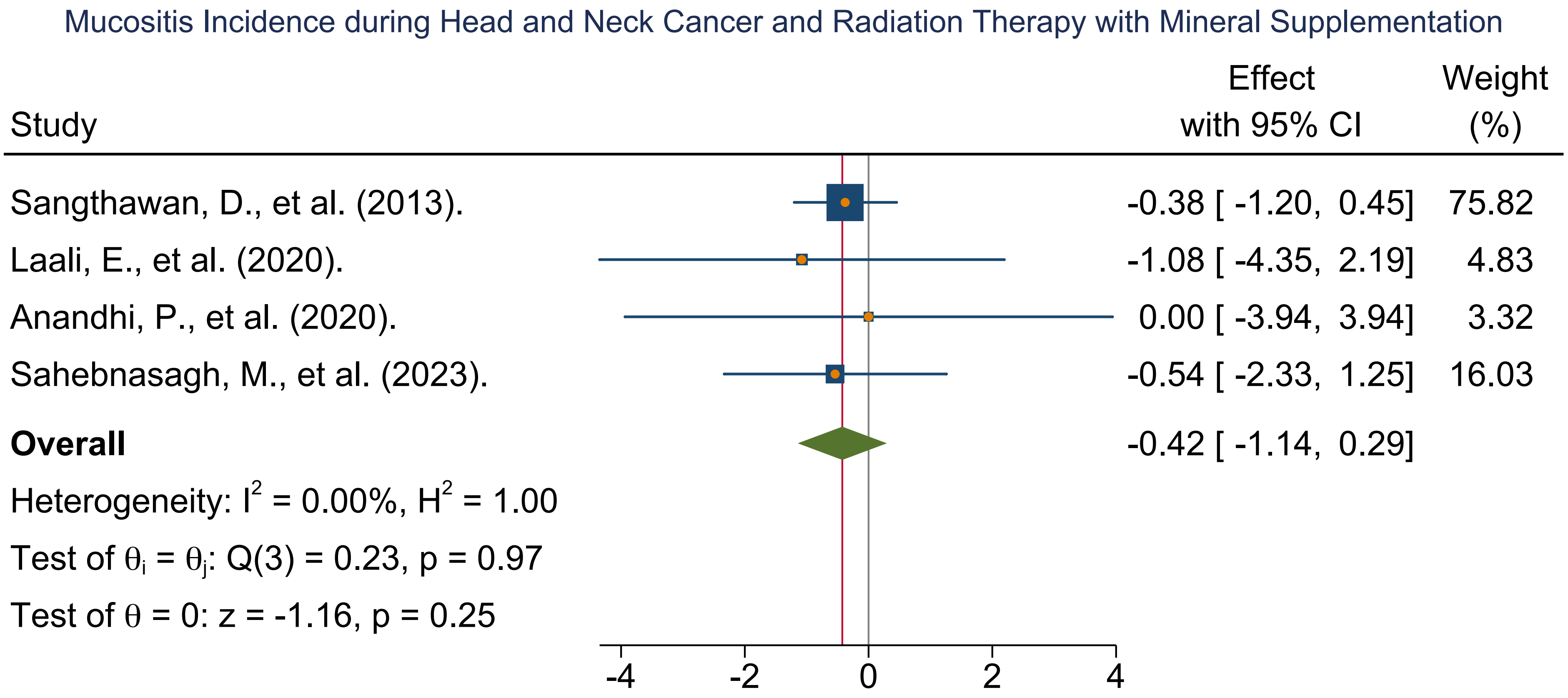
Mucositis Incidence during Head and Neck Cancer and Radiation Therapy with Mineral Supplementation
Study
Effect with 95% CI
Weight (%)
Sangthawan, D., et al. (2013). -0.38 [-1.20, 0.45] 75.82
Laali, E., et al. (2020). -1.08 [-4.35, 2.19] 4.83
Anandhi, P., et al. (2020). 0.00 [-3.94, 3.94] 3.32
Sahebnasagh, M., et al. (2023). -0.54 [-2.33, 1.25] 16.03
Overall -0.42 [-1.14, 0.29]
Heterogeneity: I^2 = 0.00%, H^2 = 1.00
Test of $\theta_i = \theta_j$: Q(3) = 0.23, p = 0.97
Test of θ = 0: z = -1.16, p = 0.25
-4
-2
0
2
4

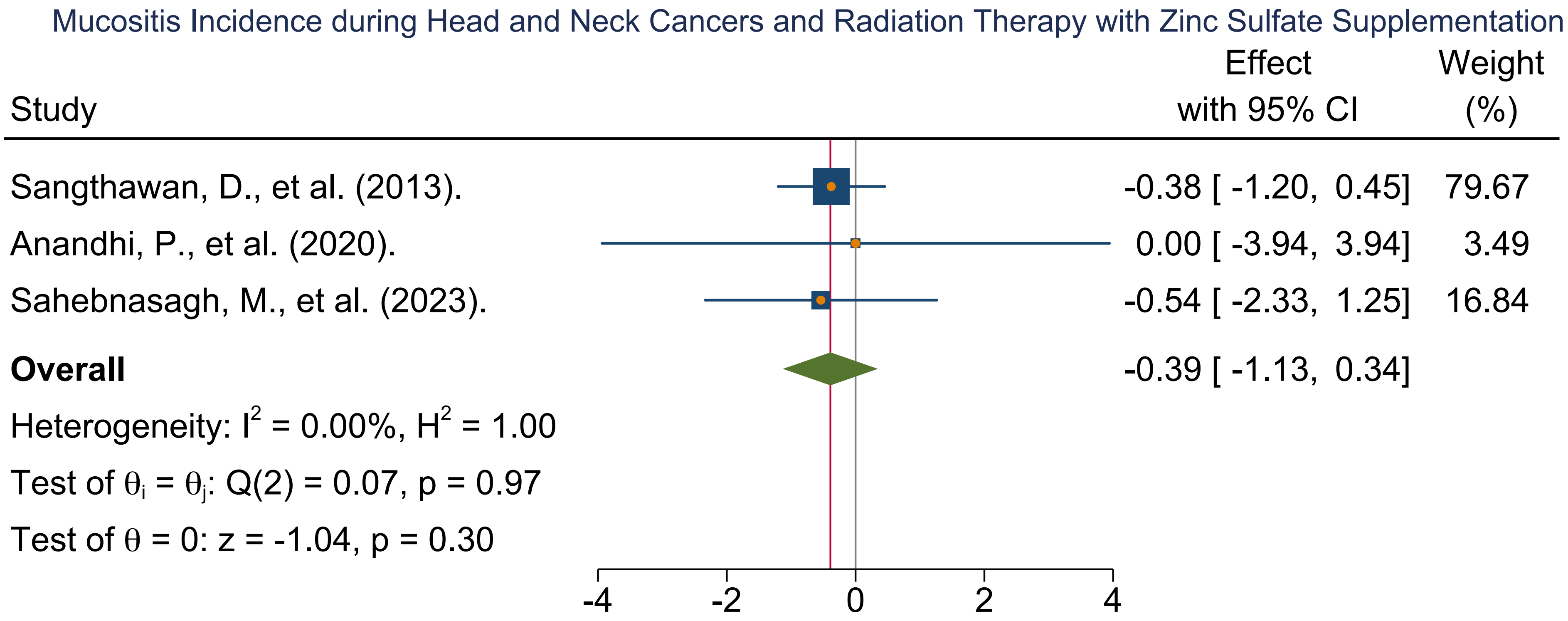

Mucositis Incidence during Head and Neck Cancers and Radiation Therapy with Zinc Sulfate Supplementation
Study
Effect with 95% CI
Weight (%)
Sangthawan, D., et al. (2013). -0.38 [-1.20, 0.45] 79.67
Anandhi, P., et al. (2020). 0.00 [-3.94, 3.94] 3.49
Sahebnasagh, M., et al. (2023). -0.54 [-2.33, 1.25] 16.84
Overall -0.39 [-1.13, 0.34]
Heterogeneity: I^2 = 0.00%, H^2 = 1.00
Test of $\theta_i = \theta_j$: Q(2) = 0.07, p = 0.97
Test of θ = 0: z = -1.04, p = 0.30
-4
-2
0
2
4

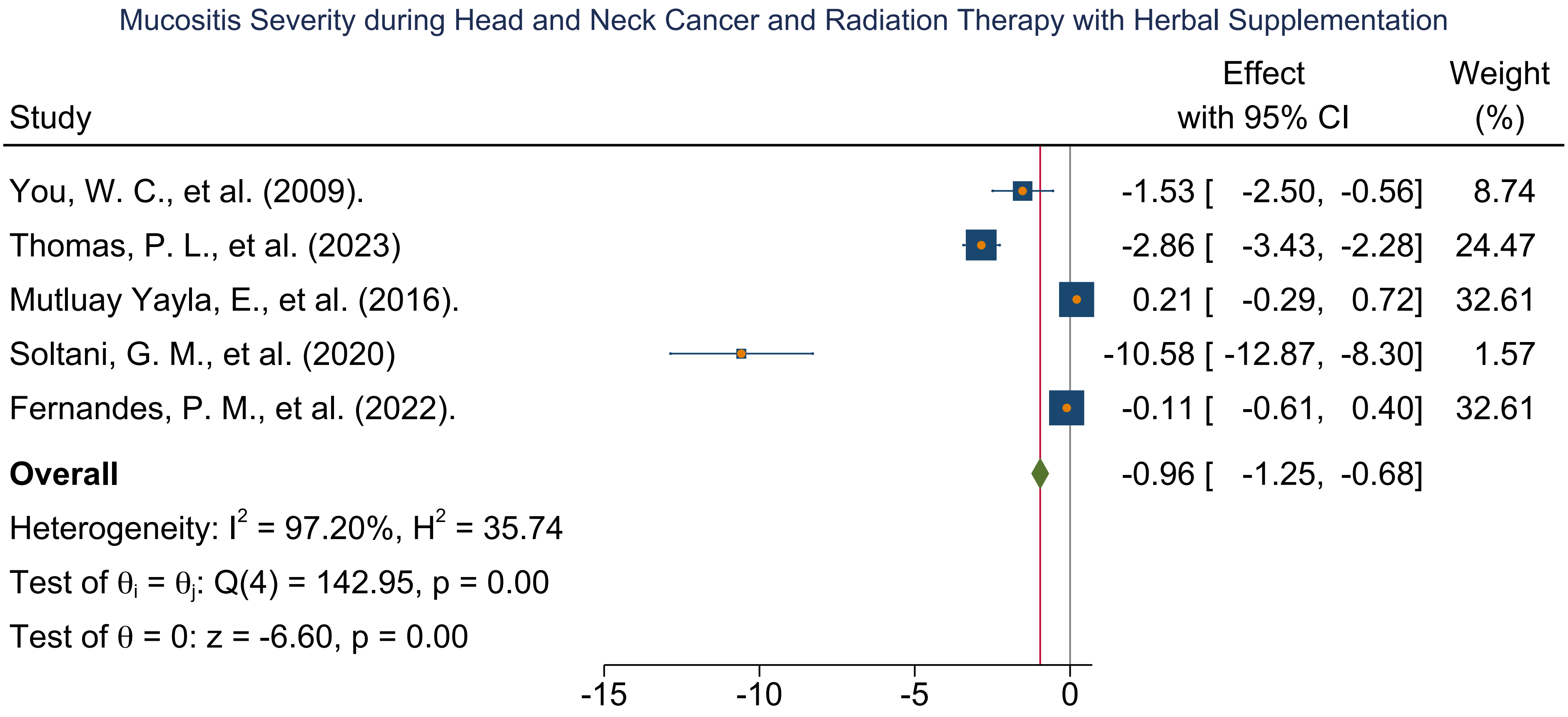
Mucositis Severity during Head and Neck Cancer and Radiation Therapy with Herbal Supplementation
Study
Effect with 95% CI
Weight (%)
You, W. C., et al. (2009). -1.53 [-2.50, -0.56] 8.74
Thomas, P. L., et al. (2023) -2.86 [-3.43, -2.28] 24.47
Mutluay Yayla, E., et al. (2016). 0.21 [-0.29, 0.72] 32.61
Soltani, G. M., et al. (2020) -10.58 [-12.87, -8.30] 1.57
Fernandes, P. M., et al. (2022). -0.11 [-0.61, 0.40] 32.61
Overall -0.96 [-1.25, -0.68]
Heterogeneity: I^2 = 97.20%, H^2 = 35.74
Test of $\theta_i = \theta_j$: Q(4) = 142.95, p = 0.00
Test of θ = 0: z = -6.60, p = 0.00
-15
-10
-5
0

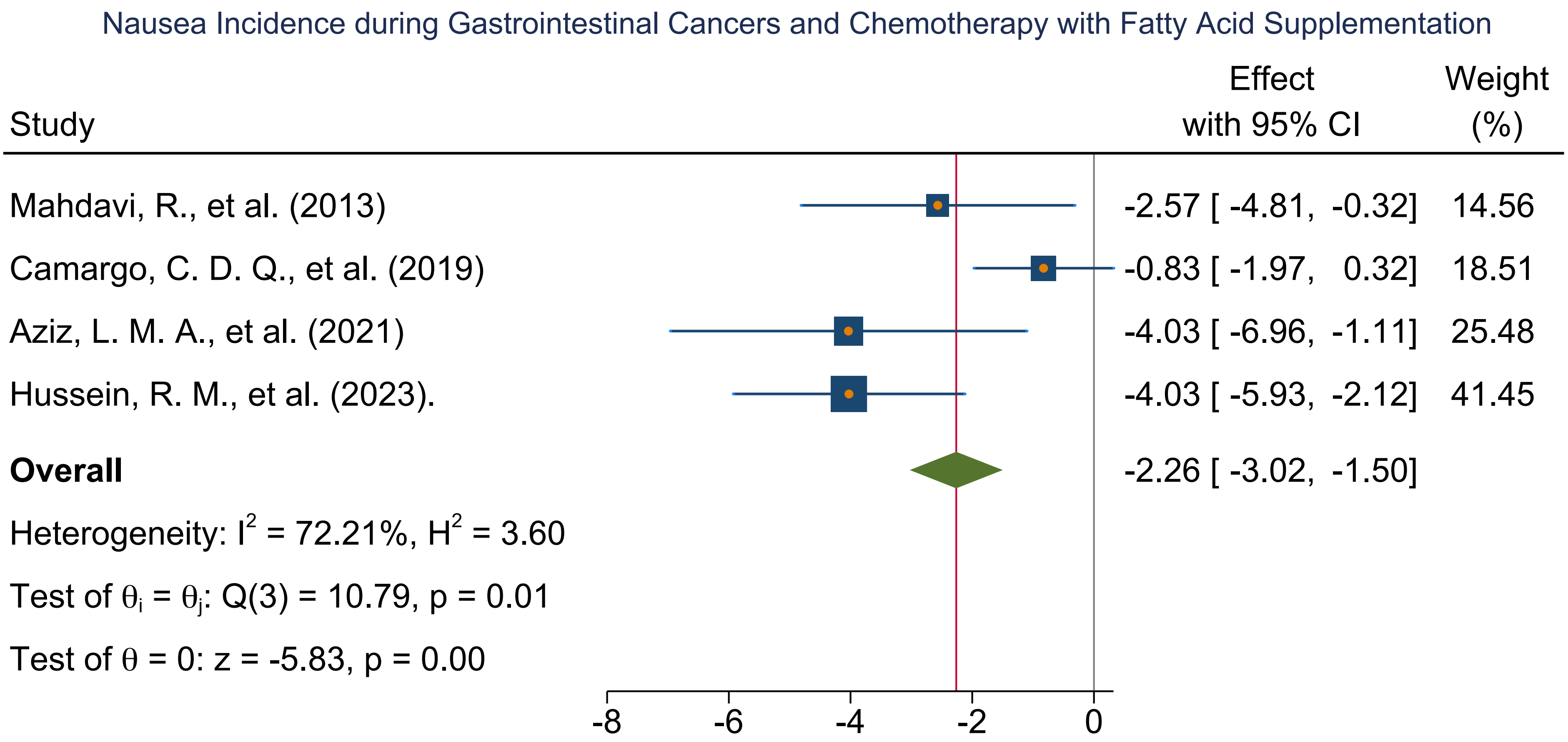
Nausea Incidence during Gastrointestinal Cancers and Chemotherapy with Fatty Acid Supplementation
Study
Effect with 95% CI
Weight (%)
Mahdavi, R., et al. (2013) -2.57 [-4.81, -0.32] 14.56
Camargo, C. D. Q., et al. (2019) -0.83 [-1.97, 0.32] 18.51
Aziz, L. M. A., et al. (2021) -4.03 [-6.96, -1.11] 25.48
Hussein, R. M., et al. (2023). -4.03 [-5.93, -2.12] 41.45
Overall -2.26 [-3.02, -1.50]
Heterogeneity: I^2 = 72.21%, H^2 = 3.60
Test of $\theta_i = \theta_j$: Q(3) = 10.79, p = 0.01
Test of θ = 0: z = -5.83, p = 0.00
-8
-6
-4
-2
0

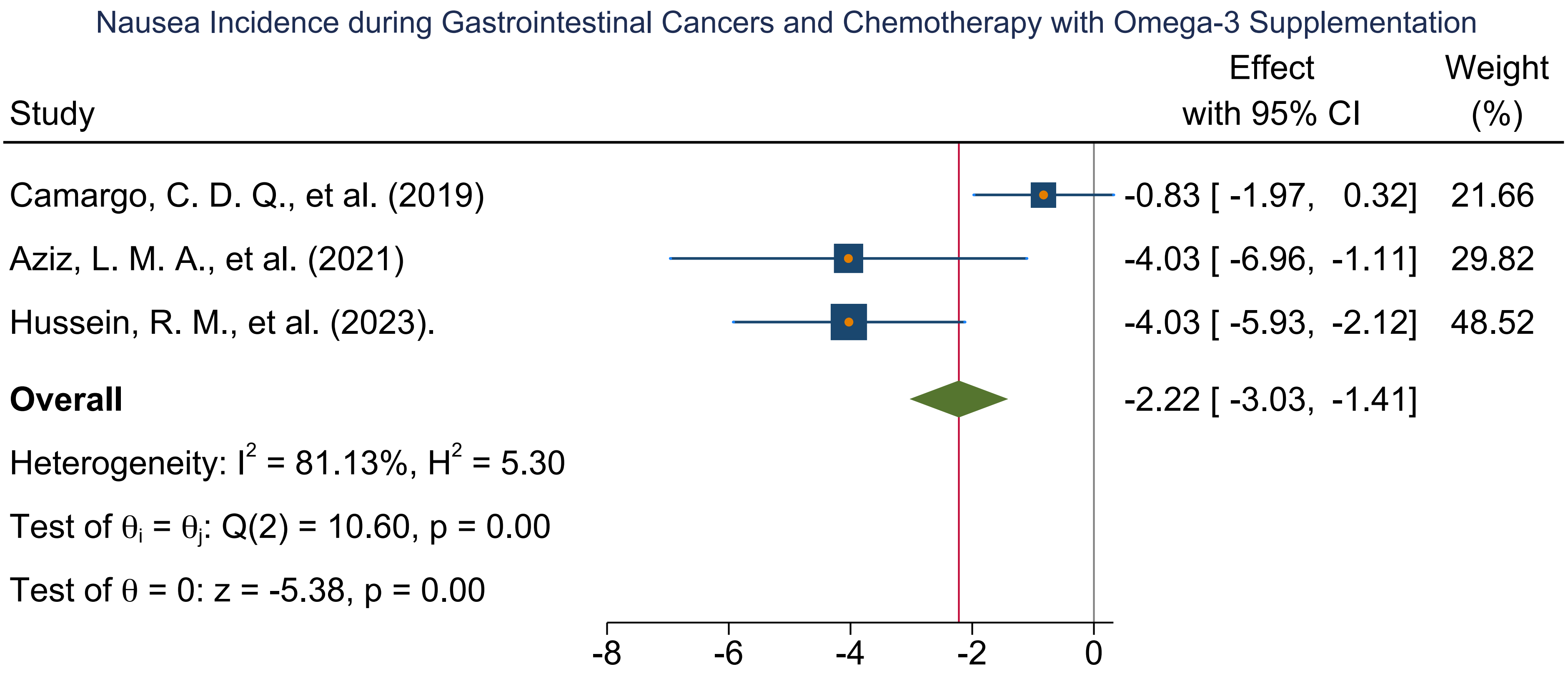
Nausea Incidence during Gastrointestinal Cancers and Chemotherapy with Omega-3 Supplementation
Study
Effect with 95% CI
Weight (%)
Camargo, C. D. Q., et al. (2019)
-0.83 [-1.97, 0.32]
21.66
Aziz, L. M. A., et al. (2021)
-4.03 [-6.96, -1.11]
29.82
Hussein, R. M., et al. (2023).
-4.03 [-5.93, -2.12]
48.52
Overall
-2.22 [-3.03, -1.41]
Heterogeneity: I^2 = 81.13%, H^2 = 5.30
Test of $\theta_i = \theta_j$: Q(2) = 10.60, p = 0.00
Test of θ = 0: z = -5.38, p = 0.00
-8
-6
-4
-2
0

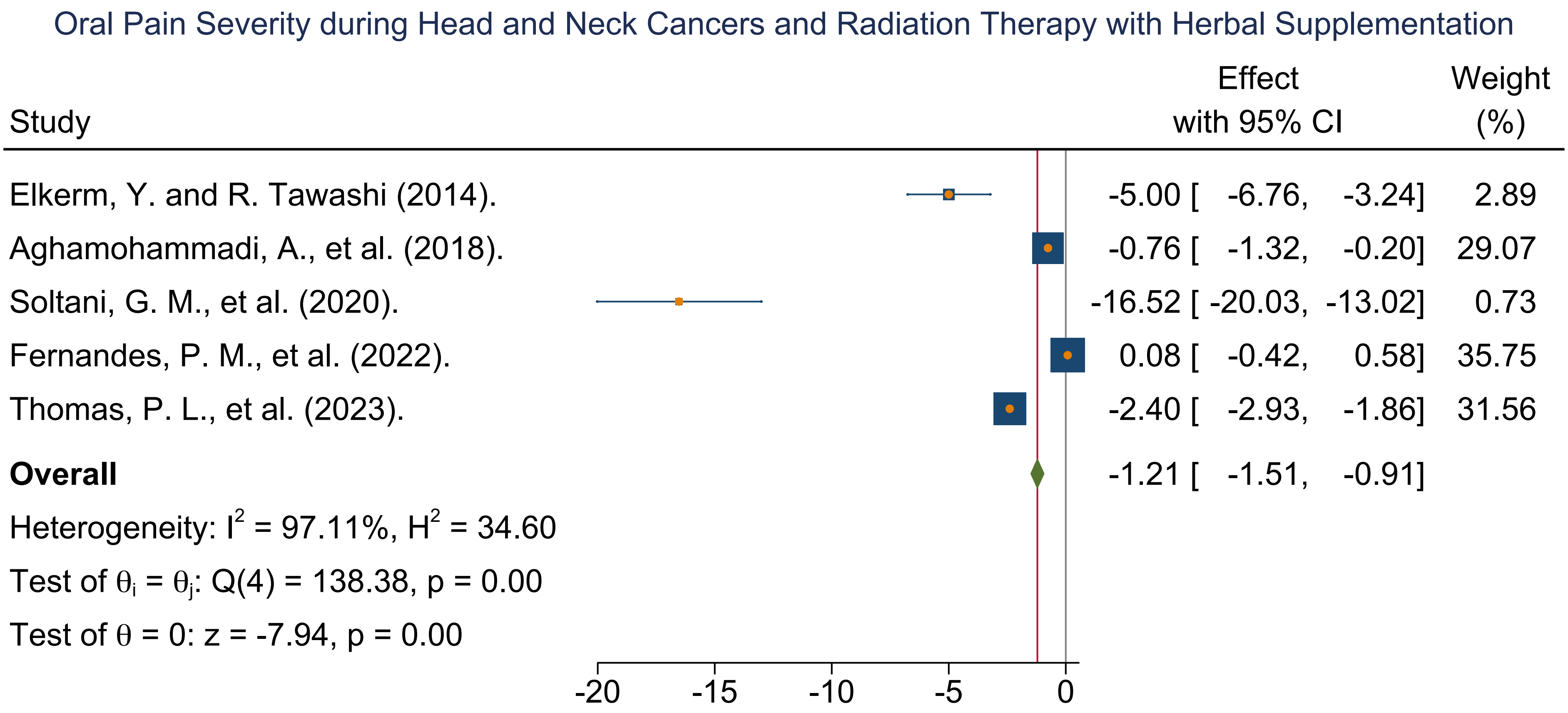
Oral Pain Severity during Head and Neck Cancers and Radiation Therapy with Herbal Supplementation
Study
Effect with 95% CI
Weight (%)
Elkerm, Y. and R. Tawashi (2014). -5.00 [-6.76, -3.24] 2.89
Aghamohammadi, A., et al. (2018). -0.76 [-1.32, -0.20] 29.07
Soltani, G. M., et al. (2020). -16.52 [-20.03, -13.02] 0.73
Fernandes, P. M., et al. (2022). 0.08 [-0.42, 0.58] 35.75
Thomas, P. L., et al. (2023). -2.40 [-2.93, -1.86] 31.56
Overall -1.21 [-1.51, -0.91]
Heterogeneity: I^2 = 97.11%, H^2 = 34.60
Test of $\theta_i = \theta_j$: Q(4) = 138.38, p = 0.00
Test of θ = 0: z = -7.94, p = 0.00
-20
-15
-10
-5
0

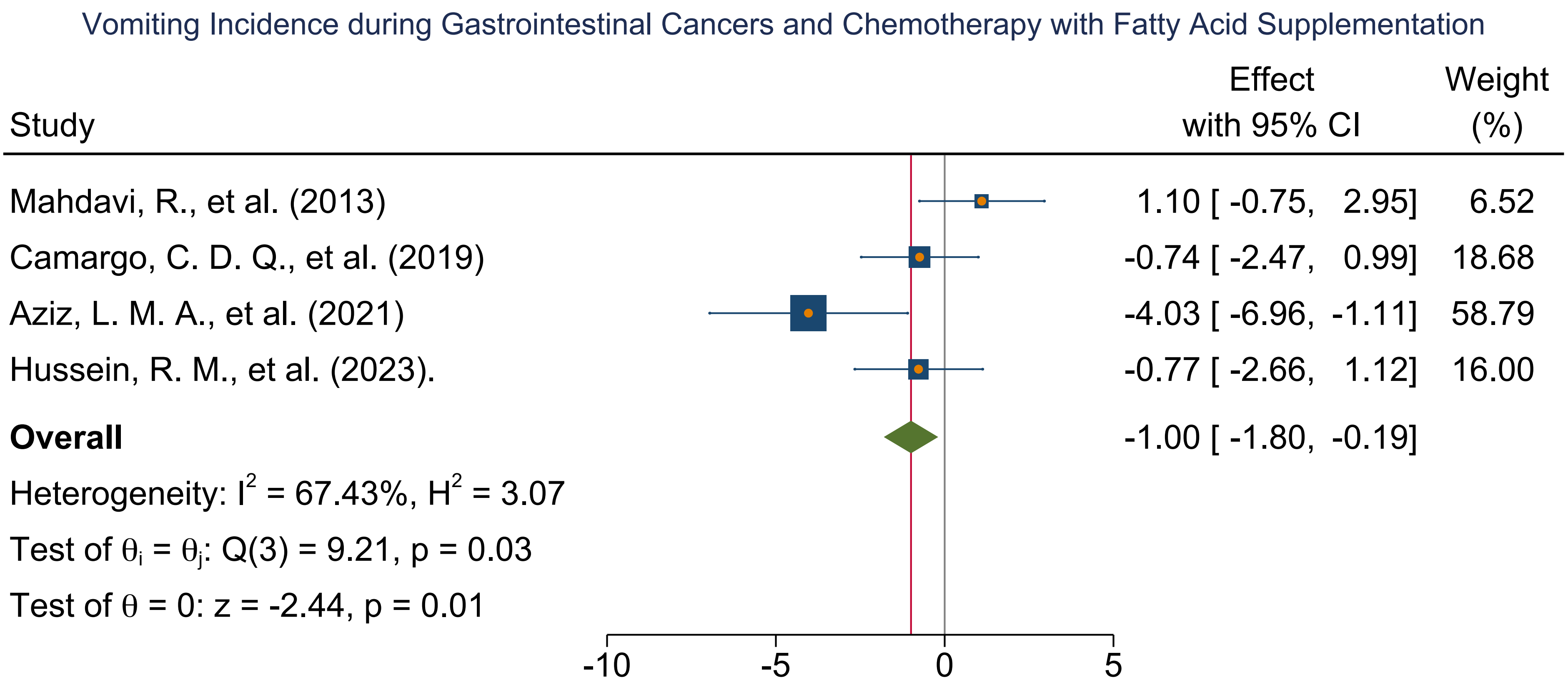
Vomiting Incidence during Gastrointestinal Cancers and Chemotherapy with Fatty Acid Supplementation
Study
Effect with 95% CI
Weight (%)
Mahdavi, R., et al. (2013) 1.10 [-0.75, 2.95] 6.52
Camargo, C. D. Q., et al. (2019) -0.74 [-2.47, 0.99] 18.68
Aziz, L. M. A., et al. (2021) -4.03 [-6.96, -1.11] 58.79
Hussein, R. M., et al. (2023). -0.77 [-2.66, 1.12] 16.00
Overall -1.00 [-1.80, -0.19]
Heterogeneity: I^2 = 67.43%, H^2 = 3.07
Test of $\theta_i = \theta_j$: Q(3) = 9.21, p = 0.03
Test of θ = 0: z = -2.44, p = 0.01
-10
-5
0
5

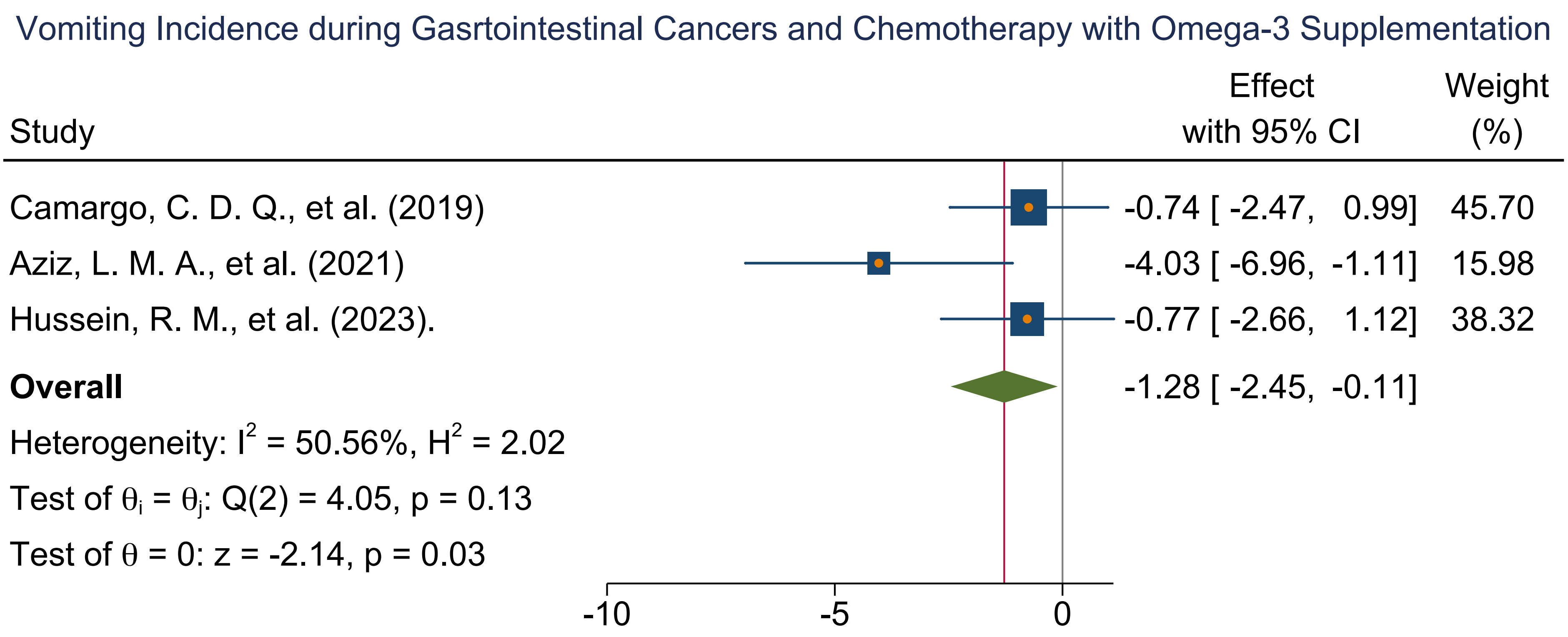
Vomiting Incidence during Gasrtointestinal Cancers and Chemotherapy with Omega-3 Supplementation
Study
Effect with 95% CI
Weight (%)
Camargo, C. D. Q., et al. (2019) -0.74 [-2.47, 0.99] 45.70
Aziz, L. M. A., et al. (2021) -4.03 [-6.96, -1.11] 15.98
Hussein, R. M., et al. (2023). -0.77 [-2.66, 1.12] 38.32
Overall -1.28 [-2.45, -0.11]
Heterogeneity: I^2 = 50.56%, H^2 = 2.02
Test of $\theta_i = \theta_j$: Q(2) = 4.05, p = 0.13
Test of θ = 0: z = -2.14, p = 0.03
-10
-5
0

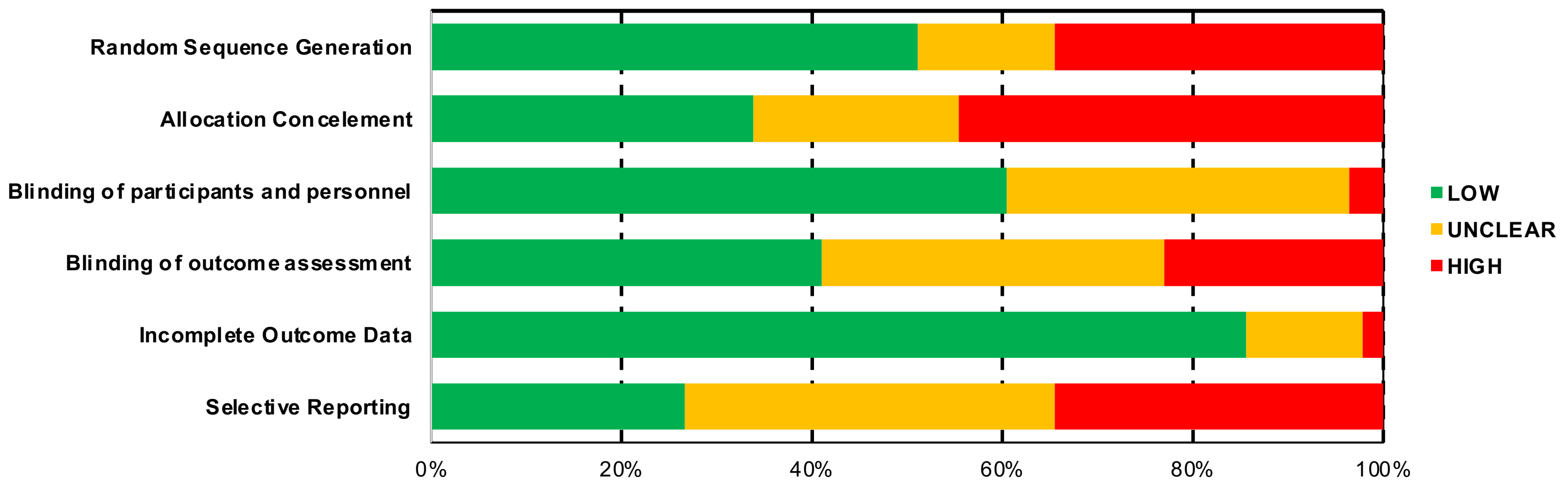

Random Sequence Generation
Allocation Con celement
Blinding of participants and personnel
Blinding of outcome assessment
Incomplete Outcome Data
Selective Reporting
0%
20%
40%
60%
80%
100%
LOW
UNCLEAR
HIGH
